# Content-rich biological network constructed by mining PubMed abstracts

**DOI:** 10.1186/1471-2105-5-147

**Published:** 2004-10-08

**Authors:** Hao Chen, Burt M Sharp

**Affiliations:** 1 Department of Pharmacology, University of Tennessee Health Science Center, Room 115 Crowe Research Building, 874 Union Avenue, Memphis, Tennessee 38163 USA

## Abstract

**Background:**

The integration of the rapidly expanding corpus of information about the genome, transcriptome, and proteome, engendered by powerful technological advances, such as microarrays, and the availability of genomic sequence from multiple species, challenges the grasp and comprehension of the scientific community. Despite the existence of text-mining methods that identify biological relationships based on the textual co-occurrence of gene/protein terms or similarities in abstract texts, knowledge of the underlying molecular connections on a large scale, which is prerequisite to understanding novel biological processes, lags far behind the accumulation of data. While computationally efficient, the co-occurrence-based approaches fail to characterize (e.g., inhibition or stimulation, directionality) biological interactions. Programs with natural language processing (NLP) capability have been created to address these limitations, however, they are in general not readily accessible to the public.

**Results:**

We present a NLP-based text-mining approach, Chilibot, which constructs content-rich relationship networks among biological concepts, genes, proteins, or drugs. Amongst its features, suggestions for new hypotheses can be generated. Lastly, we provide evidence that the connectivity of molecular networks extracted from the biological literature follows the power-law distribution, indicating scale-free topologies consistent with the results of previous experimental analyses.

**Conclusions:**

Chilibot distills scientific relationships from knowledge available throughout a wide range of biological domains and presents these in a content-rich graphical format, thus integrating general biomedical knowledge with the specialized knowledge and interests of the user. Chilibot  can be accessed free of charge to academic users.

## Background

A comprehensive understanding of the rapidly expanding corpus of information about the genome, transcriptome, and proteome at large scale requires extensive integration with existing knowledge that often pertains to a number of biological disciplines. Despite the existence of specialized databases (e.g. [1,2]), most of this knowledge is still stored in the form of unstructured free-texts. Different approaches have been developed that automatically retrieve information on molecular interactions from the biomedical literature. Some assume that the co-occurrence of gene/protein names in texts corresponds to a biological relationship [3,4]. Others assign relationships based on similarities in the texts of abstracts [5-7]. While computationally efficient, these methods do not characterize each interaction (e.g., inhibition versus stimulation, directionality). Furthermore, relationships are supported by minimal documentation, other than PubMed IDs. Natural language processing (NLP) has also been used as the basis of programs designed to retrieve more detailed information about molecular relationships ([8-11], reviewed in [12,13]). However, many of these programs were built for testing purposes and are not available to the scientific community at large [14].

**Figure 1:**
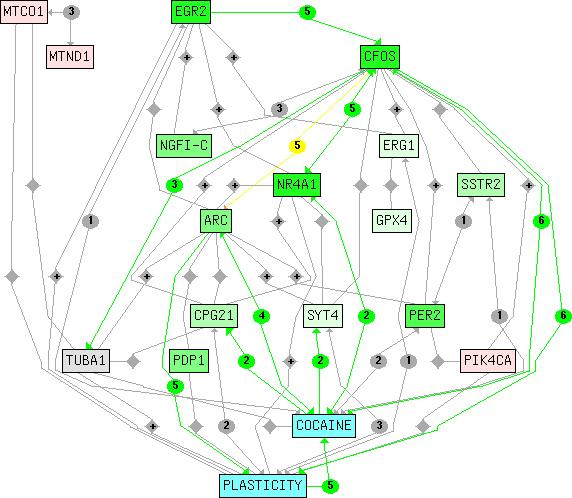
The network map of a biological network constructed by Chilibot. Chilibot queried the entire PubMed abstract database to identify a network of relationships amongst a set of genes reported to be regulated by cocaine [44], a biological concept ("plasticity"), and a drug ("cocaine"). Lines connecting rectangular nodes indicate relationships between the genes shown, and each icon in the middle of a line represents the character of the relationship. Interactive relationships (circles) are neutral (gray), stimulatory (green), inhibitory (red) or both stimulatory/inhibitory (yellow). The number within each icon indicates the quantity of abstracts retrieved for documenting that relationship. Icons containing the plus sign ("+") represent "parallel relationships". Gray rhomboidal icons indicate that only co-occurrence was detected. All arrowheads indicate the direction of the interaction, and some are bi-directional. The green or pink colors of rectangular nodes represent up- or down-regulation of the genes identified therein, respectively, based on experimental data provided by the user. More saturated colors are associated with larger changes. Nodes with no expression values (e.g., "cocaine") are in cyan. The terms and icons are linked to documentation when viewed in a web-browser. See supplementary information for subnetwork maps generated by Chilibot.

Herein, we present a text mining approach, Chilibot (chip literature robot), which constructs content-rich relationship networks between genes, proteins, drugs and biological concepts (figure 1) based on linguistic analysis of relevant records stored in the PubMed literature database. The nature of each relationship (e.g. inhibitory versus stimulative) is encoded in the network map. The network map is also annotated by sentences describing the relationships (content of the network). For example, there are an average of 24 sentences describing each relationship and 11 sentences describing each query term when a maximum of 30 abstracts are analyzed for each relationship. Thus, Chilibot provides a flexible tool for integrating the rapidly expanding body of biomedical knowledge with the highly specialized knowledge of the individual user.

Recent analyses of several types of biological networks (e.g. metabolic [15], proteomic [16], and transcriptomic [17] networks) have found that their connectivities followed the power-law distribution, specifying that the probability of any node connecting to "k" other nodes is proportional to 1/k^n^. These networks are classified as scale-free networks and are in direct contrast to the bell-shaped distributions seen in random networks [15]. Since most nodes in a scale-free network have very few connections, yet a few nodes (i.e., hubs) have a large number of connections, scale-free networks are robust, resisting the random failure of nodes, but vulnerable if hubs fail. To facilitate comparisons to the structure of other biological networks, the connectivity of networks constructed by Chilibot were analyzed and found to follow the power-law distribution characteristic of scale-free topologies.

## Results and discussion

### Design and implementation

The overall goal of Chilibot is to generate graphical representations of the relationships among user provided terms (e.g. molecules, concepts, etc). This is achieved by automatically querying the PubMed literature database and extracting information using natural language processing (NLP) techniques.

Chilibot is an Internet-based application [18]. The system has been tested on FreeBSD and Red Hat Linux operating systems. Users interact with the Chilibot server from web-browsers (e.g. Mozilla Firefox, Netscape, or Microsoft Internet Explorer). Batch queries can also be conducted, but only from the server side.

**Figure 2:**
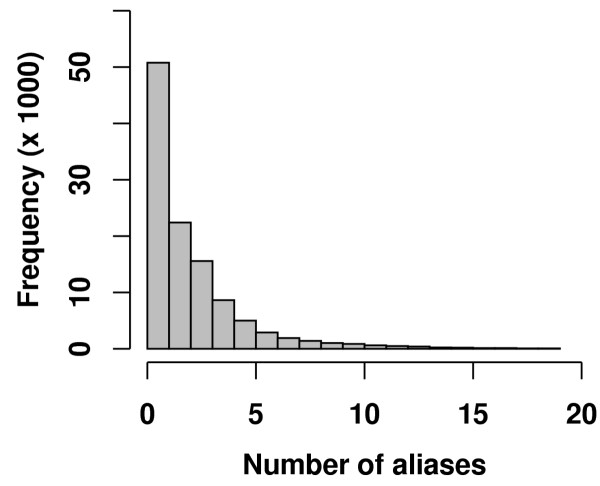
Distribution of the number of synonyms. A synonym dictionary of gene symbols was compiled from 6 databases with a total of 113,503 unique symbols. Analysis of the number of synonyms for each symbol shows that 62,178 (54.8%) had more than one.

Terms that can be queried include gene symbols, UniGene identifications (including human, rat and mouse) and/or free-form keywords (e.g. "ischemia", "apoptosis", "methylation"). Chilibot retrieves the synonyms of the queried terms from an internal database. The synonym table is compiled from 6 genomic or proteomic databases (see table 1). A total of 113,503 unique symbols were collected; amongst these, 62,178 (54.8%) contained at least one alias (figure. 2). The synonyms can be edited by users if necessary. Pair-wise queries incorporating the synonyms then are sent to PubMed using the Esearch utility, followed by retrieving relevant records using the Efetch utility. By default, a maximum of 30 abstracts per query are retrieved for analysis, however options are available to retrieve 20–50 abstracts. Both utilities are available from the National Center for Biotechnology Information (NCBI).

The texts (including each title and abstract) are then parsed into units of one sentence, which has been shown to yield higher performance levels than paragraphs or phrases in the identification of relationships from MEDLINE abstracts [19]. Sentences containing both query terms or their synonyms are subjected to part-of-speech (POS) tagging using the TnT tagger [20], which is followed by shallow parsing using CASS [21]. A set of rules (see Methods) is followed to classify these sentences into one of five categories: stimulatory (interactive), inhibitory (interactive), neutral (interactive), parallel (non-interactive) and abstract co-occurrence only. The overall relationship between each pair of query terms is then specified based on the relationships found in the sentences (see Methods).

Retrieved relationships are visualized using AiSee (AbsInt, Angewandte Informatik GmbH, Germany). Nodes (boxes) are used to represent query terms and lines for relationships. Icons with different shapes and colors are added to the middle of each line to indicate the nature of the relationship, with arrows indicating directionality. Color coding of individual nodes can be used to report the magnitude of change in experimental data, when provided by the user; different shades of green or red represent up- or down-regulation, respectively, and more saturated colors are associated with larger changes. The weight of an interactive relationship, reflecting the number of abstracts obtained from PubMed, is displayed within the icon (figure. 1). The co-ordinates of the graphical elements are used to link the documentation of the relationships and the query terms to the map. Typically, querying a list of 10 terms takes 3–4 minutes, allowing 3 seconds between PubMed connections as requested by NCBI.

### Performance evaluation

**Figure 3:**
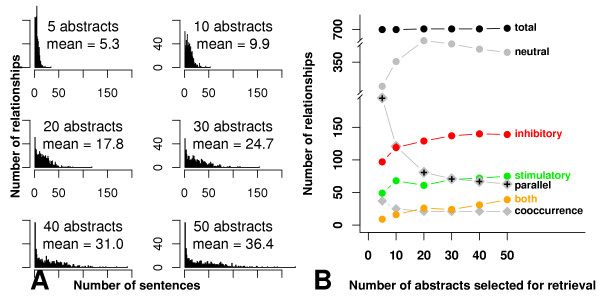
Effects of the number of abstracts obtained on retrieval, recall, and content of relationships. To measure Chilibot's level of recall, a total of 770 known relationships specified in the Database of Interacting Proteins (DIP) was used as a reference set. A. Distribution of the number of sentences describing relationships when a maximum of 5–50 abstracts were selected for retrieval. For each group, the average number of sentences documenting a relationship is reported. Of the 770 known relationships, the histograms show that an increasing number of relationships are documented by a larger number of sentences when a greater number of abstracts are specified for retrieval. B. Increasing the specified number of abstracts for retrieval from 5 to 50 had no affect on the recall of total relationships, although there were changes within relationship categories (e.g., stimulatory/inhibitory).

We used a set of 770 known relationships (see Methods) specified in the Database of Interacting Proteins (DIP) [2] to measure the performance of Chilibot in finding relationships. DIP was chosen for this purpose because it contains a large number of protein interaction relationships that are manually curated. We defined recall as the fraction of relevant relationships retrieved. The effect of the number of documents analyzed on recall is first evaluated by analyzing a maximum of 5, 10, 20, 30, 40, and 50 of the most recent abstracts for each pair of proteins. Figure 3a shows that analyzing 5 or 50 abstracts achieved recalls of 90.1% and 91.2%, respectively. Thus, increasing the number of documents analyzed does not yield an increase in recall. However, analyzing more abstracts increased the average number of statements demonstrating the relationships (figure. 3a), resulting in a steady increase in stimulatory or inhibitory relationships and a decrease in "parallel relationships" (figure. 3b). In addition, we also evaluated the effect of the total number of abstracts available in PubMed on recall. Of the 770 queries conducted by Chilibot, 66 had no reference in PubMed and no relationship was detected. Chilibot also failed to detect a relationship from two queries where each had 1 reference available. Relationships were detected among the 702 remaining queries; the number of references in PubMed ranged from less than 10 (206 queries), between 10 to 99 (299), to more than 100 (197). Thus, the ability of Chilibot to detect relationships depends on the existence of PubMed records, but is not sensitive to the number of references. Chilibot's recall proficiency may be attributable to a large dictionary of synonyms (currently from 6 databases), optimized PubMed query structure and non-alphanumeric character processing method (see Methods), and to the use of both sentences and abstracts as units of analysis. However, we were not able to directly compare the performance of Chilibot with other NLP-based PubMed-mining software because none of these are available to the public [14]. A potential approach to facilitate such performance comparisons would entail coding software according to published algorithms. However, this is not likely to replicate all details of the original software; thus, the comparisons obtained via such an approach would not be valid.

Amongst the 68 DIP relationships that Chilibot did not detect (table 2), the largest number represented a failure to recognize abstracts containing generalized protein names (e.g. PKA in PubMed abstract vs. type II-alpha form of PKA in DIP), a limitation also reported for FlyBase [22]. Recall was also limited by synonym coverage and by the presence of information in the main text, but not in the abstract. Since many of the DIP relationships were originally based on the main text of a single reference [2], the high recall of Chilibot depends on the redundancy of information in the literature.

To estimate precision, defined as the fraction of retrieved relationships that are relevant, we randomly selected 100 relationships from the 702 relationships recovered by Chilibot (86 interactive, 11 parallel, and 3 abstract co-occurrence). We manually confirmed that the documentation retrieved by Chilibot contained information about 96 of the targeted relationships, and the remaining four shared symbols with other genes. In the interactive category, directionality was correctly identified in 79.1% and inhibitory/stimulatory properties in 74.4%. The original data used to perform these analyses are available [see additional file 1 and 2].

### User interface features

One of the key features of Chilibot is its capacity to link the relationships represented in the network map directly to their supporting documentation, usually as sentences containing both of the query terms. In addition, each node is linked to its synonym list and to a set of statements demonstrating the use of the term; these statements are selected from abstract texts by an algorithm favoring conclusive statements (see Methods). By providing the literature in a condensed and highlighted form, Chilibot facilitates the rapid comprehension of the relationships by the user.

Chilibot provides several options for customizing the query process and for viewing the identified relationships. Context specific searches restrict the analysis of relationships to a specific subject area, as defined by the user. Internet searches can also be customized (e.g. searching only documents in PDF format) by using Google WebAPI. Specific subsets of relationships contained in an overall relationship map can be reconfigured. For example, the user can customize the relationship map by requesting only those relationships with direct linkage to a specific node, or those that have a requisite number of supporting publications [see additional file 3 and 4 for examples].

Chilibot also identifies key index terms common to the relationship network. To do so, Chilibot uses Medical Subject Headings (MESH) [23], a controlled vocabulary that indexes the subjects of the documents developed by the National Library of Medicine. Chilibot ranks MESH keywords indexed in the literature that supports the relationship network. The ranking is determined by the frequencies of the keywords, as well as whether the keyword is a major or minor topic of the paper (see Methods). The top ranked keywords, reflecting the subject area(s) shared by the query terms, can serve as a guide for further reading and suggest new Chilibot queries.

Chilibot also has the capability of suggesting new hypotheses based on the retrieved network of relationships. Such hypotheses, originally described by Swanson et al. as "undiscovered public knowledge" [24], referred to the inference of an interaction between two items A and C, based on knowledge that A affects B and B affects C. This involves software that generates a large list of "B" terms from titles returned by PubMed queries. The user filters these terms, aided by the titles and abstracts. Variations of this method have been designed and tested by others [25,26]. Taking a similar approach, Chilibot scans the network of retrieved relationships to find pairs of nodes that have no documented relationship, but have connections to a common tertiary node(s). These pairs of nodes are classified as having a "hypothetical relationship". The networks that contain these "hypothetical relationships", including the tertiary node(s), are then provided to the user in graphical format, with links to their documentation.

To test the value of these "hypothetical relationships" in predicting the results of future research, we queried 22 genes known to be involved in long-term potentiation (LTP), an electrophysiological phenomenon closely associated with memory formation. Chilibot identified a direct relationship between LTP and all 22 genes, along with 194 inter relationships amongst the 22 genes. We then performed retrospective studies by limiting the search to literature published before the years 2000, 1995 and 1990 [additional file 5 contains all the original search results]. The LTP-related "hypothetical relationships" identified by Chilibot, using these date-limited reference sets, are listed in table 3. As an example, by 1990, the involvement of calcium calmodulin kinase type II (CaMKII) in the induction of LTP had been established [27]. It was also known that CaMKII phosphorylates synapsin I [28,29]. Based on these and similar relationships (see table 3) that were documented in the literature available by 1990, Chilibot predicted the involvement of synapsin I in LTP, which was subsequently demonstrated empirically by 1995 [30]. Retrospective analyses like these depend on the progression of specific knowledge in scientific fields during a particular time period. Thus, if we were to test a different set of search terms, we would not expect to obtain the same number of suggested hypotheses, nor would we expect the same proportion of such hypotheses to be validated by the current literature.

Based on the literature that is currently available, Chilibot identified new hypothetical relationships, such as those between synaptophysin/CREB and synaptotagmin/CREB. Currently no direct empirical evidence for these relationships is available. However, scanning the 5' untranslated region of the synaptophysin and synaptotagmin genes did show multiple CREB binding sites, providing bioinformatics-based evidence supporting the plausibility of these potential interactions. Although these examples are promising, they are hypothetical relationships. Further review of the scientific literature, such as the sentences provided by Chilibot, is required to clarify the rationale for these hypotheses.

### Network topology of relationships retrieved from the literature

Recent large-scale studies of metabolic [15], transcriptomic [17] and proteomic [16,31] networks, based on analyses of experimental data, have found that their topologies belong within the class of scale-free networks.

**Figure 4:**
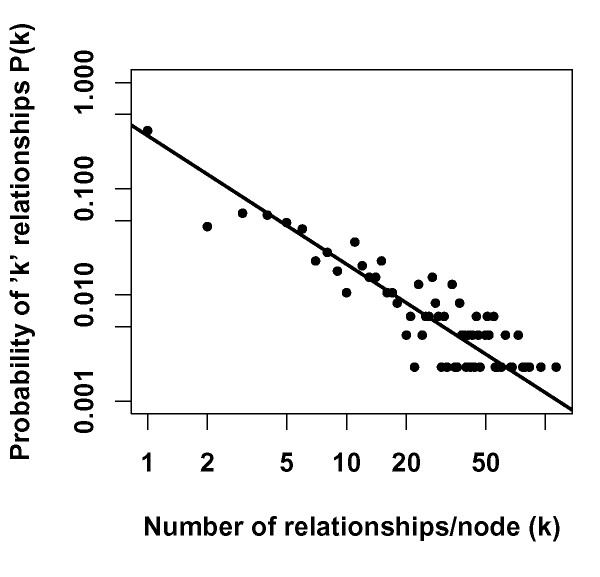
Scale-free topology of a relationship network derived from the biological literature. Chilibot was used to retrieve the relationships within 3 sets of randomly selected genes (300 genes per group). The resulting networks contain 224, 116, and 138 nodes and 3018, 962, and 1912 relationships, respectively. The distribution of the average connectivity of the 3 groups follows the power-law (P(k) ~k^-n^, n = 1.21).

For comparison to the preceding biological networks, we studied the connectivity of the literature-based networks obtained by applying Chilibot to three groups of randomly selected genes (300 genes per group). The resulting networks contain 224, 116, and 138 nodes and 3018, 962, and 1912 relationships, respectively. Visualization of the network structure of one of the groups is provided [see additional file 6]. The connectivity of the 3 groups was averaged and plotted in figure. 4, showing a power-law distribution. The relatively low value of n = 1.21 (n is approximately 2 in many of these networks [15,32,33]) may reflect the fact that many relationships are yet to be documented. In addition, we also found a positive correlation between the number of abstracts available per node and the number of connections to that node (R^2 ^= 0.76, p < 0.001). This suggests that the discovery of biological relationships attributable to specific nodes might be influenced both by the amount of scientific effort deliberately devoted to understanding that node and the intrinsic connectivity of that node. Although the commitment of greater resources by the scientific community to certain nodes may bias the topology of the scientific literature to some extent, this is likely to be regulated and limited by the strength of the findings, which would be directly related to the intrinsic connectivity of a particular node. Thus, it is reasonable to postulate that the topology of the biomedical literature on gene/protein interactions may reflect that of the interactions *per se*.

The scale-free topology of gene/protein relationships provides another dimension for comparing and prioritizing research targets after large-scale experiments. Currently, genes or proteins with large-fold changes are generally favored for further study [34]. However, by itself, a large-fold change may be insufficient to predict whether such molecules are pivotal in the regulation of important biological processes. For example, in many biological signaling pathways, a small increase in up-stream events (such as the binding of a peptide or hormone to its receptor(s)) is usually associated with a hundred to thousand-fold increase in down-stream events [35,36] (e.g., activation of mitogen-activated protein kinases or the production of cAMP). Therefore, knowledge of a network's critical nodes (i.e. hubs), which may be predicted by network connectivity [32], is likely to increase the power and efficiency of identifying potential experimental targets capable of modifying network function.

## Conclusion

Chilibot graphically summarizes the relationships amongst a large set of user provided terms by analyzing abstracts retrieved from the PubMed literature database. We have found in our benchmark tests that these retrieved relationships are reliable. We believe that the scientific community will benefit from this literature mining capability along with the many features that Chilibot provides, especially in an era of science when insight can be submerged in an overwhelming sea of data and modularized knowledge.

## Methods

### Constructing the nomenclature dictionary

Flat text file versions of the six databases (HUGO, LocusLink, OMIM, GDB, SwissProt, and SGD) were downloaded from their corresponding ftp sites. Symbol-name pairs were extracted from the corresponding fields using Perl scripts. Names were curated to remove words that are unlikely to be used in texts, such as "partial cDNA", "fragment", etc. In addition, non-alphanumerical characters were converted into spaces. Entries with the same symbol from the six databases were then combined in a case insensitive manner. The final dictionary is stored in the Postgresql relational database.

### Optimization of PubMed querying method

The NCBI Eutilies, in particular Esearch and Efetch, are used in conjunction with the Perl LWP module to interact with the  server. Optimization was necessary because phrase or adjacency searches are not supported by PubMed. Thus, when searching for names with multiple words, it is possible to retrieve abstracts that contain all the relevant words, however the words are used in different places of the abstract. Further, PubMed has an automatic term mapping feature that converts user input according to the MESH translation table. For our purposes, we considered this an undesirable feature. After small scale testing, the query structure we selected places a title and abstract restriction tag ([tiab]) after the name of the query term. This disables the term translation feature and also treats the term as a phrase when possible, according to PubMed documentation. To test the effectiveness of this strategy, we sampled 510 names with lengths ranging from 1 to 11 words. A total of 4584 abstracts were retrieved. We were able to find the query name from 4487 (97.9%) of the abstracts. We thus constructed the pair-wise PubMed query in the following format:

(Term 1 synonym 1 [tiab] OR Term 1 synonym 2 [tiab] OR ...) AND (Term 2 synonym 1 [tiab] OR Term 2 synonym 2 [tiab] OR ...)

### Acronym disambiguity

Many methods [e.g. [37-40]] have been developed to translate acronyms unambiguously into their full length terminology, since acronyms may have multiple meanings and become a source of false positives [3,41]. Chilibot provides an option to verify the meaning of acronyms when they are used as the query term. When a relevant acronym first appears, Chilibot retains a phrase immediately preceding the acronym that contains the same number of words as the number of characters in the acronym. The phrase then is compared to all synonyms of the acronym, which are retrieved from the nomenclature database of Chilibot. The abstract is excluded from analysis if less than 30% of the words in the phrase are found in the synonym list.

### Context sensitive search

All the context keywords provided by the user are combined with an "OR" operation. This string is then combined with the pair-wise PubMed queries, using an "AND" operation. The context keywords are not used in subsequent analyses.

### Synopsis generation

A synopsis is a collection of sentences used to annotate the query terms. It is generated from the first 100 sentences that contain the specific query term or its synonyms. These sentences are sorted by a weighting mechanism that favors short, conclusive sentences. Words suggesting a conclusion, such as "suggest", "found", "show", "data" etc weights as +9 points. Starting the sentence with the query term and a verb weights as +5 points. The presence of words suggesting a negative result such as "not", "lack", "fail", "without" is weighted as -3 points. Having more than 30 words also reduces the weight by 3 points. Lastly, having keywords specified by the user adds 5 points to the weight. The 15 sentences with the highest weights are displayed.

### Natural language processing

Title and abstract texts retrieved via the Efetch utility are first parsed into individual sentences using a Perl script. Only sentences containing both of the query terms or their synonyms are subjected to NLP analysis, which includes POS tagging by the TnT software [20] and shallow parsing by the CASS software [21]. Testing TnT on a small corpus of 10 PubMed abstracts (2646 words), using the supplied WSJ language model, showed 537 (20.29%) unknown words. Manual inspection identified 150 errors in the assigned POS tags. We then trained the TnT software with the GENIA corpus [42] (a collection of 2000 PubMed abstracts annotated with POS and other information). Re-analyzing the same 2646 words, using the customized language model, resulted in only 289 (10.92%) unknown words. Manual inspection identified 31 errors. Thus, the language model based on the GENIA corpus was used for all subsequent analyses. CASS software was used without further adjustment.

### Classification of relationships

All sentences containing two query terms (or their synonyms) are classified into one of six categories: stimulatory (interactive), inhibitory (interactive), both stimulatory and inhibitory (interactive), neutral (interactive), parallel (non-interactive) and abstract co-occurrence only. Sentences are classified into interactive or non-interactive relationships based on the presence or absence of a verb phrase between the two query terms. The following exceptions apply: sentences are classified as parallel when the query terms are present in two separate clauses; sentences without a verb phrase between the query terms, but with specific terms indicating interactions such as "interaction", "bind", etc., are classified as interactive; interactive relationships are converted into parallel relationship when there is a negation (such as "not") within the same clause of the verb phrase. The interactive relationship is further classified into stimulatory, inhibitory, or neutral subtypes based on the presence or absence of words describing such relationships, including "activate", "facilitate", "increase", "induce", "stimulate", "enhance", "elevate", "inactivate", "abolish", "attenuate", "block", "decrease", "eliminate", "inhibit", "reduce", "suppress". For interactive relationships, the direction is defined as from the left query term to the right term and is reversed when passive voice is detected. To avoid the influence by spurious mistakes, the overall relationship between two terms is defined as interactive only when more than 20% of the sentences are detected as either stimulatory or inhibitory. Lastly, the co-occurrence type is assigned when the two query terms are located in the same abstract but not the same sentence. We ranked the informativeness of the relationships in the following order: both stimulatory and inhibitory, either stimulatory or inhibitory, neutral interactive, parallel, abstract co-occurrence. The overall relationship between two query terms is classified as the most informative type of relationship.

### Visualization of the networks

Network layout is generated using the aiSee software. Each pair of query terms identified as having relationships is specified by nodes and represented by square boxes. The relationships are represented by solid lines. A special node with unique identification (an icon) is inserted into the middle of each line. The icon is either circular or rhomboidal depending on the relationship it represents (see legend of Figure 1). The network map as well as the links from the map to the descriptions of the relationships are obtained by calling the command line interface of aiSee.

### "Hypothetical relationship" generation and testing

After the query session is finished, the user can request Chilibot to suggest hypothetical relationships for any node that is within the retrieved network. For each node requested (NR) by the user, Chilibot scans the retrieved network to find those nodes that are not directly linked to NR, but have connections to the same tertiary nodes as NR. Chilibot then produces a new network map for each of these "hypothetical relationships", while maintaining the links to the supporting documentation. To test the usefulness of these "hypothetical relationships" in predicting future research, a total of 22 terms (ACTIN, ACTININ, AMPA, ARC, ATF, CAMKII, CAMKIV, CREB, ERK, KV4.2, NMDA, PI-3K, PKA, PKC, PLC, SYNAPSIN I, SYNAPTOPHYSIN, SYNAPTOTAGMIN, TAU, TRKA, TRKB, AND ZIF268) were queried together with LTP (long-term potentiation). Retrospective studies were performed by querying these terms again while adding the PubMed date limiting tag "&mindate=1960&maxdate=$maxdate", where the $maxdate equals to 1990, 1995, 2000, respectively.

### MESH themes

The MESH Keywords of the abstracts represented by the graph are collected and sorted by their weighted percentage. When the keyword is the major topic of the publication, it is weighted as 3. Otherwise, it is weighted as 1. The weights are then divided by the number of abstracts to obtain the weighted percentage.

### Web search and content filtering

Google WebAPI is accessed through Perl scripts. Due to the limitation of the WebAPI, the query terms are searched directly without the expanded synonyms. The URIs of the top 10 hits were retrieved from Google and then the content of these pages was obtained from their individual servers. These pages are then converted into texts, and sentences containing either one of the query terms are presented to the user. Sentences containing both of the query terms are highlighted. Links are also provided to restrict the web search to educational institutions or to files in the portable document format (PDF). Google is a trademark of Google Technology, Inc.

### Selection of relationships from the Database of Interacting Proteins (DIP)

DIP [2] is a curated protein interaction database. The version of DIP database released on April 18th, 2003 contains 18494 interactions between 7141 proteins. Relationships that originated from large scale genomic or proteomic studies were excluded, reflecting poor reliability of the data [43] and the low probability that such interactions would be described in textual forms. Proteins with no SwissProt annotation or of yeast origin were also excluded to further reduce the number of relationships to a manageable subset. This selection procedure resulted in a total of 770 relationships.

## Authors' contributions

HC conceived of the project (together with BMS), coded the Chilibot program, performed the evaluations and drafted the manuscript. BMS conceived of the project (together with HC), participated in its design, coordination and analysis, and edited and revised the manuscript.

## Note

All none-graphic files are archived with tar and compressed with bzip2 to reduce file size.

## Supplementary Material

Additional File 1 A total of 770 known relationships were used to test the recall and precision of Chilibot. A maximum of 5, 10, 20, 30, 40, or 50 most recent PubMed records for each relationship was specified for analysis. The relationships identified by Chilibot are summarized and provided in Microsoft Excel and OpenOffice format. Click here for file

Additional File 2 The original results of the above study (non-essential files are deleted to keep the file size under the limit set by BMC bioinformatics). Click here for file

Additional File 3 Sub-network graph obtained by filtering figure 1 using the number of supporting publications as a threshold criterion. Click here for file

Additional File 4 Sub-network graph obtained by filtering figure 1 to selectively display a node of interest (i.e. "cocaine") and other nodes that directly connected to it. Click here for file

Additional File 5 The original Chilibot query results of the term "long-term potentiation (LTP)" and 22 other terms, limiting the latest references analyzed to the years 1990, 1995, 2000, and 2004. Click here for file

Additional File 6 A graph demonstrating the scale-free topology of relationship networks derived from the biological literature. The network contains 138 nodes and 1912 relationships. A small fraction of the nodes (10 nodes colored in black) accounted for more than 45% of the relationships (solid lines), a characteristic of scale-free topology. Click here for file

## Figures and Tables

**Table 1:** Chilibot dictionary of gene/protein synonyms.

Database	Number of gene symbols collected
SwissProt	84462
LocusLink	23924
GDB	15770
HUGO	15905
OMIM	8291
SGD	4325

Flat text file versions of the six databases were downloaded from their corresponding ftp sites. Synonym pairs were extracted from the corresponding fields and entries with the same symbol from the six databases were then combined in a case insensitive manner. (HUGO: Human Genome Organization; OMIM: Online Mendelian Inheritance in Man; SGD: Saccharomyces Genome Database)

**Table 2:** Failure to detect known DIP relationships

Reason	Undetected relationships (%)
member of protein family (name generalization)	30.8
incomplete synonym list	26.5
no reference at abstract level	22.1
other	20.6

Chilibot was used to retrieve information about 770 pairs of known protein interactions obtained from the Database of Interacting Proteins (DIP). A total of 702 relationships were found (recall = 91.2%). Relationships were undetectable (n = 68) for the following reasons: 21 (30.8%) occurred when a specific member of the protein family (e.g. cdc25a) was recorded in DIP, yet only the general family name (e.g. cdc25) appeared in abstracts; 18 (26.5%) were due to synonyms present in abstracts and not in Chilibot's dictionary of nomenclature; 15 (22.1%) were caused by lack of documentation of the relationships in PubMed abstracts. Miscellaneous reasons accounted for the remainder (20.6%).

**Table 3:** Retrospective study of the predictive capability of the "hypothetical relationships" generated by Chilibot

Term 1	Tertiary nodes	Term 2	References Analyzed	Relationship Documented
LTP	PI-3K PKA CAMKII ACTIN ERK TAU PKC AMPA	KV4.2	1960~2000	2001~2004
LTP	PKA ACTIN SYNAPTOPHYSIN PKC NMDA TAU AMPA PLC	ERK	1960~1995	1996~2000
LTP	PKA ACTIN NMDA TAU AMPA PLC	ARC	1960~1995	1996~2000
LTP	PKA ACTIN CREB PKC PLC	PI-3K	1960~1995	1996~2000
LTP	PKA ACTIN SYNAPTOPHYSIN PKC TAU	ACTININ	1960~1995	1996~2000
LTP	PKA CAMKII CREB PKC TAU	ATF	1960~1995	1996~2000
LTP	ZIF268 PKC NMDA PLC	TRKB	1960~1995	1996~2000
LTP	PKA CAMKII CREB	CAMKIV	1960~1995	1996~2000
LTP	ZIF268 PLC	TRKA	1960~1995	1996~2000
LTP	NMDA TAU PLC ACTIN	ARC	1960~1990	1996~2000
LTP	PKC TAU CAMKII ACTIN	SYNAPSIN I	1960~1990	1991~1995
LTP	PKC NMDA TAU ACTIN	PKA	1960~1990	1991~1995
LTP	TAU ACTIN	SYNAPTOPHYSIN	1960~1990	1991~1995
LTP	TAU ACTIN	ACTININ	1960~1990	1996~2000
LTP	ACTIN	ZIF268	1960~1990	1991~1995

A "hypothetical relationship" is defined when two terms have no documented relationship, but share connections to the same tertiary node(s). To test the value of these relationships in predicting the findings of future research, 22 terms (i.e., term 2; see methods) known to be involved in long-term potentiation (LTP) (i.e. term 1) were queried by Chilibot, limiting the latest references analyzed to the years 1990, 1995, 2000, and 2004. The "hypothetical relationships" (i.e. term 1 is related to term 2) and the time periods when these hypothetical relationships were suggested and documented are listed. The original results of these searches including links to the PubMed abstracts are available [see additional file 5].
