# Supplementary material for: Content-rich biological network constructed by mining PubMed abstracts
Source: BMC Bioinformatics. 2004 Oct 8;5:147. doi: 10.1186/1471-2105-5-147 (PMC528731; doi:10.1186/1471-2105-5-147)
Supplement: Additional File 2 — The original results of the above study (non-essential files are deleted to keep the file size under the limit set by BMC bioinformatics). [file 1471-2105-5-147-S2.bz2 › chilibotAdditionalFile2/dip05/1ID9194558E1/html/BAX_BCL2L1.html]

 


 **BAX** and **BCL2L1** 
  
Found 1196 abstracts in PubMed, retrieved 05.  
 

 What does Google say? 
 PDF only 
| .edu only 

---

**Interactive relationship** (e.g. stimulation, inhibition, etc)

**Inhibitory relationship**- A small inhibitor of the interaction between  **Bax**  and Bcl X  [ **BCL2L1** ]  L can synergize with methylprednisolone to induce apoptosis in Bcl X  [ **BCL2L1** ]  L overexpressing breast cancer cells.  Ref: 12884026 J Cancer Res Clin OncolJ Cancer Res Clin Oncol,
- We have identified a small inhibitor of the interaction between  **Bax**  and Bcl X  [ **BCL2L1** ]  L that can synergize with methylprednisolone to induce apoptosis in Bcl X  [ **BCL2L1** ]  L overexpressing breast cancer cells.  Ref: 12884026 J Cancer Res Clin OncolJ Cancer Res Clin Oncol,
**Neutral relationship**- Using an assay based on biosensor technology, we screened a chemical library of 1 00 compounds for inhibitors of the interaction between  **Bax**  and Bcl X  [ **BCL2L1** ]  L.  Ref: 12884026 J Cancer Res Clin OncolJ Cancer Res Clin Oncol,
- To identify inhibitors of the interaction between  **Bax**  and Bcl X  [ **BCL2L1** ]  L.  Ref: 12884026 J Cancer Res Clin OncolJ Cancer Res Clin Oncol,

**Non-interactive relationship** (e.g. studied together, co-existance, homology, etc.)

- AIMS To correlate the expression of a series of apoptotic and oncogene markers including p53, Bcl 2,  **BAX** , Bcl XL  [ **BCL2L1** ] , p21WAF, 1 CIP1, cyclin D1, HER 2 neu in thymic epithelial tumours with histological type, stage and resectability and to determine whether the information on HER 2 neu would be valuable in identifying patients who are eligible for anti HER 2 neu treatment.  Ref: 12877732 Histopathology, 2003
- There was no difference found in the expression of proapoptotic  **Bax**  and Bcl X  [ **BCL2L1** ]  s or antiapoptotic Bcl 2 and Bcl X  [ **BCL2L1** ]  L genes nor in the expression of the tumor suppressor gene p53.  Ref: 12885947 J Leukoc Biol, 2003
- ... other Bcl 2 family proteins including  **Bax** , Bcl 2, Bcl XL  [ **BCL2L1** ] , and Bag remained unchanged.  Ref: 12874837 J Cell Biochem, 2003
