# Supplementary material for: Content-rich biological network constructed by mining PubMed abstracts
Source: BMC Bioinformatics. 2004 Oct 8;5:147. doi: 10.1186/1471-2105-5-147 (PMC528731; doi:10.1186/1471-2105-5-147)
Supplement: Additional File 2 — The original results of the above study (non-essential files are deleted to keep the file size under the limit set by BMC bioinformatics). [file 1471-2105-5-147-S2.bz2 › chilibotAdditionalFile2/dip05/2ID9194558E2/html/BCL2_RAF1.html]

 


 **BCL2** and **RAF1** 
  
Found 126 abstracts in PubMed, retrieved 05.  
 

 What does Google say? 
 PDF only 
| .edu only 

---

**Interactive relationship** (e.g. stimulation, inhibition, etc)

**Inhibitory relationship**- FDCP Mix BCL 2  [ **BCL2** ]  cells grown in IL 3 were distinguished from FDCP Mix and FDCP Mix BCL X L cells by a striking reduction in cellular levels of Raf 1  [ **RAF1** ]  protein.  Ref: 12721288 J Biol Chem< MedlineTA>J Biol Chem,
**Neutral relationship**- Replacement of the BCL 2  [ **BCL2** ]  BH4 domain with the related BCL X L BH4 sequence resulted in a switch of FDCP Mix BCL 2  [ **BCL2** ]  to erythroid fate accompanied by persistence of Raf 1  [ **RAF1** ]  protein expression.  Ref: 12721288 J Biol Chem< MedlineTA>J Biol Chem,
- These effects are associated with differential regulation of Raf 1  [ **RAF1** ]  expression, perhaps involving the previously identified interaction between BCL 2  [ **BCL2** ]  BH4 and the catalytic domain of Raf 1  [ **RAF1** ] .  Ref: 12721288 J Biol Chem< MedlineTA>J Biol Chem,

**Non-interactive relationship** (e.g. studied together, co-existance, homology, etc.)

- Using U937 leukemic cells, we evaluated the effect of docetaxel on phosphatidylcholine PC and its metabolites, phosphatidic acid PA and diacylglycerol DAG, and their impact on MAPK and NF kappa B activation, as well as on Raf 1  [ **RAF1** ]  and Bcl 2  [ **BCL2** ]  phosphorylation.  Ref: 12724857 J Exp Ther Oncol< MedlineTA>J Exp Ther Oncol,
- One antisense compound has been approved for local treatment of cytomegalovirus induced retinitis, and several others are in clinical trials, including those targeting the mRNA of Bcl 2  [ **BCL2** ] , protein kinase C alpha PKC alpha, c raf  [ **RAF1** ]  or Ha ras.  Ref: 12867066 Lung Cancer, 2003
- Moreover, enforced expression of Raf 1  [ **RAF1** ]  redirected FDCP Mix BCL 2  [ **BCL2** ]  cells to an erythroid fate, and prohibited generation of myeloid cells.  Ref: 12721288 J Biol Chem< MedlineTA>J Biol Chem, 2003
