# Supplementary material for: Content-rich biological network constructed by mining PubMed abstracts
Source: BMC Bioinformatics. 2004 Oct 8;5:147. doi: 10.1186/1471-2105-5-147 (PMC528731; doi:10.1186/1471-2105-5-147)
Supplement: Additional File 2 — The original results of the above study (non-essential files are deleted to keep the file size under the limit set by BMC bioinformatics). [file 1471-2105-5-147-S2.bz2 › chilibotAdditionalFile2/dip05/3ID9194558E4/html/TP53_BCL2.html]

 


 **TP53** and **BCL2** 
  
Found 77 abstracts in PubMed, retrieved 05.  
 

 What does Google say? 
 PDF only 
| .edu only 

---

**Interactive relationship** (e.g. stimulation, inhibition, etc)

**Non-interactive relationship** (e.g. studied together, co-existance, homology, etc.)

- PML bodies were found to localize specifically with the  **TP53**  locus in about 50% of Jurkat interphase nuclei, but never in proximity with the  **BCL2**  locus.  Ref: 12837275 Genomics, 2003
- To investigate the role of PML bodies in regulation of cell transformation and apoptosis related gene transcription, we employed the immuno FISH method to examine the relationship between PML bodies and the  **TP53**  and  **BCL2**  gene loci.  Ref: 12837275 Genomics, 2003
- Evaluation of clinical significance of  **TP53** , BCL 2  [ **BCL2** ] , BAX and MEK1 expression in 229 ovarian carcinomas treated with platinum based regimen.  Ref: 12644821 Br J Cancer, 2003
- Another novel finding is that CR was negatively influenced by high BAX expression in all patients group P=0.047 and by  **BCL2**  expression in the  **TP53**  group P=0.05.  Ref: 12644821 Br J Cancer, 2003
- We combined these approaches in a series of eight PCNSL cases, analyzing the chromosomal abnormalities using comparative genomic hybridization CGH, testing for Epstein Barr virus EBV involvement by in situ hybridization for EBER, assessing expression of p53, Bcl 2  [ **BCL2** ] , Bcl 6 and CD10 by means of immunohistochemistry, and screening for mutations of the  **TP53**  gene by DGGE.  Ref: 12802586 Virchows Arch< MedlineTA>Virchows Arch, 2003
