# Supplementary material for: Content-rich biological network constructed by mining PubMed abstracts
Source: BMC Bioinformatics. 2004 Oct 8;5:147. doi: 10.1186/1471-2105-5-147 (PMC528731; doi:10.1186/1471-2105-5-147)
Supplement: Additional File 2 — The original results of the above study (non-essential files are deleted to keep the file size under the limit set by BMC bioinformatics). [file 1471-2105-5-147-S2.bz2 › chilibotAdditionalFile2/dip05/6ID9121766E15/html/RBL2_MYOD1.html]

 


 **RBL2** and **MYOD1** 
  
Found 2 abstracts in PubMed, retrieved 2.  
 

 What does Google say? 
 PDF only 
| .edu only 

---

**Interactive relationship** (e.g. stimulation, inhibition, etc)

**Non-interactive relationship** (e.g. studied together, co-existance, homology, etc.)

- Both pRb2  [ **RBL2** ]  p130 and pRb, as well as MyoD  [ **MYOD1** ] , are up regulated in myotubes.  Ref: 9537223 Cancer Res, 1998
- Withdrawal from the cell cycle of differentiating myocytes is regulated by the myogenic basic helix loop helix bHLH protein MyoD  [ **MYOD1** ]  and the pocket proteins pRb, p107 and pRb2  [ **RBL2** ]  p130.  Ref: 9121766 Oncogene, 1997
