# Supplementary material for: Content-rich biological network constructed by mining PubMed abstracts
Source: BMC Bioinformatics. 2004 Oct 8;5:147. doi: 10.1186/1471-2105-5-147 (PMC528731; doi:10.1186/1471-2105-5-147)
Supplement: Additional File 2 — The original results of the above study (non-essential files are deleted to keep the file size under the limit set by BMC bioinformatics). [file 1471-2105-5-147-S2.bz2 › chilibotAdditionalFile2/dip05/8ID9067571E22/html/CDKN1B_CDC28.html]

 


 **CDKN1B** and **CDC28** 
  
Found 3 abstracts in PubMed, retrieved 3.  
 

 What does Google say? 
 PDF only 
| .edu only 

---

**Interactive relationship** (e.g. stimulation, inhibition, etc)

**Inhibitory relationship**- ... overexpression of  **cdc28**  protein kinase 1 CKS1 blocks the inhibition of the cyclin D1 CDK4 complex by the CDK inhibitor p27 Kip1  [ **CDKN1B** ] .  Ref: 12605034 Diagn Mol Pathol, 2003
**Neutral relationship**
