# Supplementary material for: Content-rich biological network constructed by mining PubMed abstracts
Source: BMC Bioinformatics. 2004 Oct 8;5:147. doi: 10.1186/1471-2105-5-147 (PMC528731; doi:10.1186/1471-2105-5-147)
Supplement: Additional File 2 — The original results of the above study (non-essential files are deleted to keep the file size under the limit set by BMC bioinformatics). [file 1471-2105-5-147-S2.bz2 › chilibotAdditionalFile2/dip05/9ID9009191E30/html/ACTN2_GRIN1.html]

 


 **ACTN2** and **GRIN1** 
  
Found 10 abstracts in PubMed, retrieved 05.  
 

 What does Google say? 
 PDF only 
| .edu only 

---

**Interactive relationship** (e.g. stimulation, inhibition, etc)

**Neutral relationship**- Alpha actinin alpha actinin 2  [ **ACTN2** ]  is a protein which links the NR1  [ **GRIN1** ]  and NR2B subunits of N methyl D aspartate NMDA glutamate receptors to the actin cytoskeleton.  Ref: 10925145 Brain Res Mol Brain Res, 2000
- Conversely, alpha actinin 2  [ **ACTN2** ]  antibody immunoprecipitated only modest fractions of striatal NR1  [ **GRIN1** ] , NR2A and NR2B subunits.  Ref: 10925145 Brain Res Mol Brain Res, 2000
- Additionally, the limited expression of alpha actinin 2  [ **ACTN2** ]  could have important functional implications in N methyl D aspartate receptor  [ **GRIN1** ]  localization and modulation.  Ref: 11246149 Neuroscience, 2001
- A subunit specific anti NR1  [ **GRIN1** ]  antibody co precipitated major fractions of NR2A and NR2B subunits, but only a minor fraction of striatal alpha actinin 2  [ **ACTN2** ] .  Ref: 10925145 Brain Res Mol Brain Res, 2000
- A similar reduction in the effects of ethanol on wild type NR1  [ **GRIN1** ]  2A but not NR1  [ **GRIN1** ]  2B or NR1  [ **GRIN1** ]  2C receptors was observed after co expression of full length or truncated human skeletal muscle alpha actinin 2  [ **ACTN2** ]  proteins that produce a functional knockout of the C0 domain.  Ref: 10809744 J Biol Chem, 2000

**Non-interactive relationship** (e.g. studied together, co-existance, homology, etc.)

- Differential immunoreactivity for alpha actinin 2  [ **ACTN2** ] , an N methyl D aspartate receptor  [ **GRIN1** ]  actin binding protein, in hippocampal interneurons.  Ref: 11246149 Neuroscience, 2001
- Reduced ethanol inhibition of N methyl D aspartate receptors by deletion of the NR1  [ **GRIN1** ]  C0 domain or overexpression of alpha actinin 2  [ **ACTN2** ]  proteins.  Ref: 10809744 J Biol Chem, 2000
- This study examined the immunoreactivity of alpha actinin 2  [ **ACTN2** ] , an actin binding N methyl D aspartate receptor  [ **GRIN1** ]  linking protein, in the rat hippocampal formation using double labelling immunofluorescence.  Ref: 11246149 Neuroscience, 2001
- Like Ca calmodulin, autophosphorylated CaMKII competes with alpha actinin 2  [ **ACTN2** ]  for binding to NR1  [ **GRIN1** ] .  Ref: 12379661 J Biol Chem, 2002
