# Supplementary material for: Content-rich biological network constructed by mining PubMed abstracts
Source: BMC Bioinformatics. 2004 Oct 8;5:147. doi: 10.1186/1471-2105-5-147 (PMC528731; doi:10.1186/1471-2105-5-147)
Supplement: Additional File 2 — The original results of the above study (non-essential files are deleted to keep the file size under the limit set by BMC bioinformatics). [file 1471-2105-5-147-S2.bz2 › chilibotAdditionalFile2/dip05/13ID8676499E58/html/GROL_PRNP.html]

 


 **GROL** and **PRNP** 
  
Found 9 abstracts in PubMed, retrieved 05.  
 

 What does Google say? 
 PDF only 
| .edu only 

---

**Interactive relationship** (e.g. stimulation, inhibition, etc)

**Neutral relationship**- These results show that chaperonins of the Hsp60  [ **GROL** ]  class can, in principle, mediate PrP  [ **PRNP** ]  aggregation de novo, i.e. independently of a pre existent PrP  [ **PRNP** ]  Sc template.  Ref: 11697909 J Mol Biol, 2001
- Furthermore, the levels of constitutive expression of HSP105, HSC70, HSP60  [ **GROL** ] , and HSP25 were similar between the brain tissues isolated from the PrP  [ **PRNP** ]  and PrP  [ **PRNP** ]  mice.  Ref: 9582258 Exp Neurol, 1998
- Here we report that the bacterial chaperonin GroEL  [ **GROL** ] , a close homolog of eukaryotic Hsp60  [ **GROL** ] , can catalyze the aggregation of chemically denatured and of folded, recombinant PrP  [ **PRNP** ]  in a model reaction in vitro.  Ref: 11697909 J Mol Biol, 2001
- We report here that Hsp60  [ **GROL** ] , a member of the GroEL  [ **GROL** ]  family of chaperonins, of B. abortus is capable of interacting directly or indirectly with cellular prion protein  [ **PRNP** ]  PrPC on host cells.  Ref: 12847134 J Exp Med, 2003

**Non-interactive relationship** (e.g. studied together, co-existance, homology, etc.)

- To study a role of PrPC in the regulation of expression of heat shock proteins HSPs, a group of molecular chaperones, heat induced expression of major HSPs HSP105, HSP90alpha, HSP72, HSC70, HSP60  [ **GROL** ] , and HSP25 was investigated in cultured skin fibroblasts isolated from the mice homogeneous for a disrupted PrP  [ **PRNP** ]  gene PrP  [ **PRNP** ]  mice by Western blot analysis and immunocytochemistry.  Ref: 9582258 Exp Neurol, 1998
