# Supplementary material for: Content-rich biological network constructed by mining PubMed abstracts
Source: BMC Bioinformatics. 2004 Oct 8;5:147. doi: 10.1186/1471-2105-5-147 (PMC528731; doi:10.1186/1471-2105-5-147)
Supplement: Additional File 2 — The original results of the above study (non-essential files are deleted to keep the file size under the limit set by BMC bioinformatics). [file 1471-2105-5-147-S2.bz2 › chilibotAdditionalFile2/dip05/14ID9153399E59/html/ITGB2_ICAM2.html]

 


 **ITGB2** and **ICAM2** 
  
Found 158 abstracts in PubMed, retrieved 05.  
 

 What does Google say? 
 PDF only 
| .edu only 

---

**Interactive relationship** (e.g. stimulation, inhibition, etc)

**Neutral relationship**- The beta 2 integrin LFA 1  [ **ITGB2** ]  CD11a CD18  [ **ITGB2** ]  is a leukocyte specific adhesion molecule that mediates leukocyte extravasation, antigen presentation, and T cell mediated cytolysis through its interaction with its counter receptors, ICAM 1, ICAM 2  [ **ICAM2** ] , and ICAM 3.  Ref: 12799043 J Immunol Methods, 2003

**Non-interactive relationship** (e.g. studied together, co-existance, homology, etc.)

- RESULTS Specific cation dependent binding of lymphocytes to bronchial endothelium was observed which was significantly inhibited by antibodies against P selectin, PSGL 1, L selectin, LFA 1  [ **ITGB2** ] , ICAM 1 and ICAM 2  [ **ICAM2** ]  but not E selectin, VLA 4, VCAM 1 or Mac 1.  Ref: 12454301 Thorax, 2002
