# Supplementary material for: Content-rich biological network constructed by mining PubMed abstracts
Source: BMC Bioinformatics. 2004 Oct 8;5:147. doi: 10.1186/1471-2105-5-147 (PMC528731; doi:10.1186/1471-2105-5-147)
Supplement: Additional File 2 — The original results of the above study (non-essential files are deleted to keep the file size under the limit set by BMC bioinformatics). [file 1471-2105-5-147-S2.bz2 › chilibotAdditionalFile2/dip05/15ID11821039E61/html/CARB_CARA.html]

 


 **CARB** and **CARA** 
  
Found 27 abstracts in PubMed, retrieved 05.  
 

 What does Google say? 
 PDF only 
| .edu only 

---

**Interactive relationship** (e.g. stimulation, inhibition, etc)

**Neutral relationship**- Sequencing has shown that CPS A  [ **CARA** ]  is encoded by  **carA**  GLN and  **carB**  SYN.  Ref: 10852872 J Bacteriol, 2000
- **CarA**  represses transcription from the  **carB**  promoter P B in the dark, and CarS counteracts this on illumination.  Ref: 11748235 J Biol Chem, 2002
- Among Bacteria the  **carA**  and  **carB**  genes encoding the small  **CarA**  and large  **CarB**  subunits of carbamoylphosphate synthase CPS have been lost in certain symbionts Haemophylus influenzae and in most obligate intracellular parasites Chlamydiae, Spirochaetes, Mycoplasmatales, Rickettsiae having genome sizes in the 0.7 to 1.1 Mb range.  Ref: 12107592 J Mol Evol, 2002
- **CarA**  hereafter named carbapenam synthetase has been proposed to catalyze formation of 3S, 5S carbapenam 3 carboxylic acid from 2S, 5S 5 carboxymethyl proline based on characterization of the products of fermentation experiments in Escherichia coli cells transformed with pET24a  **carB**  and pET24a carAB, and on sequence homology to beta lactam synthetase, an enzyme that catalyzes formation of a monocyclic beta lactam ring with concomitant ATP hydrolysis.  Ref: 12820893 Biochemistry, 2003

**Non-interactive relationship** (e.g. studied together, co-existance, homology, etc.)

- The Erwinia carotorova  **carA** ,  **carB** , and carC gene products are essential for the biosynthesis of 5R carbapen 2 em 3 carboxylic acid, the simplest carbapenem beta lactam antibiotic.  Ref: 12820893 Biochemistry, 2003
- Group 3 taxa the crenarchaea Pyrobaculum aerophilum, Sulfolobus solfataricus, and Sulfolobus tokodaii and the euryarchaeon Pyrococcus furiosus harbor CPS genes whose encoded proteins appear to be archaeal consistent with an archaeal origin, the  **CarA**  and  **CarB**  sequences in this group possess both unique signatures and signatures affiliating them to Eukarya.  Ref: 12107592 J Mol Evol, 2002
- that is, they are intermixed with bacterial homologues on a phylogeny of concatenated  **CarA**  and  **CarB**  sequences and are not distinguishable from bacterial sequences after searching for domain specific amino acid residue positions.  Ref: 12107592 J Mol Evol, 2002
- Group 2 taxa comprising Halobacteriales, Thermoplasmales, Methanococcales, Methanomicrobiales, Archaeoglobales harbor CPS genes whose encoded  **CarB**  and  **CarA**  subunit proteins are ostensibly bacterial in origin.  Ref: 12107592 J Mol Evol, 2002
