# Supplementary material for: Content-rich biological network constructed by mining PubMed abstracts
Source: BMC Bioinformatics. 2004 Oct 8;5:147. doi: 10.1186/1471-2105-5-147 (PMC528731; doi:10.1186/1471-2105-5-147)
Supplement: Additional File 2 — The original results of the above study (non-essential files are deleted to keep the file size under the limit set by BMC bioinformatics). [file 1471-2105-5-147-S2.bz2 › chilibotAdditionalFile2/dip05/16ID8411172E62/html/MALE_MALG.html]

 


 **MALE** and **MALG** 
  
Found 26 abstracts in PubMed, retrieved 05.  
 

 What does Google say? 
 PDF only 
| .edu only 

---

**Interactive relationship** (e.g. stimulation, inhibition, etc)

**Neutral relationship**- Five additional open reading frames, encoding components of a maltose transport system MalF and  **MalG** , a putative transcriptional regulator MalR, a cyclodextrinase CdaA, and an alpha glucosidase GlcA, were identified downstream of  **malE** .  Ref: 11053372 J Bacteriol, 2000

**Non-interactive relationship** (e.g. studied together, co-existance, homology, etc.)

- We report the characterization of TrmB, a protein of 3 00 apparent molecular weight, that is involved in the maltose specific regulation of a gene cluster in Thermococcus litoralis,  **malE**  malF  **malG**  orf trmB malK, encoding a binding protein dependent ABC transporter for trehalose and maltose.  Ref: 12426307 J Biol Chem, 2003
