# Supplementary material for: Content-rich biological network constructed by mining PubMed abstracts
Source: BMC Bioinformatics. 2004 Oct 8;5:147. doi: 10.1186/1471-2105-5-147 (PMC528731; doi:10.1186/1471-2105-5-147)
Supplement: Additional File 2 — The original results of the above study (non-essential files are deleted to keep the file size under the limit set by BMC bioinformatics). [file 1471-2105-5-147-S2.bz2 › chilibotAdditionalFile2/dip05/19ID7594592E76/html/HMGA1_SPI1.html]

 


 **HMGA1** and **SPI1** 
  
Found 1 abstracts in PubMed, retrieved 1.  
 

 What does Google say? 
 PDF only 
| .edu only 

---

**Interactive relationship** (e.g. stimulation, inhibition, etc)

**Stimulatory relationship**- EMSA analysis demonstrated that DN  **HMGA1**  disrupts established PU.1  [ **SPI1** ]  mu enhancer binding.  Ref: 11906180 Biochem Biophys Res Commun, 2002
**Neutral relationship**- **HMGA1**  functions through direct interaction with PU.1  [ **SPI1** ] , one of the ets proteins critical for enhancer activation.  Ref: 11906180 Biochem Biophys Res Commun, 2002

**Non-interactive relationship** (e.g. studied together, co-existance, homology, etc.)

- In sharp contrast, DN  **HMGA1**  had no effect on binding activity of the ETS DNA binding domains of either PU.1  [ **SPI1** ]  or Ets 1, or the bHLH zip protein TFE3, suggesting specificity.  Ref: 11906180 Biochem Biophys Res Commun, 2002
