# Supplementary material for: Content-rich biological network constructed by mining PubMed abstracts
Source: BMC Bioinformatics. 2004 Oct 8;5:147. doi: 10.1186/1471-2105-5-147 (PMC528731; doi:10.1186/1471-2105-5-147)
Supplement: Additional File 2 — The original results of the above study (non-essential files are deleted to keep the file size under the limit set by BMC bioinformatics). [file 1471-2105-5-147-S2.bz2 › chilibotAdditionalFile2/dip05/20ID7594592E77/html/SSRP1_SPI1.html]

 


 **SSRP1** and **SPI1** 
  
Found 1 abstracts in PubMed, retrieved 1.  
 

 What does Google say? 
 PDF only 
| .edu only 

---

- J Immunol, 1995   **Multiple proteins physically interact with PU.1 **[SPI1]**.
  Transcriptional synergy with NF IL6 beta C EBP delta, CRP3 .**.
  PU.1 **[SPI1]** is a transcription factor that belongs to the ets family of DNA binding proteins.
  In this study, we show by Far Western blot analyses that multiple nuclear proteins are capable of physically interacting with PU.1 **[SPI1]**.
  Using radiolabeled PU.1 **[SPI1]** protein as a probe, we screened a B cell cDNA expression library and isolated a number of clones encoding PU.1 **[SPI1]** interacting proteins.
  Three of these clones encode DNA binding proteins NF IL6 beta, HMG I Y, and SSRP **[SSRP1]** , one clone encodes a chaperone protein, and another clone encodes a multifunctional phosphatase.
  We have characterized the physical and functional interactions between PU.1 **[SPI1]** and NF IL6 beta, a leucine zipper transcription factor implicated in inflammatory responses.
  We found that deletion of the carboxyl terminal 28 amino acids of PU.1 **[SPI1]** disrupted PU.1 **[SPI1]** NF IL6 beta physical interaction.
  This deletion disrupts the PU.1 **[SPI1]** Ets domain.
  Deletion of the NF IL6 beta leucine zipper domain also greatly diminished the interaction between these two proteins.
  In transient expression assays, we found that PU.1 **[SPI1]** and NF IL6 beta can functionally cooperate to synergistically activate transcription.
  Electrophoretic mobility shift assays showed that PU.1 **[SPI1]** and NF IL6 beta can simultaneously bind to adjacent DNA binding sites, but apparently do not influence the kinetics or affinity of each other s DNA binding.
  These results suggest that transcriptional synergy is due to each protein independently influencing the basal transcription complex.
