# Supplementary material for: Content-rich biological network constructed by mining PubMed abstracts
Source: BMC Bioinformatics. 2004 Oct 8;5:147. doi: 10.1186/1471-2105-5-147 (PMC528731; doi:10.1186/1471-2105-5-147)
Supplement: Additional File 2 — The original results of the above study (non-essential files are deleted to keep the file size under the limit set by BMC bioinformatics). [file 1471-2105-5-147-S2.bz2 › chilibotAdditionalFile2/dip05/21ID9129204E79/html/TNFRSF1A_TRAF2.html]

 


 **TNFRSF1A** and **TRAF2** 
  
Found 140 abstracts in PubMed, retrieved 05.  
 

 What does Google say? 
 PDF only 
| .edu only 

---

**Interactive relationship** (e.g. stimulation, inhibition, etc)

**Neutral relationship**- In an in vitro binding assay, the intracellular domain of TNF R1  [ **TNFRSF1A** ]  bound TRADD, RIP and  **TRAF2**  but did not bind FADD or caspase 8.  Ref: 12721308 J Biol Chem< MedlineTA>J Biol Chem,

**Non-interactive relationship** (e.g. studied together, co-existance, homology, etc.)

- The initial plasma membrane bound complex complex I consists of TNFR1  [ **TNFRSF1A** ] , the adaptor TRADD, the kinase RIP1, and  **TRAF2**  and rapidly signals activation of NF kappaB.  Ref: 12887920 Cell, 2003
- Here we show that upon TNFalpha binding, TNFR1  [ **TNFRSF1A** ]  translocates to cholesterol and sphingolipid enriched membrane microdomains, termed lipid rafts, where it associates with the Ser Thr kinase RIP and the adaptor proteins TRADD and  **TRAF2** , forming a signaling complex.  Ref: 12753742 Immunity, 2003
