# Supplementary material for: Content-rich biological network constructed by mining PubMed abstracts
Source: BMC Bioinformatics. 2004 Oct 8;5:147. doi: 10.1186/1471-2105-5-147 (PMC528731; doi:10.1186/1471-2105-5-147)
Supplement: Additional File 2 — The original results of the above study (non-essential files are deleted to keep the file size under the limit set by BMC bioinformatics). [file 1471-2105-5-147-S2.bz2 › chilibotAdditionalFile2/dip05/22ID9129204E80/html/FADD_TNFRSF1A.html]

 


 **FADD** and **TNFRSF1A** 
  
Found 377 abstracts in PubMed, retrieved 05.  
 

 What does Google say? 
 PDF only 
| .edu only 

---

**Interactive relationship** (e.g. stimulation, inhibition, etc)

**Inhibitory relationship**- Neutralizing anti TNF R1  [ **TNFRSF1A** ]  antibodies inhibited macrophage activation assessed by Fas  [ **FADD** ]  L expression and NO secretion.  Ref: 12869351 Arterioscler Thromb Vasc BiolArterioscler Thromb Vasc Biol,
**Neutral relationship**- Apoptosis induced by TNF receptor I TNFR1  [ **TNFRSF1A** ]  is thought to proceed via recruitment of the adaptor  **FADD**  and caspase 8 to the receptor complex.  Ref: 12887920 Cell, 2003

**Non-interactive relationship** (e.g. studied together, co-existance, homology, etc.)

- We tested the hypothesis that activation of specific apoptotic pathways represents a mechanism for tubular cell death after CAD kidney transplantation.METHODS Serial tissue sections from paraffin embedded needle biopsy specimens obtained at approximately 1 hr of reperfusion after transplantation of 13 CAD and 12 living related donor LRD renal allografts were examined by using the terminal deoxynucleotide transferase mediated dUTP nick end labeling assay to detect apoptosis and by immunohistochemistry for expression of key pro apoptotic molecules Bax, Bak, tumor necrosis factor receptor TNFR 1  [ **TNFRSF1A** ] , Fas  [ **FADD** ] , and cytochrome c.  Ref: 12865785 Transplantation, 2003
- The chicken death receptors, Fas  [ **FADD** ] , TNFR1  [ **TNFRSF1A** ] , DR6, and TVB, are constitutively expressed in a relatively wide variety of hen tissues.  Ref: 12878204 Biochem Biophys Res Commun, 2003
