# Supplementary material for: Content-rich biological network constructed by mining PubMed abstracts
Source: BMC Bioinformatics. 2004 Oct 8;5:147. doi: 10.1186/1471-2105-5-147 (PMC528731; doi:10.1186/1471-2105-5-147)
Supplement: Additional File 2 — The original results of the above study (non-essential files are deleted to keep the file size under the limit set by BMC bioinformatics). [file 1471-2105-5-147-S2.bz2 › chilibotAdditionalFile2/dip05/23ID9020361E81/html/NFKB1_IL1R1.html]

 


 **NFKB1** and **IL1R1** 
  
Found 5 abstracts in PubMed, retrieved 05.  
 

 What does Google say? 
 PDF only 
| .edu only 

---

**Interactive relationship** (e.g. stimulation, inhibition, etc)

**Non-interactive relationship** (e.g. studied together, co-existance, homology, etc.)

- The present study uncovered a mechanism regulating fibronectin production in vascular myocytes related to a functional interplay between EBP  [ **NFKB1** ]  and the interleukin 1 receptor  [ **IL1R1** ]  type I.  Ref: 8635505 ,
