# Supplementary material for: Content-rich biological network constructed by mining PubMed abstracts
Source: BMC Bioinformatics. 2004 Oct 8;5:147. doi: 10.1186/1471-2105-5-147 (PMC528731; doi:10.1186/1471-2105-5-147)
Supplement: Additional File 2 — The original results of the above study (non-essential files are deleted to keep the file size under the limit set by BMC bioinformatics). [file 1471-2105-5-147-S2.bz2 › chilibotAdditionalFile2/dip05/24ID9219684E84/html/RASA1_HRAS.html]

 


 **RASA1** and **HRAS** 
  
Found 13 abstracts in PubMed, retrieved 05.  
 

 What does Google say? 
 PDF only 
| .edu only 

---

**Interactive relationship** (e.g. stimulation, inhibition, etc)

**Non-interactive relationship** (e.g. studied together, co-existance, homology, etc.)

- We have sought activating substitutions in c H ras  [ **HRAS** ]  in the region encoding the effector domain, on the rationale that such mutations would dissociate effector function from negative regulation by GAP  [ **RASA1** ] .  Ref: 8246952 Mol Cell Biol, 1993
