# Supplementary material for: Content-rich biological network constructed by mining PubMed abstracts
Source: BMC Bioinformatics. 2004 Oct 8;5:147. doi: 10.1186/1471-2105-5-147 (PMC528731; doi:10.1186/1471-2105-5-147)
Supplement: Additional File 2 — The original results of the above study (non-essential files are deleted to keep the file size under the limit set by BMC bioinformatics). [file 1471-2105-5-147-S2.bz2 › chilibotAdditionalFile2/dip05/25ID8999548E88/html/CASP1_IL1B.html]

 


 **CASP1** and **IL1B** 
  
Found 435 abstracts in PubMed, retrieved 05.  
 

 What does Google say? 
 PDF only 
| .edu only 

---

**Interactive relationship** (e.g. stimulation, inhibition, etc)

**Non-interactive relationship** (e.g. studied together, co-existance, homology, etc.)

- As cytoplasmatic adaptor molecules of FAS, e.g. FLIP Fas associated death domain protein FADD like interleukin 1 beta  [ **IL1B** ]  converting enzyme  [ **CASP1** ]  FLICE caspase 8 inhibitory protein, also modulate TRAIL signals, we determined whether chelerythrin affected TRAIL mediated apoptosis.  Ref: 12877678 Br J HaematolBr J Haematol,
- Changes in caspase 3 and caspase 1  [ **CASP1** ] , interleukin 1 beta  [ **IL1B** ]  IL 1 beta  [ **IL1B** ] , and collagen I, III, and IV proteins and mRNA were detected by Western blotting and Northern blotting, respectively.  Ref: 12753292 Kidney Int, 2003
- Vertex is collaborating with Aventis Pharma AG formerly Hoechst Marion Roussel Inc in the development of pralnacasan, an interleukin IL 1b  [ **IL1B** ]  converting enzyme ICE  [ **CASP1** ]  inhibitor, for the potential treatment of inflammatory diseases 17024 8829 53094.  Ref: 12789619 Idrugs, 2003
