# Supplementary material for: Content-rich biological network constructed by mining PubMed abstracts
Source: BMC Bioinformatics. 2004 Oct 8;5:147. doi: 10.1186/1471-2105-5-147 (PMC528731; doi:10.1186/1471-2105-5-147)
Supplement: Additional File 2 — The original results of the above study (non-essential files are deleted to keep the file size under the limit set by BMC bioinformatics). [file 1471-2105-5-147-S2.bz2 › chilibotAdditionalFile2/dip05/29ID8824290E95/html/CCKBR_GAS.html]

 


 **CCKBR** and **GAS** 
  
Found 4 abstracts in PubMed, retrieved 4.  
 

 What does Google say? 
 PDF only 
| .edu only 

---

- Neuropsychopharmacology, 2002   The cholecystokinin system, which has been shown to interact with both the panicogenic and respiratory systems, provides an interesting mechanism to further evaluate the central chemoreceptor and its effect on panic attack sensitivity.
  Intravenous CCK, a naturally occurring neuropeptide in the brain, has been found to induce the emotional and somatic symptoms of panic in both Panic Disorder and Normal Control subjects in a dose dependent and reproducible fashion.
  To induce these effects, lower doses of intravenous CCK are required in the PD patients, relative to the NC subjects potentially suggesting that endogenous alterations in the CCK system may be contributing to the development of PD.
  Intravenous administration of CCK 4 in association with panic also results in subjective dyspnea, that is, diminution in vital capacity without an effect on the respiratory resistance.
  CCK 4 also causes a significant increase in tidal volume and minute ventilation but has no effect on breathing frequency.
  These observations suggest that a CCK B receptor **[CCKBR]** agonist may be acting as a respiratory stimulant, exerting its effect on anxiety through a direct effect on respiration.
  This study represents an examination of the specific effects of CCK 4 on the central chemoreceptor response.
  The study used a modified rebreathing technique, which accurately measures the ventilatory response to carbon dioxide in terms of both threshold and sensitivity.
  This technique requires the subject to rebreathe from a bag containing a hyperoxic and hypercapnic **gas** mixture resulting in rapid equilibration between alveolar **gas** and arterial blood.
  Use of a hyperoxic **gas** allows for the preferential examination of the central chemoreflexes with little if any effect of the peripheral chemoreflexes.
  After significant training, 15 healthy control subjects were assigned via a double blind procedure to receive an intravenous injection of placebo or CCK 4, using a between subjects design.
  A between subjects comparison was undertaken for the injection run between subjects receiving the CCK 4 injection and those receiving the placebo injection.
  As well, a within subject comparison was undertaken to compare the results of the run following the injection vs. the previous run when no injection took place .
  No significant differences were noted between subjects who received CCK 4 as compared with placebo for basal or sub threshold ventilation, threshold CO resulting in a change in ventilation, or sensitivity of the central chemoreflex, regardless of whether a panic attack did or did not take place.
  In addition, within the group receiving the CCK 4 challenge, no significant differences were noted during run #3 and a prior run where no injection took place .
  We conclude that CCK 4 does not act to induce panic by altering the central sensitive chemoreceptor.

  - Regul Pept, 1995   **Mobilization of gastric histamine during repeated administration of a proton potassium adenosine triphosphatase inhibitor in intact and antrectomized rats.**.
    Intact and antrectomized female rats were treated with the potent proton pump inhibitor, E3810 daily 40 mg kg weight, s.c. for 4 weeks.
    Plasma gastrin concentration and urinary excretion of N terminal big gastrin **[GAS]** increased until day 14 and persisted at a high level in intact rats treated with E3810, but did not increase in antrectomized rats.
    Urinary excretion of histamine increased progressively and reached 7 times the control value following 4 weeks of treatment with E3810 in intact rats, but not in antrectomized rats.
    At the termination of the treatment, the endocrine cell density in the oxyntic mucosa of intact rats had increased by 85% with increased histamine content and elevated histidine decarboxylase activity, while antrectomized rats showed a low histamine level and low histidine decarboxylase activity.
    Administration of gastrin 17 I 10 micrograms kg weight, sc itself caused a significant increase in urinary excretion of histamine, which was inhibited by the specific gastrin receptor **[CCKBR]** antagonist, L 36 60.
    These results suggests that the massive urinary excretion of histamine caused by the treatment with E3810 reflects gastrin induced mobilization of gastric histamine.
    neither E3810 itself nor E3810 induced luminal pH elevation has direct effects on mobilization of oxyntic mucosal histamine.

    - Int J Pept Protein Res, 1991   **Fully synthetic immunogens.
      Part III.
      Synthesis of hinge peptide gastrin conjugates and their immunological properties.**.
      As core molecule for the multiple attachment of antigenic peptides we have selected the human IgG1 hinge fragment 225 232 225 232 .
      Two types of conjugates of this double chain bis cystinyl hinge peptide were prepared i by linking its C termini to NIe15 human little gastrin 7 and ii by elongating the resulting hinge peptide NIe15 little gastrin 2 17 conjugate at the two N termini with the human big gastrin **[GAS]** sequence 1 14 to produce the big gastrin **[GAS]** 1 14 hinge peptide little gastrin 2 17 conjugate.
      For the synthesis of these peptide structures both the route via the preformed double chain bis cystinyl peptide and the route via suitably protected monomeric bis cysteinyl peptides were used.
      For the latter approach advantage was taken of the previous observation about the preferred oxidation of the bis cysteinyl hinge peptide 225 232 to the dimer in parallel alignment.
      Both synthetic routes led to identical products.
      Immunization experiments in guinea pigs with the synthetic hybrids led to surprisingly strong immune responses with anti little gastrin antibody titers comparable to those induced by the iso 1 cytochrome c little gastrin 2 17 conjugate as carrier hapten system.
      These findings show that the two gastrin constructs are fully competent immunogens.
      Additionally, the gastrin receptor **[CCKBR]** like specificity of the antibodies indicates that both the synthetic hybrids and the cytochrome c conjugate allow for expression of a little gastrin specific conformational epitope similar to the bioactive structure of this hormone.
      The usefulness of such synthetic hybrids is further confirmed by the observation that the bivalent immunogen, containing both the little gastrin 2 17 and the big gastrin **[GAS]** 1 14 sequence, is capable of inducing an immune response against both antigenic sequences, although with different efficiency.
      These results fully confirm our expectations.
