# Supplementary material for: Content-rich biological network constructed by mining PubMed abstracts
Source: BMC Bioinformatics. 2004 Oct 8;5:147. doi: 10.1186/1471-2105-5-147 (PMC528731; doi:10.1186/1471-2105-5-147)
Supplement: Additional File 2 — The original results of the above study (non-essential files are deleted to keep the file size under the limit set by BMC bioinformatics). [file 1471-2105-5-147-S2.bz2 › chilibotAdditionalFile2/dip05/30ID8824290E96/html/GAS_IRS1.html]

 


 **GAS** and **IRS1** 
  
Found 7 abstracts in PubMed, retrieved 05.  
 

 What does Google say? 
 PDF only 
| .edu only 

---

**Interactive relationship** (e.g. stimulation, inhibition, etc)

**Non-interactive relationship** (e.g. studied together, co-existance, homology, etc.)

- We investigated another model with four parameters, a single shunt compliance Cim representing  **gas**  compression in the face mask in parallel with the infant s total respiratory resistance Rrs inertance Irs  [ **IRS1** ] , and compliance Crs.  Ref: 9016470 Pediatr Pulmonol, 1996
