# Supplementary material for: Content-rich biological network constructed by mining PubMed abstracts
Source: BMC Bioinformatics. 2004 Oct 8;5:147. doi: 10.1186/1471-2105-5-147 (PMC528731; doi:10.1186/1471-2105-5-147)
Supplement: Additional File 2 — The original results of the above study (non-essential files are deleted to keep the file size under the limit set by BMC bioinformatics). [file 1471-2105-5-147-S2.bz2 › chilibotAdditionalFile2/dip05/37ID8598927E147/html/TAF40_TAF6.html]

 


 **TAF40** and **TAF6** 
  
Found 4 abstracts in PubMed, retrieved 4.  
 

 What does Google say? 
 PDF only 
| .edu only 

---

**Interactive relationship** (e.g. stimulation, inhibition, etc)

**Neutral relationship**- Here, a direct interaction between the activation domain of p53 and two subunits of the TFIID complex, TAFII40  [ **TAF40** ]  and TAFII60  [ **TAF6** ] , is reported.  Ref: 7809597 Science, 1995
- This region binds basal transcriptional components such as the TATA box binding protein associated factors TAFII40  [ **TAF40** ]  and TAFII60  [ **TAF6** ]  as well as the mdm 2 and adenovirus type 5 E1B 55 kDa oncoproteins.  Ref: 9427007 Fold Des, 1997
- Finally, we observed that ERM bound TAFII60  [ **TAF6** ]  via AD1 and bound TBP and TAFII40  [ **TAF40** ] , presumably via other activation domains.  Ref: 9358152 Nucleic Acids Res, 1997

**Non-interactive relationship** (e.g. studied together, co-existance, homology, etc.)

- Together, these results suggest that TAFII40  [ **TAF40** ]  and TAFII60  [ **TAF6** ]  are important targets for transmitting activation signals between p53 and the initiation complex.  Ref: 7809597 Science, 1995
- A double point mutation in the activation domain of p53 impaired the ability of this domain to activate transcription and, simultaneously, its ability to interact with both TAFII40  [ **TAF40** ]  and TAFII60  [ **TAF6** ] .  Ref: 7809597 Science, 1995
- p53 transcriptional activation mediated by coactivators TAFII40  [ **TAF40** ]  and TAFII60  [ **TAF6** ] .  Ref: 7809597 Science, 1995
- Overexpression of TAFII40  [ **TAF40** ]  and TAFII60  [ **TAF6** ]  also inhibited activation by p53 GAL4 but had negligible effects on activation by GAL4 VP16 and Sp1, while TAFII110 did not affect any of the activators.  Ref: 8754830 Mol Cell Biol, 1996
- ... TBP or a subcomplex lacking TAFII40  [ **TAF40** ]  and TAFII60  [ **TAF6** ]  did not.  Ref: 7809597 Science, 1995
