# Supplementary material for: Content-rich biological network constructed by mining PubMed abstracts
Source: BMC Bioinformatics. 2004 Oct 8;5:147. doi: 10.1186/1471-2105-5-147 (PMC528731; doi:10.1186/1471-2105-5-147)
Supplement: Additional File 2 — The original results of the above study (non-essential files are deleted to keep the file size under the limit set by BMC bioinformatics). [file 1471-2105-5-147-S2.bz2 › chilibotAdditionalFile2/dip05/40ID8178153E160/html/TBP_TAF2.html]

 


 **TBP** and **TAF2** 
  
Found 10 abstracts in PubMed, retrieved 05.  
 

 What does Google say? 
 PDF only 
| .edu only 

---

**Interactive relationship** (e.g. stimulation, inhibition, etc)

**Neutral relationship**- This interaction might be structurally important for the functional interaction between CIF150  [ **TAF2** ]  and human TFIID  [ **TBP** ] , since CIF150  [ **TAF2** ]  stabilizes TFIID  [ **TBP** ]  binding to a core promoter.  Ref: 9418870 Mol Cell Biol, 1998

**Non-interactive relationship** (e.g. studied together, co-existance, homology, etc.)

- CIF150  [ **TAF2** ]  is capable of mediating TFIID  [ **TBP** ]  dependent Inr activity in a complementation assay, and a protein fraction lacking Inr activity lacks detectable amounts of CIF150  [ **TAF2** ] .  Ref: 9418870 Mol Cell Biol, 1998
- More importantly, we demonstrate that the human TAFII150  [ **TAF2** ]  containing TFIID  [ **TBP** ]  complex is not sufficient, in the context of all purified GTFs and RNA polymerase II, to mediate transcription synergism between TATA and initiator elements and initiator directed transcription from a TAFII dependent TATA less promoter.  Ref: 9774672 Mol Cell Biol, 1998
- Despite the striking similarity to dTAF II 150, CIF150  [ **TAF2** ]  does not appear to be associated with human TFIID  [ **TBP** ] .  Ref: 9418870 Mol Cell Biol, 1998
- We identify the human homologue of Drosophila TAFII150  [ **TAF2** ]  through cognate cDNA cloning and show that it is a tightly associated component of human TFIID  [ **TBP** ] .  Ref: 9774672 Mol Cell Biol, 1998
- Novel cofactors and TFIIA mediate functional core promoter selectivity by the human TAFII150  [ **TAF2** ]  containing TFIID  [ **TBP** ]  complex.  Ref: 9774672 Mol Cell Biol, 1998
- By in situ chromosomal hybridization, and by somatic cell and radiation hybrid analysis, we have determined the genomic position of the human genes encoding four TAFII subunits of TFIID  [ **TBP** ]  TAFII150  [ **TAF2** ] , TAFII105, TAFII68, TAFII18, the three subunits of TFIIA TFIIA35 and TFIIA19, both encoded by the same gene, and TFIIA12, CDK8, and SURB7.  Ref: 11441538 Somat Cell Mol Genet, 1999
