# Supplementary material for: Content-rich biological network constructed by mining PubMed abstracts
Source: BMC Bioinformatics. 2004 Oct 8;5:147. doi: 10.1186/1471-2105-5-147 (PMC528731; doi:10.1186/1471-2105-5-147)
Supplement: Additional File 2 — The original results of the above study (non-essential files are deleted to keep the file size under the limit set by BMC bioinformatics). [file 1471-2105-5-147-S2.bz2 › chilibotAdditionalFile2/dip05/41ID7545910E165/html/TBP_TAF40.html]

 


 **TBP** and **TAF40** 
  
Found 14 abstracts in PubMed, retrieved 05.  
 

 What does Google say? 
 PDF only 
| .edu only 

---

**Interactive relationship** (e.g. stimulation, inhibition, etc)

**Neutral relationship**- These results demonstrate that the TFIIA  **TAF40**  interaction is important in vivo and indicate a functional role for  **TAF40**  as a bridging factor between TFIIA and TFIID  [ **TBP** ] .  Ref: 11238911 Mol Cell Biol, 2001

**Non-interactive relationship** (e.g. studied together, co-existance, homology, etc.)

- No significant correlation between in vivo activation function and in vitro binding to human TATA binding protein  [ **TBP** ] , human TFIIB, or Drosophila melanogaster TAFII40  [ **TAF40** ]  was observed for this set of VP16C mutants.  Ref: 9632787 Mol Cell Biol, 1998
- We show that in addition to making direct contacts with  **TBP** , yeast  **TAF40**  interacts directly and specifically with TFIIA.  Ref: 11238911 Mol Cell Biol, 2001
- Here we show that mutations in the yeast TFIID  [ **TBP** ]  specific protein  **Taf40**  lead to a general cessation of transcription, even in the presence of excess  **TBP** , suggesting that the TFIID  [ **TBP** ]  complex is required at most promoters in vivo.  Ref: 10521393 Genes Dev, 1999
- TFIIA interacts with TFIID  [ **TBP** ]  via association with TATA binding protein  [ **TBP** ]  and  **TAF40** .  Ref: 11238911 Mol Cell Biol, 2001
- TFIID  [ **TBP** ]  specific yeast  **TAF40**  is essential for the majority of RNA polymerase II mediated transcription in vivo.  Ref: 10521393 Genes Dev, 1999
- Finally, we observed that ERM bound TAFII60 via AD1 and bound  **TBP**  and TAFII40  [ **TAF40** ] , presumably via other activation domains.  Ref: 9358152 Nucleic Acids Res, 1997
