# Supplementary material for: Content-rich biological network constructed by mining PubMed abstracts
Source: BMC Bioinformatics. 2004 Oct 8;5:147. doi: 10.1186/1471-2105-5-147 (PMC528731; doi:10.1186/1471-2105-5-147)
Supplement: Additional File 2 — The original results of the above study (non-essential files are deleted to keep the file size under the limit set by BMC bioinformatics). [file 1471-2105-5-147-S2.bz2 › chilibotAdditionalFile2/dip05/42ID7545910E166/html/TBP_TAF6.html]

 


 **TBP** and **TAF6** 
  
Found 70 abstracts in PubMed, retrieved 05.  
 

 What does Google say? 
 PDF only 
| .edu only 

---

**Interactive relationship** (e.g. stimulation, inhibition, etc)

**Neutral relationship**- The MADF domain directs sequence specific DNA binding to a site consisting of multiple trinucleotide repeats, while the BESS domain directs a variety of protein protein interactions, including interactions with itself, with Dorsal, and with a  **TBP**  associated factor  [ **TAF6** ] .  Ref: 12459265 Gene, 2002
- Finally, our results suggest that  **TBP**  associated factor  [ **TAF6** ]  components of SAGA are differentially required for  **TBP**  binding to SAGA dependent promoters.  Ref: 12370284 Mol Cell Biol, 2002
- alternatively TAFII70  [ **TAF6** ]  can be considered a component in the subset of TFIID  [ **TBP** ]  complexes that do not function during oogenesis, but are accumulated in the oocyte for later use during early development.  Ref: 11768213 Genome, 2001

**Non-interactive relationship** (e.g. studied together, co-existance, homology, etc.)

- Of these 400, interferon regulatory factor 4 IRF4, cyclin B2,  **TBP**  associated factor  [ **TAF6** ] , eukaryotic elongation factor and pim 2 were up regulated more than 3.5 fold.  Ref: 11790884 Dis Markers, 2001
- The localization of the TATA binding protein  [ **TBP** ]   **TBP**  associated factor  [ **TAF6** ]  II70 TAFII70  [ **TAF6** ]  in the germinal vesicle GV of newt oocytes was investigated.  Ref: 11768213 Genome, 2001
