# Supplementary material for: Content-rich biological network constructed by mining PubMed abstracts
Source: BMC Bioinformatics. 2004 Oct 8;5:147. doi: 10.1186/1471-2105-5-147 (PMC528731; doi:10.1186/1471-2105-5-147)
Supplement: Additional File 2 — The original results of the above study (non-essential files are deleted to keep the file size under the limit set by BMC bioinformatics). [file 1471-2105-5-147-S2.bz2 › chilibotAdditionalFile2/dip05/44ID7545910E168/html/TAF4_TAF12.html]

 


 **TAF4** and **TAF12** 
  
Found 1 abstracts in PubMed, retrieved 1.  
 

 What does Google say? 
 PDF only 
| .edu only 

---

**Interactive relationship** (e.g. stimulation, inhibition, etc)

**Non-interactive relationship** (e.g. studied together, co-existance, homology, etc.)

- This indicates that either full length  **TAF4**  contains an unusually long connecting loop between its second and third helix, and this helix is not required for stable interaction with  **TAF12** , or that  **TAF4**  represents a novel class of partial histone fold motifs.  Ref: 12237304 J Biol Chem, 2002
