# Supplementary material for: Content-rich biological network constructed by mining PubMed abstracts
Source: BMC Bioinformatics. 2004 Oct 8;5:147. doi: 10.1186/1471-2105-5-147 (PMC528731; doi:10.1186/1471-2105-5-147)
Supplement: Additional File 2 — The original results of the above study (non-essential files are deleted to keep the file size under the limit set by BMC bioinformatics). [file 1471-2105-5-147-S2.bz2 › chilibotAdditionalFile2/dip05/46ID7597030E180/html/VP16_TAF9.html]

 


 **VP16** and **TAF9** 
  
Found 3 abstracts in PubMed, retrieved 3.  
 

 What does Google say? 
 PDF only 
| .edu only 

---

- Plant Cell, 1999   **Specific interactions with TBP and TFIIB in vitro suggest that 14 3 3 proteins may participate in the regulation of transcription when part of a DNA binding complex.**.
  The 14 3 3 family of multifunctional proteins is highly conserved among animals, plants, and yeast.
  Several studies have shown that these proteins are associated with a G box DNA binding complex and are present in the nucleus in several plant and animal species.
  In this study, 14 3 3 proteins are shown to bind the TATA box binding protein TBP , transcription factor IIB TFIIB , and the human TBP associated factor **[TAF9]** hTAF II 32 in vitro but not hTAF II 55.
  The interactions with TBP and TFIIB were highly specific, requiring amino acid residues in the box 1 domain of the 14 3 3 protein.
  These interactions do not require formation of the 14 3 3 dimer and are not dependent on known 14 3 3 recognition motifs containing phosphoserine.
  The 14 3 3 TFIIB interaction appears to occur within the same domain of TFIIB that binds the human herpes simplex virus transcriptional activator **VP16**, because **VP16** and 14 3 3 were able to compete for interaction with TFIIB in vitro.
  In a plant transient expression system, 14 3 3 was able to activate GAL4 dependent beta glucuronidase reporter gene expression at low levels when translationally fused with the GAL4 DNA binding domain.
  The in vitro binding with general transcription factors TBP and TFIIB together with its nuclear location provide evidence supporting a role for 14 3 3 proteins as transcriptional activators or coactivators when part of a DNA binding complex.

  - Proc Natl Acad Sci U S A, 1997   **Transactivation by CIITA, the type II bare lymphocyte syndrome associated factor, requires participation of multiple regions of the TATA box binding protein.**.
    CIITA is a positive regulator of class II major histocompatibility complex gene transcription that has been found to be defective in one of the five complementation groups of class II major histocompatibility complex negative cell lines.
    Its N terminal region is capable of activating transcription from a reporter gene when fused to a DNA binding domain.
    We have investigated the mechanism of transactivation mediated by the CIITA activation domain by studying its role in the process of transcription initiation and elongation.
    Specifically the altered specificity TBP TATA box binding protein assay has been used to analyze the response of the CIITA activation domain to mutations in TBP known to disrupt its interaction with its associated general factors.
    Transactivation by CIITA was extremely sensitive to a mutation in TBP that in yeast is known to abolish **VP16** mediated transcription but leaves basal transcription unaffected.
    A TBP mutant defective in interaction with TBP associated factor **[TAF9]** TAFII250 also failed to mediate transactivation through the CIITA activation domain.
    Certain interactions between TBP and general factors that are specifically required for acidic activation domains were also required for CIITA mediated transactivation to reach its full potential.
    Finally, like **VP16**, CIITA was able to stimulate elongation of transcription.
    Overall the mechanism of transactivation by the human B cell specific CIITA is very similar to that mediated by the herpes virus transactivator **VP16** in the ways that have been tested.

    - Cell, 1994   **Human TAFII30 is present in a distinct TFIID complex and is required for transcriptional activation by the estrogen receptor.**.
      We showed previously that coactivators mediating stimulation by different activators were associated with the TATA binding protein TBP in distinct TFIID complexes.
      We have characterized a human TBP associated factor **[TAF9]** TAF , hTAFII30, associated with a subset of TFIID complexes.
      hTAFII30 interacts with the AF 2 containing region E of the human estrogen receptor ER , but not with ER AF 1 or **VP16**.
      An antibody against hTAFII30 inhibited transcriptional stimulation by the ER AF 2 without affecting basal or **VP16** activated transcription and allowed the separation of TFIID complex es containing hTAFII30 from complexes mediating the activity of **VP16**.
      These results directly demonstrate the existence of functionally distinct TFIID populations that share common TAFIIs but differ in specific TAFIIs.
