# Supplementary material for: Content-rich biological network constructed by mining PubMed abstracts
Source: BMC Bioinformatics. 2004 Oct 8;5:147. doi: 10.1186/1471-2105-5-147 (PMC528731; doi:10.1186/1471-2105-5-147)
Supplement: Additional File 2 — The original results of the above study (non-essential files are deleted to keep the file size under the limit set by BMC bioinformatics). [file 1471-2105-5-147-S2.bz2 › chilibotAdditionalFile2/dip05/47ID7824954E188/html/SP1_GTF2B.html]

 


 **SP1** and **GTF2B** 
  
Found 32 abstracts in PubMed, retrieved 05.  
 

 What does Google say? 
 PDF only 
| .edu only 

---

**Interactive relationship** (e.g. stimulation, inhibition, etc)

**Non-interactive relationship** (e.g. studied together, co-existance, homology, etc.)

- Our results suggested that group IIb  [ **GTF2B** ]  metals Zn II, Cd II, and Hg II were able to complex with the peptide and bind the double stranded DNA with high affinity as well as inhibiting  **Sp1**  DNA binding activity in a concentration dependent manner.  Ref: 10873713 Toxicol Appl Pharmacol, 2000
- Chromatin immunoprecipitation revealed binding of NF Y,  **Sp1**  and TFIIB  [ **GTF2B** ]  to the promoter in vivo.  Ref: 12586348 FEBS Lett, 2003
- Here, we show that the p50 subunit as well as the p50 p65 of NF kappaB, and not other factors such as  **SP1** , TFIIB  [ **GTF2B** ] , polymerase II, TFIIA, or p65, can be acetylated by CBP p300 HAT domain.  Ref: 11739381 J Biol Chem, 2002
