# Supplementary material for: Content-rich biological network constructed by mining PubMed abstracts
Source: BMC Bioinformatics. 2004 Oct 8;5:147. doi: 10.1186/1471-2105-5-147 (PMC528731; doi:10.1186/1471-2105-5-147)
Supplement: Additional File 2 — The original results of the above study (non-essential files are deleted to keep the file size under the limit set by BMC bioinformatics). [file 1471-2105-5-147-S2.bz2 › chilibotAdditionalFile2/dip05/48ID7824954E190/html/VP16_GTF2B.html]

 


 **VP16** and **GTF2B** 
  
Found 65 abstracts in PubMed, retrieved 05.  
 

 What does Google say? 
 PDF only 
| .edu only 

---

**Interactive relationship** (e.g. stimulation, inhibition, etc)

**Neutral relationship**- This result implies DNA bending and looping of promoter DNA as a result of the physical interaction between GAL4  **VP16**  and an interface of the TBP TFIIA TFIIB  [ **GTF2B** ]  complex.  Ref: 12538582 J Biol Chem, 2003
- We show that the  **VP16**  activation domain directly interacts with TATA binding protein TBP, TFIIB  [ **GTF2B** ] , and the SAGA histone acetylase complex in vivo.  Ref: 12297514 J Biol Chem, 2002
- Site specific protein DNA photo cross linking was used to show that, when bound to its cognate site at various distances upstream of the TATA element, the chimeric transcriptional activator GAL4  **VP16**  can physically interact with a TATA box binding protein TBP transcription factor IIA TFIIA TFIIB  [ **GTF2B** ]  complex assembled on the TATA element.  Ref: 12538582 J Biol Chem, 2003
