# Supplementary material for: Content-rich biological network constructed by mining PubMed abstracts
Source: BMC Bioinformatics. 2004 Oct 8;5:147. doi: 10.1186/1471-2105-5-147 (PMC528731; doi:10.1186/1471-2105-5-147)
Supplement: Additional File 2 — The original results of the above study (non-essential files are deleted to keep the file size under the limit set by BMC bioinformatics). [file 1471-2105-5-147-S2.bz2 › chilibotAdditionalFile2/dip05/49ID7854423E192/html/SRF_GTF2F1.html]

 


 **SRF** and **GTF2F1** 
  
Found 2 abstracts in PubMed, retrieved 2.  
 

 What does Google say? 
 PDF only 
| .edu only 

---

**Interactive relationship** (e.g. stimulation, inhibition, etc)

**Neutral relationship**- Using a yeast interaction assay, we find that  **SRF**  binds the RAP74  [ **GTF2F1** ]  subunit of TFIIF  [ **GTF2F1** ] .  Ref: 7854423 Nature, 1995
- TFIIF  [ **GTF2F1** ]  could also relieve squelching by  **SRF**  in vitro, suggesting that  **SRF**  may directly bind TFIIF  [ **GTF2F1** ] .  Ref: 8106390 J Biol Chem, 1994
- We found more direct evidence for  **SRF**  TFIIF  [ **GTF2F1** ]  interaction by DNA binding assays where the RAP74  [ **GTF2F1** ]  subunit of TFIIF  [ **GTF2F1** ]  bound DNA in conjunction with  **SRF** , but not alone.  Ref: 8106390 J Biol Chem, 1994
- We have found that the general transcription factor TFIIF  [ **GTF2F1** ]  has an important role in serum response factor  [ **SRF** ]   **SRF**  activated transcription in vitro.  Ref: 8106390 J Biol Chem, 1994
- Interaction with RAP74  [ **GTF2F1** ]  subunit of TFIIF  [ **GTF2F1** ]  is required for transcriptional activation by serum response factor  [ **SRF** ] .  Ref: 7854423 Nature, 1995
- Further, RAP74  [ **GTF2F1** ]  s central charged cluster domain is required for binding to  **SRF**  s activation domain.  Ref: 7854423 Nature, 1995
- These results suggest that the mechanism of transcriptional activation by  **SRF** , and perhaps some other activators, involves their interaction with TFIIF  [ **GTF2F1** ] .  Ref: 8106390 J Biol Chem, 1994
- Here we show that the general transcription factor TFIIF  [ **GTF2F1** ]  is also a target for a transcriptional activator, namely serum response factor  [ **SRF** ]   **SRF** , which binds to the c fos promoter.  Ref: 7854423 Nature, 1995

**Non-interactive relationship** (e.g. studied together, co-existance, homology, etc.)

- Role of transcription factor TFIIF  [ **GTF2F1** ]  in serum response factor  [ **SRF** ]  activated transcription.  Ref: 8106390 J Biol Chem, 1994
- The correlation of  **SRF**  RAP74  [ **GTF2F1** ]  binding with transcriptional activation suggests that RAP74  [ **GTF2F1** ]  is a critical target for  **SRF**  activated transcription.  Ref: 7854423 Nature, 1995
- Deletion of this domain impairs RAP74  [ **GTF2F1** ]  s ability to support  **SRF**  activated transcription in vitro but has little effect on the protein s basal transcription activity or its ability to support SP1 activated transcription.  Ref: 7854423 Nature, 1995
