# Supplementary material for: Content-rich biological network constructed by mining PubMed abstracts
Source: BMC Bioinformatics. 2004 Oct 8;5:147. doi: 10.1186/1471-2105-5-147 (PMC528731; doi:10.1186/1471-2105-5-147)
Supplement: Additional File 2 — The original results of the above study (non-essential files are deleted to keep the file size under the limit set by BMC bioinformatics). [file 1471-2105-5-147-S2.bz2 › chilibotAdditionalFile2/dip05/52ID8278363E196/html/SP1_TAF4.html]

 


 **SP1** and **TAF4** 
  
Found 3 abstracts in PubMed, retrieved 3.  
 

 What does Google say? 
 PDF only 
| .edu only 

---

**Interactive relationship** (e.g. stimulation, inhibition, etc)

**Non-interactive relationship** (e.g. studied together, co-existance, homology, etc.)

- Coexpression of  **Sp1**  and TAFII130  [ **TAF4** ]  in cultured striatal cells from wild type and HD transgenic mice reverses the transcriptional inhibition of the dopamine D2 receptor gene caused by mutant huntingtin, as well as protects neurons from huntingtin induced cellular toxicity.  Ref: 11988536 Science, 2002
- **Sp1**  and TAFII130  [ **TAF4** ]  transcriptional activity disrupted in early Huntington s disease.  Ref: 11988536 Science, 2002
- Here, we report that huntingtin interacts with the transcriptional activator  **Sp1**  and coactivator TAFII130  [ **TAF4** ] .  Ref: 11988536 Science, 2002
