# Supplementary material for: Content-rich biological network constructed by mining PubMed abstracts
Source: BMC Bioinformatics. 2004 Oct 8;5:147. doi: 10.1186/1471-2105-5-147 (PMC528731; doi:10.1186/1471-2105-5-147)
Supplement: Additional File 2 — The original results of the above study (non-essential files are deleted to keep the file size under the limit set by BMC bioinformatics). [file 1471-2105-5-147-S2.bz2 › chilibotAdditionalFile2/dip05/54ID2198073E208/html/PNTA_PNTB.html]

 


 **PNTA** and **PNTB** 
  
Found 4 abstracts in PubMed, retrieved 4.  
 

 What does Google say? 
 PDF only 
| .edu only 

---

**Interactive relationship** (e.g. stimulation, inhibition, etc)

**Non-interactive relationship** (e.g. studied together, co-existance, homology, etc.)

- Nucleotide sequence of the  **pntA**  and  **pntB**  genes encoding the pyridine nucleotide transhydrogenase of Escherichia coli.  Ref: 3525165 Eur J Biochem, 1986
- Expression of the Escherichia coli  **pntA**  and  **pntB**  genes, encoding nicotinamide nucleotide transhydrogenase, in Saccharomyces cerevisiae and its effect on product formation during anaerobic glucose fermentation.  Ref: 10347010 Appl Environ Microbiol, 1999
- The two mutants,  **pntA**  and  **pntB** , are recessive and unlinked to each other.  Ref: 2414152 Genetics, 1985
- The  **pntA**  and  **pntB**  genes of Escherichia coli, encoding the alpha and beta subunits of the pyridine nucleotide transhydrogenase, were cloned individually in two different compatible plasmids into Escherichia coli mutants lacking transhydrogenase activity.  Ref: 3009227 FEBS Lett, 1986
- These sequences agree with those predicted from the nucleotide sequences of the  **pntA**  and  **pntB**  genes.  Ref: 3525165 Eur J Biochem, 1986
- The sequence contains two open reading frames,  **pntA**  and  **pntB**  of 1506 and 1386 base pairs, coding for the transhydrogenase alpha and beta subunits, respectively.  Ref: 3525165 Eur J Biochem, 1986
