# Supplementary material for: Content-rich biological network constructed by mining PubMed abstracts
Source: BMC Bioinformatics. 2004 Oct 8;5:147. doi: 10.1186/1471-2105-5-147 (PMC528731; doi:10.1186/1471-2105-5-147)
Supplement: Additional File 2 — The original results of the above study (non-essential files are deleted to keep the file size under the limit set by BMC bioinformatics). [file 1471-2105-5-147-S2.bz2 › chilibotAdditionalFile2/dip05/56ID8493578E211/html/RB1_E7.html]

 


 **RB1** and **E7** 
  
Found 222 abstracts in PubMed, retrieved 05.  
 

 What does Google say? 
 PDF only 
| .edu only 

---

**Interactive relationship** (e.g. stimulation, inhibition, etc)

**Non-interactive relationship** (e.g. studied together, co-existance, homology, etc.)

- Diffuse, strong positivity with p16 in endocervical adenocarcinomas is likely caused by inactivation of the retinoblastoma  [ **RB1** ]  protein by the  **E7**  human papillomavirus oncoprotein, which acts as a p16 transcript repressor.  Ref: 12819388 Int J Gynecol Pathol, 2003
- Surprisingly,  **E7**  expression in terminally differentiated myotubes could not reactivate DNA synthesis even though the oncogene bound the retinoblastoma  [ **RB1** ]  protein, reduced its levels, and increased E2F transcriptional activity.  Ref: 12821937 Oncogene, 2003
