# Supplementary material for: Content-rich biological network constructed by mining PubMed abstracts
Source: BMC Bioinformatics. 2004 Oct 8;5:147. doi: 10.1186/1471-2105-5-147 (PMC528731; doi:10.1186/1471-2105-5-147)
Supplement: Additional File 5 — The original Chilibot query results of the term "long-term potentiation (LTP)" and 22 other terms, limiting the latest references analyzed to the years 1990, 1995, 2000, and 2004. [file 1471-2105-5-147-S5.bz2 › chilibotAdditionalFile5/ltp1990/html/right.html]

 


### Explore the interaction map

The map to the left contains many clickable elements. Clicking on the boxes will open a portal site for the represented term. Clicking on the small circles will bring up sentences describing the interaction between the terms connected by the link.

### Legends

 **Nodes and links** 

|  |  |
| --- | --- |
|  | Symbol of Gene/Protein or keywords |
|  | Interactive relationship and its weight |
|  | Non-interactive relationship |
|  | Abstract co-occurrence only |

**Node Color**

With expression values:   


Without exression values, user selectable:  


**Link Color**

|  |  |
| --- | --- |
|  | Stimulatory relationship |
|  | Inhibitory relationship |
|  | Both stimulatory and inhibitory |
|  | Neither stimulatory nor inhibitory |
