# Supplementary material for: Content-rich biological network constructed by mining PubMed abstracts
Source: BMC Bioinformatics. 2004 Oct 8;5:147. doi: 10.1186/1471-2105-5-147 (PMC528731; doi:10.1186/1471-2105-5-147)
Supplement: Additional File 5 — The original Chilibot query results of the term "long-term potentiation (LTP)" and 22 other terms, limiting the latest references analyzed to the years 1990, 1995, 2000, and 2004. [file 1471-2105-5-147-S5.bz2 › chilibotAdditionalFile5/ltp1990/html/LTP.html]

 


**LTP** (Input: LTP ) 

---


|  |
| --- |
| **Google Searches:** Entire Web  | EDU domain only  | PDF files only |

.

|  |
| --- |
| **External Links:** OMIM | LocusLink | Swissprot | GeneCards |

  
**Maps of LTP**

|  |
| --- |
| Simple Complete graph in radiant tree square layout. |

**New Hypothesis !**

|  |
| --- |
|  |

**Synonyms** 

|  |
| --- |
| - ltp   [PubMed] |
| - long term potentiation   [PubMed] |

**Synopsis**

|  |
| --- |
| - The results show that PKC activity is involved in the early stage of **LTP** development and support the idea that the early phase of **LTP** represents the same modification process as that underlying the more sustained phase of **LTP**.  Brain Res, 1990    [22] |
| - We suggest that the contributions of presynaptic and postsynaptic processes to **LTP** maintenance may be determined by the differential distribution of PKC subtypes and substrates among hippocampal synaptic zones.  Brain Res Brain Res RevBrain Res Brain Res Rev, 1990    [16] |
| - Second, synergy of IP accumulation in correlation with synergy of neurotransmitter release elicited by mAChR activation and membrane depolarization, suggests a possible role for phospholipase C PLC in the bifurcating control of neurotransmitter release and for the involvement of PLC and voltage sensitive channels in mediation of long term potentiation  [**LTP**]  **LTP** .  Neurosci Lett, 1990    [16] |
| - The postsynaptic localization of gamma and beta II PKC in CA1 suggests that both PKC subspecies may correlate to long term potentiation  [**LTP**]  in the CA1 region contributing to the postsynaptic side.  Brain Res, 1990    [16] |
| - These data provide the first evidence linking two mechanisms associated with **LTP**, NMDA receptor activation and PKC substrate phosphorylation.  Brain Res, 1988    [14] |
| - These results strongly indicate the involvement of PKC system on the **LTP** formation in the SC slices.  Brain Res, 1990    [14] |
| - However, in contrast to a high dose of PMXB, a high dose of H 7 inhibited the persistence of **LTP** when delivered 240 min after HFS.  J Neurosci, 1990    [10] |
| - PKCmediates mechanisms underlying the maintenance of **LTP**.  Brain Res, 1988    [10] |
| - A possible link between PKC activation and DA release to processes of synaptic long term potentiation  [**LTP**]  is discussed.  Biomed Biochim Acta, 1989    [10] |
| - J Physiol, 1988    [10] |
| - Measurement of PKC activity in membrane and cytosol indicated that PKC activation is only associated with the persistence phase of **LTP**.  Brain Res, 1989    [10] |
| - Unsaturated fatty acids c FAs which activate purified PKC, when injected into hippocampus, enhance **LTP**.  Brain Res, 1989    [10] |
| - theprevented maintenance of electrically induced **LTP** by polymyxin B is in fact due to inhibition of this kinase.  Exp Brain Res, 1988    [10] |
| - Proteinkinase C PKC and substrate proteins such as F1 GAP 43 have been previously implicated in the synaptic plasticity of long term potentiation  [**LTP**]  **LTP** .  Brain Res, 1990    [10] |
| - These membrane vesicle alterations are postulated to underlie the increased synaptic efficacy which marks persistent **LTP**.  Brain Res, 1989    [10] |
