# Supplementary material for: Content-rich biological network constructed by mining PubMed abstracts
Source: BMC Bioinformatics. 2004 Oct 8;5:147. doi: 10.1186/1471-2105-5-147 (PMC528731; doi:10.1186/1471-2105-5-147)
Supplement: Additional File 5 — The original Chilibot query results of the term "long-term potentiation (LTP)" and 22 other terms, limiting the latest references analyzed to the years 1990, 1995, 2000, and 2004. [file 1471-2105-5-147-S5.bz2 › chilibotAdditionalFile5/ltp1990/html/SYNAPSIN I.html]

 


**SYNAPSIN I** (Input: SYNAPSIN I ) 

---


|  |
| --- |
| **Google Searches:** Entire Web  | EDU domain only  | PDF files only |

.

|  |
| --- |
| **External Links:** OMIM | LocusLink | Swissprot | GeneCards |

  
**Maps of SYNAPSIN I**

|  |
| --- |
| Simple Complete graph in radiant tree square layout. |

**New Hypothesis !**

|  |
| --- |
|  |

**Synonyms** 

|  |
| --- |
| - synapsin i   [PubMed] |

**Synopsis**

|  |
| --- |
| - The high degree of homology between the synapsins suggests that some of the functional properties of **synapsin I** are shared by synapsin II.  Bioessays, 1990    [19] |
| - The proteins phosphorylated by the kinase with the probable exception of **synapsin I** and tyrosine hydroxylase and the role of kinase autophosphorylation in vivo remain largely unknown.  Curr Top Cell Regul, 1990    [19] |
| - Thus, our results strongly suggest that **synapsin I** as reported for kinesin does NOT bind to the 4 kDa subtilisin digested C terminal part of the tubulin molecule.  Biochem Int, 1990    [17] |
| - Rather, these results are consistent with the possibility that dephospho **synapsin I** acts by a crosslinking mechanism involving some component s of the cytoskeleton, such as F actin, to create a dense network that restricts organelle movement.  J Neurosci, 1989    [11] |
| - Characterizationof **synapsin I** fragments produced by cysteine specific cleavage a study of their interactions with F actin.  J Cell Biol, 1989    [10] |
| - The tryptic phosphopeptide corresponds exactly to a sequence in the collagenase sensitive, proline rich tail region of bovine **synapsin I**.  J Biol Chem, 1990    [10] |
| - Domain C, the central homologous domain implicated in the binding of **synapsin I** to actin and to synaptic vesicles, is divided into nine exons.  J Biol Chem, 1990    [10] |
| - thephosphorylation of **synapsin I** may be involved in regulating the translocation of synaptic vesicles to their sites of release.  NatureNature, 1990    [10] |
| - Third, AtT 20 cells express the neuron specific phosphoprotein **synapsin I** which accumulates in the growth cones prior to contacts forming between growth cones and cells.  Eur J Cell Biol, 1989    [10] |
| - 2 **synapsin I** was an excellent substrate for CAM PK II,  J Neurochem, 1987    [9] |
| - thisdecrease was larger than the lesion induced change of **synapsin I** seen in the same region.  Synapse, 1989    [9] |
| - These results support the view that dephosphorylated **synapsin I** cages synaptic vesicles while CaM kinase II, by phosphorylating **synapsin I**, decages these organelles and increases their availability for release without affecting the release mechanism itself.  Proc Natl Acad Sci U S A, 1990    [8] |
| - wepropose that calcium entry into the nerve terminal activates calcium calmodulin dependent protein kinase II  [CAMKII] , which phosphorylates **synapsin I** on site II, dissociating it from the vesicles and thereby removing a constraint in the release process.  Proc Natl Acad Sci U S A, 1985    [7] |
| - Limited proteolysis of phosphorylated **synapsin I** by V8 protease, alpha chymotrypsin or collagenase, performed on the isolated dimer and monomer, allows us to localize tentatively in the central hydrophobic core of the molecule the cysteine residues the oxidation of which by copper o phenanthroline gives rise to synapsin dimers.  Biochem J, 1989    [7] |
| - Thus, the sequences surrounding the four sites that are phosphorylated by calcium calmodulin dependent protein kinase II  [CAMKII] , namely sites 2 and 3 in rat and bovine **synapsin I**, exhibit a high degree of homology.  Proc Natl Acad Sci U S A, 1987    [7] |
