# Supplementary material for: Content-rich biological network constructed by mining PubMed abstracts
Source: BMC Bioinformatics. 2004 Oct 8;5:147. doi: 10.1186/1471-2105-5-147 (PMC528731; doi:10.1186/1471-2105-5-147)
Supplement: Additional File 5 — The original Chilibot query results of the term "long-term potentiation (LTP)" and 22 other terms, limiting the latest references analyzed to the years 1990, 1995, 2000, and 2004. [file 1471-2105-5-147-S5.bz2 › chilibotAdditionalFile5/ltp1990/html/PLC.html]

 


**PLC** (Input: PLC ) 

---


|  |
| --- |
| **Google Searches:** Entire Web  | EDU domain only  | PDF files only |

.

|  |
| --- |
| **External Links:** OMIM | LocusLink | Swissprot | GeneCards |

  
**Maps of PLC**

|  |
| --- |
| Simple Complete graph in radiant tree square layout. |

**New Hypothesis !**

|  |
| --- |
|  |

**Synonyms** 

|  |
| --- |
| - plc   [PubMed] |

**Synopsis**

|  |
| --- |
| - Our results suggest further that PKC may exert regulatory effects by altering the relationship of **PLC** gamma to its associated P Tyr proteins.  Endocrinology, 1990    [23] |
| - These results indicate that the type III isozyme is responsible for the proliferative and antiproliferative actions and suggest that the unidentified isozyme s is involved in the inhibitory actions in the WBS induced **PLC** reactions and intracellular calcium mobilization in rabbit aortic SMC.  Biochem Biophys Res Commun, 1989    [22] |
| - The key role of **PLC** versus protein kinase C PKC is stressed by the fact that the inhibition of PKC with Hidaka s compound H 7 40 microM produced only a partial blockade about 25% of lectin mitogenic effect.  Biosci Rep, 1987    [18] |
| - Our results show that proteases such as trypsin and thrombin are able to stimulate membrane bound **PLC**, but this activation does NOT seem to be related to calpain.  Arch Biochem Biophys, 1990    [17] |
| - Activation of **PLC** may be important in modulating the well known effects of PTH on bone and kidney and may be relevant to recently described actions, such as the possible role of PTH as a growth factor in skeletal tissue.  Am J Physiol, 1990    [16] |
| - Second, synergy of IP accumulation in correlation with synergy of neurotransmitter release elicited by mAChR activation and membrane depolarization, suggests a possible role for phospholipase C **PLC** in the bifurcating control of neurotransmitter release and for the involvement of **PLC** and voltage sensitive channels in mediation of long term potentiation  [LTP]  LTP .  Neurosci Lett, 1990    [16] |
| - These observations suggest that **PLC** activation by ATP involves a G protein s that is NOT ADP ribosylated by pertussis toxin and further, that ATP activation of prostaglandin biosynthesis appears to involve a different, pertussis toxin sensitive, G protein.  Adv Exp Med Biol, 1990    [13] |
| - These findings suggest that **PLC** tau, and perhaps the 76 kDa co precipitated protein, are substrates of cyclic AMP dependent protein kinase in BALB c 3T3 cells however, the lack of effect of cyclic AMP elevation on PDGF stimulated inositol phosphate formation indicates that the intrinsic activity of **PLC** tau is unaltered by cyclic AMP mediated phosphorylation.  Biochem J, 1990    [13] |
| - Removal of genistein and PDGF resulted in DNA synthesis without the occurrence of **PLC** activation.  Science, 1990    [11] |
| - The differences in affinity for PIP2 parallel the ability of these three profilins to inhibit PIP2 hydrolysis by soluble phospholipase C **PLC** .  Cell Regul, 1990    [10] |
| - It is concluded that Fc gamma receptor induced activation of **PLC** and PLA2 triggers endocytosis by activation of PKC.  FEBS Lett, 1990    [10] |
| - amplificationof calcium release from endoplasmic reticulum by a highly cooperative step in the opening of calcium channels by InsP3, and calcium dependent feedback enhancement of **PLC** function.  Cell, 1989    [10] |
| - Moreover, the potent vasoconstrictor properties of endothelin on rabbit pulmonary veins involves activation of both **PLC** and PKC,  Pulm Pharmacol, 1990    [10] |
| - 3GnRH stimulation of **PLC** activity is most likely mediated by Gp whereas some calcium mobilizing ligands operate via Gi.  Endocr Rev, 1990    [10] |
| - These findings provide evidence that an increase in cytoplasmic calcium concentration is involved in the activation of glucose transport in skeletal muscle by **PLC** Cp.  J Biol Chem, 1989    [10] |
