# Supplementary material for: Content-rich biological network constructed by mining PubMed abstracts
Source: BMC Bioinformatics. 2004 Oct 8;5:147. doi: 10.1186/1471-2105-5-147 (PMC528731; doi:10.1186/1471-2105-5-147)
Supplement: Additional File 5 — The original Chilibot query results of the term "long-term potentiation (LTP)" and 22 other terms, limiting the latest references analyzed to the years 1990, 1995, 2000, and 2004. [file 1471-2105-5-147-S5.bz2 › chilibotAdditionalFile5/ltp1990/html/CREB.html]

 


**CREB** (Input: CREB ) 

---


|  |
| --- |
| **Google Searches:** Entire Web  | EDU domain only  | PDF files only |

.

|  |
| --- |
| **External Links:** OMIM | LocusLink | Swissprot | GeneCards |

  
**Maps of CREB**

|  |
| --- |
| Simple Complete graph in radiant tree square layout. |

**New Hypothesis !**

|  |
| --- |
|  |

**Synonyms** 

|  |
| --- |
| - creb   [PubMed] |

**Synopsis**

|  |
| --- |
| - These data suggest that cyclic AMP response element binding protein ATF **CREB** or related proteins activate V beta transcription.  Mol Cell Biol, 1989    [23] |
| - Certain cell types vary in their ATF 43 complement, suggesting that **CREB** activity is modulated in a cell type specific manner through interaction with ATF 43.  Mol Cell Biol, 1990    [19] |
| - The AP 1 activity binds efficiently to both AP 1 and activating transcription factor ATF cAMP response element binding protein **CREB** binding sites present in E1A inducible promoters and presumably plays a role in the transcriptional activation of adenovirus genes by E1A proteins and cAMP.  Genes Dev, 1989    [16] |
| - These results directly demonstrate that many different transcription factor binding sites, including the E1B TATA box, a **CREB** ATF binding site, and two E2F sites, can mediate E1A transactivation.  J Virol, 1989    [11] |
| - These results demonstrate that multiple mechanisms may regulate **CREB** binding, including variations in the sequences in the promoter binding site and the presence of related DNA binding proteins.  J Virol, 1990    [11] |
| - Thus, we propose that poliovirus infection inhibits transcription from the E3 promoter, at least in part, through the dephosphorylation of **CREB** ATF.  J Virol, 1990    [10] |
| - The ATF **CREB** motif may be a target for stimulation of HCMV gene expression through either viral or cellular transcription factors.  Arch Virol, 1990    [10] |
| - Whether the consensus **CREB** ATF sequence is associated with the cAMP mediated transcription of the CYP17 gene remains to be elucidated.  Arch Biochem Biophys, 1989    [10] |
| - A similar folding transition is observed on GCN4 p binding to the related ATF **CREB** site, which contains an additional central base pair.  Nature, 1990    [10] |
| - The CRE affinity purified 120 kDa protein displays properties distinct from those of the 43 kDa **CREB** ATF polypeptide.  J Biol Chem, 1990    [10] |
| - The peptide sequences 483 and 462 amino acids, respectively derived from each of these cDNAs are identical, except for the additional 21 amino acids in ATF a, but clearly differ from the other ATF **CREB** proteins reported.  Nucleic Acids Res, 1990    [9] |
| - onlyone of the two **CREB** proteins would heterodimerize with cJun and it would NOT form dimers with JunB or cFos.  Oncogene, 1990    [9] |
| - Thesequence motif CGTCA is critical for binding of a group of cellular transcription factors ATF, **CREB**, E4F, and EivF and for activation of certain E1a inducible and cyclic AMP cAMP inducible promoters.  Mol Cell Biol, 1989    [7] |
| - However, neither domain B alone, nor ATF **CREB** binding sites respond significantly to Tax1.  EMBO J, 1990    [7] |
| - These binding domains may bind TATA region binding factors site I, the **CREB** ATF protein site II, the AP 1 protein site III, and nuclear factor I CTF site IV .  Mol Cell Biol, 1988    [7] |
