# Supplementary material for: Content-rich biological network constructed by mining PubMed abstracts
Source: BMC Bioinformatics. 2004 Oct 8;5:147. doi: 10.1186/1471-2105-5-147 (PMC528731; doi:10.1186/1471-2105-5-147)
Supplement: Additional File 5 — The original Chilibot query results of the term "long-term potentiation (LTP)" and 22 other terms, limiting the latest references analyzed to the years 1990, 1995, 2000, and 2004. [file 1471-2105-5-147-S5.bz2 › chilibotAdditionalFile5/ltp1990/html/PKC.html]

 


**PKC** (Input: PKC ) 

---


|  |
| --- |
| **Google Searches:** Entire Web  | EDU domain only  | PDF files only |

.

|  |
| --- |
| **External Links:** OMIM | LocusLink | Swissprot | GeneCards |

  
**Maps of PKC**

|  |
| --- |
| Simple Complete graph in radiant tree square layout. |

**New Hypothesis !**

|  |
| --- |
|  |

**Synonyms** 

|  |
| --- |
| - pkc   [PubMed] |

**Synopsis**

|  |
| --- |
| - Taken together with previous cellular studies of brain showing parallel levels of expression of beta **PKC** mRNA and protein F1 mRNA, the present results make it attractive to propose that beta **PKC** regulates protein F1 phosphorylation during the development of synaptic plasticity.  Biochem Biophys Res Commun, 1990    [22] |
| - The results show that **PKC** activity is involved in the early stage of LTP development and support the idea that the early phase of LTP represents the same modification process as that underlying the more sustained phase of LTP.  Brain Res, 1990    [20] |
| - These data suggest that animals with lower hippocampal **PKC** activity may have problems performing spatial reference memory tasks with the same degree of accuracy as those with higher hippocampal **PKC** activity.  Brain Res, 1990    [20] |
| - We suggest that the contributions of presynaptic and postsynaptic processes to LTP maintenance may be determined by the differential distribution of **PKC** subtypes and substrates among hippocampal synaptic zones.  Brain Res Brain Res RevBrain Res Brain Res Rev, 1990    [18] |
| - Because proteins IIIa and IIIb are known to be phosphorylated by a calcium calmodulin CaM stimulated kinase, and protein F1 is known to be a plasma membrane associated protein P 57 which releases bound CaM in response to phosphorylation by **PKC**, the present findings suggest a potential mechanism in which **PKC** mediated changes in plasma membrane proteins produce CaM kinase mediated changes in synaptic vesicle proteins through a phosphorylation cascade.  Brain Res, 1989    [16] |
| - In addition, similarities in charge heterogeneity, 2 dimensional phosphopeptide maps, and increased phosphorylation in the presence of exogenous **PKC** or **PKC** stimulators suggest that protein F1 and 80k are highly homologous to, if NOT identical to, pp46 and pp80, respectively.  J Neurosci, 1989    [15] |
| - These data provide the first evidence linking two mechanisms associated with LTP, NMDA receptor activation and **PKC** substrate phosphorylation.  Brain Res, 1988    [14] |
| - These results strongly indicate the involvement of **PKC** system on the LTP formation in the SC slices.  Brain Res, 1990    [14] |
| - These results provide the first demonstration of a causal relationship between the **PKC** substrate B 50 and the release of neurotransmitter.  Nature, 1989    [14] |
| - This provides further evidence that **PKC** acts synergistically with the consequences of repetitive synaptic activation to maintain enhancement.  J Physiol, 1988    [12] |
| - PKCmediates mechanisms underlying the maintenance of LTP.  Brain Res, 1988    [10] |
| - A possible link between **PKC** activation and DA release to processes of synaptic long term potentiation  [LTP]  is discussed.  Biomed Biochim Acta, 1989    [10] |
| - The spike potentiation developed faster than that of the EPSP raising the possibility that PDBu activates two separate **PKC** dependent processes.  Exp Brain Res, 1988    [10] |
| - Thus both postsynaptic **PKC** and CaMKII are required for the induction of LTP and a presynaptic protein kinase appears to be necessary for the expression of LTP.  Science, 1989    [10] |
| - The relation between LTP and **PKC** activation is discussed.  Exp Neurol, 1988    [10] |
