# Supplementary material for: Content-rich biological network constructed by mining PubMed abstracts
Source: BMC Bioinformatics. 2004 Oct 8;5:147. doi: 10.1186/1471-2105-5-147 (PMC528731; doi:10.1186/1471-2105-5-147)
Supplement: Additional File 5 — The original Chilibot query results of the term "long-term potentiation (LTP)" and 22 other terms, limiting the latest references analyzed to the years 1990, 1995, 2000, and 2004. [file 1471-2105-5-147-S5.bz2 › chilibotAdditionalFile5/ltp1990/html/PKA.html]

 


**PKA** (Input: PKA ) 

---


|  |
| --- |
| **Google Searches:** Entire Web  | EDU domain only  | PDF files only |

.

|  |
| --- |
| **External Links:** OMIM | LocusLink | Swissprot | GeneCards |

  
**Maps of PKA**

|  |
| --- |
| Simple Complete graph in radiant tree square layout. |

**New Hypothesis !**

|  |
| --- |
|  |

**Synonyms** 

|  |
| --- |
| - pka   [PubMed] |

**Synopsis**

|  |
| --- |
| - The observations suggest that c fos protein is involved in PKC and **PKA** signal transduction in cultured human glial cells.  Histochemistry, 1990    [19] |
| - The diverse pattern of phosphorylation of AChR by **PKA** and PKC may play a role in the regulation of its function.  Biochemistry, 1990    [19] |
| - These results suggest that induction of phosphorylation of P65 and P74 by TNF and IL 1 is NOT mediated by PKC and **PKA** but may be mediated by another protein kinase and result in overlapping of biological activities between TNF and IL 1.  Cytokine, 1990    [17] |
| - They further suggest that the activity of PKC and **PKA** may be coordinately regulated in nontumorigenic cells.  Cancer Res, 1990    [16] |
| - UCN 01 has been shown to inhibit PKC and protein kinase A **PKA** with IC50 values of 0.0041 and 0.042 microM, respectively, and UCN 02 has been shown to inhibit PKC and **PKA** with IC50 values of 0.062 and 0.25 microM, respectively.  J Antibiot (Tokyo), 1989    [16] |
| - The **pKa** of Ala 1 approximately 8.8 and the relaxation parameters of individual carbon atoms T1, T2, and the nuclear Overhauser enhancement are generally similar, suggesting a similarity in the overall protein structure.  Biochemistry, 1986    [16] |
| - Here we show that the sites of phosphorylation by four kinases **PKA**, PKC, CK and CaMK all lie in the C terminal microtubule binding half of tau, but only the phosphorylation by CaM kinase shows the pronounced shift in electrophoretic mobility characteristic for tau from Alzheimer neurofibrillary tangles.  EMBO J, 1990    [16] |
| - Examination of the pH dependence of the ESR spectra for ficin and papain alkylated with an iodoacetamide or a maleimide spin label suggested that these enzymes have an amino acid residue of **pKa** 4 probably a histidine residue around the active site cysteine.  J Biochem (Tokyo), 1987    [16] |
| - Retention was shown to be due to a cation exchange mechanism and was controlled by the solute **pKa** and the degree and type of substitution at or near the basic centre.  J Chromatogr, 1987    [15] |
| - PKC inhibitor, H7, blocked effectively the PMA plus dbcAMP induced IL 1 beta production, while the protein kinase A **PKA** inhibitor, HA1004, had no effect, suggesting that **PKA** activation is NOT involved in the mechanism of action of cAMP in this case.  Blood, 1990    [13] |
| - **PKA** specific activity was lower in PCC4 cells than in C10 cells.  Cancer Res, 1989    [12] |
| - The changes in **PKA** and PKC activities greatly modified the PKC **PKA** ratios in the cytosols and the particulate fractions of cultured cells.  Mol Cell Endocrinol, 1990    [12] |
| - Tamoxifen inhibited PKC activity IC50 = 80 microM but increased **PKA** dependent protein phosphorylation.  Cell Signal, 1990    [10] |
| - Also, the model compounds gave absolute **pKa** values in good agreement with similar chemical species reported in the literature.  Int J Pept Protein Res, 1988    [10] |
| - The relationship between postulated changes in the PKC pathway and those hypothesized for the **PKA** pathway are discussed.  Development, 1990    [10] |
