# Supplementary material for: Content-rich biological network constructed by mining PubMed abstracts
Source: BMC Bioinformatics. 2004 Oct 8;5:147. doi: 10.1186/1471-2105-5-147 (PMC528731; doi:10.1186/1471-2105-5-147)
Supplement: Additional File 5 — The original Chilibot query results of the term "long-term potentiation (LTP)" and 22 other terms, limiting the latest references analyzed to the years 1990, 1995, 2000, and 2004. [file 1471-2105-5-147-S5.bz2 › chilibotAdditionalFile5/ltp1990/html/NMDA.html]

 


**NMDA** (Input: NMDA ) 

---


|  |
| --- |
| **Google Searches:** Entire Web  | EDU domain only  | PDF files only |

.

|  |
| --- |
| **External Links:** OMIM | LocusLink | Swissprot | GeneCards |

  
**Maps of NMDA**

|  |
| --- |
| Simple Complete graph in radiant tree square layout. |

**New Hypothesis !**

|  |
| --- |
|  |

**Synonyms** 

|  |
| --- |
| - nmda   [PubMed] |

**Synopsis**

|  |
| --- |
| - These data suggest that in vivo the reducing potential of local environments may interact with endogenous glycine to regulate **NMDA** receptor function.  Brain Res, 1990    [23] |
| - These results suggest that aging results in a decreased sensitivity to **NMDA** and impaired synaptic plasticity in the neocortex.  Brain Res, 1990    [23] |
| - These experiments show that activation of **NMDA** receptors is essential for certain kinds of learning.  Philos Trans R Soc Lond B Biol Sci, 1990    [21] |
| - The post stimulation application mode excludes the possibility that adenosine interferes with **NMDA** receptor activation and its role in initiating LTP.  Neurosci Lett, 1990    [19] |
| - Although the role of N methyl D aspartate **NMDA** receptors in the induction of LTP is well established, the nature of the persistent signal underlying this synaptic enhancement is unclear.  Nature, 1988    [18] |
| - The data indicate that an **NMDA** dependent process in the amygdala subserves associative fear conditioning.  Nature, 1990    [14] |
| - The results directly confirm the calcium rise predicted by **NMDA** receptor models of LTP induction.  Nature, 1990    [14] |
| - The results indicate that activation of **NMDA** receptor coupled channels causes a reduction of adenosine sensitivity.  Brain Res, 1990    [14] |
| - These data provide the first evidence linking two mechanisms associated with LTP, **NMDA** receptor activation and PKC substrate phosphorylation.  Brain Res, 1988    [14] |
| - These results demonstrate that glycine can facilitate induction of LTP probably by activating **NMDA** receptor.  Neurosci Lett, 1990    [14] |
| - Wepresent a biophysical model of electrical and calcium dynamics following activation of N methyl D aspartate **NMDA** receptors located on a dendritic spine.  Proc Natl Acad Sci U S A, 1990    [12] |
| - Our data indicate that the potentiation of both synaptic components requires an increase in intracellular calcium, involves activation of **NMDA** N methyl D aspartate receptors, and is specific to the tetanized pathway.  Nature, 1990    [11] |
| - These results ascertain the presence and identity of synaptic **NMDA** gated ion channels, which are assumed in the current hypothesis about excitotoxicity, long term potentiation  [LTP]  and learning.  Eur J Pharmacol, 1990    [11] |
| - Maskingeffect of **NMDA** receptor antagonists on the formation of long term potentiation  [LTP]  LTP in superior colliculus slices from the guinea pig.  Brain Res, 1990    [10] |
| - LTD is obtained if postsynaptic depolarization exceeds a critical level but remains below a threshold related to **NMDA** receptor gated conductances.  Nature, 1990    [10] |
