# Supplementary material for: Content-rich biological network constructed by mining PubMed abstracts
Source: BMC Bioinformatics. 2004 Oct 8;5:147. doi: 10.1186/1471-2105-5-147 (PMC528731; doi:10.1186/1471-2105-5-147)
Supplement: Additional File 5 — The original Chilibot query results of the term "long-term potentiation (LTP)" and 22 other terms, limiting the latest references analyzed to the years 1990, 1995, 2000, and 2004. [file 1471-2105-5-147-S5.bz2 › chilibotAdditionalFile5/ltp1990/html/AMPA.html]

 


**AMPA** (Input: AMPA ) 

---


|  |
| --- |
| **Google Searches:** Entire Web  | EDU domain only  | PDF files only |

.

|  |
| --- |
| **External Links:** OMIM | LocusLink | Swissprot | GeneCards |

  
**Maps of AMPA**

|  |
| --- |
| Simple Complete graph in radiant tree square layout. |

**New Hypothesis !**

|  |
| --- |
|  |

**Synonyms** 

|  |
| --- |
| - ampa   [PubMed] |

**Synopsis**

|  |
| --- |
| - Most attention has been directed to synapses using NMDA receptors, although more recent evidence indicates potential roles for the **AMPA** receptors as well.  Trends Pharmacol Sci, 1990    [21] |
| - The role of **AMPA** receptors in the maintenance of long term potentiation is discussed.  Trends Pharmacol Sci, 1990    [21] |
| - At physiological concentrations of magnesium 1.2 mM, **AMPA** was found to potentiate NMDA induced release of 3H arachidonic acid.  Brain Res Dev Brain Res, 1990    [20] |
| - These finding suggest that phospholipase A2 may regulate the **AMPA** quisqualate receptor and could play an important role in the development of LTP.  Neurosci Lett, 1990    [19] |
| - We found that the responses to quisqualate, kainate, and **AMPA** were NOT significantly affected by SP less than 20% increase .  Neurosci Lett, 1990    [15] |
| - These results further support the hypothesis that QA, **AMPA** and KA act on a common receptor type in striatal neurons.  Eur J Pharmacol, 1990    [14] |
| - The concentration response curve for **AMPA** was flattened the depolarizations in response to kainate or GABA were preserved.  Naunyn Schmiedebergs Arch Pharmacol, 1990    [11] |
| - Distributionof a kainate **AMPA** receptor mRNA in normal and Alzheimer brain.  Neuroreport, 1990    [11] |
| - oneof these is a unitary receptor that can respond to both kainate and **AMPA**.  Trends Pharmacol Sci, 1990    [10] |
| - No effect on neuronal damage induced by NMDA or **AMPA** could be detected.  J Neurochem, 1990    [10] |
| - 6 Cyano 7 nitroquinoxaline dione CNQX, QA at greater than 10 microM, and **AMPA**   J Neurosci, 1990    [10] |
| - Limited exposure to kainate or **AMPA** did little damage to the general neuronal population, but destroyed nearly all somatostatin or parvalbumin reactive cells.  Neurology, 1990    [10] |
| - The quisqualate receptor agonist RS alpha amino 3 hydroxy 5 methyl 4 isoxazolepropionic acid **AMPA** stimulated the efflux, about equally effectively as quisqualate.  Neuropharmacology, 1990    [10] |
| - The major actions of QQ on the discharge rate of ganglion cells are mimicked by **AMPA**.  J Neurophysiol, 1990    [10] |
| - This increase in calcium i could be evoked by quisqualate or glutamate after inhibition of the kainate response by **AMPA**.  Eur J Pharmacol, 1990    [10] |
