# Supplementary material for: Content-rich biological network constructed by mining PubMed abstracts
Source: BMC Bioinformatics. 2004 Oct 8;5:147. doi: 10.1186/1471-2105-5-147 (PMC528731; doi:10.1186/1471-2105-5-147)
Supplement: Additional File 5 — The original Chilibot query results of the term "long-term potentiation (LTP)" and 22 other terms, limiting the latest references analyzed to the years 1990, 1995, 2000, and 2004. [file 1471-2105-5-147-S5.bz2 › chilibotAdditionalFile5/ltp1990/html/TAU.html]

 


**TAU** (Input: TAU ) 

---


|  |
| --- |
| **Google Searches:** Entire Web  | EDU domain only  | PDF files only |

.

|  |
| --- |
| **External Links:** OMIM | LocusLink | Swissprot | GeneCards |

  
**Maps of TAU**

|  |
| --- |
| Simple Complete graph in radiant tree square layout. |

**New Hypothesis !**

|  |
| --- |
|  |

**Synonyms** 

|  |
| --- |
| - tau   [PubMed] |

**Synopsis**

|  |
| --- |
| - Exchange lifetime data and previously reported hydrogen deuterium exchange experiments suggest that the PRG histidine N **tau** H protons are NOT involved in hydrogen bonds.  Int J Pept Protein Res, 1988    [20] |
| - Modification of the CysF9 93 beta sulfhydryl group with iodoacetamide abolishes the pH dependence of 1 **tau**, suggesting that this sulfhydryl is involved in the isomerization process.  J Mol Biol, 1989    [19] |
| - we show that Hirano bodies bind antibodies to the microtubule associated protein **tau**, a component of Alzheimer neurofibrillary tangles.  Brain Res, 1987    [19] |
| - The role of MT and in particular of the MT associated protein **tau**, as demonstrated recently, confirms the involvement of the MT.  Ann Pathol, 1988    [19] |
| - Because one mole of actin purified from the red and white muscles of Leghorn chickens and one mole of myosin contain respectively one and two moles of N **tau** methylhistidine, and the molar ratio of myosin and actin in skeletal muscle is known to be 1 6, the myofibrillar myosin and actin contents of avian skeletal muscles can be determined from the amounts of protein bound N **tau** methylhistidine found in acid hydrolysates of this tissue.  Poult Sci, 1988    [16] |
| - In addition, using permeabilized lymphoma cells, we have found that 1 GTP or GTP **tau** S augments, and pertussis toxin inhibits, phospholipase C PLC activity and receptor capping.  J Immunol, 1990    [16] |
| - These results prove that the type I, II, and III PKC are products of PKC genes, **tau**, beta, and alpha, respectively.  Biochem Biophys Res Commun, 1987    [14] |
| - Quisqualate or kainate stimulation resulted only in an increase in **tau** immunoreactivity within axons.  Neurosci Lett, 1990    [14] |
| - The phi **tau** data correlate well with known singlet oxygen yields.  Photochem Photobiol, 1990    [14] |
| - These findings suggest that PLC **tau**, and perhaps the 76 kDa co precipitated protein, are substrates of cyclic AMP dependent protein kinase in BALB c 3T3 cells however, the lack of effect of cyclic AMP elevation on PDGF stimulated inositol phosphate formation indicates that the intrinsic activity of PLC **tau** is unaltered by cyclic AMP mediated phosphorylation.  Biochem J, 1990    [13] |
| - It was shown that, like bovine adrenal 190 kDa MAP, yet distinct from brain MAP2 and **tau**, purified HeLa 180 kDa MAP does NOT interact with actin filaments.  J Biochem (Tokyo), 1987    [12] |
| - It recovered from inactivation with a voltage dependent time course **tau** = 70 msec at 90 mV and 720 msec at 40 mV.  Circ Res, 1989    [10] |
| - Closed time analysis revealed brief **tau** c = 0.4 1.0 ms zinc insensitive gaps.  J Physiol, 1990    [10] |
| - The site is likely to be phosphorylated in **tau** from Alzheimer neurofibrillary tangles.  EMBO J, 1990    [10] |
| - Theprimary structure of all actins except that isolated from Naegleria gruberi contains a unique N **tau** methylhistidine MeHis at position 73.  J Biol Chem, 1987    [10] |
