# Supplementary material for: Content-rich biological network constructed by mining PubMed abstracts
Source: BMC Bioinformatics. 2004 Oct 8;5:147. doi: 10.1186/1471-2105-5-147 (PMC528731; doi:10.1186/1471-2105-5-147)
Supplement: Additional File 5 — The original Chilibot query results of the term "long-term potentiation (LTP)" and 22 other terms, limiting the latest references analyzed to the years 1990, 1995, 2000, and 2004. [file 1471-2105-5-147-S5.bz2 › chilibotAdditionalFile5/ltp1990/html/ARC.html]

 


**ARC** (Input: ARC ) 

---


|  |
| --- |
| **Google Searches:** Entire Web  | EDU domain only  | PDF files only |

.

|  |
| --- |
| **External Links:** OMIM | LocusLink | Swissprot | GeneCards |

  
**Maps of ARC**

|  |
| --- |
| Simple Complete graph in radiant tree square layout. |

**New Hypothesis !**

|  |
| --- |
|  |

**Synonyms** 

|  |
| --- |
| - arc   [PubMed] |

**Synopsis**

|  |
| --- |
| - The data suggests that activation of the Bezold Jarisch reflex by 5 HT involves a glutamatergic synapse presumably located within the brainstem vagal reflex **arc**.  Neuropharmacology, 1987    [23] |
| - These results indicate that mu receptors may be autoreceptors on **ARC** beta endorphin neurons.  Neuroendocrinology, 1990    [14] |
| - Besides stress fibers, **arc** like actin bundles have been detected in spreading cells.  J Cell Biol, 1984    [10] |
| - An important structural feature of these cells is an actin containing **arc** like band on the periphery of lamella.  Biull Eksp Biol Med, 1981    [10] |
| - The single protein precipitin **arc** of purified B protein comigrated with the radiolabeled FXIII from Hep G2 visualized by autoradiography, indicating both electrophoretic and antigenic identity.  Blood, 1986    [10] |
| - anEAA synapse in the CVLM is important in the cardiopulmonary reflex **arc**.  Exp Brain Res, 1989    [10] |
| - Step movements of amplitude M min **arc** and pulse movements of amplitude M min **arc** and pulse width tau s were studied.  Ophthalmic Physiol Opt, 1985    [9] |
| - The calcaneofibular ligament was essentially isometric in the neutral position throughout the flexion **arc**.  Foot Ankle, 1988    [9] |
| - Contractile **arc** actin filaments were revealed to be crosslinked by thin strands by the rapid freezing deep etching replication technique.  Proc Natl Acad Sci U S A, 1988    [9] |
| - However, it was very interesting that there were differences of **ARC** versus age between the man and woman groups,i.e., **ARC** in the man subgroup had lower values and in the woman subgroup, 4 had higher values than their respective older subgroups did.  Hua Xi Yi Ke Da Xue Xue Bao, 1989    [8] |
| - Plasma LH levels were NOT affected following microinfusion of NMDA 50 pmole into the AHY, VMH, and **ARC**.  Life Sci, 1988    [6] |
| - Interference reflection microscopy demonstrated that the spread cells were attached to the substratum in **arc** shaped regions, which corresponded to arcs containing alpha actinin as seen by specific immunofluorescence of the same cells.  Exp Cell Res, 1984    [6] |
