# Supplementary material for: Content-rich biological network constructed by mining PubMed abstracts
Source: BMC Bioinformatics. 2004 Oct 8;5:147. doi: 10.1186/1471-2105-5-147 (PMC528731; doi:10.1186/1471-2105-5-147)
Supplement: Additional File 5 — The original Chilibot query results of the term "long-term potentiation (LTP)" and 22 other terms, limiting the latest references analyzed to the years 1990, 1995, 2000, and 2004. [file 1471-2105-5-147-S5.bz2 › chilibotAdditionalFile5/ltp1990/html/KV4.2.html]

 


**KV4.2** (Input: KV4.2 ) 

---


|  |
| --- |
| **Google Searches:** Entire Web  | EDU domain only  | PDF files only |

.

|  |
| --- |
| **External Links:** OMIM | LocusLink | Swissprot | GeneCards |

  
**Maps of KV4.2**

|  |
| --- |
| Simple Complete graph in radiant tree square layout. |

**New Hypothesis !**

|  |
| --- |
|  |

**Synonyms** 

|  |
| --- |
| - kv4.2   [PubMed] |

**Synopsis**
