# Supplementary material for: Content-rich biological network constructed by mining PubMed abstracts
Source: BMC Bioinformatics. 2004 Oct 8;5:147. doi: 10.1186/1471-2105-5-147 (PMC528731; doi:10.1186/1471-2105-5-147)
Supplement: Additional File 5 — The original Chilibot query results of the term "long-term potentiation (LTP)" and 22 other terms, limiting the latest references analyzed to the years 1990, 1995, 2000, and 2004. [file 1471-2105-5-147-S5.bz2 › chilibotAdditionalFile5/ltp1990/html/ATF.html]

 


**ATF** (Input: ATF ) 

---


|  |
| --- |
| **Google Searches:** Entire Web  | EDU domain only  | PDF files only |

.

|  |
| --- |
| **External Links:** OMIM | LocusLink | Swissprot | GeneCards |

  
**Maps of ATF**

|  |
| --- |
| Simple Complete graph in radiant tree square layout. |

**New Hypothesis !**

|  |
| --- |
|  |

**Synonyms** 

|  |
| --- |
| - atf   [PubMed] |

**Synopsis**

|  |
| --- |
| - These data suggest that cyclic AMP response element binding protein **ATF** CREB or related proteins activate V beta transcription.  Mol Cell Biol, 1989    [23] |
| - **ATF** 43 polypeptides do NOT appear simply to correspond to the gene products of the **ATF** multigene family, suggesting that the size of the **ATF** family at the protein level is even larger than predicted from cDNA cloning studies.  Mol Cell Biol, 1990    [16] |
| - The AP 1 activity binds efficiently to both AP 1 and activating transcription factor **ATF** cAMP response element binding protein CREB binding sites present in E1A inducible promoters and presumably plays a role in the transcriptional activation of adenovirus genes by E1A proteins and cAMP.  Genes Dev, 1989    [16] |
| - **ATF** a and **ATF** a delta therefore represent two closely related members of a larger multigene family of proteins that interact with conserved promoter elements.  Nucleic Acids Res, 1990    [13] |
| - Whereas recent results demonstrate that EIIF activity can be modulated independently by EIV, leading to transactivation of this promoter, our results and those published previously strongly indicate that the three different transcription factors that bind to TAGA, EIIF, and **ATF** motifs of the EII early promoter are all targets for EIA regulation in vivo.  J Virol, 1990    [13] |
| - A third such family known as the CREB or **ATF** proteins, bind to a sequence element present in promoters from a number of viral and cellular genes.  Oncogene, 1990    [12] |
| - These results directly demonstrate that many different transcription factor binding sites, including the E1B TATA box, a CREB **ATF** binding site, and two E2F sites, can mediate E1A transactivation.  J Virol, 1989    [11] |
| - Thus, we propose that poliovirus infection inhibits transcription from the E3 promoter, at least in part, through the dephosphorylation of CREB **ATF**.  J Virol, 1990    [10] |
| - The **ATF** CREB motif may be a target for stimulation of HCMV gene expression through either viral or cellular transcription factors.  Arch Virol, 1990    [10] |
| - Whether the consensus CREB **ATF** sequence is associated with the cAMP mediated transcription of the CYP17 gene remains to be elucidated.  Arch Biochem Biophys, 1989    [10] |
| - A similar folding transition is observed on GCN4 p binding to the related **ATF** CREB site, which contains an additional central base pair.  Nature, 1990    [10] |
| - The CRE affinity purified 120 kDa protein displays properties distinct from those of the 43 kDa CREB **ATF** polypeptide.  J Biol Chem, 1990    [10] |
| - Such a factor, termed **ATF** adenovirus transcription factor, has already been characterized and appears to have strong similarities to the transcriptional factor CREB cAMP responsive element binding protein, which binds homologous sequences in cAMP responsive genes, such as somatostatin and c fos.  Proc Natl Acad Sci U S A, 1988    [9] |
| - We therefore propose that Tax1 induction of the 21 bp enhancer element requires interaction with the two different cellular proteins identified in this study HEB1 and HEB2, rather than binding of the **ATF** factor.  EMBO J, 1990    [7] |
| - Strainwas measured in the normal anterior talofibular ligament **ATF** and the calcaneofibular ligament CF using Hall effect strain transducers in five cadaveric ankles.  Foot Ankle, 1988    [7] |
