# Supplementary material for: Content-rich biological network constructed by mining PubMed abstracts
Source: BMC Bioinformatics. 2004 Oct 8;5:147. doi: 10.1186/1471-2105-5-147 (PMC528731; doi:10.1186/1471-2105-5-147)
Supplement: Additional File 5 — The original Chilibot query results of the term "long-term potentiation (LTP)" and 22 other terms, limiting the latest references analyzed to the years 1990, 1995, 2000, and 2004. [file 1471-2105-5-147-S5.bz2 › chilibotAdditionalFile5/ltp1990/html/CAMKII.html]

 


**CAMKII** (Input: CAMKII ) 

---


|  |
| --- |
| **Google Searches:** Entire Web  | EDU domain only  | PDF files only |

.

|  |
| --- |
| **External Links:** OMIM | LocusLink | Swissprot | GeneCards |

  
**Maps of CAMKII**

|  |
| --- |
| Simple Complete graph in radiant tree square layout. |

**New Hypothesis !**

|  |
| --- |
|  |

**Synonyms** 

|  |
| --- |
| - calcium calmodulin dependent protein kinase ii   [PubMed] |
| - camkii   [PubMed] |

**Synopsis**

|  |
| --- |
| - Among the protein molecules specifically located in presynaptic terminals, synapsin I and calcium calmodulin dependent protein kinase II  [**CAMKII**]  CaM kinase II have been shown to modulate evoked transmitter release in the squid giant synapse.  Proc Natl Acad Sci U S A, 1990    [16] |
| - Our data therefore indicate that certain nerve terminal populations in the rat brain contain high levels of calcium calmodulin dependent protein kinase II  [**CAMKII**] .  Synapse, 1989    [14] |
| - Phosphorylationof synaptosomal plasma membranes from rat hippocampus in the presence of the convulsant drug 4 aminopyridine resulted in the inhibition of the phosphorylation of the nervous tissue specific protein kinase C substrate protein B 50 48 kDa and the alpha subunit of calcium calmodulin dependent protein kinase II  [**CAMKII**]  50 kDa .  Biochem Biophys Res Commun, 1987    [11] |
| - Calciumcalmodulin dependent protein kinase II  [**CAMKII**] .  Curr Top Cell Regul, 1990    [10] |
| - 17 and **CaMKII** ref.  Nature, 1988    [10] |
| - Thus both postsynaptic PKC and **CaMKII** are required for the induction of LTP and a presynaptic protein kinase appears to be necessary for the expression of LTP.  Science, 1989    [10] |
| - phosphorylationof synapsin I on sites 2 and 3 by calcium calmodulin dependent protein kinase II  [**CAMKII**]  removes this inhibitory effect.  J Neurosci, 1989    [10] |
| - wepropose that calcium entry into the nerve terminal activates calcium calmodulin dependent protein kinase II  [**CAMKII**] , which phosphorylates synapsin I on site II, dissociating it from the vesicles and thereby removing a constraint in the release process.  Proc Natl Acad Sci U S A, 1985    [7] |
| - This bundling activity is reduced when synapsin I is phosphorylated by cAMP dependent protein kinase and virtually abolished when it is phosphorylated by calcium calmodulin dependent protein kinase II  [**CAMKII**]  or by both kinases.  NatureNature, 1985    [7] |
| - Thus, the sequences surrounding the four sites that are phosphorylated by calcium calmodulin dependent protein kinase II  [**CAMKII**] , namely sites 2 and 3 in rat and bovine synapsin I, exhibit a high degree of homology.  Proc Natl Acad Sci U S A, 1987    [7] |
| - Like cofilin, ADF contains a sequence similar to the nuclear transport signal sequence of the SV40 large T antigen and a calcium calmodulin dependent protein kinase II  [**CAMKII**]  phosphorylation consensus sequence.  Biochemistry, 1990    [7] |
| - Brief freezing of synaptosomes in the presence of gamma 32P ATP and either the catalytic subunit of cyclic AMP dependent protein kinase or calcium calmodulin dependent protein kinase II  [**CAMKII**]  rendered the synaptosomal interior accessible to these agents, as reflected by the phosphorylation of substrate proteins, such as synapsin I, which reside within the nerve terminal.  J Neurochem, 1989    [7] |
| - Synapsin I, phosphorylated at sites 2 and 3 by purified calcium calmodulin dependent protein kinase II  [**CAMKII**] , bound with a 5 fold lower affinity to the vesicles than did dephospho synapsin I.  J Biol Chem, 1986    [7] |
| - The most attractive candidates have been calcium calmodulin dependent protein kinase II  [**CAMKII**]  CaM KII refs 13 16, protein kinase C refs 17 19, and the calcium dependent protease, calpain.  Nature, 1989    [7] |
| - A protein kinase activity that copurifies with SBP SBP kinase was partially characterized and compared with calcium calmodulin dependent protein kinase II  [**CAMKII**]  CAM PK II .  J Neurochem, 1987    [6] |
