# Supplementary material for: Content-rich biological network constructed by mining PubMed abstracts
Source: BMC Bioinformatics. 2004 Oct 8;5:147. doi: 10.1186/1471-2105-5-147 (PMC528731; doi:10.1186/1471-2105-5-147)
Supplement: Additional File 5 — The original Chilibot query results of the term "long-term potentiation (LTP)" and 22 other terms, limiting the latest references analyzed to the years 1990, 1995, 2000, and 2004. [file 1471-2105-5-147-S5.bz2 › chilibotAdditionalFile5/ltp1990/html/ACTIN.html]

 


**ACTIN** (Input: ACTIN ) 

---


|  |
| --- |
| **Google Searches:** Entire Web  | EDU domain only  | PDF files only |

.

|  |
| --- |
| **External Links:** OMIM | LocusLink | Swissprot | GeneCards |

  
**Maps of ACTIN**

|  |
| --- |
| Simple Complete graph in radiant tree square layout. |

**New Hypothesis !**

|  |
| --- |
|  |

**Synonyms** 

|  |
| --- |
| - actin   [PubMed] |

**Synopsis**

|  |
| --- |
| - Since in other respects villin appears to be an unrelated protein, these results suggest the possibility that certain **actin** binding proteins may show immunologic cross reactivity due to convergent evolution within the acting binding domain.  J Cell Biochem, 1988    [20] |
| - SynapsinI plays an important role in the regulation of neurotransmitter release, since it binds to synaptic vesicles and to the cytoskeleton, and it bundles F **actin** and microtubules.  Biochem Int, 1990    [19] |
| - While the exact mechanism of staurosporine induced **actin** reorganization remains to be determined, the observed effects of staurosporine on PKC deficient cells make a role for PKC unlikely.  Exp Cell Res, 1990    [19] |
| - We showed that c myc beta **actin** ratios were as follows ethanol control, 100 14%.  J Biol Chem, 1989    [18] |
| - These findings suggest that SF induced epithelial mobility may be mediated, in part, by protein synthesis, alterations in protein phosphorylation ?inhibition of PKC, and **actin** filament reorganization.  J Cell Sci, 1990    [16] |
| - The finding that H 7 can elicit shape changes, **actin** polymerization and pinocytosis suggests that these events can occur without activation of protein kinase C PKC .  J Cell Sci, 1990    [16] |
| - Additional cytochemical and biochemical data indicate that the lymphoma 41 kDa protein is closely associated with several cytoskeletal proteins e.g., **actin**, myosin, and fodrin all of which colocalize under receptor cap structures.  J Immunol, 1990    [11] |
| - Rather, these results are consistent with the possibility that dephospho synapsin I acts by a crosslinking mechanism involving some component s of the cytoskeleton, such as F **actin**, to create a dense network that restricts organelle movement.  J Neurosci, 1989    [11] |
| - These results, demonstrating an interaction of synapsin I with **actin** in vitro, support the possibility that synapsin I is involved in clustering of synaptic vesicles at the presynaptic terminal.  NatureNature, 1989    [11] |
| - Phosphorylation of band 4.1 by PKC in vitro results in a dramatic reduction in band 4.1 binding to spectrin and **actin**, as well as to the cytoplasmic domain of band 3.  FEBS Lett, 1989    [11] |
| - In conclusion, three domains of synapsin I appear to be involved in F **actin** binding and bundling.  J Cell Biol, 1989    [10] |
| - In Acanthamoeba, the two isoforms of profilin may have specialized functions on the basis of their identical approximately 10 microM affinities for **actin** monomers and different affinities for PIP2.  Cell Regul, 1990    [10] |
| - Erythrocyte protein 4.1,   Biochem Biophys Res Commun, 1986    [10] |
| - inaddition, it bundles F **actin** and microtubules.  Biochem J, 1989    [10] |
| - acomparison of their structure and interactions with spectrin binding proteins ankyrin, **actin**, synapsin I, amelin, and calmodulin .  CRC Crit Rev Biochem, 1988    [10] |
