# Supplementary material for: Content-rich biological network constructed by mining PubMed abstracts
Source: BMC Bioinformatics. 2004 Oct 8;5:147. doi: 10.1186/1471-2105-5-147 (PMC528731; doi:10.1186/1471-2105-5-147)
Supplement: Additional File 5 — The original Chilibot query results of the term "long-term potentiation (LTP)" and 22 other terms, limiting the latest references analyzed to the years 1990, 1995, 2000, and 2004. [file 1471-2105-5-147-S5.bz2 › chilibotAdditionalFile5/ltp1990/html/ACTININ.html]

 


**ACTININ** (Input: ACTININ ) 

---


|  |
| --- |
| **Google Searches:** Entire Web  | EDU domain only  | PDF files only |

.

|  |
| --- |
| **External Links:** OMIM | LocusLink | Swissprot | GeneCards |

  
**Maps of ACTININ**

|  |
| --- |
| Simple Complete graph in radiant tree square layout. |

**New Hypothesis !**

|  |
| --- |
|  |

**Synonyms** 

|  |
| --- |
| - actinin   [PubMed] |

**Synopsis**

|  |
| --- |
| - These results lead us to suggest that this integrin alpha **actinin** linkage may contribute to the attachment of actin filaments to the membrane in certain locations.  J Cell Biol, 1990    [23] |
| - The authors suggest that the major function of actin, myosin, and alpha **actinin** containing filament bundles in mesangial cells is to create an isometric tension or minute isotonic contractions to counteract the distending forces of the rather high intracapillary hydraulic pressure and its resulting pressure gradients across the capillary wall and across the perimesangial GBM.  Am J Pathol, 1990    [20] |
| - On the basis of these results and considering the role of clathrin in intracellular transport and its capacity to interact with actin and alpha **actinin**, we suggest that clathrin may have diverse roles in the assembly, integrity, and functioning of the sarcomere and its integration with the sarcolemma.  Exp Cell Res, 1990    [20] |
| - These paracrystalline inclusions have been shown previously using immunocytochemistry to share epitopes with actin, tropomyosin, alpha **actinin** and vinculin.  Brain Res, 1987    [19] |
| - The other involves the actin binding protein, alpha **actinin**, which has been found to interact with several integrins.  Cell Differ Dev, 1990    [19] |
| - The cytoplasmic dense bodies, labeled with antibodies to alpha **actinin** exhibited a regular, diagonal arrangement in both extended cells and in cells shortened in solution to one fifth of their extended length after the same shortening, the fibrils of the cytoskeleton that showed colocalization with the dense bodies in extended cells became crumpled and disordered.  J Cell Biol, 1990    [16] |
| - A hexapeptide with sequence LKHAET, which occurs at the beginning of each of the repeated segments of actobindin, is very similar to sequences found in tropomyosin, muscle myosin heavy chain, paramyosin, and Dictyostelium alpha **actinin**.  J Biol Chem, 1990    [16] |
| - Also, no homology to the spectrin like segments which comprise most of the mass of spectrin, alpha **actinin**, and dystrophin was found.  Biochemistry, 1990    [15] |
| - Previousstudies have shown that cis unsaturated free fatty acids uFFAs are able to cause alterations in the normal distribution pattern of certain cytoskeletal proteins in lymphocytes, including tubulin, actin, alpha **actinin**, and myosin.  Cell Biophys, 1990    [13] |
| - Platelet suspensions exposed to bovine vWf without stirring ie, nonagglutinated or platelets in which agglutination was inhibited with ADP showed smaller cytoskeletons with little ABP, 235 Kd protein, and alpha **actinin**.  Blood, 1990    [12] |
| - An accumulation of alpha **actinin** at the Z bands characterizes nemalin myopathy.  Ann Pathol, 1988    [10] |
| - Homology to alpha **actinin** extends beyond domain I into the N terminal portion of domain II.  J Biol Chem, 1990    [10] |
| - The sequence presented represents the region of greatest homology among the spectrin supergene family spectrin, dystrophin, alpha **actinin** .  J Biol Chem, 1990    [10] |
| - The connecting structure may consist of two alpha **actinin** molecules linking actin filaments of opposite polarity.  J Cell Biol, 1990    [10] |
| - We thus conclude that, in vivo, alpha **actinin** acts as an actin gelling protein.  FEBS Lett, 1990    [10] |
