# Supplementary material for: Content-rich biological network constructed by mining PubMed abstracts
Source: BMC Bioinformatics. 2004 Oct 8;5:147. doi: 10.1186/1471-2105-5-147 (PMC528731; doi:10.1186/1471-2105-5-147)
Supplement: Additional File 5 — The original Chilibot query results of the term "long-term potentiation (LTP)" and 22 other terms, limiting the latest references analyzed to the years 1990, 1995, 2000, and 2004. [file 1471-2105-5-147-S5.bz2 › chilibotAdditionalFile5/ltp1990/html/SYNAPTOPHYSIN.html]

 


**SYNAPTOPHYSIN** (Input: SYNAPTOPHYSIN ) 

---


|  |
| --- |
| **Google Searches:** Entire Web  | EDU domain only  | PDF files only |

.

|  |
| --- |
| **External Links:** OMIM | LocusLink | Swissprot | GeneCards |

  
**Maps of SYNAPTOPHYSIN**

|  |
| --- |
| Simple Complete graph in radiant tree square layout. |

**New Hypothesis !**

|  |
| --- |
|  |

**Synonyms** 

|  |
| --- |
| - synaptophysin   [PubMed] |

**Synopsis**

|  |
| --- |
| - The stromal cells of cerebellar haemangioblastomas show foci of positive staining for S 100 protein, neuron specific enolase and **synaptophysin**, thereby clearly indicating their neuroectodermal origin.  Acta Neuropathol (Berl), 1990    [19] |
| - We have found that the intrinsic membrane proteins **synaptophysin** and p65 had an identical distribution and were restricted to low density fractions of the gradient which contained numerous clear microvesicles with a size range the same as that of small synaptic vesicles.  J Cell Biol, 1989    [15] |
| - Bindingsites for antibodies against membrane proteins of synaptic vesicles have been shown to be enhanced at nodes of Ranvier in electromotor axons of the electric ray Torpedo marmorata and sciatic nerve axons of the rat, using indirect immunofluorescence and monoclonal antibodies against the synaptic vesicle transmembrane proteins SV2 and **synaptophysin** rat or SV2 Torpedo .  Cell Tissue Res, 1989    [13] |
| - We used antibodies against two proteins found in synaptic terminals synapsin I and **synaptophysin** as synaptic markers in the hippocampal complexes of eight patients with autopsy proven AD and eight nondemented control subjects.  Neurology, 1989    [13] |
| - All tumors expressed the pan neuroendocrine markers **synaptophysin** and chromogranin A.  Am J Pathol, 1990    [10] |
| - The cDNA deduced amino acid sequence revealed the presence of an unusual carboxy terminus which has homologies to the C termini of Octopus rhodopsin and **synaptophysin**.  FEBS Lett, 1990    [10] |
| - None contained desmin, actin, cytokeratin, epithelial membrane antigen, or **synaptophysin**.  Arch Pathol Lab Med, 1990    [10] |
| - Both types of tumor giant cells stained for vimentin, alpha 1 antitrypsin, alpha 1 antichymotrypsin, **synaptophysin**, muscle actin, and neuron specific enolase,  Hum Pathol, 1990    [10] |
| - Finally, the synaptic zone, demonstrated with antibodies to the synaptic vesicle protein **synaptophysin**, contains few cytoskeletal elements.  J Neurocytol, 1988    [10] |
| - **Synaptophysin** is an intrinsic transmembrane glycoprotein.  J Neurosci, 1988    [10] |
| - Anintrinsic membrane protein of brain synaptic vesicles with Mr 3 00 p38, **synaptophysin** has recently been partially characterized Jahn, R.  J Cell Biol, 1986    [10] |
| - werestrongly reactive for neuron specific enolase, **synaptophysin**, and neuronal cytoskeletal proteins 68 and 200 kd subunits of neurofilament protein, microtubule associated protein 2, and tau .  Arch Pathol Lab Med, 1989    [10] |
| - Neuron, 1990    [10] |
| - Neurons were present, as determined by immunostaining with antibodies to 4 neuron specific proteins neuron specific enolase, microtubule associated protein 2, tau protein and **synaptophysin**.  Brain Res Dev Brain Res, 1990    [9] |
| - Synaptogenesis was confirmed by immunostaining the cells with antisera against synapsin I and **synaptophysin**, two proteins associated with synaptic vesicles.  J Neurochem, 1989    [9] |
