# Supplementary material for: Content-rich biological network constructed by mining PubMed abstracts
Source: BMC Bioinformatics. 2004 Oct 8;5:147. doi: 10.1186/1471-2105-5-147 (PMC528731; doi:10.1186/1471-2105-5-147)
Supplement: Additional File 5 — The original Chilibot query results of the term "long-term potentiation (LTP)" and 22 other terms, limiting the latest references analyzed to the years 1990, 1995, 2000, and 2004. [file 1471-2105-5-147-S5.bz2 › chilibotAdditionalFile5/ltp1990/html/LTP_PLC.html]

 


 **LTP** and **PLC** 
  
Found 2 abstracts in PubMed,  **2 abstracts were retrieved and analyzed**.  


---

 Search Google  |
 PDF files only 
|  EDU domain only 

---

**Interactive relationship** (e.g. stimulation, inhibition, etc)

**Parallel relationship** (e.g. studied together, co-existance, homology, etc.)

- Second, synergy of IP accumulation in correlation with synergy of neurotransmitter release elicited by mAChR activation and membrane depolarization, suggests a possible role for phospholipase C  **PLC**  in the bifurcating control of neurotransmitter release and for the involvement of  **PLC**  and voltage sensitive channels in mediation of long term potentiation  [ **LTP** ]   **LTP** .  Ref: 1969129 Neurosci Lett, 1990
