# Supplementary material for: Content-rich biological network constructed by mining PubMed abstracts
Source: BMC Bioinformatics. 2004 Oct 8;5:147. doi: 10.1186/1471-2105-5-147 (PMC528731; doi:10.1186/1471-2105-5-147)
Supplement: Additional File 5 — The original Chilibot query results of the term "long-term potentiation (LTP)" and 22 other terms, limiting the latest references analyzed to the years 1990, 1995, 2000, and 2004. [file 1471-2105-5-147-S5.bz2 › chilibotAdditionalFile5/ltp1990/html/LTP_PKC.html]

 


 **LTP** and **PKC** 
  
Found 28 abstracts in PubMed,  **28 abstracts were retrieved and analyzed**.  


---

 Search Google  |
 PDF files only 
|  EDU domain only 

---

**Interactive relationship** (e.g. stimulation, inhibition, etc)

- **PKC**  inhibitor treatment 10 min after  **LTP**  onset induced decay of responses to pre  **LTP**  baseline levels within 50 min of ejection.  Ref: 2825923 Brain Res, 1987
- that membrane  **PKC**  activity was increased by translocation from the cytosol following  **LTP**  ;.  Ref: 3828760 Brain Res, 1986
- Protein kinase C  **PKC**  activity is increased following hippocampal long term potentiation  [ **LTP** ]   **LTP** .  Ref: 2213144 J Neurosci, 1990
- Measurement of  **PKC**  activity in membrane and cytosol indicated that  **PKC**  activation is only associated with the persistence phase of  **LTP** .  Ref: 2765925 Brain Res, 1989
- Activation of protein kinase C  **PKC**  facilitates long term potentiation  [ **LTP** ]   **LTP** , a model of memory, and increases its substrate protein F1 aka GAP43 phosphorylation in direct relation to synaptic enhancement.  Ref: 2598047 Brain Res, 1989
- Unsaturated fatty acids c FAs which activate purified  **PKC** , when injected into hippocampus, enhance  **LTP** .  Ref: 2598047 Brain Res, 1989
- Both correlational evidence measures of  **PKC**  activity, protein F1 phosphorylation, and PI turnover and interventive evidence application of  **PKC**  inhibitors and activators indicate that  **PKC**  activation is necessary for maintenance of the  **LTP**  response.  Ref: 2679942 Brain Res Brain Res RevBrain Res Brain Res Rev, 1989
- The involvement of protein kinase C  **PKC**  mediated processes in mechanisms of long term potentiation  [ **LTP** ]   **LTP**  was suggested by recent studies which have demonstrated a correlation between  **PKC**  activation and  **LTP** .  Ref: 2833996 Brain Res, 1988
- Previous correlative and interventive work from this laboratory has suggested that activation of protein kinase C  **PKC**  is important for the maintenance of the hippocampal long term potentiation  [ **LTP** ]   **LTP**  response.  Ref: 2824717 J Neurosci, 1987
- Thus both postsynaptic  **PKC**  and CaMKII are required for the induction of  **LTP**  and a presynaptic protein kinase appears to be necessary for the expression of  **LTP** .  Ref: 2549638 Science, 1989
- A separate line of evidence has suggested that activation of protein kinase C  **PKC**  and the consequent phosphorylation of its substrates is necessary for the maintenance of the  **LTP**  response.  Ref: 2905192 Brain Res, 1988
- This free oleate then could act in synergy with processes that render  **PKC**  oleate sensitive to produce a persistent activation of  **PKC** , which is critical for and leads to the persistence of the  **LTP**  response.  Ref: 2824717 J Neurosci, 1987
- We have proposed that the translocation activation of protein kinase C  **PKC**  in synergism with a calcium mediated event plays an essential role in hippocampal long term potentiation  [ **LTP** ]   **LTP** .  Ref: 2765925 Brain Res, 1989
- Induction of  **LTP**  is blocked by intracellular delivery of H 7, a general protein kinase inhibitor, or  **PKC**  19 31, a selective protein kinase C  **PKC**  inhibitor, or CaMKII 273 302, a selective inhibitor of the multifunctional calcium calmodulin dependent protein kinase CaMKII.  Ref: 2549638 Science, 1989
- These results provide direct evidence that the  **PKC**  activation is not essential for the initial phase of  **LTP** , but is a necessary condition for a medium and a late, protein synthesis dependent phase in this monosynaptic pathway, i.e. for the maintenance of synaptic  **LTP** .  Ref: 2833996 Brain Res, 1988
- Protein kinase C  **PKC**  stimulators, 12 O tetradecanoyl phorbol 13 acetate TPA or cis unsaturated fatty acid UFA, have been shown to prolong synaptic enhancement induced by long term potentiation  [ **LTP** ]   **LTP** .  Ref: 3418528 J Physiol, 1988
- In mammalian brain  **PKC**  is present in high concentrations and has been shown to phosphorylate several substrate phosphoproteins, one of which may be involved in the generation of long term potentiation  [ **LTP** ]   **LTP** , a long lasting increase in synaptic efficacy evoked by brief, high frequency stimulation.  Ref: 3010137 NatureNature, 1988
- **PKC**  mediates mechanisms underlying the maintenance of  **LTP** .  Ref: 3179724 Brain Res, 1988
- Recent findings suggest that protein kinase C  **PKC**  regulates the persistence of long term potentiation  [ **LTP** ]   **LTP** .  Ref: 2825923 Brain Res, 1987
- The postsynaptic localization of gamma and beta II  **PKC**  in CA1 suggests that both  **PKC**  subspecies may correlate to long term potentiation  [ **LTP** ]  in the CA1 region contributing to the postsynaptic side.  Ref: 2202488 Brain Res, 1990
- Previous findings suggest 1 that altering protein kinase C  **PKC**  activity alters the persistence of long term potentiation  [ **LTP** ]   **LTP**  in the intact hippocampal formation.  Ref: 2790456 Brain Res, 1989
- Because phorbol esters have no effect on the initial potentiation produced with HFS, and because  **PKC**  activity appears to be related to the persistence of  **LTP**  and not to the initial change, we concluded that  **PKC**  regulates a post initiation component of  **LTP** .  Ref: 2213144 J Neurosci, 1990
- Regulation of neural protein kinase C  **PKC**  activity appears to directly affect the persistence of long term potentiation  [ **LTP** ]   **LTP** .  Ref: 2918368 J Neurosci, 1989
- Involvement of protein phosphorylation in  **LTP**  has been widely proposed, with protein kinase C  **PKC**  and calcium calmodulin kinase type II CaMKII as leading candidates.  Ref: 2847049 Nature, 1988
- The results show that  **PKC**  activity is involved in the early stage of  **LTP**  development and support the idea that the early phase of  **LTP**  represents the same modification process as that underlying the more sustained phase of  **LTP** .  Ref: 2207675 Brain Res, 1990
- Protein kinase C  **PKC**  is believed to have a crucial role in synaptic transmitter release and long term potentiation  [ **LTP** ] .  Ref: 2812003 Nature, 1989
- and 2 that  **PKC**  activity is directly correlated with persistence of  **LTP**  in vivo as measured by the in vitro phosphorylation of two major  **PKC**  substrates in adult hippocampus, protein F1 and 80k.  Ref: 2790456 Brain Res, 1989
- These findings, in conjunction with previous biochemical studies, raise the possibility that, in mammalian brain,  **PKC**  plays a role in controlling the release of neurotransmitter and may be involved in the generation of  **LTP** .  Ref: 3010137 NatureNature, 1989
- Protein kinase C  **PKC**  and substrate proteins such as F1 GAP 43 have been previously implicated in the synaptic plasticity of long term potentiation  [ **LTP** ]   **LTP** .  Ref: 2144782 Brain Res, 1990
- Inhibition of postsynaptic  **PKC**  or CaMKII blocks induction but not expression of  **LTP** .  Ref: 2549638 Science, 1989
- 1986 is mediated by  **PKC**  activation, we have tested polymyxin B as well as the potent  **PKC**  inhibitor K 252b during phorbol ester induced  **LTP** .  Ref: 2843394 Exp Brain Res, 1988

**Parallel relationship** (e.g. studied together, co-existance, homology, etc.)

- To determine if  **PKC**  stimulators prolong  **LTP**  by acting selectively at synapses given high frequency stimulation or by actions that are not synapse specific e.g. increased postsynaptic excitability we examined the effect of TPA or UFA on input selective enhancement.  Ref: 3418528 J Physiol, 1988
- Activation of protein kinase C  **PKC**  via neurotransmitter coupling processes has been associated with long term potentiation  [ **LTP** ]   **LTP**  or classical conditioning, but whether natural variation in  **PKC**  activity affects learning performance remains to be determined.  Ref: 2400904 Brain Res, 1990
- To study the role of protein kinase C  **PKC**  and its substrates in neuronal function, we have investigated the in vitro endogenous phosphorylation of the neuronal phosphoprotein F1 after induction of synaptic plasticity by long term potentiation  [ **LTP** ]   **LTP** .  Ref: 3794793 J Neurosci, 1986
- 1988, the protein kinase C  **PKC**  inhibitor polymyxin B prevents the maintenance of electrically induced long term potentiation  [ **LTP** ]   **LTP**  of synaptic transmission to CA1 neurons, indicating that posttranslational phosphorylation processes mediated by  **PKC**  are involved in mechanisms underlying this form of synaptic plasticity.  Ref: 2843394 Exp Brain Res, 1988
- Alternatively, if  **LTP**  maintenance is mediated by postsynaptic alteration, a signal contained within the dendritic spine would suffice to activate postsynaptic  **PKC**  mediated maintenance processes.  Ref: 2679942 Brain Res Brain Res RevBrain Res Brain Res Rev, 1988
- We determined whether the beta or gamma protein kinase C  **PKC**  subtypes implicated in long term potentiation  [ **LTP** ]   **LTP**  selectively regulates protein F1 phosphorylation.  Ref: 2145833 Biochem Biophys Res Commun, 1990
- A possible link between  **PKC**  activation and DA release to processes of synaptic long term potentiation  [ **LTP** ]  is discussed.  Ref: 2803280 Biomed Biochim Acta, 1989
- We suggest that the contributions of presynaptic and postsynaptic processes to  **LTP**  maintenance may be determined by the differential distribution of  **PKC**  subtypes and substrates among hippocampal synaptic zones.  Ref: 2679942 Brain Res Brain Res RevBrain Res Brain Res Rev, 1989
- Here we test whether the persistent signal in  **LTP**  is an enduring phosphoester bond, a long lived kinase activator, or a constitutively active protein kinase by using H 7, which inhibits activated protein kinases and sphingosine, which competes with activators of  **PKC**  ref.  Ref: 2847049 Nature, 1988
- Third, translocation of  **PKC**  activity to the membrane was significantly increased after oleate enhanced  **LTP**  relative to vehicle controls.  Ref: 2824717 J Neurosci, 1987
- One such study demonstrated that application of the cis unsaturated fatty acid, oleate, a newly discovered  **PKC**  activator, could prolong the time course of  **LTP** .  Ref: 2824717 J Neurosci, 1987
- In addition, the in vitro phosphorylation of a brain specific  **PKC**  substrate, protein F1 Mr 47 kDa, pl 4.5, has been directly correlated with persistence of  **LTP**  Lovinger et al.  Ref: 2918368 J Neurosci, 1989
- In  **LTP** , activation of this pathway may stabilize  **PKC**  in an activated state, and thus contribute to maintenance of the potentiated response.  Ref: 2679942 Brain Res Brain Res RevBrain Res Brain Res Rev, 1989
- To test the hypothesis that  **PKC**  inhibition would decrease persistence of potentiation we applied  **PKC**  inhibitors mellitin, polymyxin B, H 7 by micropressure ejection to the intact hippocampus either before or after  **LTP**  induction.  Ref: 2825923 Brain Res, 1987
- Whereas the EPSP  **LTP**  was fully blocked, some potentiation of the population spike still remained, suggesting the independence of  **PKC**  of the additional spike E S potentiation for the first 6 h.  Ref: 2833996 Brain Res, 1988
- The relation between  **LTP**  and  **PKC**  activation is discussed.  Ref: 2848709 Exp Neurol, 1988
- These results strongly indicate the involvement of  **PKC**  system on the  **LTP**  formation in the SC slices.  Ref: 1982236 Brain Res, 1990
- To determine if  **PKC**  activation is a consequence of NMDA receptor activation during  **LTP** , we applied the NMDA receptor antagonist drug, DL aminophosphonovalerate APV both immediately prior to and following high frequency stimulation, resulting in successful and unsuccessful blockade of  **LTP**  initiation, respectively.  Ref: 2905192 Brain Res, 1988
- To investigate the involvement of the protein kinase C  **PKC**  system on the  **LTP**  formation in the SC, the effects of  **PKC**  activator phorbol ester and  **PKC**  inhibitors polymyxin B, melittin and H 7 on the  **LTP**  formation have been studied.  Ref: 1982236 Brain Res, 1990
- **PKC**  inhibitors such as polymyxin B 10 7 M, melittin 10 8 M and H 7 10 4 M prevented  **LTP**  formation.  Ref: 1982236 Brain Res, 1990
- These data provide the first evidence linking two mechanisms associated with  **LTP** , NMDA receptor activation and  **PKC**  substrate phosphorylation.  Ref: 2905192 Brain Res, 1988
- Second, oleate enhancement of  **LTP**  was more potent when applied in the perforant path synaptic terminal zone than in the dentate hilus, implying that the site of oleate action is at the synapse where  **PKC**  is reported to be enriched.  Ref: 2824717 J Neurosci, 1987
- Therefore, we have examined the induction and maintenance of  **LTP**  in rat hippocampal slices in the presence of a relatively selective  **PKC**  inhibitor, using extracellular electrophysiological techniques.  Ref: 2833996 Brain Res, 1988
- The effect of the exogenous protein kinase C  **PKC**  activator phorbol 1 3 diacetate PDAc on the early 0 10 min time course of long term potentiation  [ **LTP** ]   **LTP**  has been studied in the CA1 region of the guinea pig hippocampal slice.  Ref: 2207675 Brain Res, 1990
- If  **LTP**  maintenance is mediated by presynaptic alteration, as has been indicated by measurement of glutamate release, then one must posit a signal that travels from the postsynaptic to the presynaptic membrane to activate presynaptic  **PKC** .  Ref: 2679942 Brain Res Brain Res RevBrain Res Brain Res Rev, 1990
- To define the time domain in which  **PKC**  activation is necessary for  **LTP** , we studied the effect of the  **PKC**  inhibitors polymyxin B PMXB and 1 5 isoquinolinesulfonyl 2 methylpiperazine H 7 micropressure ejected at different time points before and after the induction of  **LTP** .  Ref: 2213144 J Neurosci, 1990
- Our data are in line with current evidence from several laboratories that CaM and protein kinase C  **PKC**  dependent processes are involved in  **LTP**  and support the hypothesis that CaM mediates initiation.  Ref: 3179724 Brain Res, 1988
