# Supplementary material for: Content-rich biological network constructed by mining PubMed abstracts
Source: BMC Bioinformatics. 2004 Oct 8;5:147. doi: 10.1186/1471-2105-5-147 (PMC528731; doi:10.1186/1471-2105-5-147)
Supplement: Additional File 5 — The original Chilibot query results of the term "long-term potentiation (LTP)" and 22 other terms, limiting the latest references analyzed to the years 1990, 1995, 2000, and 2004. [file 1471-2105-5-147-S5.bz2 › chilibotAdditionalFile5/ltp1990/html/LTP_NMDA.html]

 


 **LTP** and **NMDA** 
  
Found 137 abstracts in PubMed,  **30 abstracts were retrieved and analyzed**.  


---

 Search Google  |
 PDF files only 
|  EDU domain only 

---

**Interactive relationship** (e.g. stimulation, inhibition, etc)

- Here we report that a component of  **LTP**  not requiring the activation of  **NMDA**  receptors can be induced in area CA1.  Ref: 1977084 Nature, 1990
- Induction of  **LTP**  requires calcium influx into postsynaptic neurons through ion channels gated by  **NMDA**  N methyl D aspartate receptors in hippocampus area CA1 and dentate gyrus and neocortex.  Ref: 1977084 Nature, 1990
- Although the induction of associational commissural  **LTP**  requires in the postsynaptic cell N methyl D aspartate  **NMDA**  receptor activation, membrane depolarization, and a rise in calcium, mossy fiber  **LTP**  does not.  Ref: 2114039 Science, 1990
- Release of arachidonic acid as a result of stimulation of  **NMDA**  receptors has been proposed to play a part in the establishment of long term potentiation  [ **LTP** ] .  Ref: 1975645 Nature, 1990
- **NMDA**  receptors appear to be directly involved in the induction of long term potentiation  [ **LTP** ]   **LTP**  at the hippocampal level, and quisqualate kainate receptors in the expression of  **LTP** .  Ref: 1983215 EncephaleEncephale, 1990
- The induction of long term potentiation  [ **LTP** ]   **LTP**  at hippocampal mossy fiber synapses requires an increase in postsynaptic calcium i but is independent of N methyl D aspartate  **NMDA**  receptor activation.  Ref: 2230936 J Neurophysiol, 1990
- Inescapable... stress inhibits the induction of Long Term Potentiation  [ **LTP** ]   **LTP**  in the CA1 region of hippocampus, a process that is dependent upon activation of the N methyl D aspartate  **NMDA**  subtype of glutamate receptor.  Ref: 2171955 Eur J Pharmacol, 1990
- Long term potentiation  [ **LTP** ]  of synaptic transmission in the hippocampal pyramidal cell, a form of neuronal plasticity that is thought to represent a cellular correlate of learning and memory, is dependent on calcium entry mediated by synaptic activation of glutamate receptors that have a high affinity for  **NMDA**  N methyl D aspartate and are located in distal dendrites.  Ref: 2169591 Nature, 1990
- Because  **LTP**  in this region is blocked by the  **NMDA**  N methyl D aspartate receptor antagonist AP5 DL 2 amino 5 phosphonovaleric acid and the calcium permeability of  **NMDA**  receptors is controlled by a voltage dependent magnesium block, a model has emerged that suggests that the calcium permeability of  **NMDA**  receptor coupled ion channels is the biophysical basis for  **LTP**  induction.  Ref: 1972782 Nature, 1990
- Antagonism of  **NMDA**  mediated transmission by MK 801 has been shown to block long term potentiation  [ **LTP** ]   **LTP**  in vitro and delay electrical kindling of the amygdala.  Ref: 2204470 Brain Res, 1990
- **NMDA**  antagonists such as AP5 prevent induction of long term potentiation  [ **LTP** ] , an activity dependent enhancement of synaptic efficacy mediated by neural mechanisms that might also underlie learning and memory.  Ref: 1972778 Nature, 1990

**Parallel relationship** (e.g. studied together, co-existance, homology, etc.)

- Two selective modulators of N methyl D aspartate  **NMDA**  receptor function, dithiothreitol DTT and glycine, each dramatically enhanced long term potentiation  [ **LTP** ]   **LTP**  in area CA1 of the hippocampus slice.  Ref: 1975761 Brain Res, 1990
- The  **NMDA**  antagonist, MK 801, suppresses long term potentiation  [ **LTP** ] , kindling, and kindling induced potentiation in the perforant path of the unanesthetized rat.  Ref: 2204470 Brain Res, 1990
- **LTP**  is induced at synapses that correlate in their activity, and the signal for induction is calcium influx through N methyl D aspartate  **NMDA**  receptor channels.  Ref: 1976253 Proc Natl Acad Sci U S A, 1990
- In most hippocampal and all neocortical pathways studied so far, the induction of  **LTP**  requires the activation of N methyl D aspartate  **NMDA**  receptor gated conductances.  Ref: 1975639 Nature, 1990
- These results ascertain the presence and identity of synaptic  **NMDA**  gated ion channels, which are assumed in the current hypothesis about excitotoxicity, long term potentiation  [ **LTP** ]  and learning.  Ref: 1981559 Eur J Pharmacol, 1990
- The results directly confirm the calcium rise predicted by  **NMDA**  receptor models of  **LTP**  induction.  Ref: 1972782 Nature, 1990
- Masking effect of  **NMDA**  receptor antagonists on the formation of long term potentiation  [ **LTP** ]   **LTP**  in superior colliculus slices from the guinea pig.  Ref: 1975212 Brain Res, 1990
- 2 Amino 5 phosphonovalerate APV blocked induction of  **LTP**  in both pathways, indicating that N methyl D aspartate  **NMDA**  receptor activation is required for induction.  Ref: 1978790 Brain Res, 1990
- The post stimulation application mode excludes the possibility that adenosine interferes with  **NMDA**  receptor activation and its role in initiating  **LTP** .  Ref: 2097583 Neurosci Lett, 1990
- Associative stimulation of N methyl D aspartate  **NMDA**  receptors and quisqualate ionotropic receptors Qi induces long term potentiation  [ **LTP** ]  at particular glutamatergic synapses.  Ref: 1975645 Nature, 1990
- These results demonstrate that glycine can facilitate induction of  **LTP**  probably by activating  **NMDA**  receptor.  Ref: 1981255 Neurosci Lett, 1990
- **NMDA**  dependent induction of long term potentiation  [ **LTP** ]  in afferent and association fiber systems of piriform cortex in vitro.  Ref: 1978790 Brain Res, 1990
- **NMDA**  depolarizations and long term potentiation  [ **LTP** ]  are reduced in the aged rat neocortex.  Ref: 2271944 Brain Res, 1990
