# Supplementary material for: Content-rich biological network constructed by mining PubMed abstracts
Source: BMC Bioinformatics. 2004 Oct 8;5:147. doi: 10.1186/1471-2105-5-147 (PMC528731; doi:10.1186/1471-2105-5-147)
Supplement: Additional File 5 — The original Chilibot query results of the term "long-term potentiation (LTP)" and 22 other terms, limiting the latest references analyzed to the years 1990, 1995, 2000, and 2004. [file 1471-2105-5-147-S5.bz2 › chilibotAdditionalFile5/ltp1990/html/LTP_AMPA.html]

 


 **LTP** and **AMPA** 
  
Found 5 abstracts in PubMed,  **5 abstracts were retrieved and analyzed**.  


---

 Search Google  |
 PDF files only 
|  EDU domain only 

---

**Interactive relationship** (e.g. stimulation, inhibition, etc)

- The expression of long term potentiation  [ **LTP** ]   **LTP**  in area CA1 of hippocampus has been proposed to result from an increased sensitivity of the  **AMPA**  quisqualate receptors.  Ref: 2177177 Neurosci Lett, 1990
- These finding suggest that phospholipase A2 may regulate the  **AMPA**  quisqualate receptor and could play an important role in the development of  **LTP** .  Ref: 2177177 Neurosci Lett, 1990

**Parallel relationship** (e.g. studied together, co-existance, homology, etc.)

- The role of  **AMPA**  receptors in the maintenance of long term potentiation  [ **LTP** ]  is also discussed.  Ref: 2167544 Trends Pharmacol Sci, 1990
