# Supplementary material for: Content-rich biological network constructed by mining PubMed abstracts
Source: BMC Bioinformatics. 2004 Oct 8;5:147. doi: 10.1186/1471-2105-5-147 (PMC528731; doi:10.1186/1471-2105-5-147)
Supplement: Additional File 5 — The original Chilibot query results of the term "long-term potentiation (LTP)" and 22 other terms, limiting the latest references analyzed to the years 1990, 1995, 2000, and 2004. [file 1471-2105-5-147-S5.bz2 › chilibotAdditionalFile5/ltp1990/html/SYNAPSIN I_PKC.html]

 


 **SYNAPSIN I** and **PKC** 
  
Found 1 abstracts in PubMed,  **1 abstracts were retrieved and analyzed**.  


---

 Search Google  |
 PDF files only 
|  EDU domain only 

---

- Endocrinology, 1989   **Protein kinase C catalyzed phosphorylation of sterol carrier protein 2.**.
  The transport of cholesterol to the inner mitochondrial membrane, a key step in steroidogenesis, is subject to hormonal modulation that, at least in part, could be mediated by protein phosphorylation.
  This step is stimulated by sterol carrier protein 2 SCP2 and Ca2ion .
  To explore whether SCP2 itself is a potential control point for regulation by Ca2ion dependent phosphorylation we investigated whether highly purified SCP2 could serve as a substrate for major type Ca2ion and non Ca2ion dependent protein kinases.
  Phosphorylation by calmodulin protein kinase II CaM PK II , myosin light chain kinase MLCK , cAMP dependent kinase PKA and protein kinase C **PKC** was monitored under optimal conditions for each enzyme.
  PKA, CaM PK II and MLCK catalyzed the radiolabeling of histone 2A, **synapsin I** and myosin light chain MLC , known substrates for these kinases, respectively, yet no phosphate transfer to SCP2 was observed.
  In contrast, **PKC** from two different sources rat and calf brain effectively catalyzed the phosphorylation of the highly purified SCP2.
  The phosphorylation of SCP2 depended on the addition of Ca2ion and phospholipids and was completely blocked by Polymyxin B, a **PKC** inhibitor.
  **PKC** catalyzed phosphorylation of SCP2 displayed a similar dependence on the concentration of ATP.
  Lineweaver Burk plots of the data indicate Km values for ATP of approximately 6 microM for the phosphorylation of SCP2.
  Our results, which have revealed for the first time that SCP2 is a substrate for **PKC**, are consistent with the possibilities that the control of steroidogenesis by tropic hormones and by **PKC** activation are mediated, at least in part, by the phosphorylation dephosphorylation of SCP2.
