# Supplementary material for: Content-rich biological network constructed by mining PubMed abstracts
Source: BMC Bioinformatics. 2004 Oct 8;5:147. doi: 10.1186/1471-2105-5-147 (PMC528731; doi:10.1186/1471-2105-5-147)
Supplement: Additional File 5 — The original Chilibot query results of the term "long-term potentiation (LTP)" and 22 other terms, limiting the latest references analyzed to the years 1990, 1995, 2000, and 2004. [file 1471-2105-5-147-S5.bz2 › chilibotAdditionalFile5/ltp1990/html/SYNAPSIN I_ACTIN.html]

 


 **SYNAPSIN I** and **ACTIN** 
  
Found 18 abstracts in PubMed,  **18 abstracts were retrieved and analyzed**.  


---

 Search Google  |
 PDF files only 
|  EDU domain only 

---

**Interactive relationship** (e.g. stimulation, inhibition, etc)

- These results, demonstrating an interaction of  **synapsin I**  with  **actin**  in vitro, support the possibility that  **synapsin I**  is involved in clustering of synaptic vesicles at the presynaptic terminal.  Ref: 3104800 NatureNature, 1989
- As purified from brain homogenates,  **synapsin I**  decreases the steady state viscosity of solutions containing F  **actin** , enhances the sedimentation of  **actin** , and bundles  **actin**  filaments.  Ref: 3115996 J Cell Biol, 1987
- To elucidate the structural basis for the interactions between  **synapsin I**  and F  **actin**  and how it relates to other characteristics of  **synapsin I** , we have performed a structure function analysis of fragments of  **synapsin I**  produced by cysteine specific cleavage with 2 nitro 5 thiocyanobenzoic acid.  Ref: 2497104 J Cell Biol, 1989
- **Synapsin I**  plays an important role in the regulation of neurotransmitter release, since it binds to synaptic vesicles and to the cytoskeleton, and it bundles F  **actin**  and microtubules.  Ref: 2129153 Biochem Int, 1990
- a comparison of their structure and interactions with spectrin binding proteins ankyrin,  **actin** ,  **synapsin I** , amelin, and calmodulin.  Ref: 3048888 CRC Crit Rev Biochem, 1988

**Parallel relationship** (e.g. studied together, co-existance, homology, etc.)

- Domain C, the central homologous domain implicated in the binding of  **synapsin I**  to  **actin**  and to synaptic vesicles, is divided into nine exons.  Ref: 2110562 J Biol Chem, 1990
- **Synapsin I**  is a neuronal phosphoprotein that can bundle  **actin**  filaments in vitro.  Ref: 3125185 J Cell Biochem, 1988
- **Synapsin I** , a neuron specific phosphoprotein interacting with small synaptic vesicles and F  **actin** .  Ref: 2517594 Cell Biol Int Rep, 1989
- Two complementary peptide fragments of synapsin generated by 2 nitro 5 thiocyanobenzoic cleavage and which map to opposite ends of the molecule participate in the bundling process, either by binding directly to  **actin**  or by binding to other  **synapsin I**  molecules.  Ref: 3115996 J Cell Biol, 1987
- Rather, these results are consistent with the possibility that dephospho  **synapsin I**  acts by a crosslinking mechanism involving some component s of the cytoskeleton, such as F  **actin** , to create a dense network that restricts organelle movement.  Ref: 2512374 J Neurosci, 1989
- **Synapsin I**  an  **actin**  bundling protein under phosphorylation control.  Ref: 3115996 J Cell Biol, 1987
- Because of these properties, it has been hypothesized that  **synapsin I**  acts as a dynamic link between synaptic vesicles an the  **actin**  meshwork of the nerve terminal, thereby modulating the release of neurotransmitter.  Ref: 2517594 Cell Biol Int Rep, 1989
- **Synapsin I**  isolated by this procedure retains its functional properties, demonstrated by the ability of  **synapsin I**  to stimulate the formation of a brain spectrin 240 235  **synapsin I**  F  **actin**  ternary complex as determined by a low shear falling ball viscometry assay.  Ref: 3094836 Brain Res Bull, 1986
- In contrast to phosphorylation of sites two and three in intact  **synapsin I** , which abolishes F  **actin**  bundling activity, phosphorylation of these sites in the middle tail fragment failed to abolish this activity.  Ref: 2497104 J Cell Biol, 1989
- We have examined the interaction of purified phosphorylated and unphosphorylated bovine and human  **synapsin I**  with tubulin and  **actin**  filaments, using cosedimentation, viscometric, electrophoretic, and morphologic assays.  Ref: 3115996 J Cell Biol, 1987
- A domain of  **synapsin I**  involved with  **actin**  bundling shares immunologic cross reactivity with villin.  Ref: 3125185 J Cell Biochem, 1988
- In conclusion, three domains of  **synapsin I**  appear to be involved in F  **actin**  binding and bundling.  Ref: 2497104 J Cell Biol, 1989
- A 51 54 kD middle tail fragment retained the F  **actin**  binding and bundling activity of  **synapsin I** , but the isolated tail fragment did not retain either activity.  Ref: 2497104 J Cell Biol, 1989
- **Synapsin I**  bundles F  **actin**  in a phosphorylation dependent manner.  Ref: 3104800 NatureNature, 1989
- We report here the ability of the dephospho form of  **synapsin I**  to bundle F  **actin** .  Ref: 3104800 NatureNature, 1989
- **Synapsin I**  is also able to interact with  **actin**  filaments in a phosphorylation dependent manner.  Ref: 2517594 Cell Biol Int Rep, 1989
- Erythrocyte protein 4.1... mimics the function of this brain factor using a reconstitution system including purified calspectin, calmodulin and F  **actin** .  Ref: 3096326 Biochem Biophys Res Commun, 1986
- Identification of a new 84 82 kDa calmodulin binding protein, which also interacts with  **actin**  filaments, tubulin and spectrin, as  **synapsin I** .  Ref: 3030806 FEBS Lett, 1987
- Characterization of  **synapsin I**  fragments produced by cysteine specific cleavage a study of their interactions with F  **actin** .  Ref: 2497104 J Cell Biol, 1989
- We propose that  **synapsin I**  links synaptic vesicles to  **actin**  filaments in the presynaptic nerve terminal.  Ref: 2117454 Bioessays, 1990
- In vivo,  **synapsin I**  may link small synaptic vesicles to the  **actin**  based cortical cytoskeleton, and coordinate their availability for release in a Ca dependent fashion.  Ref: 3115996 J Cell Biol, 1987
