# Supplementary material for: Content-rich biological network constructed by mining PubMed abstracts
Source: BMC Bioinformatics. 2004 Oct 8;5:147. doi: 10.1186/1471-2105-5-147 (PMC528731; doi:10.1186/1471-2105-5-147)
Supplement: Additional File 5 — The original Chilibot query results of the term "long-term potentiation (LTP)" and 22 other terms, limiting the latest references analyzed to the years 1990, 1995, 2000, and 2004. [file 1471-2105-5-147-S5.bz2 › chilibotAdditionalFile5/ltp1990/html/SYNAPSIN I_SYNAPTOPHYSIN.html]

 


 **SYNAPSIN I** and **SYNAPTOPHYSIN** 
  
Found 11 abstracts in PubMed,  **11 abstracts were retrieved and analyzed**.  


---

 Search Google  |
 PDF files only 
|  EDU domain only 

---

**Interactive relationship** (e.g. stimulation, inhibition, etc)

**Parallel relationship** (e.g. studied together, co-existance, homology, etc.)

- **Synaptophysin**  and  **synapsin I**  as tools for the study of the exo endocytotic cycle.  Ref: 2517595 Cell Biol Int Rep, 1989
- **Synaptophysin** , an integral protein of the synaptic vesicle membrane, and  **synapsin I** , a phosphoprotein associated with the cytoplasmic side of synaptic vesicles, represent useful markers that allow to follow the movements of the vesicle membrane during recycling.  Ref: 2517595 Cell Biol Int Rep, 1989
- The distribution of two synaptic vesicle specific phosphoproteins,  **synaptophysin**  and  **synapsin I** , during intense quantal secretion was studied by applying an immunogold labeling technique to ultrathin frozen sections.  Ref: 1967610 J Cell Biol, 1990
- Redistribution of  **synaptophysin**  and  **synapsin I**  during alpha latrotoxin induced release of neurotransmitter at the neuromuscular junction.  Ref: 1967610 J Cell Biol, 1990
- **Synapsin I**  and  **synaptophysin**  protein p38 are 2 major protein components of the membranes of small synaptic vesicles of virtually all presynaptic nerve endings.  Ref: 3130468 J Neurosci, 1988
- The synaptic vesicle proteins  **synapsin I**  and  **synaptophysin**  protein P38 are concentrated both in efferent and afferent nerve endings of the skeletal muscle.  Ref: 3130468 J Neurosci, 1988
- Synaptogenesis was also confirmed by immunostaining the cells with antisera against  **synapsin I**  and  **synaptophysin** , two proteins associated with synaptic vesicles.  Ref: 2564424 J Neurochem, 1989
- Secretory function of the vestibular nerve calyx suggested by presence of vesicles,  **synapsin I** , and  **synaptophysin** .  Ref: 3143814 J Neurosci, 1988
- In brains of patients with Parkinson disease or progressive supranuclear palsy with postmortem delays comparable to those of controls, the levels of these proteins as well as those of synaptic  **synapsin I**  and  **synaptophysin**  and glial glial fibrillary acidic protein and myelin basic protein markers were not significantly modified.  Ref: 2928345 Proc Natl Acad Sci U S A, 1989
- For this purpose, we have analysed the levels of two different synaptic vesicle associated proteins,  **synapsin I**  and protein p38 also called  **synaptophysin** , in the neostriatum after specific lesions.  Ref: 3142072 Synapse, 1988
- By immunogold electron microscopy carried out on bovine neurohypophysis we have found that three of these proteins,  **synapsin I** , Protein III, and  **synaptophysin**  protein p38 were concentrated on microvesicles but were not detectable in the membranes of neurosecretory granules.  Ref: 2513331 J Cell Biol, 1989
- We used antibodies against two proteins found in synaptic terminals  **synapsin I**  and  **synaptophysin**  as synaptic markers in the hippocampal complexes of eight patients with autopsy proven AD and eight nondemented control subjects.  Ref: 2927643 Neurology, 1989
