# Supplementary material for: Content-rich biological network constructed by mining PubMed abstracts
Source: BMC Bioinformatics. 2004 Oct 8;5:147. doi: 10.1186/1471-2105-5-147 (PMC528731; doi:10.1186/1471-2105-5-147)
Supplement: Additional File 5 — The original Chilibot query results of the term "long-term potentiation (LTP)" and 22 other terms, limiting the latest references analyzed to the years 1990, 1995, 2000, and 2004. [file 1471-2105-5-147-S5.bz2 › chilibotAdditionalFile5/ltp1990/html/PLC_PKC.html]

 


 **PLC** and **PKC** 
  
Found 18 abstracts in PubMed,  **18 abstracts were retrieved and analyzed**.  


---

 Search Google  |
 PDF files only 
|  EDU domain only 

---

**Interactive relationship** (e.g. stimulation, inhibition, etc)

- It is concluded that Fc gamma receptor induced activation of  **PLC**  and PLA2 triggers endocytosis by activation of  **PKC** .  Ref: 2147663 FEBS Lett, 1990
- Under those conditions, AVP induced PGI2 production did not correlate either with PMA induced membranous  **PKC**  activity or with AVP induced  **PLC**  activation.  Ref: 2113056 J Biol Chem, 1990
- **PLC**  Cp and PMA both caused a approximately 2 fold increase in membrane associated  **PKC**  activity.  Ref: 2600081 J Biol Chem, 1989
- The different effect of  **PKC**  stimulation on PI metabolism in Jurkat cells and T lymphoblasts reveals heterogeneity of  **PLC**  regulation in T lymphocytes.  Ref: 2158513 J Immunol, 1990
- Protein kinase C  **PKC**  and myosin light chain kinase MLCK, which are physiologically activated by  **PLC**  triggered second messengers diacylglycerol and calcium ions, respectively, phosphorylate specific proteins closely involved in the cell functional responses.  Ref: 2124475 Arch Mal Coeur Vaiss, 1990
- Exposure of endothelial monolayers to phospholipase C  **PLC** , which increases membrane phosphatidylinositide turnover, or to alpha thrombin also induced concentration dependent activation of  **PKC**  and increases in 125I albumin endothelial permeability.  Ref: 2347922 J Clin Invest, 1990
- The attendant diacylglycerol release and activation of  **PKC**  may terminate  **PLC**  gamma activation, in part by inhibiting  **PLC**  gamma phosphorylation by the EGF receptor.  Ref: 1698145 Endocrinology, 1990
- Our results suggest further that  **PKC**  may exert regulatory effects by altering the relationship of  **PLC**  gamma to its associated P Tyr proteins.  Ref: 1698145 Endocrinology, 1990
- We propose, therefore, that rather than having a direct effect on enzyme activity, the phosphorylation of  **PLC**  beta by  **PKC**  may alter its interaction with a putative guanine nucleotide binding regulatory protein and thereby prevent its activation.  Ref: 2211670 J Biol Chem, 1990
- inhibition of  **PKC**  activity did not prevent desensitization of  **PLC**  by PAF.  Ref: 2761347 Life Sci, 1989
- Activation of protein kinase C  **PKC**  with a phorbol ester inhibited IC50 = 3 10 nM both EGF dependent PtdIns hydrolysis and  **PLC**  gamma phosphorylation by more than 90%.  Ref: 1698145 Endocrinology, 1990

**Parallel relationship** (e.g. studied together, co-existance, homology, etc.)

- The key role of  **PLC**  versus protein kinase C  **PKC**  is stressed by the fact that the inhibition of  **PKC**  with Hidaka s compound H 7 40 microM produced only a partial blockade about 25% of lectin mitogenic effect.  Ref: 3427220 Biosci Rep, 1987
- The proliferative and antiproliferative actions of  **PKC**  were abolished in parallel with the loss of the down regulation sensitive component of  **PKC** , but the inhibitory actions in the whole blood serum WBS induced phospholipase C  **PLC**  reactions and intracellular calcium mobilization were not affected.  Ref: 2742575 Biochem Biophys Res Commun, 1989
- Moreover, the potent vasoconstrictor properties of endothelin on rabbit pulmonary veins involves activation of both  **PLC**  and  **PKC** , but not PLA2.  Ref: 2135207 Pulm Pharmacol, 1990
- Phosphorylation of bovine brain  **PLC**  beta by  **PKC**  in vitro resulted in a stoichiometric incorporation of phosphate at serine 887, without any concomitant effect on  **PLC**  beta activity.  Ref: 2211670 J Biol Chem, 1990
- To determine whether or not the targets of TPA activated  **PKC**  include one or more isozymes of  **PLC** , studies were carried out with PC12, C6Bu1, and NIH 3T3 cells, which contain at least three  **PLC**  isozymes,  **PLC**  beta,  **PLC**  gamma, and  **PLC**  delta.  Ref: 2211670 J Biol Chem, 1990
- This study demonstrates that PAF stimulated  **PLC**  activity is negatively affected by protein kinase C  **PKC**  activation.  Ref: 2761347 Life Sci, 1989
- In this study, we have examined and compared between platelets of spontaneously hypertensive rats SHR and their normotensive controls Wistar Kyoto WKY, the patterns of protein phosphorylation obtained either with the receptor mediated agonist thrombini.e. which acts via  **PLC**  or with direct activators of the protein kinases,  **PKC**  and MLCK.  Ref: 2124475 Arch Mal Coeur Vaiss, 1990
- Thus, the possibility is raised that, while inhibiting AVP induced  **PLC**  activation, PMA induced  **PKC**  activation increases the calcium sensitivity of the cellular signaling system leading to PGI2 production.  Ref: 2113056 J Biol Chem, 1990
- Treatment of a variety of cells and tissues with 12 O tetradecanoylphorbol 13 acetate TPA, an activator of protein kinase C  **PKC**  results in the inhibition of receptor coupled inositol phospholipid specific phospholipase C  **PLC**  activity.  Ref: 2211670 J Biol Chem, 1990
- The activation of endothelial  **PKC**  directly by PMA or OAG and by  **PLC**  and alpha thrombin increases the transendothelial albumin permeability, indicating that  **PKC**  activation is an important signal transduction pathway by which extracellular mediators increase endothelial macromolecular transport.  Ref: 2347922 J Clin Invest, 1990
- Platelet derived growth factor PDGF induction of DNA synthesis is believed to involve activation of phospholipase C  **PLC**  and subsequent accumulation of inositol, 5 triphosphate I, 5 P3, increase in intracellular calcium, activation of protein kinase C  **PKC** , and receptor down regulation.  Ref: 2163545 Science, 1990
