# Supplementary material for: Content-rich biological network constructed by mining PubMed abstracts
Source: BMC Bioinformatics. 2004 Oct 8;5:147. doi: 10.1186/1471-2105-5-147 (PMC528731; doi:10.1186/1471-2105-5-147)
Supplement: Additional File 5 — The original Chilibot query results of the term "long-term potentiation (LTP)" and 22 other terms, limiting the latest references analyzed to the years 1990, 1995, 2000, and 2004. [file 1471-2105-5-147-S5.bz2 › chilibotAdditionalFile5/ltp1990/html/PLC_AMPA.html]

 


 **PLC** and **AMPA** 
  
Found 1 abstracts in PubMed,  **1 abstracts were retrieved and analyzed**.  


---

 Search Google  |
 PDF files only 
|  EDU domain only 

---

**Interactive relationship** (e.g. stimulation, inhibition, etc)

- **PLC**  from C. perfringens produced an increased affinity of the quisqualate DL alpha amino 3 hydroxy 5 methylisoxazole 4 propionic acid  **AMPA**  receptor for its ligand.  Ref: 2154675 Mol Pharmacol, 1990
- In contrast to  **PLC**  from C. perfringens, phosphatidylinositol specific  **PLC**  treatment did not detectably modify the binding properties of the quisqualate  **AMPA**  receptor or the NMDA receptor channel.  Ref: 2154675 Mol Pharmacol, 1990
