# Supplementary material for: Content-rich biological network constructed by mining PubMed abstracts
Source: BMC Bioinformatics. 2004 Oct 8;5:147. doi: 10.1186/1471-2105-5-147 (PMC528731; doi:10.1186/1471-2105-5-147)
Supplement: Additional File 5 — The original Chilibot query results of the term "long-term potentiation (LTP)" and 22 other terms, limiting the latest references analyzed to the years 1990, 1995, 2000, and 2004. [file 1471-2105-5-147-S5.bz2 › chilibotAdditionalFile5/ltp1990/html/PLC_TAU.html]

 


 **PLC** and **TAU** 
  
Found 3 abstracts in PubMed,  **3 abstracts were retrieved and analyzed**.  


---

 Search Google  |
 PDF files only 
|  EDU domain only 

---

**Interactive relationship** (e.g. stimulation, inhibition, etc)

- In contrast, activation of G proteins with GTP  **tau**  S or AlF4 stimulates both prostaglandin synthesis and  **PLC** .  Ref: 2122640 Adv Exp Med Biol, 1990

- :-)

  **Parallel relationship** (e.g. studied together, co-existance, homology, etc.)

  - These findings suggest that  **PLC**   **tau** , and perhaps the 76 kDa co precipitated protein, are substrates of cyclic AMP dependent protein kinase in BALB c 3T3 cells however, the lack of effect of cyclic AMP elevation on PDGF stimulated inositol phosphate formation indicates that the intrinsic activity of  **PLC**   **tau**  is unaltered by cyclic AMP mediated phosphorylation.  Ref: 1702622 Biochem J, 1990
  - Previous studies have demonstrated enhanced phosphorylation of phospholipase C  **tau**   **PLC**   **tau** , a key regulatory enzyme in phosphoinositide metabolism, in cells treated with platelet derived growth factor PDGF and epidermal growth factor, both of which act via specific receptor tyrosine kinases.  Ref: 1702622 Biochem J, 1990
  - Treatment of cells with cyclic AMP agonists also enhanced, with similar kinetics, the phosphorylation of a 76 kDa protein co precipitated by anti  **PLC**   **tau**  monoclonal antibodies.  Ref: 1702622 Biochem J, 1990
  - In addition, using permeabilized lymphoma cells, we have found that 1 GTP or GTP  **tau**  S augments, and pertussis toxin inhibits, phospholipase C  **PLC**  activity and receptor capping.  Ref: 1968926 J Immunol, 1990
