# Supplementary material for: Content-rich biological network constructed by mining PubMed abstracts
Source: BMC Bioinformatics. 2004 Oct 8;5:147. doi: 10.1186/1471-2105-5-147 (PMC528731; doi:10.1186/1471-2105-5-147)
Supplement: Additional File 5 — The original Chilibot query results of the term "long-term potentiation (LTP)" and 22 other terms, limiting the latest references analyzed to the years 1990, 1995, 2000, and 2004. [file 1471-2105-5-147-S5.bz2 › chilibotAdditionalFile5/ltp1990/html/PLC_ARC.html]

 


 **PLC** and **ARC** 
  
Found 1 abstracts in PubMed,  **1 abstracts were retrieved and analyzed**.  


---

 Search Google  |
 PDF files only 
|  EDU domain only 

---

- Blood, 1986   **Biosynthesis of factor XIII B subunit by human hepatoma cell lines.**.
  The plasma transglutaminase, factor XIIIa FXIIIa , circulates as a zymogen containing two proteins, A and B, arranged in a noncovalent tetrameric complex, A2B2.
  Biosynthesis of plasma FXIII has not previously been demonstrated.
  In the present study, direct evidence has been obtained that two human hepatoma cell lines, Hep G2 and **PLC** PRF 5, synthesize and secrete FXIII B protein.
  Secretion of the B subunit of FXIII by Hep G2 was demonstrated by immunoblotting.
  De novo synthesis by Hep G2 was confirmed in 35S methionine labeled cultures.
  Radiolabeled conditioned medium was concentrated, mixed 1 1 with purified B protein, and examined by crossed immunoelectrophoresis with antiserum to the B subunit.
  The single protein precipitin **arc** of purified B protein comigrated with the radiolabeled FXIII from Hep G2 visualized by autoradiography, indicating both electrophoretic and antigenic identity.
  The data presented here represent the first demonstrations of biosynthesis of FXIII B protein by any cell type and suggest that the liver is the site of synthesis of FXIII B protein.
  Further analysis of concentrated Hep G2 serum free conditioned medium SFCM and cell lysate by immunoblotting following nondenaturing agarose gel electrophoresis demonstrated the FXIII A protein as well as the B protein and also revealed synthesis and secretion of the A and B proteins by **PLC** PRF 5.
  Crossed immunoelectrophoresis studies of Hep G2 SFCM and cell lysate suggest that Hep G2 cells also synthesize and secrete the plasma FXIII zymogen.
  With a specific radioimmunoassay for B protein, FXIII was found in Hep G2 SFCM at approximately 4 ng mL.
  with an amplified rocket immunoelectrophoresis technique the level was approximately 5 ng mL.
