# Supplementary material for: Content-rich biological network constructed by mining PubMed abstracts
Source: BMC Bioinformatics. 2004 Oct 8;5:147. doi: 10.1186/1471-2105-5-147 (PMC528731; doi:10.1186/1471-2105-5-147)
Supplement: Additional File 5 — The original Chilibot query results of the term "long-term potentiation (LTP)" and 22 other terms, limiting the latest references analyzed to the years 1990, 1995, 2000, and 2004. [file 1471-2105-5-147-S5.bz2 › chilibotAdditionalFile5/ltp1990/html/PLC_ACTIN.html]

 


 **PLC** and **ACTIN** 
  
Found 5 abstracts in PubMed,  **5 abstracts were retrieved and analyzed**.  


---

 Search Google  |
 PDF files only 
|  EDU domain only 

---

- Cell Regul, 1990   **The affinities of human platelet and Acanthamoeba profilin isoforms for polyphosphoinositides account for their relative abilities to inhibit phospholipase C.**.
  In light of recent work implicating profilin from human platelets as a possible regulator of both cytoskeletal dynamics and inositol phospholipid mediated signaling, we have further characterized the interaction of platelet profilin and the two isoforms of Acanthamoeba profilin with inositol phospholipids.
  Profilin from human platelets binds to phosphatidylinositol 4 monophosphate PIP and phosphatidylinositol bisphosphate PIP2 with relatively high affinity Kd approximately 1 microM for PIP2 by equilibrium gel filtration , but interacts only weakly if at all with phosphatidylinositol PI or inositol trisphosphate IP3 in small zone gel filtration assays.
  The two isoforms of Acanthamoeba profilin both have a lower affinity for PIP2 than does human platelet profilin, but the more basic profilin isoform from Acanthamoeba profilin II has a much higher approximately 10 microM Kd affinity than the acidic isoform profilin I, 100 to 500 microM Kd .
  None of the profilins bind to phosphatidylserine PS or phosphatidylcholine PC in small zone gel filtration experiments.
  The differences in affinity for PIP2 parallel the ability of these three profilins to inhibit PIP2 hydrolysis by soluble phospholipase C **PLC** .
  The results show that the interaction of profilins with PIP2 is specific with respect to both the lipid and the proteins.
  In Acanthamoeba, the two isoforms of profilin may have specialized functions on the basis of their identical approximately 10 microM affinities for **actin** monomers and different affinities for PIP2.

  - J Immunol, 1990   **Interactions between a lymphoma membrane associated guanosine 5 triphosphate binding protein and the cytoskeleton during receptor patching and capping.**.
    In this study we have used several complementary techniques to isolate and characterize a lymphoma membrane associated 41 kDa protein that shares a number of structural and functional similarities with the alpha i subunit of the guanosine 5 triphosphate GTP binding protein e.g., Gi alpha like protein .
    In addition, using permeabilized lymphoma cells, we have found that 1 GTP or GTP tau S augments, and pertussis toxin inhibits, phospholipase C **PLC** activity and receptor capping.
    and 2 the addition of lymphoma 41 kDa Gi alpha like protein stimulates **PLC** activity and receptor patching capping, and reverses the inhibitory effect of pertussis toxin on both activity and receptor patching capping.
    Additional cytochemical and biochemical data indicate that the lymphoma 41 kDa protein is closely associated with several cytoskeletal proteins e.g., **actin**, myosin, and fodrin all of which colocalize under receptor cap structures.
    Furthermore, both the 41 kDa mediated phospholipase C activity and receptor patching capping are inhibited by cytochalasin D a microfilament disrupting drug and W 7 drug a calmodulin inhibitor .
    Together, these data provide strong evidence for a functional association between the lymphoma membrane cytoskeleton and the 41 kDa Gi alpha like protein.
    Specifically, this association appears to be required for the activation of phospholipase C that results in inositol triphosphate production, subsequent internal Ca2ion release, and finally surface receptor patching and capping.

    - Arch Biochem Biophys, 1990   **Persistent activation of platelet membrane phospholipase C by proteolytic action of trypsin and thrombin.**.
      Trypsin causes rapid activation of intact platelets that mimics many actions of thrombin, including the stimulation of phospholipase C **PLC** .
      We have examined the effects of thrombin and trypsin on **PLC** in a platelet membrane preparation using exogenous 3H phosphatidylinositol bisphosphate PIP2 as substrate.
      Trypsin induced PIP2 breakdown, which was maximal at 20 micrograms ml, but was reduced at higher concentrations.
      alpha and gamma Thrombins also stimulated **PLC** induced hydrolysis of PIP2 in membranes.
      This effect was inhibited by leupeptin.
      Exogenous 3H phosphatidylinositol 4 monophosphate PIP was hydrolyzed in response to both thrombin and trypsin in the same ratio as PIP2.
      Activation of membrane bound **PLC** persisted after removal of thrombin and trypsin.
      The hydrolysis of 3H phosphatidylinositol was not activated by alpha thrombin and trypsin.
      We examined the question of whether calpain was involved in the observed **PLC** activation by thrombin and trypsin.
      Although dibucaine activated a Ca2ion dependent protease as judged by the hydrolysis of **actin** binding protein and by the activation of phosphoprotein phosphatases, it failed to stimulate the generation of phosphatidic acid in 32P prelabeled platelets.
      Moreover, when **PLC** was assayed in the membranes, the addition of Ca2ion activated neutral proteinases did not increase the rate of hydrolysis of either PIP or PIP2.
      Our results show that proteases such as trypsin and thrombin are able to stimulate membrane bound **PLC**, but this activation does not seem to be related to calpain.

      - Eur J Immunol, 1988   **Interleukin 6, the third mediator of acute phase reaction, modulates hepatic protein synthesis in human and mouse.
        Comparison with interleukin 1 beta and tumor necrosis factor alpha.**.
        Interleukin 6 IL6 is the new definition of a group of cytokines previously named according to their biological activity, e.g. B cell stimulatory factor 2 BSF 2 , hybridoma plasmocytoma growth factor HGF , interferon beta 2 IFN beta 2 , hepatocyte stimulating factor HSF .
        It has recently been suggested that IL6 may represent the major mediator of acute phase protein response whereas IL1 beta and TNF alpha could play a minor role.
        We compared the effect of the three cytokines on hepatic protein synthesis by performing in vitro as well as in vivo experiments.
        Human hepatoma cells **PLC** PRF5 were exposed to each cytokine separately for 20 h, and the effect was then studied at the protein and RNA level.
        All three cytokines reduced albumin and increased C3 and ceruloplasmin biosynthesis.
        The cytokines induced the same effect at the RNA level indicating that the modulation was pretranslational.
        The effect of the cytokines was specific since **actin** gene expression was not changed.
        furthermore the effect was blocked by specific antibodies against the cytokines.
        The effect of the single cytokines was dose and time dependent, and quantitatively comparable.
        None of the cytokines was able to alter alpha 1 anti trypsin synthesis.
        In vivo experiments with mice showed that IL1 beta and TNF alpha both induce serum amyloid A SAA mRNA in the mouse liver and increase factor B Bf gene expression.
        Human recombinant IL6 induced SAA gene expression and it also had a weak positive effect on Bf gene expression after i.p. injection.
        These data demonstrate that the three cytokines studied are quantitatively and qualitatively comparable,.
        all three are probably involved in acute phase protein response.

        - Eur J Clin Invest, 1988   **Alpha and gamma interferon IFN alpha, IFN gamma but not interleukin 1 IL 1 modulate synthesis and secretion of beta 2 microglobulin by hepatocytes.**.
          Soluble serum beta 2 microglobulin has been thought to result from membrane shedding by activated T lymphocytes.
          This hypothesis could explain the increase of beta 2 microglobulin serum levels during virally induced mononucleosis, but not elevated levels as observed in other virally induced and in malignant diseases.
          In this paper we demonstrate that beta 2 microglobulin is a true secretory protein,.
          its synthesis in hepatocytes is modulated by IFNs but not by IL 1.
          While the 4 00 MW HLA antigen can be found only in cell lysates, beta 2 microglobulin is shown to be secreted also into the culture medium like other secretory proteins e.g. albumin factor B complement C3 .
          Furthermore, interferon alpha IFN alpha as well as interferon gamma IFN gamma directly stimulate, in a dose and time dependent manner, beta 2 microglobulin synthesis by human hepatoma cells Mz Hep 1 and **PLC** PRF5 and murine hepatocyte primary cultures.
          The increase of beta 2 microglobulin production induced by interferons is demonstrated at both the protein and the RNA level, indicating that interferon acts at a pretranslational level.
          The interferon effect on beta 2 microglobulin synthesis is specific since synthesis of secretory proteins like complement C3 or albumin, and of a structural protein like **actin**, remains unchanged.
          In contrast to IFN, IL 1, the main mediator of acute phase response, does not change beta 2 M biosynthesis rate.
          These data indicate that i beta 2 microglobulin is a secretory protein, ii IFNs but not IL 1 can mediate increased beta 2 M serum levels, and iii the liver may be its primary source.
