# Supplementary material for: Content-rich biological network constructed by mining PubMed abstracts
Source: BMC Bioinformatics. 2004 Oct 8;5:147. doi: 10.1186/1471-2105-5-147 (PMC528731; doi:10.1186/1471-2105-5-147)
Supplement: Additional File 5 — The original Chilibot query results of the term "long-term potentiation (LTP)" and 22 other terms, limiting the latest references analyzed to the years 1990, 1995, 2000, and 2004. [file 1471-2105-5-147-S5.bz2 › chilibotAdditionalFile5/ltp1990/html/CREB_ATF.html]

 


 **CREB** and **ATF** 
  
Found 19 abstracts in PubMed,  **19 abstracts were retrieved and analyzed**.  


---

 Search Google  |
 PDF files only 
|  EDU domain only 

---

**Interactive relationship** (e.g. stimulation, inhibition, etc)

- A third such family known as the  **CREB**  or  **ATF**  proteins, bind to a sequence element present in promoters from a number of viral and cellular genes.  Ref: 2138276 Oncogene, 1990
- The peptide sequences 483 and 462 amino acids, respectively derived from each of these cDNAs are identical, except for the additional 21 amino acids in  **ATF**  a, but clearly differ from the other  **ATF**   **CREB**  proteins reported.  Ref: 1694576 Nucleic Acids Res, 1990
- **ATF**  43 polypeptides are related by their reactivity with anti  **ATF**  43, DNA binding specificity, complex formation with  **CREB** , heat stability, and phosphorylation by protein kinase A.  Ref: 2147221 Mol Cell Biol, 1990
- These binding domains may bind TATA region binding factors site I, the  **CREB**   **ATF**  protein site II, the AP 1 protein site III, and nuclear factor I CTF site IV.  Ref: 2975753 Mol Cell Biol, 1988
- Certain cell types vary in their  **ATF**  43 complement, suggesting that  **CREB**  activity is modulated in a cell type specific manner through interaction with  **ATF**  43.  Ref: 2147221 Mol Cell Biol, 1990
- The c jun and c fos proteins also bound to both the somatostatin CRE and E3  **ATF**  binding sites, but  **CREB**  did not bind to AP1 recognition sites nor was it capable of forming heterodimers with either c jun or c fos.  Ref: 1974651 J Virol, 1990
- The AP 1 activity binds efficiently to both AP 1 and activating transcription factor  **ATF**  cAMP response element binding protein  **CREB**  binding sites present in E1A inducible promoters and presumably plays a role in the transcriptional activation of adenovirus genes by E1A proteins and cAMP.  Ref: 2559873 Genes Dev, 1989
- Such a factor, termed  **ATF**  adenovirus transcription factor, has already been characterized and appears to have strong similarities to the transcriptional factor  **CREB**  cAMP responsive element binding protein, which binds homologous sequences in cAMP responsive genes, such as somatostatin and c fos.  Ref: 2902626 Proc Natl Acad Sci U S A, 1988
- We wished to characterize  **CREB** , c jun, and c fos binding to these sites in the somatostatin gene CRE and in the adenovirus early region 3 promoter E3  **ATF** .  Ref: 1974651 J Virol, 1990
- Truncations of the  **CREB**  protein, which eliminated regions of the protein containing consensus sites for phosphorylation by protein kinase A, protein kinase C, and casein kinase II, bound to both the CRE and  **ATF**  sites, indicating that these consensus sites were not essential for DNA binding or dimer formation.  Ref: 1974651 J Virol, 1990
- The cellular transcription factor  **CREB**  corresponds to activating transcription factor 47  **ATF**  47 and forms complexes with a group of polypeptides related to  **ATF**  43.  Ref: 2147221 Mol Cell Biol, 1990
- Among these factors, polypeptides termed activating transcription factor 43  **ATF**  43 and  **ATF**  47 have been purified from HeLa cells and a factor referred to as cyclic AMP response element binding protein  **CREB**  has been isolated from PC12 cells and rat brain.  Ref: 2147221 Mol Cell Biol, 1990

**Parallel relationship** (e.g. studied together, co-existance, homology, etc.)

- The CRE affinity purified 120 kDa protein displays properties distinct from those of the 43 kDa  **CREB**   **ATF**  polypeptide.  Ref: 2137455 J Biol Chem, 1990
- furthermore, gel retardation assays failed to reveal dramatic differences in the total amount of  **CREB**   **ATF** , AP 1, and NF 1 binding activity present in mock or poliovirus infected cell extracts.  Ref: 2166827 J Virol, 1990
- Loss of a phosphorylated form of transcription factor  **CREB**   **ATF**  in poliovirus infected cells.  Ref: 2166827 J Virol, 1990
- The ATGACGTCAT sequence strongly resembles sites bound by the yeast and mammalian  **ATF**   **CREB**  family of proteins, suggesting that GCN4 and the  **ATF**   **CREB**  proteins recognize similar half sites but have different spacing requirements.  Ref: 2204805 Mol Cell Biol, 1990
- We demonstrated that  **CREB**  and  **ATF**  47 are identical.  Ref: 2147221 Mol Cell Biol, 1990
- We found that a single  **CREB**   **ATF**  binding site resulted in a surprisingly strong promoter which responded to E1A.  Ref: 2545919 J Virol, 1989
- this induction was dependent upon the phosphorylation of  **CREB**   **ATF** .  Ref: 2166827 J Virol, 1990
- However, in vitro kinase reactions demonstrated that mock and poliovirus infected cell extracts contained similar levels of  **CREB**   **ATF** .  Ref: 2166827 J Virol, 1990
- These include binding sites for NF 1 site IV, AP1 site III,  **CREB**  activating transcription factor  **ATF**  site II, and TATA site I.  Ref: 2139139 J Virol, 1990
- The major promoter responds strongly to virus encoded trans activators EIA and EIV and contains four elements a TAGA motif analogous to the TATA box, two EIIF sites present in an inverted orientation, and an  **ATF**   **CREB**  site.  Ref: 2139891 J Virol, 1990
- A group of factors known as activating transcription factors  **ATF**  have been found to bind to the latter and related sequences found upstream of early adenovirus promoters induced by E1A, and these factors are highly homologous to the  **CREB**  protein.  Ref: 1974651 J Virol, 1990
- Mutations that define the optimal half site for binding yeast GCN4 activator protein and identify an  **ATF**   **CREB**  like repressor that recognizes similar DNA sites.  Ref: 2204805 Mol Cell Biol, 1990
- Deletion analysis indicated that the  **ATF**   **CREB**  site was crucial for E1A mediated stimulation.  Ref: 2142416 Arch Virol, 1990
- These results directly demonstrate that many different transcription factor binding sites, including the E1B TATA box, a  **CREB**   **ATF**  binding site, and two E2F sites, can mediate E1A transactivation.  Ref: 2545919 J Virol, 1989
- Thus, we propose that poliovirus infection inhibits transcription from the E3 promoter, at least in part, through the dephosphorylation of  **CREB**   **ATF** .  Ref: 2166827 J Virol, 1990
- Expression from the E3 promoter was shown to be activated by  **CREB**   **ATF**  in vivo.  Ref: 2166827 J Virol, 1990
- A similar folding transition is observed on GCN4 p binding to the related  **ATF**   **CREB**  site, which contains an additional central base pair.  Ref: 2145515 Nature, 1990
- A computer based search of this region of bovine CYP17 for consensus sequences associated with binding of transcription factorsi.e., GR, PR,  **CREB**   **ATF** , AP1, AP2, AP3, AP4, AP5, OTF, CTF NF1, SP1 shows only the consensus  **CREB**   **ATF**  sequence TGACGT which is also found to be at approximately the same position in the human CYP17 gene.  Ref: 2543297 Arch Biochem Biophys, 1989
- The sequence motif CGTCA is critical for binding of a group of cellular transcription factors  **ATF** ,  **CREB** , E4F, and EivF and for activation of certain E1a inducible and cyclic AMP cAMP inducible promoters.  Ref: 2555692 Mol Cell Biol, 1989
- These regions contain binding sites for transcription factors NF 1 site IV, AP 1 site III,  **CREB**   **ATF**  site II, and the TATA factor site I.  Ref: 2166827 J Virol, 1990
- We also found that the cis requirements for stable DNA binding by  **ATF**  43 and  **CREB**  are different.  Ref: 2147221 Mol Cell Biol, 1990
- Whether the consensus  **CREB**   **ATF**  sequence is associated with the cAMP mediated transcription of the CYP17 gene remains to be elucidated.  Ref: 2543297 Arch Biochem Biophys, 1989
- The  **ATF**   **CREB**  motif may be a target for stimulation of HCMV gene expression through either viral or cellular transcription factors.  Ref: 2142416 Arch Virol, 1990
- Unexpectedly, in the context of the his3 promoter, the ATGACGTCAT derivative reduced transcription below the basal level in a GCN4 independent manner, presumably reflecting DNA binding by a distinct  **ATF**   **CREB**  like repressor protein.  Ref: 2204805 Mol Cell Biol, 1990
- Radioimmunoprecipitation reactions performed with antiserum against  **CREB**   **ATF**  revealed a severe reduction in a phosphorylated form of the protein present in poliovirus infected cell extracts.  Ref: 2166827 J Virol, 1990
- Gel retardation assays did reveal significant qualitative differences in the DNA protein complexes formed with a  **CREB**   **ATF**  binding site in extracts prepared from poliovirus infected cells as compared to mock infected cell extracts.  Ref: 2166827 J Virol, 1990
- Adenovirus early region 3 promoter regulation by E1A E1B is independent of alterations in DNA binding and gene activation of  **CREB**   **ATF**  and AP1.  Ref: 2139139 J Virol, 1990
- **CREB**  and  **ATF**  43 form protein protein complexes.  Ref: 2147221 Mol Cell Biol, 1990
- Insertion of a synthetic oligonucleotide homologous to a 19 bp motif and containing an  **ATF**   **CREB**  binding site into an HCMV chimera lacking  **ATF**   **CREB**  motifs conferred E1A responsivity on HCMV promoter mediated CAT expression whereas insertion of a similar oligonucleotide containing a change of two bases in the sequence of the  **ATF**   **CREB**  site did not.  Ref: 2142416 Arch Virol, 1990
- However, neither domain B alone, nor  **ATF**   **CREB**  binding sites respond significantly to Tax1.  Ref: 2311587 EMBO J, 1990
- The 19 bp motif contains a potential binding site for the cellular transcription factor  **ATF**   **CREB** .  Ref: 2142416 Arch Virol, 1990
- These data suggest that cyclic AMP response element binding protein  **ATF**   **CREB**  or related proteins activate V beta transcription.  Ref: 2557542 Mol Cell Biol, 1989
- A  **CREB**   **ATF**  binding site placed upstream of the E1B TATA box behaved much like the wild type E1B promoter, which is composed of a single Sp1 binding site plus a TATA box.  Ref: 2545919 J Virol, 1989
