# Supplementary material for: Content-rich biological network constructed by mining PubMed abstracts
Source: BMC Bioinformatics. 2004 Oct 8;5:147. doi: 10.1186/1471-2105-5-147 (PMC528731; doi:10.1186/1471-2105-5-147)
Supplement: Additional File 5 — The original Chilibot query results of the term "long-term potentiation (LTP)" and 22 other terms, limiting the latest references analyzed to the years 1990, 1995, 2000, and 2004. [file 1471-2105-5-147-S5.bz2 › chilibotAdditionalFile5/ltp1990/html/PKC_PKA.html]

 


 **PKC** and **PKA** 
  
Found 40 abstracts in PubMed,  **30 abstracts were retrieved and analyzed**.  


---

 Search Google  |
 PDF files only 
|  EDU domain only 

---

**Interactive relationship** (e.g. stimulation, inhibition, etc)

- Tamoxifen inhibited  **PKC**  activity IC50 = 80 microM but increased  **PKA**  dependent protein phosphorylation.  Ref: 2252838 Cell Signal, 1990
- In LLC PK1 porcine epithelial cells, the urokinase type plasminogen activator u PA mRNA and protein can be induced either by stimulation of the protein kinase C  **PKC**  pathway using a tumor promoter PMA or by stimulation of the protein kinase A  **PKA**  pathway with calcitonin SCT.  Ref: 2583867 Int J Cancer, 1989
- In contrast, PTH activation of cAMP dependent protein kinase  **PKA** , while also rapid, was greater in magnitude 0.10 to 0.50, persistent, and occurred at a threshold level of 3 x 10 10 M PTH, compared to 10 8 M for  **PKC** .  Ref: 2930565 Biochem Biophys Res Commun, 1989
- The changes in  **PKA**  and  **PKC**  activities greatly modified the  **PKC**   **PKA**  ratios in the cytosols and the particulate fractions of cultured cells.  Ref: 2170212 Mol Cell Endocrinol, 1990
- The role of tyrosine protein kinases in connexon gating may involve interaction with the  **pkA**  and  **pkC**  regulatory cascades.  Ref: 2194784 Endocr Rev, 1990
- The subunit specificity for  **PKC**  is different from that observed for cAMP dependent protein kinase  **PKA** .  Ref: 2397211 Biochemistry, 1990
- In contrast, PDBu depleted quiescent DMBA31 cells of  **PKC**  activity only and had no effect on the  **PKA**  activity.  Ref: 2171757 Cancer Res, 1990
- **pKc**  = 10.12 and pKd = 9.88 are in good concordance with those obtained from the conventional regression linear method  **pKa**  = 9.45.  Ref: 2570856 J Pharm Pharmacol, 1989
- **PKC**  activity was quantitated by measuring PDBu specific phosphorylation of a 4 00 molecular weight protein, and  **PKA**  activity monitored by measuring prostacyclin dependent phosphorylation of a 2 00 molecular weight protein.  Ref: 2252838 Cell Signal, 1990
- A hybrid  **PKC**  gene, PKAC, was constructed by substituting the coding region for the N terminal 253 amino acids of  **PKC**  alpha with the N terminal 17 amino acids of the cyclic AMP dependent protein kinase catalytic subunit  **PKA** .  Ref: 2785241 Mol Cell Biol, 1989
- In contrast to the in vitro situation where staurosporine inhibited  **PKC**  activity, in the cell culture system the microbial agent caused an early translocation of  **PKC**  and inhibited  **PKA** .  Ref: 2583867 Int J Cancer, 1989
- Protein phosphorylation mediated by  **PKA**  inhibits the Na H exchanger while that mediated by  **PKC**  stimulates activity.  Ref: 2554050 Kidney Int, 1989

**Parallel relationship** (e.g. studied together, co-existance, homology, etc.)

- As in these typical TNF producer cells, the production of TNF is also controlled by  **PKA**  and  **PKC** , a regulatory circuit is proposed, by which these two independent signal pathways antagonistically regulate TNF production and, at the receptor level, TNF sensitivity.  Ref: 2549168 J Exp Med, 1989
- Furthermore, when nuclei from unstimulated cells were incubated with  **PKA**  or  **PKC**  treated cytosolic fraction for 30 min at 30 degrees C, NF kappa B was translocated into the nuclei.  Ref: 2548081 Mol Cell Biol, 1989
- Neither bPTH 3 34 nor bPTH 7 34 activated either protein kinase, while both antagonized rPTH 1 34 induced  **PKC**  translocation more effectively than  **PKA**  activation.  Ref: 2930565 Biochem Biophys Res Commun, 1989
- The observations suggest that c fos protein is involved in  **PKC**  and  **PKA**  signal transduction in cultured human glial cells.  Ref: 2127926 Histochemistry, 1990
- Proton and calcium transporting systems are mainly activated by serine threonine protein kinases such as  **pkA**  and  **pkC** .  Ref: 2194784 Endocr Rev, 1990
- Protein kinase C  **PKC**  and cyclic AMP dependent protein kinase  **PKA**  were activated selectively by treating platelets with phorbol dibutyrate PDBu or prostacyclin PGl2.  Ref: 2252838 Cell Signal, 1990
- In quiescent A31 cells, phorbol dibutyrate PDBu caused a 10 fold reduction in  **PKC**  activity and a 5 fold reduction in  **PKA**  activity.  Ref: 2171757 Cancer Res, 1990
- Phorbol ester binding activity was absent in both constructs but was preserved in another hybrid gene, PKCA, which was composed of the coding region for 1 to 253 amino acids of  **PKC**  alpha at the N terminal side and the coding region for 18 to 350 amino acids of  **PKA**  at the C terminal side.  Ref: 2785241 Mol Cell Biol, 1989
- cAMP dependent protein kinase  **PKA**  and phospholipid dependent protein kinase  **PKC**  play a role in nerve growth factor NGF mediated differentiation.  Ref: 1690563 Neuron, 1990
- As activation of  **PKA**  does not slow down the degradation rate of TNF Rs, but rather enhances protein synthesis dependent reexpression of TNF Rs after transient  **PKC**  mediated transmodulation and after tryptic digestion of TNF Rs, it is concluded that  **PKA**  stimulates TNF R synthesis.  Ref: 2549168 J Exp Med, 1989
- Thus, NGF increases Na channel number in PC12 cells in part by activating  **PKA**  but apparently not  **PKC** .  Ref: 1690563 Neuron, 1990
- UCN 01 has been shown to inhibit  **PKC**  and protein kinase A  **PKA**  with IC50 values of 0.0041 and 0.042 microM, respectively, and UCN 02 has been shown to inhibit  **PKC**  and  **PKA**  with IC50 values of 0.062 and 0.25 microM, respectively.  Ref: 2656615 J Antibiot (Tokyo), 1989
- The morphological and functional characteristics and the activities of cyclic AMP  **PKA**  I and  **PKA**  II and calcium and phospholipid dependent  **PKC**  protein kinases were studied in 2 day old suspension cultures of porcine thyroid cells and were compared with those in freshly dissociated cells and intact glands.  Ref: 2170212 Mol Cell Endocrinol, 1990
- Both secretagogues stimulated beta EP release within 5 min and therefore both  **PKA**  and  **PKC**  are potential mediators of the acute phase of hormonal stimulation of the corticotrope.  Ref: 2325905 Neurosci Lett, 1990
- The diverse pattern of phosphorylation of AChR by  **PKA**  and  **PKC**  may play a role in the regulation of its function.  Ref: 2397211 Biochemistry, 1990
- Here we show that the sites of phosphorylation by four kinases  **PKA** ,  **PKC** , CK and CaMK all lie in the C terminal microtubule binding half of tau, but only the phosphorylation by CaM kinase shows the pronounced shift in electrophoretic mobility characteristic for tau from Alzheimer neurofibrillary tangles.  Ref: 2120043 EMBO J, 1990
- Basically, this centers around the most consistent findings namely, that activation of  **pkA**  has an enhancing effect on cell communication while activation of  **pkC**  decreases that process.  Ref: 2194784 Endocr Rev, 1990
- Protein kinase C  **PKC**  and cyclic AMP dependent protein kinase  **PKA**  are important for normal cell proliferation.  Ref: 2171757 Cancer Res, 1990
- To determine the target for the protein kinases we purified and characterized both NF kappa B and I kappa B and found that I kappa B is phosphorylated and inactivated in the presence of  **PKC**  and HRI but not  **PKA** .  Ref: 2157987 Nature, 1990
- They further suggest that the activity of  **PKC**  and  **PKA**  may be coordinately regulated in nontumorigenic cells.  Ref: 2171757 Cancer Res, 1990
- In contrast, substitution of Ser 16 17 for threonine, which preserved phosphorylation in unstimulated cells but not  **PKA**  and  **PKC**  enhanced phosphorylation, resulted in a partially active gag v erbA protein.  Ref: 1979040 Genes Dev, 1990
- We thus assume that in LLC PK1 cells the  **PKA**  and  **PKC**  signal transferring pathways can function independently.  Ref: 2583867 Int J Cancer, 1989
- DNA binding activity of NF kappa B was induced in the cytosolic fraction of unstimulated 70Z 3 murine pre B cells by incubation with the catalytic subunit of cyclic AMP dependent protein kinase  **PKA**  or protein kinase C  **PKC** .  Ref: 2548081 Mol Cell Biol, 1989
- Phosphorylation by calmodulin protein kinase II CaM PK II, myosin light chain kinase MLCK, cAMP dependent kinase  **PKA**  and protein kinase C  **PKC**  was monitored under optimal conditions for each enzyme.  Ref: 2737166 Endocrinology, 1989
- The v erbA gene product, a 75 kD gag v erbA fusion protein, is phosphorylated on Ser 16 17 of its v erbA encoded domain, and phosphorylation at this site is increased in vivo after activation of either the  **PKA**  or  **PKC**  signal transduction pathways.  Ref: 1979040 Genes Dev, 1990
- The effect of  **PKA**  and  **PKC**  on the Na H exchanger in native membranes and in solubilized brush border membrane proteins appears to be consistent with most of the published observations in intact cells.  Ref: 2554050 Kidney Int, 1989
- Incubations with phorbol dibutyrate, 8 Br cAMP and sodium nitroprusside representing signal transduction pathways of  **PKC** ,  **PKA**  and cyclic GMP kinase, respectively, were carried out for 60 and 120 min.  Ref: 2127926 Histochemistry, 1990
- In contrast,  **PKA**  and  **PKC**  did not activate NF kappa B in nuclear extracts from unstimulated cells.  Ref: 2548081 Mol Cell Biol, 1989
- Whereas  **PKC**  phosphorylates predominantly the delta subunit and the phosphorylation of the gamma subunit by this enzyme is very low,  **PKA**  phosphorylates both subunits to a similar high extent.  Ref: 2397211 Biochemistry, 1990
- **PKC**  inhibitor, H7, also blocked effectively the PMA plus dbcAMP induced IL 1 beta production, while the protein kinase A  **PKA**  inhibitor, HA1004, had no effect, suggesting that  **PKA**  activation is not involved in the mechanism of action of cAMP in this case.  Ref: 2175219 Blood, 1990
- In the presence of 10 5 M forskolin, an additional application of 10 8 M 12 O tetradecanoylphorbol 13 acetate, an activator of protein kinase C  **PKC** , produced a further increase in IK, suggesting that the active sites of  **PKA**  and  **PKC**  on the IK channel are different.  Ref: 2161457 J Physiol, 1990
- Activation of the signal transduction pathways mediated by protein kinase A  **PKA**  or protein kinase C  **PKC**  led to different responses of several serum inducible genes including the jun gene family, c fos, c myc, krox 20 and krox 24.  Ref: 2562123 New Biol, 1989
- We have employed an isolated ovine anterior pituitary cell superfusion system to determine the dynamic effects of forskolin, a protein kinase A  **PKA**  stimulator, and phorbol 12 myristate 13 acetate PMA, a protein kinase C  **PKC**  activator.  Ref: 2325905 Neurosci Lett, 1990
- Therefore in the IMCD cell activation of  **PKC**  but not  **PKA**  stimulates a rise in pHi via the Na H exchanger.  Ref: 2171360 Am J Physiol, 1990
- Our findings support the conclusion that NF kappa B exists in the cytoplasm of unstimulated cells in an inactive form that can be converted by exposure to  **PKA**  or  **PKC**  to an active DNA binding form that can translocate to the nucleus.  Ref: 2548081 Mol Cell Biol, 1989
- Using gel retardation assays we found that  **PKC** , cyclic AMP dependent protein kinase  **PKA**  and a haem regulated eIF 2 kinase HRI could activate NF kappa B in vitro.  Ref: 2157987 Nature, 1990
- These results suggest that induction of phosphorylation of P65 and P74 by TNF and IL 1 is not mediated by  **PKC**  and  **PKA**  but may be mediated by another protein kinase and result in overlapping of biological activities between TNF and IL 1.  Ref: 1966546 Cytokine, 1990
- Conversion of Ser 16 17 into alanine, although not affecting nuclear localization or DNA binding of the gag erbA protein, prevented phosphorylation of the v erbA encoded domain of the protein both in unstimulated cells or after stimulation by  **PKA**  and  **PKC**  activators.  Ref: 1979040 Genes Dev, 1990
- Thus, tyrosine protein kinases may invoke  **pkA**  and  **pkC**  pathways.  Ref: 2194784 Endocr Rev, 1990
- At the apical membrane, the Cl channel is regulated by phosphorylation with  **PKA**  and  **PKC** .  Ref: 2158763 Annu Rev Physiol, 1990
- Both retro inverso peptides are highly specific for  **PKC**  versus adenosine cAMP dependent protein kinase  **PKA**  and are totally stable towards proteolysis by trypsin or pronase.  Ref: 2514686 Biochem Biophys Res Commun, 1989
- The relationship between postulated changes in the  **PKC**  pathway and those hypothesized for the  **PKA**  pathway are discussed.  Ref: 2401213 Development, 1990
- This synthetic peptide is phosphorylated by both  **PKA**  and  **PKC** , suggesting that in the intact receptor both kinases may phosphorylate the gamma subunit at a similar site, as has been previously demonstrated by us for the delta subunit Safran, A.  Ref: 2397211 Biochemistry, 1990
- We conclude that beta adrenergic modulation of IK is mediated by cyclic AMP dependent phosphorylation but not by an increase in calcium i, that  **PKA**  and  **PKC**  enhance IK independently,.  Ref: 2161457 J Physiol, 1990
