# Supplementary material for: Content-rich biological network constructed by mining PubMed abstracts
Source: BMC Bioinformatics. 2004 Oct 8;5:147. doi: 10.1186/1471-2105-5-147 (PMC528731; doi:10.1186/1471-2105-5-147)
Supplement: Additional File 5 — The original Chilibot query results of the term "long-term potentiation (LTP)" and 22 other terms, limiting the latest references analyzed to the years 1990, 1995, 2000, and 2004. [file 1471-2105-5-147-S5.bz2 › chilibotAdditionalFile5/ltp1990/html/PKC_NMDA.html]

 


 **PKC** and **NMDA** 
  
Found 5 abstracts in PubMed,  **5 abstracts were retrieved and analyzed**.  


---

 Search Google  |
 PDF files only 
|  EDU domain only 

---

**Interactive relationship** (e.g. stimulation, inhibition, etc)

- To determine if  **PKC**  activation is a consequence of  **NMDA**  receptor activation during LTP, we applied the  **NMDA**  receptor antagonist drug, DL aminophosphonovalerate APV both immediately prior to and following high frequency stimulation, resulting in successful and unsuccessful blockade of LTP initiation, respectively.  Ref: 2905192 Brain Res, 1988
- Stimulation of N methyl DL aspartase  **NMDA**  receptors induces an influx of calcium, which is needed for HLTP maintenance, as are the activation of protein kinase C  **PKC**  and the synthesis of new proteins, for example calmodulin.  Ref: 1964542 Ann Med Interne (Paris), 1990

- :-)

  **Parallel relationship** (e.g. studied together, co-existance, homology, etc.)

  - These data provide the first evidence linking two mechanisms associated with LTP,  **NMDA**  receptor activation and  **PKC**  substrate phosphorylation.  Ref: 2905192 Brain Res, 1988
