# Supplementary material for: Content-rich biological network constructed by mining PubMed abstracts
Source: BMC Bioinformatics. 2004 Oct 8;5:147. doi: 10.1186/1471-2105-5-147 (PMC528731; doi:10.1186/1471-2105-5-147)
Supplement: Additional File 5 — The original Chilibot query results of the term "long-term potentiation (LTP)" and 22 other terms, limiting the latest references analyzed to the years 1990, 1995, 2000, and 2004. [file 1471-2105-5-147-S5.bz2 › chilibotAdditionalFile5/ltp1990/html/PKC_TAU.html]

 


 **PKC** and **TAU** 
  
Found 3 abstracts in PubMed,  **3 abstracts were retrieved and analyzed**.  


---

 Search Google  |
 PDF files only 
|  EDU domain only 

---

**Interactive relationship** (e.g. stimulation, inhibition, etc)

- These results prove that the type I, II, and III  **PKC**  are products of  **PKC**  genes,  **tau** , beta, and alpha, respectively.  Ref: 3426619 Biochem Biophys Res Commun, 1987
- Immunoblot analysis revealed that the expressed  **PKC**  in COS cells transfected with either alpha, beta, or  **tau**  cDNA of  **PKC**  were recognized by specific antibodies against the type III, II, and I  **PKC**  isozymes, respectively.  Ref: 3426619 Biochem Biophys Res Commun, 1987
- Here we show that the sites of phosphorylation by four kinases PKA,  **PKC** , CK and CaMK all lie in the C terminal microtubule binding half of  **tau** , but only the phosphorylation by CaM kinase shows the pronounced shift in electrophoretic mobility characteristic for  **tau**  from Alzheimer neurofibrillary tangles.  Ref: 2120043 EMBO J, 1990

**Parallel relationship** (e.g. studied together, co-existance, homology, etc.)

- Immunocytochemical studies of rat cerebellum using specific antibodies against type I, II, and III  **PKC**  revealed the presence of the type I  **PKC**  in the Purkinje cells where transcripts of  **tau**  cDNA were localized, the type II  **PKC**  in the granule cells where transcripts of beta cDNA were detected, and the type III  **PKC**  in both the Purkinje and granule cells.  Ref: 3426619 Biochem Biophys Res Commun, 1987
