# Supplementary material for: Content-rich biological network constructed by mining PubMed abstracts
Source: BMC Bioinformatics. 2004 Oct 8;5:147. doi: 10.1186/1471-2105-5-147 (PMC528731; doi:10.1186/1471-2105-5-147)
Supplement: Additional File 5 — The original Chilibot query results of the term "long-term potentiation (LTP)" and 22 other terms, limiting the latest references analyzed to the years 1990, 1995, 2000, and 2004. [file 1471-2105-5-147-S5.bz2 › chilibotAdditionalFile5/ltp1990/html/PKC_CAMKII.html]

 


 **PKC** and **CAMKII** 
  
Found 3 abstracts in PubMed,  **3 abstracts were retrieved and analyzed**.  


---

 Search Google  |
 PDF files only 
|  EDU domain only 

---

**Interactive relationship** (e.g. stimulation, inhibition, etc)

**Parallel relationship** (e.g. studied together, co-existance, homology, etc.)

- Inhibition of postsynaptic  **PKC**  or  **CaMKII**  blocks induction but not expression of LTP.  Ref: 2549638 Science, 1989
- Synthetic peptides corresponding to the autoinhibitory domains of calcium calmodulin dependent protein kinase II  [ **CAMKII** ]  CaMK 281 309, smooth muscle myosin light chain kinase MLCK 480 501, and protein kinase C  **PKC**  19 36 as well as a peptide derived from the heat stable inhibitor of cAMP dependent protein kinase PKI tide were tested for their inhibitory specificities.  Ref: 2153665 J Biol Chem, 1990
- Thus both postsynaptic  **PKC**  and  **CaMKII**  are required for the induction of LTP and a presynaptic protein kinase appears to be necessary for the expression of LTP.  Ref: 2549638 Science, 1989
- Induction of LTP is blocked by intracellular delivery of H 7, a general protein kinase inhibitor, or  **PKC**  19 31, a selective protein kinase C  **PKC**  inhibitor, or  **CaMKII**  273 302, a selective inhibitor of the multifunctional calcium calmodulin dependent protein kinase  **CaMKII** .  Ref: 2549638 Science, 1989
- Involvement of protein phosphorylation in LTP has been widely proposed, with protein kinase C  **PKC**  and calcium calmodulin kinase type II  **CaMKII**  as leading candidates.  Ref: 2847049 Nature, 1988
