# Supplementary material for: Content-rich biological network constructed by mining PubMed abstracts
Source: BMC Bioinformatics. 2004 Oct 8;5:147. doi: 10.1186/1471-2105-5-147 (PMC528731; doi:10.1186/1471-2105-5-147)
Supplement: Additional File 5 — The original Chilibot query results of the term "long-term potentiation (LTP)" and 22 other terms, limiting the latest references analyzed to the years 1990, 1995, 2000, and 2004. [file 1471-2105-5-147-S5.bz2 › chilibotAdditionalFile5/ltp1990/html/PKC_ACTIN.html]

 


 **PKC** and **ACTIN** 
  
Found 14 abstracts in PubMed,  **14 abstracts were retrieved and analyzed**.  


---

 Search Google  |
 PDF files only 
|  EDU domain only 

---

**Interactive relationship** (e.g. stimulation, inhibition, etc)

- The protein kinase C  **PKC**  inhibitor staurosporine was found to dramatically alter the  **actin**  microfilament cytoskeleton of a variety of cultured cells, including PTK2 epithelial cells, Swiss 3T3 fibroblasts, and human foreskin fibroblasts.  Ref: 2185942 Exp Cell Res, 1990
- Introduction of calcium into platelets by the use of the ionophore A23187 or stimulation of protein kinase C  **PkC**  by the phorbol ester TPA did not induce  **actin**  polymerization.  Ref: 2826193 Exp Cell Res, 1988
- The  **actin**  filament disruption induced by staurosporine was distinguishable from the  **actin**  reorganization induced by exposure to the tumor promoter and activator of  **PKC**  phorbol myristate acetate PMA.  Ref: 2185942 Exp Cell Res, 1990
- Swiss 3T3 cells made deficient in  **PKC**  by prolonged exposure to PMA  **PKC**  down regulation exhibited  **actin**  alterations in response to staurosporine which were comparable to those in cells which had not been exposed to the phorbol ester.  Ref: 2185942 Exp Cell Res, 1990

**Parallel relationship** (e.g. studied together, co-existance, homology, etc.)

- However, unlike calcium which controls the binding of  **PKC**  to the lipid component on cell membranes, zinc controls the distribution of  **PKC**  to membrane cytoskeleton, possibly  **actin** .  Ref: 2226843 FEBS Lett, 1990
- Phosphorylation of band 4.1 by  **PKC**  in vitro results in a dramatic reduction in band 4.1 binding to spectrin and  **actin** , as well as to the cytoplasmic domain of band 3.  Ref: 2583288 FEBS Lett, 1989
- While the exact mechanism of staurosporine induced  **actin**  reorganization remains to be determined, the observed effects of staurosporine on  **PKC**  deficient cells make a role for  **PKC**  unlikely.  Ref: 2185942 Exp Cell Res, 1990
- Four calcium and phospholipid binding proteins purified from mononuclear cells were characterized for  **PKC**  and EGF phosphorylation,  **actin**  binding capacity, and partial tissue distribution.  Ref: 2550491 J Cell Biochem, 1989
- In a parallel control experiment, the  **actin**  cytoskeleton of  **PKC**  deficient 3T3 cells was unaffected in response to PMA, consistent with down regulation of this kinase.  Ref: 2185942 Exp Cell Res, 1990
- These studies may therefore establish a link between  **PKC**  activation and the induction of specific cellular proteins, such as  **actin**  and gelsolin, as important monocyte maturation products without which the cells will be restricted from acquiring certain macrophage activities.  Ref: 1963774 Biochem Int, 1990
- The finding that H 7 can elicit shape changes,  **actin**  polymerization and pinocytosis suggests that these events can occur without activation of protein kinase C  **PKC** .  Ref: 1695636 J Cell Sci, 1990
- Retinoic acid which inhibits  **PkC**  was also without effect on thrombin induced  **actin**  polymerization.  Ref: 2826193 Exp Cell Res, 1988
- These findings suggest that SF induced epithelial mobility may be mediated, in part, by protein synthesis, alterations in protein phosphorylation ? inhibition of  **PKC** , and  **actin**  filament reorganization.  Ref: 1704377 J Cell Sci, 1990
