# Supplementary material for: Content-rich biological network constructed by mining PubMed abstracts
Source: BMC Bioinformatics. 2004 Oct 8;5:147. doi: 10.1186/1471-2105-5-147 (PMC528731; doi:10.1186/1471-2105-5-147)
Supplement: Additional File 5 — The original Chilibot query results of the term "long-term potentiation (LTP)" and 22 other terms, limiting the latest references analyzed to the years 1990, 1995, 2000, and 2004. [file 1471-2105-5-147-S5.bz2 › chilibotAdditionalFile5/ltp1990/html/PKA_NMDA.html]

 


 **PKA** and **NMDA** 
  
Found 1 abstracts in PubMed,  **1 abstracts were retrieved and analyzed**.  


---

 Search Google  |
 PDF files only 
|  EDU domain only 

---

- J Med Chem, 1990   **A unified approach to systematic isosteric substitution for acidic groups and application to **NMDA** antagonists related to 2 amino 7 phosphonoheptanoate.**.
  A systematic approach to the replacement of acidic groups with potential bioisosteres is described.
  The strategy involves simple nucleophilic displacement of a common alkyl halide precursor with a variety of mercaptoazoles and related molecules.
  The mercaptoazoles and their oxidized derivatives sulfinyl and sulfonylazoles represent a series of possible surrogates for acidic groups which span a **pKa** range from about 4.5 11.5.
  This simple strategy was extended to include 2 hydroxy or 2 aminothiophenyl groups which function as relatively nonacidic isosteres for a phosphonic acid.
  By replacing the phosphonic acid of 2 amino 7 phosphonoheptanoate AP 7 with these groups, we have synthesized novel N methyl d aspartate **NMDA** antagonists.
