# Supplementary material for: Content-rich biological network constructed by mining PubMed abstracts
Source: BMC Bioinformatics. 2004 Oct 8;5:147. doi: 10.1186/1471-2105-5-147 (PMC528731; doi:10.1186/1471-2105-5-147)
Supplement: Additional File 5 — The original Chilibot query results of the term "long-term potentiation (LTP)" and 22 other terms, limiting the latest references analyzed to the years 1990, 1995, 2000, and 2004. [file 1471-2105-5-147-S5.bz2 › chilibotAdditionalFile5/ltp1990/html/PKA_TAU.html]

 


 **PKA** and **TAU** 
  
Found 15 abstracts in PubMed,  **15 abstracts were retrieved and analyzed**.  


---

 Search Google  |
 PDF files only 
|  EDU domain only 

---

**Interactive relationship** (e.g. stimulation, inhibition, etc)

- Here we show that the sites of phosphorylation by four kinases  **PKA** , PKC, CK and CaMK all lie in the C terminal microtubule binding half of  **tau** , but only the phosphorylation by CaM kinase shows the pronounced shift in electrophoretic mobility characteristic for  **tau**  from Alzheimer neurofibrillary tangles.  Ref: 2120043 EMBO J, 1990
