# Supplementary material for: Content-rich biological network constructed by mining PubMed abstracts
Source: BMC Bioinformatics. 2004 Oct 8;5:147. doi: 10.1186/1471-2105-5-147 (PMC528731; doi:10.1186/1471-2105-5-147)
Supplement: Additional File 5 — The original Chilibot query results of the term "long-term potentiation (LTP)" and 22 other terms, limiting the latest references analyzed to the years 1990, 1995, 2000, and 2004. [file 1471-2105-5-147-S5.bz2 › chilibotAdditionalFile5/ltp1990/html/PKA_ACTIN.html]

 


 **PKA** and **ACTIN** 
  
Found 1 abstracts in PubMed,  **1 abstracts were retrieved and analyzed**.  


---

 Search Google  |
 PDF files only 
|  EDU domain only 

---

- Biochim Biophys Acta, 1983   **The solubilization of cytoskeletons of human erythrocyte membranes by p mercuribenzene sulphonate.**.
  The disruption of erythrocyte membrane cytoskeletons brought about by treatment with p mercuribenzene sulphonate PMBS has been followed by measurements of turbidity and the binding of 203Hg labelled PMBS.
  After pretreatment with N ethylmaleimide to block readily reactive sulphydryl groups, incubation with 203Hg PMBS showed incorporation of approximately 4 moles radiolabel per mole of spectrin and one per mole of **actin**.
  The incorporation of radiolabel paralleled the decrease in turbidity, and the labelling of spectrin paralleled that of **actin**.
  The kinetics were pseudo first order, and the pH dependence of the observed rate constant indicated a normal **pKa** value for the sulphydryl group involved.
  The calculated second order rate constant for the reaction of the sulphydryl anion with PMBS was several orders of magnitude less than expected from model compound studies.
  The results suggest that association between spectrin and **actin** may result in the steric hindrance of reactivity of a limited number of sulphydryl groups in each protein.
  Disruption of the spectrin **actin** association may then be linked to the modification of the sulphydryl groups.
