# Supplementary material for: Content-rich biological network constructed by mining PubMed abstracts
Source: BMC Bioinformatics. 2004 Oct 8;5:147. doi: 10.1186/1471-2105-5-147 (PMC528731; doi:10.1186/1471-2105-5-147)
Supplement: Additional File 5 — The original Chilibot query results of the term "long-term potentiation (LTP)" and 22 other terms, limiting the latest references analyzed to the years 1990, 1995, 2000, and 2004. [file 1471-2105-5-147-S5.bz2 › chilibotAdditionalFile5/ltp1990/html/NMDA_AMPA.html]

 


 **NMDA** and **AMPA** 
  
Found 105 abstracts in PubMed,  **30 abstracts were retrieved and analyzed**.  


---

 Search Google  |
 PDF files only 
|  EDU domain only 

---

**Interactive relationship** (e.g. stimulation, inhibition, etc)

- **NMDA**  antagonists did not protect against  **AMPA**  or kainate induced convulsions.  Ref: 2171944 Eur J Pharmacol, 1990
- At physiological concentrations of magnesium 1.2 mM,  **AMPA**  was found to potentiate  **NMDA**  induced release of 3H arachidonic acid.  Ref: 1982526 Brain Res Dev Brain Res, 1990
- **NMDA**  and kainic acid receptors have a complementary distribution to  **AMPA**  receptors in the human cerebellum.  Ref: 2178036 Brain Res, 1990
- The relative potency as  **AMPA**  receptor agonists of  **AMPA** , N methyl  **AMPA** , N, N dimethyl  **AMPA**  and RS 3 hydroxy, tetrahydroisoxazolo c pyridine 5 carboxylic acid 5 HPCA, a bicyclic analogue of  **AMPA** , was distinctly different from that of the AMAA series of compounds as  **NMDA**  agonists.  Ref: 1963602 Eur J Pharmacol, 1990
- The two heterocyclic aspartic acid and glutamic acid analogues derived from ibotenic acid, RS 2 amino 2 3 hydroxy 5 methylisoxazol 4 yl acetic acid AMAA and RS 2 amino 3 3 hydroxy 5 methylisoxazol 4 yl propionic acid  **AMPA**  have previously been shown to be selective agonists at N methyl D aspartic acid  **NMDA**  and  **AMPA**  receptors, respectively.  Ref: 1963602 Eur J Pharmacol, 1990
- alpha Amino 3 hydroxy 5 methyl 4 isoxazole propionic acid  **AMPA**  was the most potent convulsant ED50 0.6 mg kg, followed by kainate ED50 1.5 mg kg, N methyl D aspartate  **NMDA**  ED50 3.1 mg kg, then quisqualate ED50 5.1 mg kg.  Ref: 2171944 Eur J Pharmacol, 1990
- Dinitroquinoxaline dione DNQX selectively prevented the effect of  **AMPA**  at doses which had no effect on  **NMDA**  or kainate convulsions.  Ref: 2171944 Eur J Pharmacol, 1990

**Parallel relationship** (e.g. studied together, co-existance, homology, etc.)

- Using neonatal rats three pharmacologically distinct excitatory amino acid receptor effects were demonstrated following administration of  **NMDA** ,  **AMPA**  or kainate.  Ref: 2171944 Eur J Pharmacol, 1990
- Most attention has been directed to synapses using  **NMDA**  receptors, although more recent evidence indicates potential roles for the  **AMPA**  receptors as well.  Ref: 2168104 Trends Pharmacol Sci, 1990
- Since glutamate activates both N methyl D aspartate  **NMDA**  and non  **NMDA**  receptors in rat dorsal horn neurons, selective agonists, kainate, quisqualate, alpha amino 3 hydroxy 5 methyl 4 isoxazole propionic acid  **AMPA**  and  **NMDA**  were used to determine which subtype of excitatory amino acid receptors interacted with SP.  Ref: 1705317 Neurosci Lett, 1990
- Philanthotoxin caused a dose dependent reduction of responses to quisqualate, alpha amino 3 hydroxy 5 phenyl 4 isoxazolepropionate  **AMPA**  and kainate with little effect on those to N methyl D aspartate  **NMDA** .  Ref: 2085718 Br J Pharmacol, 1990
- No effect on neuronal damage induced by  **NMDA**  or  **AMPA**  could be detected.  Ref: 1976762 J Neurochem, 1990
- The distributions of N methyl D aspartate  **NMDA** , alpha amino 3 hydroxy S methyl 4 isoxazole propionic acid  **AMPA**  and kainic acid KA receptors were determined in the human cerebellum using autoradiography.  Ref: 2178036 Brain Res, 1990
- Although a combination of these antagonists blocked stimulation induced synaptic field potentials as well as  **AMPA**  and  **NMDA**  induced ionic changes and associated field potentials, Quis induced ionic changes and associated field potentials could still be observed after 60 min.  Ref: 1979665 Neurosci Lett, 1990
- N methyl D aspartate  **NMDA**  and alpha amino 3 hydroxy 5 methyl 4 isoxazolepropionate  **AMPA**  did not increase calcium i.  Ref: 1977884 J Neurochem, 1990
- Excitatory amino acids mediate fast synaptic transmission in the central nervous system through the activation of at least three distinct ionotropic receptors N methyl D aspartate  **NMDA** , the alpha amino 3 hydroxy 5 methyl isoxasole 4 propionate  **AMPA**  quisqualate QUIS and the kainate subtypes for reviews, see refs.  Ref: 2172830 Nature, 1990
- In the CA1 stratum pyramidale of rat hippocampal slices we have used calcium and Na sensitive microelectrodes to measure the changes in calcium o, Na o and associated slow field potentials elicited by the iontophoretic application of the excitatory amino acids N methyl D aspartic acid  **NMDA** , quisqualic acid Quis, alpha amino 3 hydroxy 5 methyl 4 isoazolepropionic acid  **AMPA**  and glutamic acid Glu in the presence of the  **NMDA**  receptor antagonists 2 amino 5 phosphonovaleric acid AP5 and ketamine and the non  **NMDA**  receptor antagonist 6 cyano 7 nitroquinoxaline dione CNQX.  Ref: 1979665 Neurosci Lett, 1990
- Under these conditions, glutamate and the sulfur amino acids activated a rapidly desensitizing response, similar to that evoked by micromolar concentrations of quisqualate and  **AMPA** , but mM concentrations of L aspartate, homoquinolinic acid, and quinolinic acid failed to elicit a non  **NMDA**  receptor mediated response.  Ref: 2165523 J Neurosci, 1990
- We exposed murine cortical neuronal cell cultures for 24 hours to defined concentrations of N methyl D aspartate  **NMDA** , kainate, or alpha amino 3 hydroxy 5 methyl 4 isoxazolepropionic acid  **AMPA** , and assessed the resultant neuronal degeneration quantitatively by the efflux of lactate dehydrogenase to the bathing medium.  Ref: 1974332 Neurology, 1990
- In the absence of kainate, both quisqualate and  **AMPA**  increased calcium i though less so than did  **NMDA**  or kainate.  Ref: 1980647 Eur J Pharmacol, 1990
- From the properties of these and of a purified non  **NMDA**  receptor, and the pharmacology of the native responses to  **AMPA**  and kainate, the authors conclude that multiple non  **NMDA**  subtypes exist.  Ref: 1964256 Trends Pharmacol Sci, 1990
- We examined the effects of iontophoretically applied glutamate GLU, aspartate ASP, and the specific agonists kainate KA, quisqualate QQ, RS alpha amino 3 hydroxy 5 methyl 4 isoxazole propionic acid  **AMPA** , and N methyl D aspartate  **NMDA**  on the spontaneous and light driven activity of ganglion cells.  Ref: 1980925 J Neurophysiol, 1990
- A grease gap method was used to record depolarizations evoked in CA1 hippocampal pyramidal cells by the excitants  **NMDA**  and  **AMPA**  alpha amino 3 hydroxy 5 methyl 4 isoxazolepropionate.  Ref: 2149086 Brain Res Dev Brain Res, 1990
- Philanthotoxin blocks quisqualate,  **AMPA**  and kainate... induced excitation of rat brainstem neurones in vivo.  Ref: 2085718 Br J Pharmacol, 1990
- The binding sites measured were the N methyl D aspartate  **NMDA**  sensitive sites for L 3H glutamate and 3H MK 801 sites transmitter recognition site and ion channel of the  **NMDA**  receptor, respectively, 3H alpha amino 3 hydroxy 5 methyl 4 isoxazolepropionate  **AMPA**  sites quisqualate receptor, 3H kainate sites kainate receptor and  **NMDA**  insensitive sites for L 3H glutamate.  Ref: 1964104 Brain Res, 1990
- In contrast to the cerebral cortex, where KA receptors have a complementary distribution to  **NMDA**  and  **AMPA**  receptors,  **AMPA**  receptors were concentrated in the cerebellar molecular layer while  **NMDA**  and KA receptors were concentrated in the granular layer.  Ref: 2178036 Brain Res, 1990
- Glutamate, the major excitatory neurotransmitter in the central nervous system, activates at least three types of channel forming receptors defined by the selective agonists N methyl D aspartate  **NMDA** , kainate, and quisqualate or more selectively by alpha amino 3 hydroxy 5 methyl 4 isoxazolepropionic acid  **AMPA** .  Ref: 1702227 Proc Natl Acad Sci U S A, 1990
