# Supplementary material for: Content-rich biological network constructed by mining PubMed abstracts
Source: BMC Bioinformatics. 2004 Oct 8;5:147. doi: 10.1186/1471-2105-5-147 (PMC528731; doi:10.1186/1471-2105-5-147)
Supplement: Additional File 5 — The original Chilibot query results of the term "long-term potentiation (LTP)" and 22 other terms, limiting the latest references analyzed to the years 1990, 1995, 2000, and 2004. [file 1471-2105-5-147-S5.bz2 › chilibotAdditionalFile5/ltp1990/html/NMDA_TAU.html]

 


 **NMDA** and **TAU** 
  
Found 7 abstracts in PubMed,  **7 abstracts were retrieved and analyzed**.  


---

 Search Google  |
 PDF files only 
|  EDU domain only 

---

**Interactive relationship** (e.g. stimulation, inhibition, etc)

- Stimulation with glutamate 0.1 1 mM or N methyl D aspartate  **NMDA**  0.1 mM resulted in a dramatic increase in the intensity of  **tau**  labelling in axons and the appearance of staining within a proportion of neuronal cell bodies and dendrites.  Ref: 2110639 Neurosci Lett, 1990

- :-)

  **Parallel relationship** (e.g. studied together, co-existance, homology, etc.)

  - In the presence of 5 microM  **NMDA**  at 60 mV,  **tau**  o2 = 10.49 ms and  **tau**  o1 = 1.47 ms.  Ref: 1703572 J Physiol, 1990
  - **NMDA**  responses were antagonized by 2 amino 5 phosphonovalerate APV without any effect on gamma n or  **tau**  values measured by noise analysis.  Ref: 2457087 J Physiol, 1988
