# Supplementary material for: Content-rich biological network constructed by mining PubMed abstracts
Source: BMC Bioinformatics. 2004 Oct 8;5:147. doi: 10.1186/1471-2105-5-147 (PMC528731; doi:10.1186/1471-2105-5-147)
Supplement: Additional File 5 — The original Chilibot query results of the term "long-term potentiation (LTP)" and 22 other terms, limiting the latest references analyzed to the years 1990, 1995, 2000, and 2004. [file 1471-2105-5-147-S5.bz2 › chilibotAdditionalFile5/ltp1990/html/NMDA_CAMKII.html]

 


 **NMDA** and **CAMKII** 
  
Found 3 abstracts in PubMed,  **3 abstracts were retrieved and analyzed**.  


---

 Search Google  |
 PDF files only 
|  EDU domain only 

---

- Nature, 1989   **An essential role for postsynaptic calmodulin and protein kinase activity in long term potentiation.**.
  The phenomenon of long term potentiation LTP , a long lasting increase in the strength of synaptic transmission which is due to brief, repetitive activation of excitatory afferent fibres, is one of the most striking examples of synaptic plasticity in the mammalian brain.
  In the CA1 region of the hippocampus, the induction of LTP requires activation of **NMDA** N methyl D aspartate receptors by synaptically released glutamate with concomitant postsynaptic membrane depolarization.
  This relieves the voltage dependent magnesium block of the **NMDA** receptor ion channel, allowing calcium to flow into the dendritic spine.
  Although calcium has been shown to be a necessary trigger for LTP refs 1 2 , little is known about the immediate biochemical processes that are activated by calcium and are responsible for LTP.
  The most attractive candidates have been calcium calmodulin dependent protein kinase II **[CAMKII]** CaM KII refs 13 16 , protein kinase C refs 17 19 , and the calcium dependent protease, calpain.
  Extracellular application of protein kinase inhibitors to the hippocampal slice preparation blocks the induction of LTP refs 21 23 but it is unclear whether this is due to a pre and or postsynaptic action.
  We have found that intracellular injection into CA1 pyramidal cells of the protein kinase inhibitor H 7, or of the calmodulin antagonist calmidazolium, blocks LTP.
  Furthermore, LTP is blocked by the injection of synthetic peptides that are potent calmodulin antagonists and inhibit CaM KII auto and substrate phosphorylation.
  These findings demonstrate that in the postsynaptic cell both activation of calmodulin and kinase activity are required for the generation of LTP, and focus further attention on the potential role of CaM KII in LTP.

  - Nature, 1988   **Persistent protein kinase activity underlying long term potentiation.**.
    Long term potentiation LTP of synaptic transmission in the hippocampus is a much studied example of synaptic plasticity.
    Although the role of N methyl D aspartate **NMDA** receptors in the induction of LTP is well established, the nature of the persistent signal underlying this synaptic enhancement is unclear.
    Involvement of protein phosphorylation in LTP has been widely proposed, with protein kinase C PKC and calcium calmodulin kinase type II **CaMKII** as leading candidates.
    Here we test whether the persistent signal in LTP is an enduring phosphoester bond, a long lived kinase activator, or a constitutively active protein kinase by using H 7, which inhibits activated protein kinases and sphingosine, which competes with activators of PKC ref.
    17 and **CaMKII** ref.
    18 .
    H 7 suppressed established LTP, indicating that the synaptic potentiation is sustained by persistent protein kinase activity rather than a stably phosphorylated substrate.
    In contrast, sphingosine did not inhibit established LTP, although it was effective when applied before tetanic stimulation.
    This suggests that persistent kinase activity is not maintained by a long lived activator, but is effectively constitutive.
    Surprisingly, the H 7 block of LTP was reversible.
    evidently, the kinase directly underlying LTP remains activated even though its catalytic activity is interrupted indicating that such kinase activity does not sustain itself simply through continual autophosphorylation see refs 5 .

    - Biochem Biophys Res Commun, 1987   **4 Aminopyridine inhibits synaptosomal plasma membrane protein phosphorylation in vitro effect of the selective **NMDA** antagonist 2 amino 5 phosphonovalerate.**.
      Phosphorylation of synaptosomal plasma membranes from rat hippocampus in the presence of the convulsant drug 4 aminopyridine resulted in the inhibition of the phosphorylation of the nervous tissue specific protein kinase C substrate protein B 50 48 kDa and the alpha subunit of calcium calmodulin dependent protein kinase II **[CAMKII]** 50 kDa .
      Preincubation of SPM with 2 amino 5 phosphonovalerate prevents the inhibition of B 50 phosphorylation by 4 aminopyridine, but had no effect on the inhibition of 50 kDa phosphorylation.
      2 Amino 5 phosphonovalerate is known to be a specific N methyl D aspartate antagonist and has anti epileptic activity in vitro and in vivo.
      Several other anti epileptic drugs tested did not influence the 4 aminopyridine induced inhibition of protein phosphorylation.
