# Supplementary material for: Content-rich biological network constructed by mining PubMed abstracts
Source: BMC Bioinformatics. 2004 Oct 8;5:147. doi: 10.1186/1471-2105-5-147 (PMC528731; doi:10.1186/1471-2105-5-147)
Supplement: Additional File 5 — The original Chilibot query results of the term "long-term potentiation (LTP)" and 22 other terms, limiting the latest references analyzed to the years 1990, 1995, 2000, and 2004. [file 1471-2105-5-147-S5.bz2 › chilibotAdditionalFile5/ltp1990/html/NMDA_ACTIN.html]

 


 **NMDA** and **ACTIN** 
  
Found 1 abstracts in PubMed,  **1 abstracts were retrieved and analyzed**.  


---

 Search Google  |
 PDF files only 
|  EDU domain only 

---

- Glia, 1990   **The excitatory neurotransmitter glutamate causes filopodia formation in cultured hippocampal astrocytes.**.
  Can neurons induce surrounding glia to provide a more favorable microenvironment? Synapses and nerve growth cones have been shown to release neurotransmitters Hume et al.
  Nature 1983;305 632 634.
  Kater et al.
  Trends Neurosci.
  1988;11 315 321.
  Young and Poo Nature 1983;305 634 637 providing a possible mechanism for this type of control.
  The excitatory neurotransmitter glutamate induces an increase in the number of filopodia on the surface of astrocytes cultured from the neonatal rat hippocampus.
  This seems to be associated with a receptor mediated event that is activated to a lesser degree by the quisqualate and kainate, but not **NMDA** receptors.
  In addition, time lapse video recordings have revealed a rapid extension of filopodia from the apical margins of cells treated with glutamate.
  The apical margins of glutamate treated cells studied with electron microscopy contained dense cortical **actin** networks that are devoid of microtubules.
  Coated pits are often seen to invaginate from the apical membrane in the vicinity of filopodia.
  A receptor binding step may be followed by a rapid reorganization of cortical **actin** resulting in **actin** containing filopodia.
  This process may be mediated by inositol lipid hydrolysis.
  Pyramidal neurons settled on glial cultures induced filopodia to form around the entire margin of growth cones and neurite tips suggesting that these events might occur in situ.
