# Supplementary material for: Content-rich biological network constructed by mining PubMed abstracts
Source: BMC Bioinformatics. 2004 Oct 8;5:147. doi: 10.1186/1471-2105-5-147 (PMC528731; doi:10.1186/1471-2105-5-147)
Supplement: Additional File 5 — The original Chilibot query results of the term "long-term potentiation (LTP)" and 22 other terms, limiting the latest references analyzed to the years 1990, 1995, 2000, and 2004. [file 1471-2105-5-147-S5.bz2 › chilibotAdditionalFile5/ltp1990/html/TAU_ACTIN.html]

 


 **TAU** and **ACTIN** 
  
Found 39 abstracts in PubMed,  **30 abstracts were retrieved and analyzed**.  


---

 Search Google  |
 PDF files only 
|  EDU domain only 

---

**Interactive relationship** (e.g. stimulation, inhibition, etc)

- In contrast, the  **tau**  factor  **actin**  filament interaction could only be detected by the low shear viscosity, but not by the high speed centrifugation method.  Ref: 4005297 Biochim Biophys Acta, 1985
- In conclusion, the interaction of MAP2 and  **tau**  factor  **actin**  filaments is regulated by calcium and calmodulin in a flip flop switch.  Ref: 4005297 Biochim Biophys Acta, 1985
- The amino acid composition closely resembles other actins except that Naegleria  **actin**  lacks N  **tau**  methylhistidine.  Ref: 6233284 J Biol Chem, 1984
- Calmodulin inhibits interaction of  **actin**  with MAP2 and  **Tau** , two major microtubule associated proteins.  Ref: 4030771 J Biol Chem, 1985
- MAP2 and  **tau**  factor aggregated to form  **actin**  bundles as shown by electron microscopy.  Ref: 4005297 Biochim Biophys Acta, 1985
- We argue that release of N  **tau**  methylhistidine is indicative of cardiac  **actin**  degradation.  Ref: 3541899 Biochem J, 1986
- This result indicates that N  **tau**  methylhistidine is not a prerequisite for  **actin**   **actin**  or  **actin**  myosin interactions.  Ref: 6233284 J Biol Chem, 1984
- A well known calcium dependent regulatory protein, calmodulin, inhibited both MAP2  **actin**  and  **Tau**   **actin**  interaction in a calcium dependent manner.  Ref: 4030771 J Biol Chem, 1985
- **Tau**  is not an effective cross linker of  **actin**  and microtubules even though it can interact with each polymer individually.  Ref: 3460455 Ann N Y Acad Sci, 1986
- MAP2 microtubule associated protein 2 and  **tau**  factor are calmodulin binding and  **actin**  filament interacting proteins, respectively.  Ref: 4005297 Biochim Biophys Acta, 1985
- Labels attached to F  **actin**  had a value of  **tau**  c, measured using saturation transfer electron paramagnetic resonance, of 2 X 10 5 s, which shows that the probe has a greater degree of mobility than the filament.  Ref: 3028257 Arch Biochem Biophys, 1987
- Furthermore, reactivity to  **tau** , although not associated with ubiquitin but with  **Actin**  has been described in Hirano bodies.  Ref: 1966495 Gac Med MexGac Med Mex, 1987
- The tubulin binding sequence of brain microtubule associated proteins,  **tau**  and MAP 2, is also involved in  **actin**  binding.  Ref: 2115775 Biochem J, 1990
- We have previously shown that microtubule associated protein 2 MAP2 and  **Tau** , two major microtubule associated proteins, interact with  **actin**  differently as measured by low shear viscosity.  Ref: 4030771 J Biol Chem, 1985
- The results suggest that  **tau**  could be one of the proteins involved in the concerted regulation of microtubule and  **actin**  networks in the membrane vicinity.  Ref: 6743699 Biochimie, 1984
- Labels attached to G  **actin**  displayed an absorption spectrum characteristic of rotational motion with a correlation time  **tau**  c of 7 X 10 9 s, which is faster than that for the whole molecule.  Ref: 3028257 Arch Biochem Biophys, 1987
- The interaction of  **actin**  with a synthetic peptide which corresponds to one of the repeated tubulin binding sites present in  **tau**  and MAP 2 microtubule associated protein 2 proteins has been analysed.  Ref: 2115775 Biochem J, 1990
- Evidence for an interaction between these two proteins is provided by spectrin affinity chromatography of brain MAPs, gel overlay of electrophoresed MAPs with 125I labelled spectrin, incorporation of  **tau**  factor in human erythrocyte ghosts, and demonstration that  **tau**  inhibits the F  **actin**  cross linking activity of tetrameric spectrin.  Ref: 6743699 Biochimie, 1984
- The protein kinase C mediated phosphorylation of  **tau**  reduced its abilities to promote tubulin polymerization and to cross link  **actin**  filaments.  Ref: 3109941 FEBS Lett, 1987
- The binding of heavy meromyosin or troponin tropomyosin to labeled  **actin**  resulted in a further increase in the rotational correlation times, with the greatest decrease in mobility  **tau**  c = 1 X 10 4 s observed when both were bound.  Ref: 3028257 Arch Biochem Biophys, 1987

**Parallel relationship** (e.g. studied together, co-existance, homology, etc.)

- Calmodulin inhibited MAP2 and  **tau**  factor induced  **actin**  gelation MAP2 and  **tau**  factor  **actin**  interaction only in the presence of calcium, but not in its absence.  Ref: 4005297 Biochim Biophys Acta, 1985
- In contrast,  **Tau**  reduced high shear viscosity of  **actin**  filaments and enhanced the turbidity which were due to formation of  **actin**  filament bundles as shown by electron microscopy.  Ref: 4030771 J Biol Chem, 1985
- By dynamic light scattering, the intensity autocorrelation function, G2  **tau**  = B 1 beta g1  **tau**  2, was obtained over the scattering angles theta from 30 to 130 degrees in steps of 10 degrees for semidilute solutions of muscle F  **actin**  and of F  **actin**  complexed with heavy meromyosin in the absence of ATP acto HMM, where B is the baseline and beta a constant.  Ref: 3663844 Biophys Chem, 1987
- The analysis, which uses affinity chromatography of G  **actin**  on a column containing the synthetic peptide, and the co sedimentation and co localization of F  **actin**  and the peptide as determined by immunoelectron microscopy, indicates that the part of the amino acid sequence of  **tau**  involved in the binding of tubulin is also involved in  **actin**  binding.  Ref: 2115775 Biochem J, 1990
- The intensity correlation functions G2  **tau**  of scattered light from a G  **actin**  solution containing 2 mM Tris HCl pH 8.0 and 0.1 mM ATP were analyzed by a cumulant expansion method, and the translational diffusion coefficient was determined to be D = 8.07 0.10 X 10 7 cm2 s at 20 degrees C.  Ref: 3828469 Biophys Chem, 1986
- The calmodulin dependent inhibition was canceled by higher concentrations of MAP2 or  **Tau** , and calmodulin had no effect on the viscosity of  **actin**  filament alone, indicating that this inhibition is based on the stoichiometric interaction of calmodulin with MAP2 or  **Tau** .  Ref: 4030771 J Biol Chem, 1985
- These methods should be especially valuable for determining myosin,  **actin** , and elastin in tissue hydrolysates from the amounts of N  **tau**  methylhistidine, desmosine, or isodesmosine present, respectively, and for studying protein methylation, hydroxylation, cross linking formation, and the turnover rates of contractile and connective tissue proteins in biological systems.  Ref: 3578753 Anal Biochem, 1987
- Because one mole of  **actin**  purified from the red and white muscles of Leghorn chickens and one mole of myosin contain respectively one and two moles of N  **tau**  methylhistidine, and the molar ratio of myosin and  **actin**  in skeletal muscle is known to be 1 6, the myofibrillar myosin and  **actin**  contents of avian skeletal muscles can be determined from the amounts of protein bound N  **tau**  methylhistidine found in acid hydrolysates of this tissue.  Ref: 3237578 Poult Sci, 1988
- The  **actin**  filament cross linking activity of whole MAPs, MAP 2, and  **tau**  depended on the MAP phosphate content.  Ref: 6304075 J Biol Chem, 1983
- We have determined the absolute phosphate content of microtubule associated proteins MAPs and established that phosphorylation inhibits the  **actin**  filament cross linking activity of MAPs and both of the major MAP components, MAP 2 and  **tau** .  Ref: 6304075 J Biol Chem, 1983
- Myelin basic protein as well as MAP2 could serve as good substrates for this kinase, but 40S ribosomal protein S6, casein, histone, phosphorylase b, protamine, tubulin,  **actin**  and  **tau**  could not.  Ref: 2551690 Eur J Biochem, 1989
- The turnover of 3 methylhistidine N  **tau**  methylhistidine and in some cases  **actin** , myosin heavy chain and aldolase in skeletal muscle was measured in a number of experiments in growing and adult rats in the fed and overnight starved states.  Ref: 6615482 Biochem J, 1983
- These data clearly show that, distinct from MAP2 and  **tau** , this MAP does not interact with  **actin** .  Ref: 3782289 J Cell Biol, 1986
- Studies on the role of  **actin**  s N  **tau**  methylhistidine using oligodeoxynucleotide directed site specific mutagenesis.  Ref: 3301854 J Biol Chem, 1987
- However, the inclusions did not react with any of the following antibodies Abs or antisera anti phosphorylated neurofilament NF Ab, anti nonphosphorylated NF Abs 160 and 200 kDa, anti paired helical filament antiserum, anti  **tau**  antiserum, anti tubulin Abs alpha and beta, anti microtubule associated protein antiserum, anti glial fibrillary acidic protein antiserum, anti vimentin Ab, anti desmin Ab, anti cytokeratin Abs low and high molecular weights, anti  **actin**  antiserum, anti skeletal myosin antiserum and anti myelin basic protein Ab.  Ref: 2163181 Acta Neuropathol (Berl), 1990
- MAP2 or  **tau**  factor induced bundle formation of  **actin**  filaments was inhibited only in the presence of calcium and calmodulin, but not in the presence or absence of calcium.  Ref: 4005297 Biochim Biophys Acta, 1985
- It was shown that, like bovine adrenal 190 kDa MAP, yet distinct from brain MAP2 and  **tau** , purified HeLa 180 kDa MAP does not interact with  **actin**  filaments.  Ref: 3481365 J Biochem (Tokyo), 1987
