# Supplementary material for: Content-rich biological network constructed by mining PubMed abstracts
Source: BMC Bioinformatics. 2004 Oct 8;5:147. doi: 10.1186/1471-2105-5-147 (PMC528731; doi:10.1186/1471-2105-5-147)
Supplement: Additional File 5 — The original Chilibot query results of the term "long-term potentiation (LTP)" and 22 other terms, limiting the latest references analyzed to the years 1990, 1995, 2000, and 2004. [file 1471-2105-5-147-S5.bz2 › chilibotAdditionalFile5/ltp1990/html/TAU_ACTININ.html]

 


 **TAU** and **ACTININ** 
  
Found 2 abstracts in PubMed,  **2 abstracts were retrieved and analyzed**.  


---

 Search Google  |
 PDF files only 
|  EDU domain only 

---

- Ann Pathol, 1988    **Pathology of the cytoskeleton** .
  In the last years, considerable advances have been made in the study of the proteins and polypeptides of the cytoskeleton, and its three main components microfilaments MF , intermediate filaments IMF and microtubules MT .
  The principal properties of these elements and those of many associated proteins are recalled.
  The actin MF are mainly involved in cell contractility, the IMF in cell shape, while the MT and their associated proteins are involved in intracellular transport.
  Some pathological modifications of the cytoskeleton will be considered.
  In the liver, accumulations of keratin result in the formation of Mallory s hyalin, found in several types of cirrhosis and hepatomas.
  In muscle, accumulations of desmin are observed in various myopathies.
  An accumulation of alpha **actinin** at the Z bands characterizes nemalin myopathy.
  In several forms of hemolytic anemias, alterations of the membranous cytoskeletal components of the red blood cells spectrin, ankyrin, actin may explain their abnormal shape and excessive fragility.
  In the nervous system, many pathological conditions are related to abnormal cytoskeletal components.
  In Parkinson s disease, Lewy bodies are an accumulation of neurofilaments IMF .
  In Alzheimer s disease, and some related conditions, the intraneuronal tangles are associated with modifications of MT and neurofilaments.
  The role of MT and in particular of the MT associated protein **tau**, as demonstrated recently, confirms the involvement of the MT.
  The observed disturbances of MT related axonal flow may explain some of the known functional changes in these forms of dementia.

  - Brain Res, 1987   **Hirano bodies contain **tau** protein.**.
    Hirano bodies are intraneuronal inclusions whose frequency increases with age and Alzheimer s disease.
    These paracrystalline inclusions have been shown previously using immunocytochemistry to share epitopes with actin, tropomyosin, alpha **actinin** and vinculin.
    Hirano bodies have not previously been demonstrated to share components with neurofibrillary tangles, another intraneuronal inclusion characteristic of Alzheimer s disease.
    In this study, we show that Hirano bodies bind antibodies to the microtubule associated protein **tau**, a component of Alzheimer neurofibrillary tangles.
