# Supplementary material for: Content-rich biological network constructed by mining PubMed abstracts
Source: BMC Bioinformatics. 2004 Oct 8;5:147. doi: 10.1186/1471-2105-5-147 (PMC528731; doi:10.1186/1471-2105-5-147)
Supplement: Additional File 5 — The original Chilibot query results of the term "long-term potentiation (LTP)" and 22 other terms, limiting the latest references analyzed to the years 1990, 1995, 2000, and 2004. [file 1471-2105-5-147-S5.bz2 › chilibotAdditionalFile5/ltp1990/html/TAU_SYNAPTOPHYSIN.html]

 


 **TAU** and **SYNAPTOPHYSIN** 
  
Found 5 abstracts in PubMed,  **5 abstracts were retrieved and analyzed**.  


---

 Search Google  |
 PDF files only 
|  EDU domain only 

---

**Interactive relationship** (e.g. stimulation, inhibition, etc)

**Parallel relationship** (e.g. studied together, co-existance, homology, etc.)

- ... reactivity for microtubule associated protein 2 MAP2,  **tau** , the 200 kilodalton isoform of neurofilament protein, neuron specific enolase and  **synaptophysin**  was primarily seen in maturing neurons.  Ref: 2586719 Neuropathol Appl NeurobiolNeuropathol Appl Neurobiol, 1987
- Mature ganglion cells were also immunoreactive for proteins associated with the neuronal cytoskeleton including microtubule associated proteins, MAP2 and  **tau** , and higher molecular weight phosphorylated and non phosphorylated neurofilament subunits, neuron specific enolase, and  **synaptophysin** .  Ref: 2817080 Am J Pathol, 1989
- were strongly reactive for neuron specific enolase,  **synaptophysin** , and neuronal cytoskeletal proteins 68 and 200 kd subunits of neurofilament protein, microtubule associated protein 2, and  **tau** .  Ref: 2757489 Arch Pathol Lab Med, 1989
- Neurons were present, as determined by immunostaining with antibodies to 4 neuron specific proteins neuron specific enolase, microtubule associated protein 2,  **tau**  protein and  **synaptophysin** .  Ref: 2350878 Brain Res Dev Brain Res, 1990
