# Supplementary material for: Content-rich biological network constructed by mining PubMed abstracts
Source: BMC Bioinformatics. 2004 Oct 8;5:147. doi: 10.1186/1471-2105-5-147 (PMC528731; doi:10.1186/1471-2105-5-147)
Supplement: Additional File 5 — The original Chilibot query results of the term "long-term potentiation (LTP)" and 22 other terms, limiting the latest references analyzed to the years 1990, 1995, 2000, and 2004. [file 1471-2105-5-147-S5.bz2 › chilibotAdditionalFile5/ltp1990/html/ARC_ACTIN.html]

 


 **ARC** and **ACTIN** 
  
Found 3 abstracts in PubMed,  **3 abstracts were retrieved and analyzed**.  


---

 Search Google  |
 PDF files only 
|  EDU domain only 

---

**Interactive relationship** (e.g. stimulation, inhibition, etc)

- An important structural feature of these cells is an  **actin**  containing  **arc**  like band on the periphery of lamella.  Ref: 7197563 Biull Eksp Biol Med, 1981

**Parallel relationship** (e.g. studied together, co-existance, homology, etc.)

- Contractile  **arc**   **actin**  filaments were revealed to be crosslinked by thin strands by the rapid freezing deep etching replication technique.  Ref: 3413069 Proc Natl Acad Sci U S A, 1988
- From electron microscopic observation of thin sections of the furrow, it was suggested that the  **actin**  bundles of the contractile  **arc**  were organized from preexisting cortical filaments that were connected to the plasma membrane by granular materials at their barbed ends.  Ref: 3413069 Proc Natl Acad Sci U S A, 1988
- Besides stress fibers,  **arc**  like  **actin**  bundles have also been detected in spreading cells.  Ref: 6541223 J Cell Biol, 1984
