# Supplementary material for: Content-rich biological network constructed by mining PubMed abstracts
Source: BMC Bioinformatics. 2004 Oct 8;5:147. doi: 10.1186/1471-2105-5-147 (PMC528731; doi:10.1186/1471-2105-5-147)
Supplement: Additional File 5 — The original Chilibot query results of the term "long-term potentiation (LTP)" and 22 other terms, limiting the latest references analyzed to the years 1990, 1995, 2000, and 2004. [file 1471-2105-5-147-S5.bz2 › chilibotAdditionalFile5/ltp1990/html/ACTIN_ACTININ.html]

 


 **ACTIN** and **ACTININ** 
  
Found 400 abstracts in PubMed,  **30 abstracts were retrieved and analyzed**.  


---

 Search Google  |
 PDF files only 
|  EDU domain only 

---

**Interactive relationship** (e.g. stimulation, inhibition, etc)

- The interaction of filamentous  **actin**  with alpha  **actinin** , an  **actin**  cross linking protein, is well established.  Ref: 2124107 Biochem Biophys Res Commun, 1990
- On the other hand, monomeric  **actin**  alpha  **actinin**  interaction has been a subject of controversy.  Ref: 2124107 Biochem Biophys Res Commun, 1990
- These  **actin**  structures colocalize with the  **actin**  bundling protein alpha  **actinin**  as well as with tropomyosin.  Ref: 2117270 Proc Natl Acad Sci U S A, 1990
- We thus conclude that, in vivo, alpha  **actinin**  acts as an  **actin**  gelling protein.  Ref: 2226825 FEBS Lett, 1990
- These results lead us to suggest that this integrin alpha  **actinin**  linkage may contribute to the attachment of  **actin**  filaments to the membrane in certain locations.  Ref: 2116421 J Cell Biol, 1990
- The connecting structure is probably composed of alpha  **actinin**  which is located in Z bands and cross links  **actin**  filaments.  Ref: 2269662 J Cell Biol, 1990
- The functional form of filamin is single stranded, in contrast to alpha  **actinin**  and spectrin which are antiparallel double stranded  **actin**  cross linkers.  Ref: 2248958 Biochemistry, 1990
- In this report, we have characterized the interaction of monomeric  **actin** , coated on plastic plates under conditions of non polymerization, with alpha  **actinin**  in presence of magnesium.  Ref: 2124107 Biochem Biophys Res Commun, 1990
- Here we report our finding of an interaction between the cytoplasmic domain of beta 1 integrin and the  **actin**  binding protein alpha  **actinin** .  Ref: 2116421 J Cell Biol, 1990
- In the presence of dextran, to minimize protein loss, the supercontracted products were star like in form, comprising long  **actin**  bundles radiating in all directions from a central core containing myosin, desmin, and alpha  **actinin** .  Ref: 2277067 J Cell Biol, 1990
- Secondary antibody staining for alpha  **actinin** , a protein that binds and crosslinks  **actin** , was more prominent after treatment with thyrotropin but decreased after TPA.  Ref: 2247845 Surgery, 1990
- At 37 degrees C, in the presence of 6% w v polyethylene glycol 600 0 nM alpha  **actinin**  from chicken gizzard induces the gelation of 12 microM  **actin** .  Ref: 2226825 FEBS Lett, 1990

**Parallel relationship** (e.g. studied together, co-existance, homology, etc.)

- The authors suggest that the major function of  **actin** , myosin, and alpha  **actinin**  containing filament bundles in mesangial cells is to create an isometric tension or minute isotonic contractions to counteract the distending forces of the rather high intracapillary hydraulic pressure and its resulting pressure gradients across the capillary wall and across the perimesangial GBM.  Ref: 2260624 Am J Pathol, 1990
- Futhermore we recognize sequence elements of a putative  **actin**  binding domain of alpha  **actinin** , the calpactin I or p 36 sequence, and a consensus motif present in the repeats of the gene product of the candidate unc 87 gene of C. elegans S.D.  Ref: 2253766 FEBS Lett, 1990
- The sarcomeric localization of antibodies to desmin, alpha  **actinin** , titin, troponin I, alpha  **actin** , myosin heavy chain, and myomesin in these converted myoblasts are indistinguishable from in vivo and in vitro normal myoblasts.  Ref: 2172969 Proc Natl Acad Sci U S A, 1990
- This repeat is homologous with the  **actin**  binding domain of alpha  **actinin**  and the amino terminal domains of dystrophin,  **actin**  gelation protein, and beta spectrin.  Ref: 2391360 J Cell Biol, 1990
- Localization of a new alpha  **actinin**  binding site in the COOH terminal part of  **actin**  sequence.  Ref: 2124107 Biochem Biophys Res Commun, 1990
- In cultured mesangial cells,  **actin** , myosin, and alpha  **actinin**  constitute a considerable amount of the total cellular protein contents.  Ref: 2260624 Am J Pathol, 1990
- Microinjection of fluorescently labeled alpha  **actinin**  in living cells demonstrates that the formation of these F  **actin**  projections is associated with bacterial movement,  **actin**  filaments rapidly assembling behind the bacteria as they migrate through the cytoplasm.  Ref: 2117270 Proc Natl Acad Sci U S A, 1990
- Previous studies have shown that cis unsaturated free fatty acids uFFAs are able to cause alterations in the normal distribution pattern of certain cytoskeletal proteins in lymphocytes, including tubulin,  **actin** , alpha  **actinin** , and myosin.  Ref: 1714352 Cell Biophys, 1990
- In addition, PTH induced cytoskeletal disassembly as shown by a 52 70% decrease in the Triton insoluble fractions of  **actin** , alpha tubulins and alpha  **actinin** .  Ref: 2168775 Bone Miner, 1990
- This was shown for  **actin** , myosin, and alpha  **actinin**  by immunoblotting as well as by immunoelectron microscopy.  Ref: 2260624 Am J Pathol, 1990
- On the basis of these results and considering the role of clathrin in intracellular transport and its capacity to interact with  **actin**  and alpha  **actinin** , we suggest that clathrin may have diverse roles in the assembly, integrity, and functioning of the sarcomere and its integration with the sarcolemma.  Ref: 1701722 Exp Cell Res, 1990
- 5 OH 2D 10 nM, 24 h also induced a 40 64% decrease in the polymerized fractions of  **actin** , alpha tubulins and alpha  **actinin** .  Ref: 2168775 Bone Miner, 1990
- The connecting structure may consist of two alpha  **actinin**  molecules linking  **actin**  filaments of opposite polarity.  Ref: 2269662 J Cell Biol, 1990
- The  **actin**  binding domain is near the amino terminus of the subunit where the amino acid sequence is similar to other  **actin**  filament binding proteins, including alpha  **actinin** , beta spectrin, dystrophin, and Dictyostelium abp 120.  Ref: 2391361 J Cell Biol, 1990
- This protein has been purported to be, or be related to alpha  **actinin** , a highly conserved family of  **actin**  binding cytoskeletal proteins common to many tissues across a wide phylogenetic range.  Ref: 1700943 Curr Eye Res, 1990
- Platelet agglutination induced by bovine vWf generated a complete cytoskeletal core Triton insoluble residue, shown by sodium dodecyl sulfate polyacrylamide gel electrophoresis SDS PAGE to be composed of  **actin**  binding protein ABP 260 Kd, 235 Kd protein, myosin heavy chain 200 Kd, alpha  **actinin**  100 Kd, and  **actin**  43 Kd.  Ref: 2119831 Blood, 1990
- Untreated human bone cells showed elongated morphology associated with high levels of  **actin** , vimentin, alpha and beta tubulins and alpha  **actinin**  as determined by 2 dimensional gel electrophoresis and 35S methionine labelling of cytoskeletal proteins.  Ref: 2168775 Bone Miner, 1990
- Sequences derived from large peptides mapping near the amino terminal show homology to the amino terminal  **actin**  binding site of alpha  **actinin**  chicken fibroblast and Dictyostelium, Dictyostelium 120 kDa  **actin**  gelation factor, beta spectrin human red cell and Drosophila, and human dystrophin.  Ref: 2248958 Biochemistry, 1990
- A number of cytoskeletal associated proteins that are concentrated in focal contacts, namely alpha  **actinin** , vinculin, talin, and integrin, have been shown to interact in vitro such that they suggest a potential link between  **actin**  filaments and the membrane.  Ref: 2116421 J Cell Biol, 1990
- In mesangial cells in situ  **actin** , myosin and alpha  **actinin**  were found to be colocalized within conspicuous microfilament bundles that traverse the cell body or major processes in various directions and project into either the tonguelike pericapillary processes, which run toward mesangial angles, or into the microvilluslike lateral extensions that abut on the perimesangial portion of the glomerular basement membrane GBM.  Ref: 2260624 Am J Pathol, 1990
- Thereby, the GBM of opposing mesangial angles as well as of opposing portions of the perimesangial GBM are regularly interconnected by filament bundles within mesangial cells that contain  **actin** , myosin, and alpha  **actinin** .  Ref: 2260624 Am J Pathol, 1990
- The  **actin**  gelling activity of chicken gizzard alpha  **actinin**  at physiological temperature is triggered by water sequestration.  Ref: 2226825 FEBS Lett, 1990
- Sequences in this region exhibit striking sequence homology, at both nucleotide and amino acid levels, to the N terminal  **actin**  binding domains of alpha  **actinin**  and dystrophin.  Ref: 2195026 J Biol Chem, 1990
- During premyofibril stages, I Z I proteins were detected first alpha  **actinin**  dots and diffuse  **actin**  phalloidin and anti troponin C staining, and later in these areas connectin and myosin dots appeared with nearly identical distribution.  Ref: 2266168 J Muscle Res Cell Motil, 1990
- Glomerular mesangial cells of the rat kidney contain  **actin** , nonmuscle myosin, tropomyosin, and the muscular Z line protein, alpha  **actinin** .  Ref: 2260624 Am J Pathol, 1990
- We report the cloning and characterization of a full length cDNA encoding the human cytoskeletal isoform of alpha  **actinin**  alpha A, a ubiquitous  **actin**  binding protein that shares structural homology with spectrin and dystrophin.  Ref: 2349951 Am J Hum Genet, 1990
- Using a photoactivatable, radiolabel transfer cross linker, 1 N 2 hydroxy 5 azidobenzoyl 2 aminoethyl 4 N hydroxysuccinimidyl succinate, we have determined that the binding site for protein 4.1 on spectrin resides in the N terminal region of beta spectrin within a sequence homologous to the  **actin**  binding region of alpha  **actinin** .  Ref: 2249696 Eur J Biochem, 1990
- Assessment of relative molecular weight, immunologic cross reactivity, and partial sequence analysis suggest that the 115 kD lens fiber cell cytoskeletal protein and alpha  **actinin**  are either unrelated, or, at best, that the lens protein represents an unusually divergent isoform of the alpha  **actin**  family of proteins.  Ref: 1700943 Curr Eye Res, 1990
- The other involves the  **actin**  binding protein, alpha  **actinin** , which has been found to interact with several integrins.  Ref: 2129156 Cell Differ Dev, 1990
