# Supplementary material for: Content-rich biological network constructed by mining PubMed abstracts
Source: BMC Bioinformatics. 2004 Oct 8;5:147. doi: 10.1186/1471-2105-5-147 (PMC528731; doi:10.1186/1471-2105-5-147)
Supplement: Additional File 5 — The original Chilibot query results of the term "long-term potentiation (LTP)" and 22 other terms, limiting the latest references analyzed to the years 1990, 1995, 2000, and 2004. [file 1471-2105-5-147-S5.bz2 › chilibotAdditionalFile5/ltp1990/html/ACTIN_SYNAPTOPHYSIN.html]

 


 **ACTIN** and **SYNAPTOPHYSIN** 
  
Found 11 abstracts in PubMed,  **11 abstracts were retrieved and analyzed**.  


---

 Search Google  |
 PDF files only 
|  EDU domain only 

---

**Interactive relationship** (e.g. stimulation, inhibition, etc)

**Parallel relationship** (e.g. studied together, co-existance, homology, etc.)

- In an attempt to address this issue, we performed an immunohistochemical analysis of nine cases, using antibodies to vimentin, S100 protein, Leu 7 antigen, neuron specific enolase,  **synaptophysin** , desmin, muscle specific  **actin** , cytokeratin, and epithelial membrane antigen, and the avidin biotin peroxidase complex ABC method.  Ref: 2202275 Arch Pathol Lab Med, 1990
- Both types of tumor giant cells stained for vimentin, alpha 1 antitrypsin, alpha 1 antichymotrypsin,  **synaptophysin** , muscle  **actin** , and neuron specific enolase, but not for epithelial markers.  Ref: 2227926 Hum Pathol, 1990
- Vimentin was expressed in the tumor cells, but there was no immunoreactivity for cytokeratins, neurofilaments, muscle  **actin** ,  **synaptophysin** , S 100, melanoma antigen HMB 45, epithelial membrane antigen, neuron specific enolase, Leu 7, leucocyte common antigen or lysozyme alpha 1 antitrypsin.  Ref: 3372787 J Cutan Pathol, 1988
- Immunohistochemical analysis indicates that the cells express  **actin**  but lack S 100 protein,  **synaptophysin** , desmin, keratin, and epithelial membrane antigen.  Ref: 2712186 Am J Surg Pathol, 1989
- None contained desmin,  **actin** , cytokeratin, epithelial membrane antigen, or  **synaptophysin** .  Ref: 2202275 Arch Pathol Lab Med, 1990
