# Supplementary material for: Content-rich biological network constructed by mining PubMed abstracts
Source: BMC Bioinformatics. 2004 Oct 8;5:147. doi: 10.1186/1471-2105-5-147 (PMC528731; doi:10.1186/1471-2105-5-147)
Supplement: Additional File 5 — The original Chilibot query results of the term "long-term potentiation (LTP)" and 22 other terms, limiting the latest references analyzed to the years 1990, 1995, 2000, and 2004. [file 1471-2105-5-147-S5.bz2 › chilibotAdditionalFile5/ltp1995/html/LTP.html]

 


**LTP** (Input: LTP ) 

---


|  |
| --- |
| **Google Searches:** Entire Web  | EDU domain only  | PDF files only |

.

|  |
| --- |
| **External Links:** OMIM | LocusLink | Swissprot | GeneCards |

  
**Maps of LTP**

|  |
| --- |
| Simple Complete graph in radiant tree square layout. |

**New Hypothesis !**

|  |
| --- |
|  |

**Synonyms** 

|  |
| --- |
| - ltp   [PubMed] |
| - long term potentiation   [PubMed] |

**Synopsis**

|  |
| --- |
| - These results show that, during the maintenance phase of **LTP** in the hippocampus, there are selective increases in the expression of serine threonine kinases.  Neuron, 1994    [23] |
| - This hypothesis is confirmed by the experiments in which **LTP** like phenomena for early and late cortical IPSPs were shown to be the result of inactivation of PKA and PKC.  Zh Vyssh Nerv Deiat Im I P PavlovaZh Vyssh Nerv Deiat Im I P Pavlova, 1994    [22] |
| - These results suggest that glutamate can activate CaM kinase II through NMDA receptors in the induction of **LTP** and in turn stimulates the phosphorylation of target proteins such as MAP2 and synapsin I.  Nippon Yakurigaku Zasshi, 1993    [20] |
| - The data suggest that a membrane insertion of alpha beta PKC is NOT a prerequisite for the **LTP** induced increased phosphorylation of PKC substrates.  Brain Res, 1995    [20] |
| - As the epsilon PKC could be activated by arachidonic acid, it is strongly suggested that in the case of **LTP**, the presynaptic epsilon PKC is activated by arachidonic acid released postsynaptically and phosphorylates GAP 43, resulting in the increase in glutamate release.  Nippon Yakurigaku Zasshi, 1995    [20] |
| - In addition, a role for C kinase in long term potentiation  [**LTP**]  has been proposed.  Synapse, 1992    [19] |
| - protein kinase A, tyrosine kinases, mitogen activated kinase play roles in the expression of **LTP**.  Int J Biochem, 1994    [19] |
| - Recent findings demonstrate a central role for the cAMP signaling pathway in the persistent phase of long term potentiation  [**LTP**] .  Curr Opin Neurobiol, 1995    [19] |
| - Such animals have previously been shown to exhibit deficits in NE sensitivity as well as significant impairment in their ability to exhibit **LTP**.  Proc Natl Acad Sci U S A, 1991    [19] |
| - Experiments performed to determine whether the **LTP** involves a stimulus induced release of dopamine or requires a background level of dopamine receptor activation suggest neither is the case, as tetanization in the presence of a D1 receptor antagonist, which blocks the dopamine effects, produced an **LTP** comparable to that in the absence of the blocker.  Neuropharmacology, 1995    [18] |
| - Production of NO by neurons is critical for facilitated synaptic transmission in models of synaptic plasticity such as long term potentiation  [**LTP**]  and long term depression, suggesting a role for NO as a retrograde messenger that could complete a hypothetical feedback loop by strengthening the connection between postsynaptic and presynaptic cells.  Nature, 1993    [16] |
| - This effect, presumably mediated by effects on calmodulin, is a previously unreported action of cocaine, and suggests that cocaine at high dose levels might disrupt types of learning that are mediated by an **LTP** like mechanism.  Brain Res, 1993    [16] |
| - Moreover, the effects of these compounds on hippocampal transmission, in general, suggest that attribution of the amnestic consequences of their administration to impaired **LTP** may be unwarranted.  Behav Neural Biol, 1994    [16] |
| - Second, synergy of IP accumulation in correlation with synergy of neurotransmitter release elicited by mAChR activation and membrane depolarization, suggests a possible role for phospholipase C PLC in the bifurcating control of neurotransmitter release and for the involvement of PLC and voltage sensitive channels in mediation of long term potentiation  [**LTP**]  **LTP** .  Neurosci Lett, 1990    [16] |
| - Consistent with models claiming a role for long term potentiation  [**LTP**]  **LTP** in memory, **LTP** in hippocampal slices from CREB mutants decayed to baseline 90 min after tetanic stimulation.  Cell, 1994    [16] |
