# Supplementary material for: Content-rich biological network constructed by mining PubMed abstracts
Source: BMC Bioinformatics. 2004 Oct 8;5:147. doi: 10.1186/1471-2105-5-147 (PMC528731; doi:10.1186/1471-2105-5-147)
Supplement: Additional File 5 — The original Chilibot query results of the term "long-term potentiation (LTP)" and 22 other terms, limiting the latest references analyzed to the years 1990, 1995, 2000, and 2004. [file 1471-2105-5-147-S5.bz2 › chilibotAdditionalFile5/ltp1995/html/SYNAPSIN I.html]

 


**SYNAPSIN I** (Input: SYNAPSIN I ) 

---


|  |
| --- |
| **Google Searches:** Entire Web  | EDU domain only  | PDF files only |

.

|  |
| --- |
| **External Links:** OMIM | LocusLink | Swissprot | GeneCards |

  
**Maps of SYNAPSIN I**

|  |
| --- |
| Simple Complete graph in radiant tree square layout. |

**New Hypothesis !**

|  |
| --- |
|  |

**Synonyms** 

|  |
| --- |
| - synapsin i   [PubMed] |

**Synopsis**

|  |
| --- |
| - These results suggest that CaM KII activity, possibly through phosphorylation of presynaptic **synapsin I**, is required for sustained synaptic transmission at mammalian synapses.  Brain Res, 1993    [23] |
| - These results suggest significant contribution of **synapsin I** to the formation and maintenance of the presynaptic structure.  J Cell Biol, 1995    [23] |
| - These results suggest that glutamate can activate CaM kinase II through NMDA receptors in the induction of LTP and in turn stimulates the phosphorylation of target proteins such as MAP2 and **synapsin I**.  Nippon Yakurigaku Zasshi, 1993    [20] |
| - These results suggest that glutamate can activate CaM kinase II through the ionotropic NMDA receptor, which in turn increases the phosphorylation of microtuble associated protein 2 and **synapsin I**.  J Biol Chem, 1992    [20] |
| - SynapsinI and synapsin II are widely expressed synaptic vesicle phosphoproteins that have been proposed to play an important role in synaptic transmission and synaptic plasticity.  Neuropharmacology, 1995    [19] |
| - The enzyme phosphorylated smooth muscle myosin light chain, **synapsin I**, microtubule associated protein 2, tau protein, myelin basic protein, histone H1, and tyrosine hydroxylase in a calcium calmodulin dependent manner, suggesting that the enzyme is a multifunctional calmodulin dependent protein kinase capable of phosphorylating a large number of substrates.  J Biol Chem, 1992    [16] |
| - We hypothesize that the beta adrenergic agonist stimulated phosphorylation of **synapsin I** and synapsin II in young rats plays a role in the increase in transmitter release produced by NE in the dentate.  Proc Natl Acad Sci U S A, 1991    [16] |
| - Phalloidin staining and immunohistochemistry showed that the neuroblast was richer in F actin, beta tubulin, MAP1, MAP2, tau, calspectin, and **synapsin I** than the matrix cell.  Arch Histol Cytol, 1992    [15] |
| - From this data we conclude that the **synapsin I** gene is a target of the zif268 transcription factor.  J Biol Chem, 1994    [14] |
| - These studies indicated that PdBu increased the phosphorylation of multiple sites on **synapsin I**.  Synapse, 1992    [10] |
| - Regulation of neuronal plasticity by phosphorylation of **synapsin I** and of postsynaptic substrates necessary for long term potentiation is another dynamic area of investigation.  Annu Rev Biochem, 1992    [10] |
| - Biochemical experiments demonstrated that cocaine can inhibit phosphorylation of purified **Synapsin I** by calcium calmodulin dependent protein kinase II.  Brain Res, 1993    [10] |
| - Polyphosphoinositidesas activators of PKC dependent **synapsin I** phosphorylation.  FEBS Lett, 1991    [10] |
| - Third, AtT 20 cells express the neuron specific phosphoprotein **synapsin I** which accumulates in the growth cones prior to contacts forming between growth cones and cells.  Eur J Cell Biol, 1989    [10] |
| - Here we report that application of high   J Biol Chem, 1995    [10] |
