# Supplementary material for: Content-rich biological network constructed by mining PubMed abstracts
Source: BMC Bioinformatics. 2004 Oct 8;5:147. doi: 10.1186/1471-2105-5-147 (PMC528731; doi:10.1186/1471-2105-5-147)
Supplement: Additional File 5 — The original Chilibot query results of the term "long-term potentiation (LTP)" and 22 other terms, limiting the latest references analyzed to the years 1990, 1995, 2000, and 2004. [file 1471-2105-5-147-S5.bz2 › chilibotAdditionalFile5/ltp1995/html/PLC.html]

 


**PLC** (Input: PLC ) 

---


|  |
| --- |
| **Google Searches:** Entire Web  | EDU domain only  | PDF files only |

.

|  |
| --- |
| **External Links:** OMIM | LocusLink | Swissprot | GeneCards |

  
**Maps of PLC**

|  |
| --- |
| Simple Complete graph in radiant tree square layout. |

**New Hypothesis !**

|  |
| --- |
|  |

**Synonyms** 

|  |
| --- |
| - plc   [PubMed] |

**Synopsis**

|  |
| --- |
| - These results suggest that PGF2 alpha induced PLD activation is different from **PLC** PKC systems.  Prostaglandins, 1995    [23] |
| - These results suggest that two different pathways, the c ras and the **PLC** gamma pathway, are activated by TrkB receptors in primary neurons.  J Neurochem, 1995    [23] |
| - These results strongly suggest that a PC **PLC** aSMase pathway contributes directly to the propagation of Fas APO 1 generated apoptotic signal in lymphoid cells.  EMBO J, 1995    [23] |
| - This response was inhibited by preincubating the cells with an inhibitor of phospholipase C **PLC**, U73122, suggesting that **PLC** mediates the induction of PKC translocation by PDGF.  Neurochem Res, 1995    [20] |
| - These results suggest that lyn plays an important role in CD40 mediated PTK activation and identify **PLC** gamma 2 and PI 3 kinase  [PI-3K]  targets for CD40 mediated phosphorylation, suggesting a role for these two enzymes in CD40 signal transduction.  J Exp Med, 1994    [20] |
| - Collectively, these data suggest that LTB4 activates the NADPH oxidase in eosinophils by PLD and PtdIns 3 kinase independent mechanisms that involve calcium, **PLC** and PKC.  Biochem J, 1995    [20] |
| - The results suggest a crosstalk between a G protein linked receptor and a receptor tyrosine kinase, involving signalling via **PLC** beta and PKC to a downstream protein tyrosine phosphatase functioning in the control of EGF receptor activity.  FEBS Lett, 1995    [20] |
| - These results suggest that mastoparan induces phosphatidylcholine PC hydrolysis by activation of PLD, NOT by activation of phosphatidylcholine specific phospholipase C PC **PLC** .  Br J Pharmacol, 1995    [20] |
| - expression of **PLC** gamma is very low in intact vascular wall where SMC show a contractile phenotype, and induced when SMC are converted to a synthetic phenotype in culture.  Ann N Y Acad Sci, 1995    [16] |
| - These observations suggest that the 1S,3R ACPD induced burst firing is NOT mediated by **PLC** coupled metabotropic glutamate receptors.  Neuropharmacology, 1994    [16] |
| - PC12 cells expressing the beta PDGF receptor extend neurites in response to PDGF in the absence of signalling through PI 3 kinase  [PI-3K] , RasGAP, and **PLC** gamma 1.  Curr Biol, 1995    [16] |
| - Second, synergy of IP accumulation in correlation with synergy of neurotransmitter release elicited by mAChR activation and membrane depolarization, suggests a possible role for phospholipase C **PLC** in the bifurcating control of neurotransmitter release and for the involvement of **PLC** and voltage sensitive channels in mediation of long term potentiation  [LTP]  LTP .  Neurosci Lett, 1990    [16] |
| - It activates phospholipase C **PLC** resulting in an increase in cytosolic calcium and diacylglycerol DAG that are the physiological activators of protein kinase C PKC .  FEBS Lett, 1995    [14] |
| - These results indicate that tyrosine phosphorylation of **PLC** gamma 1, GAP, and PI 3 kinase  [PI-3K]  are specific responses for VSMC hyperplasia  Am J Physiol, 1994    [14] |
| - When cells are stimulated with a ligand for a receptor, such as ATP or PDGF, **PLC** is activated via either a G protein dependent or independent process, leading to the production of diacylglycerol DAG and inositol triphosphate IP3 .  J Lipid Mediat Cell Signal, 1995    [13] |
