# Supplementary material for: Content-rich biological network constructed by mining PubMed abstracts
Source: BMC Bioinformatics. 2004 Oct 8;5:147. doi: 10.1186/1471-2105-5-147 (PMC528731; doi:10.1186/1471-2105-5-147)
Supplement: Additional File 5 — The original Chilibot query results of the term "long-term potentiation (LTP)" and 22 other terms, limiting the latest references analyzed to the years 1990, 1995, 2000, and 2004. [file 1471-2105-5-147-S5.bz2 › chilibotAdditionalFile5/ltp1995/html/TRKA.html]

 


**TRKA** (Input: TRKA ) 

---


|  |
| --- |
| **Google Searches:** Entire Web  | EDU domain only  | PDF files only |

.

|  |
| --- |
| **External Links:** OMIM | LocusLink | Swissprot | GeneCards |

  
**Maps of TRKA**

|  |
| --- |
| Simple Complete graph in radiant tree square layout. |

**New Hypothesis !**

|  |
| --- |
|  |

**Synonyms** 

|  |
| --- |
| - trk a   [PubMed] |
| - trka   [PubMed] |

**Synopsis**

|  |
| --- |
| - These results show that NT3 can signal through **TrkA** and TrkB in neurons at certain stages of development and may explain why the phenotype of NT3 mice is more severe than that of trkC mice.  EMBO J, 1995    [20] |
| - Function perturbing antibodies to the p75 low affinity NGF receptor potentiated the NT 3 responses of both forms of **TrkA** in the transfected PC12nnr5 cell lines, suggesting that the low affinity NGF receptor suppresses the ability of **TrkA** to respond to NT 3.  Proc Natl Acad Sci U S A, 1994    [16] |
| - Mechanistically, NGF stimulation of PC12 cells resulted in a weak or possibly indirect association between **trkA** and PI 3 kinase  [PI-3K] .  J Neurosci Res, 1995    [14] |
| - Our results indicate that trk A  [**TRKA**] , B and C messenger RNAs are expressed throughout development, and in adulthood.  Neuroscience, 1995    [14] |
| - Regardless of the presence or absence of p75 NGFR, neither DA group expresses **trkA** mRNA, indicating that these two major hypothalamic subsets of DNA neurons are NGF insensitive.  J Neurosci, 1995    [14] |
| - At P5, the innermost part of the inner nuclear layer INL expressed **TrkA**, TrkB and p75 mRNAs.  Brain Res, 1995    [14] |
| - trkC mRNA is expressed first, followed by trkB mRNA and finally **trkA** mRNA.  Neuroreport, 1995    [14] |
| - **trkA** mRNA, which encodes the high affinity NGF receptor, was undetectable in either region.  Brain Res Dev Brain Res, 1995    [13] |
| - andhigh levels of trkC mRNA, and relatively high amount of trkB mRNA, while levels of **trkA** mRNA was undetectable.  Neuroreport, 1995    [13] |
| - Furthermore, even after transfection with exogenous TRK A  [**TRKA**] , early responses were restored but later events such as neurite outgrowth did NOT occur, suggesting that downstream responsiveness is blocked as well.  Prog Clin Biol Res, 1994    [12] |
| - Taken together, the results indicate the existence in postnatal rat brain of a large overlapping population of cholinergic neurons that are responsive to ligands for the neurotrophin receptors **TrkA** and TrkB,   Brain Res, 1995    [11] |
| - In contrast to the drop in NGF and **trkA** mRNA expression, NT 4 mRNA levels increased at the time of follicular assembly, coinciding with the abrupt appearance of trkB mRNA.  Endocrinology, 1995    [11] |
| - Thus, astroglial cells in culture internalize NGF through a specific receptor mediated process, express **trkA** and full length trkB mRNAs at low levels, and respond to exogenous NGF by expressing a fibrous morphology under serum free culture conditions.  J Neurosci Res, 1995    [11] |
| - Further tracking down of this NGF binding site in **TrkA** strikingly revealed that a single LRM of 24 amino acids could bind NGF selectively with nanomolar affinity.  J Biol Chem, 1995    [10] |
| - NT 3 is a somewhat promiscuous ligand that can activate **TrkA** and TrkB receptors at high concentrations.  Ann N Y Acad Sci, 1995    [10] |
