# Supplementary material for: Content-rich biological network constructed by mining PubMed abstracts
Source: BMC Bioinformatics. 2004 Oct 8;5:147. doi: 10.1186/1471-2105-5-147 (PMC528731; doi:10.1186/1471-2105-5-147)
Supplement: Additional File 5 — The original Chilibot query results of the term "long-term potentiation (LTP)" and 22 other terms, limiting the latest references analyzed to the years 1990, 1995, 2000, and 2004. [file 1471-2105-5-147-S5.bz2 › chilibotAdditionalFile5/ltp1995/html/TRKB.html]

 


**TRKB** (Input: TRKB ) 

---


|  |
| --- |
| **Google Searches:** Entire Web  | EDU domain only  | PDF files only |

.

|  |
| --- |
| **External Links:** OMIM | LocusLink | Swissprot | GeneCards |

  
**Maps of TRKB**

|  |
| --- |
| Simple Complete graph in radiant tree square layout. |

**New Hypothesis !**

|  |
| --- |
|  |

**Synonyms** 

|  |
| --- |
| - trk b   [PubMed] |
| - trkb   [PubMed] |

**Synopsis**

|  |
| --- |
| - These results suggest that two different pathways, the c ras and the PLC gamma pathway, are activated by **TrkB** receptors in primary neurons.  J Neurochem, 1995    [23] |
| - These results show that NT3 can signal through TrkA and **TrkB** in neurons at certain stages of development and may explain why the phenotype of NT3 mice is more severe than that of trkC mice.  EMBO J, 1995    [20] |
| - The invariant neurotrophin messenger RNA levels, and the changing expression of trk B  [**TRKB**]  and trk C during ontogeny, suggest that trophic regulation of glial development is primarily governed through modulation of receptor expression.  Neuroscience, 1995    [16] |
| - In the inner ear, **TrkB** and TrkC dependent neurons were shown to at least partially depend on each other for survival, most likely indirectly due to abnormal development of their common targets.  Development, 1995    [15] |
| - No immunolabelling for neuronal **trkB** like proteins was observed, but **trkB** and trkC like proteins IR was found in non neuronal cells.  Mech Ageing Dev, 1995    [15] |
| - At P5, the innermost part of the inner nuclear layer INL expressed TrkA, **TrkB** and p75 mRNAs.  Brain Res, 1995    [14] |
| - trkC mRNA is expressed first, followed by **trkB** mRNA and finally trkA mRNA.  Neuroreport, 1995    [14] |
| - **TrkB** was present at the mRNA as well as protein level only in its truncated form.  Eur J Neurosci, 1995    [13] |
| - andhigh levels of trkC mRNA, and relatively high amount of **trkB** mRNA, while levels of trkA mRNA was undetectable.  Neuroreport, 1995    [13] |
| - Taken together, the results indicate the existence in postnatal rat brain of a large overlapping population of cholinergic neurons that are responsive to ligands for the neurotrophin receptors TrkA and **TrkB**,   Brain Res, 1995    [11] |
| - The present study demonstrates increased BDNF IR in the mossy fiber terminal zone of hippocampus after exposure to KA, as well as an increase in **trkB** mRNA, and provides evidence of local release of this neurotrophin into the surrounding neuropil where it would be available for local utilization.  J Neurosci, 1994    [11] |
| - tuberoinfundibular DA neurons do NOT express **trkB** mRNA, but some have trkC mRNA, which encodes the receptor for neurotrophin 3 .  J Neurosci, 1995    [11] |
| - Thus, astroglial cells in culture internalize NGF through a specific receptor mediated process, express trkA and full length **trkB** mRNAs at low levels, and respond to exogenous NGF by expressing a fibrous morphology under serum free culture conditions.  J Neurosci Res, 1995    [11] |
| - Cycloheximide had no effect on **trkB** mRNA expression but attenuated the injury induced increase in trkC mRNA expression in dentate gyrus at 4 h by 75%.  Neuroreport, 1995    [11] |
| - In addition, diabetes induced a reduction in the expression levels of the neurotrophin receptors **trkB** mRNA decreased by 50% after 6 weeks of diabetes, but returned to control levels after 12 weeks.  Neurosci Lett, 1995    [11] |
