# Supplementary material for: Content-rich biological network constructed by mining PubMed abstracts
Source: BMC Bioinformatics. 2004 Oct 8;5:147. doi: 10.1186/1471-2105-5-147 (PMC528731; doi:10.1186/1471-2105-5-147)
Supplement: Additional File 5 — The original Chilibot query results of the term "long-term potentiation (LTP)" and 22 other terms, limiting the latest references analyzed to the years 1990, 1995, 2000, and 2004. [file 1471-2105-5-147-S5.bz2 › chilibotAdditionalFile5/ltp1995/html/ERK.html]

 


**ERK** (Input: ERK ) 

---


|  |
| --- |
| **Google Searches:** Entire Web  | EDU domain only  | PDF files only |

.

|  |
| --- |
| **External Links:** OMIM | LocusLink | Swissprot | GeneCards |

  
**Maps of ERK**

|  |
| --- |
| Simple Complete graph in radiant tree square layout. |

**New Hypothesis !**

|  |
| --- |
|  |

**Synonyms** 

|  |
| --- |
| - erk   [PubMed] |

**Synopsis**

|  |
| --- |
| - Both cortical and brainstem type Lewy bodies in diffuse Lewy body disease and brainstem type Lewy bodies in Parkinson s disease were found to be immunoreactive for cdk5  Am J Pathol, 1995    [18] |
| - The present study indicates that the bradykinin stimulated **ERK** 2 pathway is entirely cyclic AMP sensitive, and suggests that coincident signal detection by adenylate cyclase may be an important physiological route for the modulation of early mitogenic signalling.  Biochem J, 1995    [16] |
| - The results support the hypothesis that mos acts through the MAP kinase cascade MKK 1 and **ERK** 2 to induce cell transformation.  Cell Growth Differ, 1995    [14] |
| - Unlike known TPA resistant cells whose resistance is mainly due to lack or down modulation of protein kinase C, UT16 cells showed TPA induced activation of PKC, Raf 1, and **ERK** MAP kinases similar to the parental U937 cells.  Oncogene, 1995    [13] |
| - Injury to hypoglossal motor neurons resulted in an increase in extracellular regulated kinase **ERK**, or MAP kinase and **ERK** kinase MEK, or MAP kinase kinase mRNAs, but in a decrease in the expression of the catalytic subunits of PKA C alpha and C beta mRNAs.  Brain Res Mol Brain Res, 1995    [11] |
| - The results obtained with the Raf 1 AS oligonucleotide indicate that this serine threonine kinase is dispensable for **ERK** kinase activation, but needed for the PBu2 mitogenic signaling even as late as 7 h after the delivery of the signal.  Eur J Immunol, 1994    [11] |
| - The tyrosine kinase inhibitors herbimycin A or genistein blocked both the accumulation of 19 out of 20 signal transduction molecules and JNK and **ERK** mediated signaling.  J Cell Biol, 1995    [10] |
| - andPI 3 kinase  [PI-3K]  and **ERK** activation.  Neuron, 1995    [10] |
| - In most eukaryotes, Ras functions as a positive regulator of an **ERK** MAPK signal transduction cascade through the activation of a MEKK.  FASEB J, 1995    [10] |
| - We conclude that PKC and EGF act through parallel pathways to stimulate **ERK** phosphorylation and activity.  J Cell Physiol, 1995    [10] |
| - AMPA,   J Biol Chem, 1995    [10] |
| - Regulationof the MAP kinase cascade in PC12 cells B Raf activates MEK 1 MAP kinase or **ERK** kinase and is inhibited by cAMP.  FEBS Lett, 1995    [10] |
| - We explored the neuroanatomic distribution of **ERK** immunoreactivity in the rhesus monkey brain.  Neurosci Lett, 1994    [10] |
| - raf and **Erk** evidenced decreased mobility consistent with an activated state in JHLa1,   Virology, 1995    [10] |
| - Fas APO 1 cross linking in these clones fails to activate PC PLC and aSMase, while nSMase, **ERK** 2 and PLA2 activates are induced.  EMBO J, 1995    [10] |
