# Supplementary material for: Content-rich biological network constructed by mining PubMed abstracts
Source: BMC Bioinformatics. 2004 Oct 8;5:147. doi: 10.1186/1471-2105-5-147 (PMC528731; doi:10.1186/1471-2105-5-147)
Supplement: Additional File 5 — The original Chilibot query results of the term "long-term potentiation (LTP)" and 22 other terms, limiting the latest references analyzed to the years 1990, 1995, 2000, and 2004. [file 1471-2105-5-147-S5.bz2 › chilibotAdditionalFile5/ltp1995/html/CREB.html]

 


**CREB** (Input: CREB ) 

---


|  |
| --- |
| **Google Searches:** Entire Web  | EDU domain only  | PDF files only |

.

|  |
| --- |
| **External Links:** OMIM | LocusLink | Swissprot | GeneCards |

  
**Maps of CREB**

|  |
| --- |
| Simple Complete graph in radiant tree square layout. |

**New Hypothesis !**

|  |
| --- |
|  |

**Synonyms** 

|  |
| --- |
| - creb   [PubMed] |

**Synopsis**

|  |
| --- |
| - **CREB** is a hormonally stimulated transcriptional activator, and CaM kinase may lie on the pathway to its activation.  Annu Rev Biochem, 1992    [28] |
| - These results suggest that the phosphorylation and dephosphorylation of **CREB** controls its ability to regulate transcription in membrane depolarized cells.  J Biol Chem, 1995    [25] |
| - Taken together with PKC mediation of **CREB** Ser133 phosphorylation in B cells, these results suggest that the dominant mode of **CREB** regulation is cell type specific.  J Immunol, 1995    [23] |
| - These findings indicate that dephosphorylated **CREB** is a negative regulator of C EBP activated transcription of the somatostatin gene promoter in RIN 1027 B2 cells.  Mol Cell Biol, 1995    [22] |
| - Genetic approaches have established that the transcription factor **CREB** is essential for long term memory.  Curr Opin Neurobiol, 1995    [17] |
| - Experiments with a series of simplified reporter genes in combination with specific recombinant protein kinase inhibitors suggest that induction of gene activity following NO amplified calcium action involves protein kinase A dependent activation of the transcription factor **CREB**.  Nature, 1993    [16] |
| - However, this paper shows that 12 O tetradecanoylphorbol 13 acetate TPA which activates PKC also leads to the phosphorylation of **CREB** in oPT cells, suggesting the potential involvement of other signal transduction pathways in the transcriptional regulation of these cells.  J Neuroendocrinol, 1994    [16] |
| - These observations suggest that Tax enhances **CREB** mediated transactivation of the HTLV I promoter by a mechanism apart from, and or in addition to, the reported stabilization of DNA binding by interaction with the bZIP domain of **CREB**.  FEBS Lett, 1995    [16] |
| - In summary, PKA signaling and transacting factors such as **CREB**, Fos and Jun are probably involved in transcriptional inhibition of GnRH gene by hCG in GT1 7 neurons.  Mol Cell Endocrinol, 1995    [16] |
| - These results implicate **CREB** dependent transcription in mammalian long term memory.  Cell, 1994    [14] |
| - The data indicate that **CREB** activity in the developing palate is most likely to be regulated at the level of protein phosphorylation as opposed to changes in levels of **CREB** protein expression.  J Cell Physiol, 1995    [11] |
| - We propose that the hierarchical phosphorylation at the PKA and GSK 3 sites of **CREB** are essential for cAMP control of **CREB**.  J Biol Chem, 1994    [10] |
| - Recombinant ATF 1 and **CREB** 1 proteins bound HIF1 probes either as homodimers or as heterodimers, indicating a new binding specificity for ATF 1 **CREB** 1.  Nucleic Acids Res, 1995    [10] |
| - ThecAMP responsive element CRE modulator protein CREM alpha has been proposed to be a negative regulator of the CRE binding protein **CREB** .  J Biol Chem, 1994    [10] |
| - The presence and activity of PKC, PKA, and P **CREB** in developing chicken skin are further characterized by immunoblot, kinase activity, and gel shift assays.  Dev Biol, 1995    [10] |
