# Supplementary material for: Content-rich biological network constructed by mining PubMed abstracts
Source: BMC Bioinformatics. 2004 Oct 8;5:147. doi: 10.1186/1471-2105-5-147 (PMC528731; doi:10.1186/1471-2105-5-147)
Supplement: Additional File 5 — The original Chilibot query results of the term "long-term potentiation (LTP)" and 22 other terms, limiting the latest references analyzed to the years 1990, 1995, 2000, and 2004. [file 1471-2105-5-147-S5.bz2 › chilibotAdditionalFile5/ltp1995/html/PI-3K.html]

 


**PI-3K** (Input: PI-3K ) 

---


|  |
| --- |
| **Google Searches:** Entire Web  | EDU domain only  | PDF files only |

.

|  |
| --- |
| **External Links:** OMIM | LocusLink | Swissprot | GeneCards |

  
**Maps of PI-3K**

|  |
| --- |
| Simple Complete graph in radiant tree square layout. |

**New Hypothesis !**

|  |
| --- |
|  |

**Synonyms** 

|  |
| --- |
| - pi-3k   [PubMed] |
| - pi-3-kinase   [PubMed] |
| - pi3-kinase   [PubMed] |

**Synopsis**

|  |
| --- |
| - Taken together, these results suggest that a PI 3 kinase  [**PI-3K**]  activation induced by FcR ligation is functionally coupled to granule exocytosis and ADCC.  J Exp Med, 1994    [23] |
| - Thus, these results show important functional differences between the alpha and beta isoforms of p85 in vivo and point to c cbl as a potentially important mediator of some of the functions of PI 3 kinase  [**PI-3K**]  in intact cells.  J Biol Chem, 1995    [20] |
| - These results suggest that lyn plays an important role in CD40 mediated PTK activation and identify PLC gamma 2 and PI 3 kinase  [**PI-3K**]  targets for CD40 mediated phosphorylation, suggesting a role for these two enzymes in CD40 signal transduction.  J Exp Med, 1994    [20] |
| - These findings suggest that the sequential activation of PI 3 kinase  [**PI-3K**] , Ras protein, and MAP kinase is involved in the insulin signaling pathway s during differentiation by hormones IBMX and in consequence of the inhibition of PI 3 kinase  [**PI-3K**]  by wortmannin, the activation of Ras protein and MAP kinase which acts downstream of PI 3 kinase  [**PI-3K**]  is suppressed and results in the inhibition of adipocyte differentiation.  Biochem Biophys Res Commun, 1995    [20] |
| - These results suggest that wortmannin has a potent inhibitory effect on PI 3 kinase  [**PI-3K**]  and a weak inhibitory effect on PKC activity, and both effects cause a significant inhibition of insulin stimulated glucose uptake in rat adipocytes.  Diabetes Res Clin Pract, 1995    [20] |
| - These studies suggest a role of PI 3 kinase  [**PI-3K**]  in the 5 HT induced potentiation of epinephrine mediated platelet aggregation.  Res Commun Mol Pathol Pharmacol, 1995    [19] |
| - PI 3 kinase  [**PI-3K**]  has been shown, like Raf, to be a direct effector of Ras.  J Biol Chem, 1994    [19] |
| - The results suggest that wortmannin sensitive molecules such as PI3 kinase  [**PI-3K**] , are involved in the V1 receptor mediated activation of the MAP kinase pathway independent of TPA sensitive PKC.  FEBS Lett, 1995    [17] |
| - As the three dimensional structures of the phosphatidylinositol PI 3 kinase  [**PI-3K**] , Lck, Src and Abl SH2 domains have been shown to be similar, we have modelled other SH2 domains that show distinct sequence specificity to allow comparative analysis of SH2 phosphopeptide interactions.  Protein Eng, 1995    [16] |
| - The data further dissociate activation of PI 3 kinase  [**PI-3K**]  from mitogenesis in human mesangial cells.  Kidney Int, 1994    [14] |
| - These results indicate that tyrosine phosphorylation of PLC gamma 1, GAP, and PI 3 kinase  [**PI-3K**]  are specific responses for VSMC hyperplasia  Am J Physiol, 1994    [14] |
| - These results are most consistent with the interpretation that PI 3 kinase  [**PI-3K**]  regulates the trafficking of lysosomal enzymes by interfering with a M6PR dependent sorting event in the TGN.  J Cell Biol, 1995    [11] |
| - 2insulin stimulated increase in PI 3 kinase  [**PI-3K**]  activity associated with insulin receptor substrate 1.  Mol Endocrinol, 1994    [10] |
| - In conclusion, SCF induced PI 3 kinase  [**PI-3K**]  activation paralleled the increased SCF induced mitogenicity after inhibition of PKC.  J Biol Chem, 1994    [10] |
| - Like the inhibition of GSK3 refs 1 4, the activation of PKB is prevented by inhibitors of phosphatidylinositol PI 3 kinase  [**PI-3K**] .  NatureNature, 1994    [10] |
