# Supplementary material for: Content-rich biological network constructed by mining PubMed abstracts
Source: BMC Bioinformatics. 2004 Oct 8;5:147. doi: 10.1186/1471-2105-5-147 (PMC528731; doi:10.1186/1471-2105-5-147)
Supplement: Additional File 5 — The original Chilibot query results of the term "long-term potentiation (LTP)" and 22 other terms, limiting the latest references analyzed to the years 1990, 1995, 2000, and 2004. [file 1471-2105-5-147-S5.bz2 › chilibotAdditionalFile5/ltp1995/html/PKC.html]

 


**PKC** (Input: PKC ) 

---


|  |
| --- |
| **Google Searches:** Entire Web  | EDU domain only  | PDF files only |

.

|  |
| --- |
| **External Links:** OMIM | LocusLink | Swissprot | GeneCards |

  
**Maps of PKC**

|  |
| --- |
| Simple Complete graph in radiant tree square layout. |

**New Hypothesis !**

|  |
| --- |
|  |

**Synonyms** 

|  |
| --- |
| - pkc   [PubMed] |

**Synopsis**

|  |
| --- |
| - As the epsilon **PKC** could be activated by arachidonic acid, it is strongly suggested that in the case of LTP, the presynaptic epsilon **PKC** is activated by arachidonic acid released postsynaptically and phosphorylates GAP 43, resulting in the increase in glutamate release.  Nippon Yakurigaku Zasshi, 1995    [29] |
| - The data suggest that a membrane insertion of alpha beta **PKC** is NOT a prerequisite for the LTP induced increased phosphorylation of **PKC** substrates.  Brain Res, 1995    [27] |
| - These data suggest different physiological roles of calcium dependent **PKC** isoenzymes in activity dependent synaptic plasticity.  Neuroreport, 1994    [25] |
| - The present findings, together with previous data reporting a similar temporal course in the effects of intrahippocampal or intraamygdala infusion of specific **PKC** inhibitors on memory, suggest that **PKC** activation plays a role in the acquisition and consolidation of an inhibitory avoidance learning.  Brain Res, 1995    [22] |
| - This hypothesis is confirmed by the experiments in which LTP like phenomena for early and late cortical IPSPs were shown to be the result of inactivation of PKA and **PKC**.  Zh Vyssh Nerv Deiat Im I P PavlovaZh Vyssh Nerv Deiat Im I P Pavlova, 1995    [22] |
| - These findings suggest that  calcium influx through voltage gated channels and co activation of **PKC** by mGluRs are both necessary for induction of LTPk.  Hippocampus, 1994    [19] |
| - Moreover, these animals show area specific changes in the phosphorylation state of the protein B 50 GAP 43, a well characterized neuron specific substrate for **PKC**.  NeurotoxicologyNeurotoxicology, 1994    [19] |
| - One way to investigate the role of **PKC** in long term potentiation  [LTP]  is to determine the degree of phosphorylation of its substrates after in situ phosphorylation in hippocampal slices.  Behav Brain Res, 1995    [16] |
| - The role of several biological molecules in learning and memory are considered, for example, protein kinase C **PKC**, Ca Calmodulin kinase II CaMKII, GAP 43, and glutamate receptors.  Mol Cell BiochemMol Cell Biochem, 1995    [16] |
| - The present study employs the principles of component task analysis to examine the role of membrane associated **PKC** mPKC in hippocampal dependent memory when all factors other than the type of learning were equivalent.  Brain Res, 1995    [15] |
| - These results indicate that arachidonic acid may contribute to LTP maintenance by activation of presynaptic **PKC** and phosphorylation of GAP 43 substrate.  J Neurochem, 1995    [14] |
| - The results confirm that activity driven retinotopic sharpening is very sensitive to manipulations of kinases, especially **PKC**.  J Neurobiol, 1994    [14] |
| - The results are consistent with the view that **PKC** activity may be implicated in a pre long term stage of memory processing.  Behav Brain Res, 1994    [14] |
| - functionalcross talk between calcium CaM and **PKC** pathways occurs during the induction of LTP.  Neuron, 1995    [12] |
| - Of the **PKC** isoforms, only gamma **PKC** was up regulated substantially 2 hr after LTP induction, declining to control levels 48 hr later.  Neuron, 1994    [11] |
