# Supplementary material for: Content-rich biological network constructed by mining PubMed abstracts
Source: BMC Bioinformatics. 2004 Oct 8;5:147. doi: 10.1186/1471-2105-5-147 (PMC528731; doi:10.1186/1471-2105-5-147)
Supplement: Additional File 5 — The original Chilibot query results of the term "long-term potentiation (LTP)" and 22 other terms, limiting the latest references analyzed to the years 1990, 1995, 2000, and 2004. [file 1471-2105-5-147-S5.bz2 › chilibotAdditionalFile5/ltp1995/html/PKA.html]

 


**PKA** (Input: PKA ) 

---


|  |
| --- |
| **Google Searches:** Entire Web  | EDU domain only  | PDF files only |

.

|  |
| --- |
| **External Links:** OMIM | LocusLink | Swissprot | GeneCards |

  
**Maps of PKA**

|  |
| --- |
| Simple Complete graph in radiant tree square layout. |

**New Hypothesis !**

|  |
| --- |
|  |

**Synonyms** 

|  |
| --- |
| - pka   [PubMed] |

**Synopsis**

|  |
| --- |
| - These data suggest that besides protein kinase C the synergistic activation of **PKA** is necessary for the maintenance of LTP.  Neuroreport, 1993    [30] |
| - These results suggest that **PKA** mediated phosphorylation of PLC may regulate TCR CD3 induced InsPL hydrolysis.  Biochem J, 1992    [23] |
| - These results suggest that CD3 stimulation activates PIP2 hydrolysis by inducing tyrosine phosphorylation of PLC gamma 1, which is regulated negatively by PKC and **PKA**.  J Biol Chem, 1991    [23] |
| - This hypothesis is confirmed by the experiments in which LTP like phenomena for early and late cortical IPSPs were shown to be the result of inactivation of **PKA** and PKC.  Zh Vyssh Nerv Deiat Im I P PavlovaZh Vyssh Nerv Deiat Im I P Pavlova, 1991    [22] |
| - These results are consistent with the hypothesis that the developmental changes in phosphorylation of endogenous substrates by **PKA** is controlled largely by changes in the concentration of those substrates.  Neurochem Res, 1991    [20] |
| - Finally, reporter gene assays in HeLa cells treated with either a cAMP analogue or a phorbol ester suggest that the **PKA**,  Nucleic Acids Res, 1995    [19] |
| - Phosphorylation of one or more membrane associated proteins by **PKA** may regulate myometrial PLC activity and play a role in the inhibitory effects of isoproterenol and relaxin.  Endocrinology, 1992    [19] |
| - Ablation by gene targeting of the C beta 1 or the RI beta isoform of **PKA** produces a selective defect in mossy fiber LTP, providing genetic evidence for the role of these isoforms in the mossy fiber pathway.  Cell, 1995    [18] |
| - Since the late phase of CA1 LTP requires **PKA** but is normal in RI beta mutant mice, our data further suggest that different forms of synaptic plasticity are likely to employ different combinations of regulatory and catalytic subunits.  Proc Natl Acad Sci U S A, 1995    [17] |
| - Concentrations of H8 N 2 methylamino ethyl 5 isoquinoline sulfonamide, diHCl inhibitory to cAMP dependent **PKA** prevented the inhibitory effect of forskolin on surface IgM mediated calcium response, suggesting that cAMP exerted its effects through **PKA**.  J Immunol, 1991    [16] |
| - In summary, **PKA** signaling and transacting factors such as CREB, Fos and Jun are probably involved in transcriptional inhibition of GnRH gene by hCG in GT1 7 neurons.  Mol Cell Endocrinol, 1995    [16] |
| - Inhibitors of the NGF activated protein kinase N PKN were found to partially and in some cases transiently block VGF induction by NGF while in **PKA** deficient PC12 cells, VGF induction by NGF was comparable to that observed in parental PC12 cells.  FEBS Lett, 1995    [15] |
| - These results imply that H ras functions, in this system, to decrease levels of cAMP, thus negating the regulatory effect of **PKA** on PLC.  Biochem Biophys Res Commun, 1994    [14] |
| - Cross talk between **PKA** signaling and tyrosine kinase receptor signaling results in **PKA** inhibition of the MAP kinase cascade, probably at the level of Raf.  Ann N Y Acad Sci, 1995    [14] |
| - Site specific mutagenesis of the serine residue representing a **PKA** consensus site completely eliminates **PKA** mediated phosphorylation of this site as well as the potentiation of the glutamate response.  Nature, 1993    [12] |
