# Supplementary material for: Content-rich biological network constructed by mining PubMed abstracts
Source: BMC Bioinformatics. 2004 Oct 8;5:147. doi: 10.1186/1471-2105-5-147 (PMC528731; doi:10.1186/1471-2105-5-147)
Supplement: Additional File 5 — The original Chilibot query results of the term "long-term potentiation (LTP)" and 22 other terms, limiting the latest references analyzed to the years 1990, 1995, 2000, and 2004. [file 1471-2105-5-147-S5.bz2 › chilibotAdditionalFile5/ltp1995/html/NMDA.html]

 


**NMDA** (Input: NMDA ) 

---


|  |
| --- |
| **Google Searches:** Entire Web  | EDU domain only  | PDF files only |

.

|  |
| --- |
| **External Links:** OMIM | LocusLink | Swissprot | GeneCards |

  
**Maps of NMDA**

|  |
| --- |
| Simple Complete graph in radiant tree square layout. |

**New Hypothesis !**

|  |
| --- |
|  |

**Synonyms** 

|  |
| --- |
| - nmda   [PubMed] |

**Synopsis**

|  |
| --- |
| - These results suggest that NO formation secondary to calcium influx by **NMDA** receptor activation leads to similar modifications of PLC delta to those seen in AD.  J Neurosci Res, 1995    [23] |
| - These results suggest that glutamate can activate CaM kinase II through **NMDA** receptors in the induction of LTP and in turn stimulates the phosphorylation of target proteins such as MAP2 and synapsin I.  Nippon Yakurigaku Zasshi, 1993    [22] |
| - We suggest that AMPA and **NMDA** components are potentiated through two different presumably postsynaptic processes.  J Neurophysiol, 1995    [21] |
| - These results suggest that glutamate can activate CaM kinase II through the ionotropic **NMDA** receptor, which in turn increases the phosphorylation of microtuble associated protein 2 and synapsin I.  J Biol Chem, 1992    [20] |
| - They further support the assumption of the essential role of the **NMDA** R1 receptor subunit in the induction of LTP.  Neurosci Lett, 1995    [19] |
| - Finally, we found that the expression of E S potentiation did NOT require the activation of **NMDA** receptors.  J Neurosci, 1995    [16] |
| - As suggested by studies of long term potentiation  [LTP] , **NMDA** receptor dependent postsynaptic calcium appears to be essential for the development of these storage sites and indeed to trigger their development.  Pharmacol Biochem Behav, 1995    [16] |
| - Recentwork has suggested that some proportion of excitatory synapses on hippocampal CA1 pyramidal cells that express **NMDA** receptors NMDARs may NOT express functional AMPA receptors AMPARs, thus making these synapses silent at the resting membrane potential.  Neuron, 1995    [15] |
| - Because **NMDA** receptor stimulation amplifies glutamate release, our data are consistent with presynaptic A1 receptor mediated inhibition of EAA release and consequent downregulation of NO production.  Stroke, 1995    [14] |
| - Post 2 DG long term potentiation  [LTP]  2 DG LTP is prevented by block of N methyl D aspartate **NMDA** receptors NMDARs .  J Neurophysiol, 1995    [12] |
| - When slices were superfused with DL 2 amino 5 phosphonovaleric acid an N methyl D aspartate, **NMDA**, antagonist and 6 cyano 7 nitroquinoxaline dione a non **NMDA** glutamate antagonist, the potentiation of the monosynaptic fast IPSP could still be induced and maintained, suggesting that polysynaptic influences were unnecessary for this process.  Can J Physiol Pharmacol, 1995    [12] |
| - The synaptic modifications during theta require **NMDA** receptors and muscarinic receptors.  Neuron, 1995    [12] |
| - Widely spaced trains of high frequency stimulation generated cAMP postsynaptically via **NMDA** receptors and calmodulin, consistent with the calcium calmodulin mediated stimulation of postsynaptic adenylyl cyclase.  Neuron, 1995    [12] |
| - Ethanol applied before  Brain Res, 1995    [10] |
| - ACPD failed to induce LTP of pharmacologically isolated **NMDA** receptor mediated EPSPs.  Neuropharmacology, 1995    [10] |
