# Supplementary material for: Content-rich biological network constructed by mining PubMed abstracts
Source: BMC Bioinformatics. 2004 Oct 8;5:147. doi: 10.1186/1471-2105-5-147 (PMC528731; doi:10.1186/1471-2105-5-147)
Supplement: Additional File 5 — The original Chilibot query results of the term "long-term potentiation (LTP)" and 22 other terms, limiting the latest references analyzed to the years 1990, 1995, 2000, and 2004. [file 1471-2105-5-147-S5.bz2 › chilibotAdditionalFile5/ltp1995/html/AMPA.html]

 


**AMPA** (Input: AMPA ) 

---


|  |
| --- |
| **Google Searches:** Entire Web  | EDU domain only  | PDF files only |

.

|  |
| --- |
| **External Links:** OMIM | LocusLink | Swissprot | GeneCards |

  
**Maps of AMPA**

|  |
| --- |
| Simple Complete graph in radiant tree square layout. |

**New Hypothesis !**

|  |
| --- |
|  |

**Synonyms** 

|  |
| --- |
| - ampa   [PubMed] |

**Synopsis**

|  |
| --- |
| - These data suggest that GYKI 52466 and cyclothiazide probably mediate their effects on the **AMPA** receptor via different binding sites.  Neurosci Lett, 1994    [23] |
| - Considered together, these data suggest that ACPD induced LTP is due to a direct increase in the **AMPA** receptor mediated synaptic conductance and involves postsynaptic induction and expression mechanisms.  Neuropharmacology, 1995    [20] |
| - These data suggest that in this chronic model of epileptiform activity, there is long term potentiation  [LTP]  of excitatory mediated events regulated primarily by **AMPA** receptors.  Epilepsy Res, 1995    [20] |
| - We suggest that **AMPA** and NMDA components are potentiated through two different presumably postsynaptic processes.  J Neurophysiol, 1995    [19] |
| - This suggests that tetanic stimulation produced LTP of **AMPA** and LTD of NMDA receptor mediated responses simultaneously.  Eur J Neurosci, 1995    [19] |
| - The possible clinical usefulness of **AMPA** receptor antagonists as antiepileptic drugs is suggested.  Brain Res, 1995    [19] |
| - Tetanic stimulation in control media evoked a statistically identical long term potentiation  [LTP]  LTP of both the **AMPA** and NMDA receptor mediated components of the dual component EPSC AM PAR and NMDAR EPSCs, as shown by a similar percentage increase in both components when measured at a holding potential of 30 mV, and by an identical time course of the pre and post LTP induced EPSC at 30 mV and 70 mV.  J Neurosci, 1995    [16] |
| - The role of these different modulatory sites of the NMDA receptor and their relation with LTP are reviewed with a particular attention to the redox site which seems to be a selective target to distinguish between **AMPA** and NMDA LTP.  J Neurobiol, 1995    [16] |
| - these studies have suggested that mossy fibre synapses activate primarily or exclusively alpha amino 3 hydroxy 5 methyl 4 isoxazolepropionic acid **AMPA** receptors and, indeed, these synapses express a form of LTP that is entirely independent of NMDA receptors.  Nature, 1995    [15] |
| - Recentwork has suggested that some proportion of excitatory synapses on hippocampal CA1 pyramidal cells that express NMDA receptors NMDARs may NOT express functional **AMPA** receptors AMPARs, thus making these synapses silent at the resting membrane potential.  Neuron, 1995    [15] |
| - The results indicate that activation of endogenous phospholipases may be an important mechanism in the regulation of **AMPA** receptor properties in LTP.  Brain Res, 1995    [14] |
| - These data are consistent with the hypothesis that the movement of **AMPA** receptors between cellular compartments in the postsynaptic neurone could constitute one mechanism underlying long term potentiation  [LTP]  in the hippocampus.  Br J Pharmacol, 1995    [13] |
| - At the time of expression, memory and LTP are blocked by antagonists of glutamate **AMPA** receptors and are accompanied by an enhanced sensitivity of these receptors.  Neurobiol Learn Mem, 1995    [12] |
| - This may explain increased KCl and **AMPA** induced InsP1 accumulation whereas receptor coupled PLC activation is less affected.  Brain Res, 1993    [10] |
| - This leads to an increase in protein phosphatase and decrease in protein kinases concentrations and cause dephosphorylation of ionotropic **AMPA**, NMDA, and GABAa receptors.  Zh Vyssh Nerv Deiat Im I P PavlovaZh Vyssh Nerv Deiat Im I P Pavlova, 1993    [10] |
