# Supplementary material for: Content-rich biological network constructed by mining PubMed abstracts
Source: BMC Bioinformatics. 2004 Oct 8;5:147. doi: 10.1186/1471-2105-5-147 (PMC528731; doi:10.1186/1471-2105-5-147)
Supplement: Additional File 5 — The original Chilibot query results of the term "long-term potentiation (LTP)" and 22 other terms, limiting the latest references analyzed to the years 1990, 1995, 2000, and 2004. [file 1471-2105-5-147-S5.bz2 › chilibotAdditionalFile5/ltp1995/html/TAU.html]

 


**TAU** (Input: TAU ) 

---


|  |
| --- |
| **Google Searches:** Entire Web  | EDU domain only  | PDF files only |

.

|  |
| --- |
| **External Links:** OMIM | LocusLink | Swissprot | GeneCards |

  
**Maps of TAU**

|  |
| --- |
| Simple Complete graph in radiant tree square layout. |

**New Hypothesis !**

|  |
| --- |
|  |

**Synonyms** 

|  |
| --- |
| - tau   [PubMed] |

**Synopsis**

|  |
| --- |
| - Antibodies against microtubule associated protein **Tau**, which is expressed in developing neurites, showed that T3 enhanced neurite formation.  Neuroreport, 1992    [19] |
| - This is the first animal model in which abnormal phosphorylation of **Tau** has been shown to be produced experimentally in vivo.  Brain Res, 1995    [19] |
| - A role for ERKs has been postulated in Alzheimer s disease, where they have been implicated in phosphorylation of **tau** in neurofibrillary tangles.  Neurosci Lett, 1994    [19] |
| - Furthermore, MAP 2 and **tau** protein showed a development dependent change in expression from the juvenile toward the adult form.  J Neurochem, 1992    [19] |
| - Both cortical and brainstem type Lewy bodies in diffuse Lewy body disease and brainstem type Lewy bodies in Parkinson s disease were found to be immunoreactive for cdk5  Am J Pathol, 1995    [18] |
| - The enzyme phosphorylated smooth muscle myosin light chain, synapsin I, microtubule associated protein 2, **tau** protein, myelin basic protein, histone H1, and tyrosine hydroxylase in a calcium calmodulin dependent manner, suggesting that the enzyme is a multifunctional calmodulin dependent protein kinase capable of phosphorylating a large number of substrates.  J Biol Chem, 1992    [16] |
| - In addition, using permeabilized lymphoma cells, we have found that 1 GTP or GTP **tau** S augments, and pertussis toxin inhibits, phospholipase C PLC activity and receptor capping.  J Immunol, 1990    [16] |
| - During postnatal development, the intensity of the phosphate dependent staining decreased, suggesting that phosphorylation of **tau** proteins in perikarya and dendrites may be essential for early steps in neuronal morphogenesis during cat cerebellum development.  Brain Res Bull, 1994    [16] |
| - MAPK immunoreactivity is present in the same neurons as NFT and in the same subcellular compartments as **tau**, supporting a role for MAPKs in **tau** phosphorylation in Alzheimer s disease.  Am J Pathol, 1994    [16] |
| - Phalloidin staining and immunohistochemistry showed that the neuroblast was richer in F actin, beta tubulin, MAP1, MAP2, **tau**, calspectin, and synapsin I than the matrix cell.  Arch Histol Cytol, 1992    [15] |
| - These results prove that the type I, II, and III PKC are products of PKC genes, **tau**, beta, and alpha, respectively.  Biochem Biophys Res Commun, 1987    [14] |
| - These findings suggest that PLC **tau**, and perhaps the 76 kDa co precipitated protein, are substrates of cyclic AMP dependent protein kinase in BALB c 3T3 cells however, the lack of effect of cyclic AMP elevation on PDGF stimulated inositol phosphate formation indicates that the intrinsic activity of PLC **tau** is unaltered by cyclic AMP mediated phosphorylation.  Biochem J, 1990    [13] |
| - Western blot analysis showed no major changes in pituitary PKC alpha, PKC beta and PKC zeta when 6 day old and 3 month old female rats were compared, while PKC **tau** was NOT detected.  Mol Cell Endocrinol, 1994    [12] |
| - Cholinergic neurons are the main pharmacologic target but there are many other ones GABA ergic system, **Tau** protein, amyloid.  EncephaleEncephale, 1994    [10] |
| - The site is likely to be phosphorylated in **tau** from Alzheimer neurofibrillary tangles.  EMBO J, 1990    [10] |
