# Supplementary material for: Content-rich biological network constructed by mining PubMed abstracts
Source: BMC Bioinformatics. 2004 Oct 8;5:147. doi: 10.1186/1471-2105-5-147 (PMC528731; doi:10.1186/1471-2105-5-147)
Supplement: Additional File 5 — The original Chilibot query results of the term "long-term potentiation (LTP)" and 22 other terms, limiting the latest references analyzed to the years 1990, 1995, 2000, and 2004. [file 1471-2105-5-147-S5.bz2 › chilibotAdditionalFile5/ltp1995/html/ARC.html]

 


**ARC** (Input: ARC ) 

---


|  |
| --- |
| **Google Searches:** Entire Web  | EDU domain only  | PDF files only |

.

|  |
| --- |
| **External Links:** OMIM | LocusLink | Swissprot | GeneCards |

  
**Maps of ARC**

|  |
| --- |
| Simple Complete graph in radiant tree square layout. |

**New Hypothesis !**

|  |
| --- |
|  |

**Synonyms** 

|  |
| --- |
| - arc   [PubMed] |

**Synopsis**

|  |
| --- |
| - The data suggests that activation of the Bezold Jarisch reflex by 5 HT involves a glutamatergic synapse presumably located within the brainstem vagal reflex **arc**.  Neuropharmacology, 1987    [23] |
| - Similar experiments with constructs containing alpha cardiac, alpha smooth muscle and gamma cytoplasmic actins have shown that expression of epitope tagged actins in **ARC** result in different epitope staining patterns.  Symp Soc Exp Biol, 1992    [20] |
| - Our observations suggest that **Arc** may play a role in activity dependent plasticity of dendrites.  Neuron, 1995    [19] |
| - In addition to actin, the **ARC** was enriched with proteins that showed cross reactivity to antibodies to alpha actinin and the 50K actin binding protein elongation factor 1 alpha from Dictyostelium.  Arch Med Res, 1992    [17] |
| - Comparison of these results with those in the cat suggests the presence of a basic horizontal vestibulo ocular reflex that is very similarly organized, and corroborates the hypothesis that major behavioural differences in the performance of compensatory eye movements between species result from the properties of supplementary networks and NOT from differences in a common three neuron vestibulo ocular **arc**.  Eur J Neurosci, 1993    [17] |
| - AMPA receptor GluR1 immunoreactive levels in the magnocellular preoptic area mPOA, the arcuate nucleus **ARC**, and the suprachiasmatic nucleus SCN were found to be markedly elevated during the time of the LH surge in estradiol progesterone treated castrate rats compared to those of the vehicle only treated castrate rat.  Front Neuroendocrinol, 1994    [15] |
| - These results indicate that mu receptors may be autoreceptors on **ARC** beta endorphin neurons.  Neuroendocrinology, 1990    [14] |
| - Sperm centration occurs along one side of the egg and appears to follow an **arc** like trajectory as a result of vegetal and inward movements affected by colchicine and cytochalasin B  Dev Biol, 1994    [11] |
| - Besides stress fibers, **arc** like actin bundles have been detected in spreading cells.  J Cell Biol, 1984    [10] |
| - Conversely, K cells exhibited delayed **arc** formation and a reduction in number of cells forming arcs.  J Cell Biol, 1995    [10] |
| - As **Arc** appears to be component of the neuronal cytoskeleton, it may be involved in structural alterations underlying neuronal plasticity triggered by cocaine.  J Neurochem, 1995    [10] |
| - An important structural feature of these cells is an actin containing **arc** like band on the periphery of lamella.  Biull Eksp Biol Med, 1981    [10] |
| - **ARC** and PAG stimulated inhibited a nociceptive reflex tail dip in 52.5 degrees C water, and naloxone did NOT reliably reverse this inhibition.  Brain Res, 1995    [10] |
| - anEAA synapse in the CVLM is important in the cardiopulmonary reflex **arc**.  Exp Brain Res, 1989    [10] |
| - This fold develops into an **arc**.  Symp Soc Exp Biol, 1993    [10] |
