# Supplementary material for: Content-rich biological network constructed by mining PubMed abstracts
Source: BMC Bioinformatics. 2004 Oct 8;5:147. doi: 10.1186/1471-2105-5-147 (PMC528731; doi:10.1186/1471-2105-5-147)
Supplement: Additional File 5 — The original Chilibot query results of the term "long-term potentiation (LTP)" and 22 other terms, limiting the latest references analyzed to the years 1990, 1995, 2000, and 2004. [file 1471-2105-5-147-S5.bz2 › chilibotAdditionalFile5/ltp1995/html/ATF.html]

 


**ATF** (Input: ATF ) 

---


|  |
| --- |
| **Google Searches:** Entire Web  | EDU domain only  | PDF files only |

.

|  |
| --- |
| **External Links:** OMIM | LocusLink | Swissprot | GeneCards |

  
**Maps of ATF**

|  |
| --- |
| Simple Complete graph in radiant tree square layout. |

**New Hypothesis !**

|  |
| --- |
|  |

**Synonyms** 

|  |
| --- |
| - atf   [PubMed] |

**Synopsis**

|  |
| --- |
| - These results suggest that BLV Tax interacts directly with CREB **ATF** like factors to activate viral mRNA transcription.  Virology, 1995    [23] |
| - Our data further suggest that c Fos can act as a repressor of the c Jun **ATF** 2 binding site, revealing an important functional difference, with respect to canonical AP 1 elements.  Oncogene, 1995    [20] |
| - These results suggest a model involving the inhibition of IFN gamma AP 1 CREB **ATF** DNA binding complexes as one of the mechanisms involved in the negative regulatory action of glucocorticoids on IFN gamma gene expression and support the relevance of AP 1 CREB **ATF** binding factors during the transcriptional activation of the IFN gamma promoter in T cells.  J Biol Chem, 1995    [20] |
| - They further suggest that the **ATF** CREB YY1 complex serves as a target for the adenovirus 243 amino acid E1A protein.  J Virol, 1995    [19] |
| - The regulation of **ATF** 1 expression through RNA stability in cells of different states suggests that **ATF** 1 may play an active role in cell growth and differentiation.  J Immunol, 1995    [19] |
| - This shows that at least one specific member of the **ATF** CREB family of transcription factors is involved in mediating transactivation by the HCMV IE86 protein.  J Virol, 1995    [19] |
| - In addition, electrophoretic mobility shift assays demonstrate that a B **ATF** c Jun protein complex can interact with DNA containing a consensus binding site for AP 1, suggesting that B **ATF** functions as a tissue specific modulator of the AP 1 transcription complex in human cells.  Oncogene, 1995    [18] |
| - A CREB **ATF** element was found to be essential for basal transcription of the flt 1 expression.  J Biol Chem, 1995    [18] |
| - We found that Tax enhanced the binding of one member of the **ATF** CREB family, CREB 1, to each of the three HTLV I LTR 21 bp repeats  J Virol, 1995    [16] |
| - Phosphorylation dependent activation of the DNA binding activity of **ATF** 2, which appears to be regulated by the stress activated protein kinases, may play an important role in the earliest stages of the genetic response to ischemia reperfusion by targeting **ATF** 2 and c Jun to specific promoters, including the c jun promoter and those containing **ATF** CREs.  J Biol Chem, 1995    [16] |
| - While oligonucleotides containing either legitimate **ATF** CRE or AP 1 binding sequences competed for binding, antibody supershift experiments suggested that neither CREB **ATF** 1 nor AP 1 are major factors binding to IdATF.  Mol Cell Biol, 1995    [13] |
| - TheX ray structure of the GCN4 bZIP protein bound to DNA containing the **ATF** CREB recognition sequence has been refined at 2.2 A.  J Mol Biol, 1995    [10] |
| - Furthermore the **ATF** like binding activity detected in extracts from fission yeast cells is entirely lost upon deletion of the atf1 gene.  EMBO J, 1995    [10] |
| - Supershift electrophoretic gel mobility shift assays and immunoprecipitation analysis provided further evidence that both CREB and **ATF** 1 are present in the complex.  J Immunol, 1995    [10] |
| - We conclude that the **ATF** 1 CREB heterodimer is involved in the constitutive expression of the Na,K ATPase alpha 1 subunit gene.  Nucleic Acids Res, 1995    [10] |
