# Supplementary material for: Content-rich biological network constructed by mining PubMed abstracts
Source: BMC Bioinformatics. 2004 Oct 8;5:147. doi: 10.1186/1471-2105-5-147 (PMC528731; doi:10.1186/1471-2105-5-147)
Supplement: Additional File 5 — The original Chilibot query results of the term "long-term potentiation (LTP)" and 22 other terms, limiting the latest references analyzed to the years 1990, 1995, 2000, and 2004. [file 1471-2105-5-147-S5.bz2 › chilibotAdditionalFile5/ltp1995/html/ZIF268.html]

 


**ZIF268** (Input: ZIF268 ) 

---


|  |
| --- |
| **Google Searches:** Entire Web  | EDU domain only  | PDF files only |

.

|  |
| --- |
| **External Links:** OMIM | LocusLink | Swissprot | GeneCards |

  
**Maps of ZIF268**

|  |
| --- |
| Simple Complete graph in radiant tree square layout. |

**New Hypothesis !**

|  |
| --- |
|  |

**Synonyms** 

|  |
| --- |
| - zif268   [PubMed] |

**Synopsis**

|  |
| --- |
| - These findings suggest that genetic and activity dependent regulation of **zif268** may influence learning performance.  Hippocampus, 1994    [19] |
| - However, the TrkA isoform containing the variable exon showed significantly higher activation by NT 3, which was detected by stimulation of TrkA autophosphorylation, induction of **ZIF268** transcription, and cellular differentiation.  Proc Natl Acad Sci U S A, 1994    [15] |
| - From this data we conclude that the synapsin I gene is a target of the **zif268** transcription factor.  J Biol Chem, 1994    [14] |
| - the 50 train stimulus pattern resulted in a robust induction of c fos and c jun mRNA, in addition to **zif268** and junB.  J Neurosci, 1993    [14] |
| - Band 3 formed with gamma actin and **zif268** 3 SRE probes.  J Biol Chem, 1994    [10] |
| - We conclude that the **zif268** SRE like sequences are functional and probably account for the coordinate induction of **zif268** and c fos.  Mol Cell Biol, 1989    [10] |
| - Neuronal excitation elicits rapid transcriptional activation of several immediate early genes, for example c fos, c jun and **zif268**.  Nature, 1993    [10] |
| - RNAs from several brain regions were analyzed by Northern blot hybridization for their relative concentrations of nine IEG mRNAs c fos, c jun, junB, TIS 1 nur77, TIS7, TIS8 **zif268**, TIS10, TIS11, and TIS21 .  Brain Res Mol Brain Res, 1993    [10] |
| - Proteins encoded by the TIS genes include two transcription factors TIS8 known as egr1 NGFIA **zif268** and TIS1 also known as NGFIB nur77 N10 .  Biochim Biophys Acta, 1995    [10] |
| - administration of maximal electroconvulsive shock led to robust **zif268** activation throughout the hippocampus, enhancement of synaptic responses, occlusion of LTP produced by discrete high frequency stimulation, and spatial learning deficits in the water task.  J Neurosci, 1994    [7] |
| - The levels of messenger RNAs for c fos, **zif268**, brain derived neurotrophic factor and trkB were consistently increased in cortex ipsilaterally to the lesion, while c jun messenger RNA content was only slightly increased.  Neuroscience, 1993    [6] |
