# Supplementary material for: Content-rich biological network constructed by mining PubMed abstracts
Source: BMC Bioinformatics. 2004 Oct 8;5:147. doi: 10.1186/1471-2105-5-147 (PMC528731; doi:10.1186/1471-2105-5-147)
Supplement: Additional File 5 — The original Chilibot query results of the term "long-term potentiation (LTP)" and 22 other terms, limiting the latest references analyzed to the years 1990, 1995, 2000, and 2004. [file 1471-2105-5-147-S5.bz2 › chilibotAdditionalFile5/ltp1995/html/CAMKII.html]

 


**CAMKII** (Input: CAMKII ) 

---


|  |
| --- |
| **Google Searches:** Entire Web  | EDU domain only  | PDF files only |

.

|  |
| --- |
| **External Links:** OMIM | LocusLink | Swissprot | GeneCards |

  
**Maps of CAMKII**

|  |
| --- |
| Simple Complete graph in radiant tree square layout. |

**New Hypothesis !**

|  |
| --- |
|  |

**Synonyms** 

|  |
| --- |
| - calcium calmodulin dependent protein kinase ii   [PubMed] |
| - camkii   [PubMed] |

**Synopsis**

|  |
| --- |
| - These results suggest that postsynaptic **CaMKII** plays a role in the induction of LTP LTD in visual cortex.  Neuroreport, 1992    [23] |
| - These results, together with previous studies, suggest that postsynaptic **CaMKII** activity is necessary and sufficient to generate LTP.  Science, 1994    [23] |
| - Expression of the alpha isoform of **CaMKII** showed a transient increase over the soma and a more persistent increase over the dendritic field of dentate granule cells.  Neuron, 1994    [19] |
| - Calciumcalmodulin dependent protein kinase II  [**CAMKII**]  role in learning and memory.  Mol Cell Biochem, 1993    [19] |
| - Among the protein molecules specifically located in presynaptic terminals, synapsin I and calcium calmodulin dependent protein kinase II  [**CAMKII**]  CaM kinase II have been shown to modulate evoked transmitter release in the squid giant synapse.  Proc Natl Acad Sci U S A, 1990    [16] |
| - The role of several biological molecules in learning and memory are considered, for example, protein kinase C PKC, Ca Calmodulin kinase II **CaMKII**, GAP 43, and glutamate receptors.  Mol Cell BiochemMol Cell Biochem, 1990    [16] |
| - Calcium calmodulin dependent protein kinase II  [**CAMKII**]  CaM kinase II is the most abundant protein kinase in the brain and is believed to play an important role in the regulation of synaptic transmission, long term potentiation  [LTP]  and other forms of neuronal plasticity.  Brain Res, 1994    [16] |
| - Calciumcalmodulin dependent protein kinase II  [**CAMKII**]  CamK II is a major neuronal protein which plays a significant role in the cellular process of long term potentiation  [LTP]  LTP, and vesicular release of neurotransmitters.  J Neurosci, 1995    [16] |
| - Extracellularapplication of protein kinase inhibitors was used to examine the role of calcium calmodulin dependent protein kinase II  [**CAMKII**]  CaM KII in synaptic transmission in the CA1 region of rat hippocampus.  Brain Res, 1993    [15] |
| - Our data therefore indicate that certain nerve terminal populations in the rat brain contain high levels of calcium calmodulin dependent protein kinase II  [**CAMKII**] .  Synapse, 1989    [14] |
| - The biphasic regulation of **CaMKII** alpha mRNA may be of considerable functional importance for the long term response of granule cells to local stimulation of NMDA receptors or NO release.  Brain Res Mol Brain Res, 1995    [14] |
| - The **CaMKII** Asp 286 mice show normal LTP at high frequency stimulation, but in the 5 10 Hz range, they show a shift in the frequency response curve favoring LTD.  Cell, 1995    [13] |
| - These mutant mice exhibit specific learning impairments, an indication that alpha **CaMKII** has a prominent role in spatial learning, but that it is NOT essential for some types of non spatial learning.  Science, 1992    [13] |
| - Protein kinases other than **CaMKII** or PKC ex.  Int J Biochem, 1994    [10] |
| - Calciumcalmodulin dependent protein kinase II  [**CAMKII**] .  Curr Top Cell Regul, 1990    [10] |
