# Supplementary material for: Content-rich biological network constructed by mining PubMed abstracts
Source: BMC Bioinformatics. 2004 Oct 8;5:147. doi: 10.1186/1471-2105-5-147 (PMC528731; doi:10.1186/1471-2105-5-147)
Supplement: Additional File 5 — The original Chilibot query results of the term "long-term potentiation (LTP)" and 22 other terms, limiting the latest references analyzed to the years 1990, 1995, 2000, and 2004. [file 1471-2105-5-147-S5.bz2 › chilibotAdditionalFile5/ltp1995/html/ACTIN.html]

 


**ACTIN** (Input: ACTIN ) 

---


|  |
| --- |
| **Google Searches:** Entire Web  | EDU domain only  | PDF files only |

.

|  |
| --- |
| **External Links:** OMIM | LocusLink | Swissprot | GeneCards |

  
**Maps of ACTIN**

|  |
| --- |
| Simple Complete graph in radiant tree square layout. |

**New Hypothesis !**

|  |
| --- |
|  |

**Synonyms** 

|  |
| --- |
| - actin   [PubMed] |

**Synopsis**

|  |
| --- |
| - These data suggest that synapsin IIa may cross link synaptic vesicles and **actin** filaments in the nerve terminal.  J Neurochem, 1994    [23] |
| - An original transformation of the fluorescence data, which estimates the disappearance rate of **actin** monomer toward the critical concentration, is presented and shown to be of general usefulness for the study of **actin** binding proteins.  J Biol Chem, 1992    [20] |
| - SynapsinI plays an important role in the regulation of neurotransmitter release, since it binds to synaptic vesicles and to the cytoskeleton, and it bundles F **actin** and microtubules.  Biochem Int, 1990    [19] |
| - Thesynapsins are neuronal phosphoproteins that bind to small synaptic vesicles and to **actin** filaments and are believed to play a regulatory role in neurotransmitter release.  J Biol Chem, 1993    [19] |
| - These observations suggest that synapsin I has a phosphorylation dependent nucleating effect on **actin** polymerization.  J Biol Chem, 1992    [19] |
| - The **actin** binding activity of synapsin I phosphorylated by cAMP dependent protein kinase or by calmodulin dependent protein kinase II showed similar sensitivity to calmodulin inhibition to unphosphorylated synapsin I.  Biochemistry, 1995    [19] |
| - Brain beta spectrin contains three structural domains and we suggest the position of several functional domains including f **actin**, synapsin I, ankyrin and spectrin self association sites.  Brain Res Mol Brain Res, 1993    [19] |
| - Our findings suggest that synapsin I exerts a control on the physical characteristics of the cytoskeletal network of the nerve terminal and are consistent with the proposed role of synapsin I in mediating the interaction of synaptic vesicles with **actin**.  J Physiol Paris, 1993    [16] |
| - Phalloidin staining and immunohistochemistry showed that the neuroblast was richer in F **actin**, beta tubulin, MAP1, MAP2, tau, calspectin, and synapsin I than the matrix cell.  Arch Histol Cytol, 1992    [15] |
| - Wehave analyzed by in situ hybridization the distribution of Fx an **actin** sequestering peptide mRNA in the brain of young and old rats.  Neurosci Lett, 1993    [14] |
| - These data support the view that synapsin I is involved in the regulation of the dynamics of the **actin** based network during the exo endocytotic cycle.  Neuron, 1992    [14] |
| - The 45 residue COOH extension that distinguishes synapsin Ia from synapsin Ib appears NOT to be involved with **actin** binding, since no differences were found in the ability of N40b and N34 to be photo cross linked to **actin**.  Biochemistry, 1991    [12] |
| - Binding of synapsin I to **actin** can be demonstrated when synaptic vesicles are present in the medium and appears to be modulated by ionic strength and synapsin I phosphorylation.  FEBS Lett, 1993    [10] |
| - We propose that synapsin I links synaptic vesicles to **actin** filaments in the presynaptic nerve terminal.  Bioessays, 1990    [10] |
| - Along the axon, phosphorylation could be functional in preventing synapsin I from forming, with **actin**, a dense meshwork that would restrict organelle movement.  J Neurosci, 1991    [10] |
