# Supplementary material for: Content-rich biological network constructed by mining PubMed abstracts
Source: BMC Bioinformatics. 2004 Oct 8;5:147. doi: 10.1186/1471-2105-5-147 (PMC528731; doi:10.1186/1471-2105-5-147)
Supplement: Additional File 5 — The original Chilibot query results of the term "long-term potentiation (LTP)" and 22 other terms, limiting the latest references analyzed to the years 1990, 1995, 2000, and 2004. [file 1471-2105-5-147-S5.bz2 › chilibotAdditionalFile5/ltp1995/html/ACTININ.html]

 


**ACTININ** (Input: ACTININ ) 

---


|  |
| --- |
| **Google Searches:** Entire Web  | EDU domain only  | PDF files only |

.

|  |
| --- |
| **External Links:** OMIM | LocusLink | Swissprot | GeneCards |

  
**Maps of ACTININ**

|  |
| --- |
| Simple Complete graph in radiant tree square layout. |

**New Hypothesis !**

|  |
| --- |
|  |

**Synonyms** 

|  |
| --- |
| - actinin   [PubMed] |

**Synopsis**

|  |
| --- |
| - These results suggest that PI 3 kinase  [PI-3K]  binds to alpha **actinin** and regulates cytoskeletal reorganization.  Biochem J, 1994    [23] |
| - These paracrystalline inclusions have been shown previously using immunocytochemistry to share epitopes with actin, tropomyosin, alpha **actinin** and vinculin.  Brain Res, 1987    [19] |
| - This shows that the native thin filaments contained alpha **actinin** and nebulin.  J Biochem (Tokyo), 1995    [19] |
| - Cortical actin and alpha **actinin** of the bacterium treated cells showed disorganization, and pericellular fibronectin was degraded by both whole T. denticola and the isolated proteinase.  Infect Immun, 1995    [18] |
| - Examination of doubly stained cardiocytes with anti annexin V and anti alpha **actinin** by a confocal laser scanning microscope suggests that annexin V is localized in association with the Z line of rat cardiac myocytes.  Cardiovasc Res, 1995    [16] |
| - We found that stimulation of adherent neutrophils with TNF causes the redistribution to a Triton insoluble fraction of alpha **actinin**, beta 2 integrins, and the four components whose assembly constitutes an active NADPH oxidase the gp91 phox, p22 phox, p47 phox, and p67 phox proteins.  J Leukoc Biol, 1995    [16] |
| - Therefore, this study shows that alpha **actinin** accumulation is a consistent, specific manifestation of the AE phenotype and forms the basis for the development of a safe alternative test for detecting AE bacteria.  J Infect Dis, 1995    [16] |
| - Staining of PKC and vimentin, alpha **actinin**, and vinculin suggests that PKC participates actively in the transduction of mechanical signals to the cell through focal adhesions and the cytoskeleton, although only PKC seemed to change with short time periods of strain.  J Bone Miner Res, 1994    [16] |
| - Consequently, the 550 kDa protein may play an important role in the binding of myofibrils to the basal lamina by interaction with F actin, alpha **actinin**, laminin, fibronectin or Type IV collagen.  Biochim Biophys Acta, 1995    [16] |
| - Although the reason for fish white muscle alpha **actinin** s close affinity to actin was NOT clearly established, our results suggested that the physicochemical environment of the Z filaments in Z disks might be crucial.  Comp Biochem Physiol B Biochem Mol Biol, 1995    [16] |
| - alongwith the unsuitability of actin polymers from oxidized monomers to interact with both filamin and alpha **actinin**, suggest that hydrogen peroxide influences actin dynamics mainly by changing the F actin structure.  Biophys J, 1995    [16] |
| - Confocal microscopy showed alpha **actinin** to be localized along actin stress fibers in wild type cells, and in 5.51 cells stress fibers were absent and alpha **actinin** was associated with F actin in the filopodia.  Exp Cell Res, 1995    [15] |
| - In addition to actin, the ARC was enriched with proteins that showed cross reactivity to antibodies to alpha **actinin** and the 50K actin binding protein elongation factor 1 alpha from Dictyostelium.  Arch Med Res, 1992    [15] |
| - Using antibodies directed against the 27 kDa actin binding domain of alpha **actinin**, a reliable carrier for actin binding sites ABS 1, ABS 2 and ABS 3 involved in dystrophin and filamin, it was shown that PIP2 affects the ABS 3 environment.  Biochem Biophys Res Commun, 1995    [15] |
| - In actin bundles, the actin cross linking protein, alpha **actinin**, was excluded from sites of myosin localization but concentrated in paired sites flanking each myosin ribbon, suggesting that myosin filament association may initiate a pathway for the formation of actin filament bundles.  J Cell Biol, 1995    [15] |
