# Supplementary material for: Content-rich biological network constructed by mining PubMed abstracts
Source: BMC Bioinformatics. 2004 Oct 8;5:147. doi: 10.1186/1471-2105-5-147 (PMC528731; doi:10.1186/1471-2105-5-147)
Supplement: Additional File 5 — The original Chilibot query results of the term "long-term potentiation (LTP)" and 22 other terms, limiting the latest references analyzed to the years 1990, 1995, 2000, and 2004. [file 1471-2105-5-147-S5.bz2 › chilibotAdditionalFile5/ltp1995/html/SYNAPTOTAGMIN.html]

 


**SYNAPTOTAGMIN** (Input: SYNAPTOTAGMIN ) 

---


|  |
| --- |
| **Google Searches:** Entire Web  | EDU domain only  | PDF files only |

.

|  |
| --- |
| **External Links:** OMIM | LocusLink | Swissprot | GeneCards |

  
**Maps of SYNAPTOTAGMIN**

|  |
| --- |
| Simple Complete graph in radiant tree square layout. |

**New Hypothesis !**

|  |
| --- |
|  |

**Synonyms** 

|  |
| --- |
| - synaptotagmin   [PubMed] |

**Synopsis**

|  |
| --- |
| - Since both types of organelles undergo calcium dependent exocytosis, these findings support a general role of **synaptotagmin** as an exocytotic calcium receptor.  J Neurosci, 1993    [19] |
| - These synapses expressed proteins characteristic of mature synapses immunofluorescence staining showed the presence of synaptophysin, **synaptotagmin**, VAMP synaptobrevin 2, syntaxin and neurexin.  J Physiol Paris, 1995    [19] |
| - Evidence is mounting that the vesicle proteins vamp, rab3A, synaptophysin, **synaptotagmin** and SV2 play an important role in regulated exocytosis, by regulating neurotransmitter uptake, vesicle targeting and fusion with the presynaptic plasma membrane.  J Cell Sci Suppl, 1993    [16] |
| - Evidence is mounting that the vesicle proteins vamp, rab3A, synaptophysin, **synaptotagmin** and SV2 play an important role in regulated exocytosis, by regulating neurotransmitter uptake, vesicle targeting and fusion with the presynaptic plasma membrane.  Prog Brain Res, 1995    [16] |
| - However, in the adrenal medulla, **synaptotagmin** was found in both synaptophysin containing microvesicles and in chromaffin granules that are devoid of synaptophysin, suggesting a shared role for **synaptotagmin** in the exocytosis of small synaptic vesicles and large dense core catecholaminergic vesicles.  J Biol Chem, 1991    [15] |
| - These results provide support for the hypothesis that **synaptotagmin**, a Ca and phospholipid binding protein, is important for regulated exocytosis in neurons.  Cell, 1993    [14] |
| - As presynaptic calcium entry regulates synaptic vesicle fusion, our results indicate that **synaptotagmin** might link neuronal activity with synaptic growth.  Nature, 1993    [14] |
| - In particular, mRNAs encoding synaptophysin IIb and **synaptotagmin** II which is undetectable at E9 become predominant.  Mol Cell Neurosci, 1995    [14] |
| - Membrane proteins of SSV involved in fusion **synaptotagmin** and trafficking Rab 3 together with synaptophysin have been identified on SLMV.  Neurochem Int, 1995    [10] |
| - Neuroscience, 1994    [10] |
| - Immunoprecipitation with anti syntaxin or anti VAMP antibodies revealed a syntaxin SNAP25 VAMP **synaptotagmin** complex.  FEBS Lett, 1995    [10] |
| - The contrasting effects of neuronotypic differentiation on levels of synaptophysin and p65 **synaptotagmin** indicate potential differences in the regulation of these proteins in PC12 cells.  J Neurochem, 1993    [10] |
| - We created cell lines expressing both SV2 and **synaptotagmin**, both **synaptotagmin** and synaptophysin, and lines expressing all three synaptic vesicle proteins.  J Cell Biol, 1993    [10] |
| - **Synaptotagmin**, synaptobrevin, p29 and SV2 immunoreactivities retained a primarily punctate distribution.  J Cell Biol, 1993    [10] |
| - For these experiments, we cloned a chick p65 **synaptotagmin** cDNA using a reduced stringency screen with a rat p65 cDNA probe.  Dev Biol, 1993    [10] |
