# Supplementary material for: Content-rich biological network constructed by mining PubMed abstracts
Source: BMC Bioinformatics. 2004 Oct 8;5:147. doi: 10.1186/1471-2105-5-147 (PMC528731; doi:10.1186/1471-2105-5-147)
Supplement: Additional File 5 — The original Chilibot query results of the term "long-term potentiation (LTP)" and 22 other terms, limiting the latest references analyzed to the years 1990, 1995, 2000, and 2004. [file 1471-2105-5-147-S5.bz2 › chilibotAdditionalFile5/ltp1995/html/SYNAPTOPHYSIN.html]

 


**SYNAPTOPHYSIN** (Input: SYNAPTOPHYSIN ) 

---


|  |
| --- |
| **Google Searches:** Entire Web  | EDU domain only  | PDF files only |

.

|  |
| --- |
| **External Links:** OMIM | LocusLink | Swissprot | GeneCards |

  
**Maps of SYNAPTOPHYSIN**

|  |
| --- |
| Simple Complete graph in radiant tree square layout. |

**New Hypothesis !**

|  |
| --- |
|  |

**Synonyms** 

|  |
| --- |
| - synaptophysin   [PubMed] |

**Synopsis**

|  |
| --- |
| - These results suggest that in the dog spinal cord and dorsal root ganglia, peptide containing pathways complete their development during postnatal life, together with the full expression of NSE and synapsin I **synaptophysin** immunoreactivities.  Am J Anat, 1991    [20] |
| - These results suggest an analogy to **synaptophysin** loss and the appearance of dendritic threads described in Alzheimer s disease AD, as an early stage in the formation of neurofibrillary tangles NFT .  Brain Res, 1995    [20] |
| - T3 in physiological concentrations increased both granule neuron survival after three days in culture and synaptic vesicle protein formation, as shown by immunostaining with antibodies against **synaptophysin**.  Neuroreport, 1992    [19] |
| - In experiments in which cultured neurons were stained immunocytochemically with antibody against **synaptophysin** after electrophysiological recordings, hot spots were found to correspond to probable synaptic sites.  J Neurophysiol, 1995    [18] |
| - In adult retina, synapsin I and **synaptophysin** were mainly localized in synaptic fields and processes but all three proteins showed a distinct pattern of distribution.  J Neurosci Res, 1992    [18] |
| - Three dimensional 3 D reconstructions of serial optical sections showed that the tau positive tortuous axons clustered in the neuritic plaques were often continuous with **synaptophysin** positive distended terminals.  J Neuropathol Exp Neurol, 1993    [18] |
| - However, both proteins are found on membranes which are immunoisolated with antibodies directed against the synaptic vesicle membrane protein **synaptophysin**, suggesting that dynamin and p145 are localized in part on organelles which represent intermediate stages in the reformation of synapatic vesicles during recycling.  J Biol Chem, 1994    [16] |
| - Hence, the pinealocyte is the first neuroendocrine cell type that has now been shown to concentrate **synaptophysin** positive microvesicles in perivascular process endings.  J Neurosci Res, 1993    [16] |
| - These observations show that the distribution pattern of Synapsin I and **Synaptophysin** in peripheral extensions of vestibular afferent neurons during development is identical to that described in axonal processes of CNS neurons.  J Neurosci, 1991    [16] |
| - This study showed that microwave antigen retrieval significantly enhances the immunoreactivity of SNAP 25, chromogranin A, **synaptophysin**, GAP 43, ubiquitin and tau, in addition to that of beta APP, in formalin fixed, paraffin embedded tissue, and reveals NF68 antigenicity where it was NOT previously detectable.  Acta Neuropathol (Berl), 1994    [12] |
| - Immunocytochemical reactions with antibodies to **synaptophysin** and glial fibrillary acidic protein on adjacent sections demonstrated coexpression in individual tumor cells.  Lab Invest, 1991    [10] |
| - Neuroscience, 1994    [10] |
| - Concomitantly, the two synaptic vesicle proteins dropped, **synaptophysin** > 50% and synapsin I > 85%.  Neurosci Lett, 1995    [10] |
| - The enriched KA2 immunoreactivity co localized with the synaptic vesicle protein **synaptophysin** at the resolution of light microscopy, indicating synaptic localization of KA2.  Neuroscience, 1995    [10] |
| - Early in the developing cerebellar cortex, the expression of NF protein and **synaptophysin** occurred in discrete patches or columns similar to those described for other antigens .  J Comp Neurol, 1993    [10] |
