# Supplementary material for: Content-rich biological network constructed by mining PubMed abstracts
Source: BMC Bioinformatics. 2004 Oct 8;5:147. doi: 10.1186/1471-2105-5-147 (PMC528731; doi:10.1186/1471-2105-5-147)
Supplement: Additional File 5 — The original Chilibot query results of the term "long-term potentiation (LTP)" and 22 other terms, limiting the latest references analyzed to the years 1990, 1995, 2000, and 2004. [file 1471-2105-5-147-S5.bz2 › chilibotAdditionalFile5/ltp1995/html/LTP_SYNAPSIN I.html]

 


 **LTP** and **SYNAPSIN I** 
  
Found 9 abstracts in PubMed,  **9 abstracts were retrieved and analyzed**.  


---

 Search Google  |
 PDF files only 
|  EDU domain only 

---

**Interactive relationship** (e.g. stimulation, inhibition, etc)

**Parallel relationship** (e.g. studied together, co-existance, homology, etc.)

- Electrophysiology reveals that mice lacking  **synapsin I**  exhibit a selective increase in paired pulse facilitation, with no major alterations in other synaptic parameters such as long term potentiation  [ **LTP** ] .  Ref: 7902212 Cell, 1993
- These results suggest that glutamate can activate CaM kinase II through NMDA receptors in the induction of  **LTP**  and in turn stimulates the phosphorylation of target proteins such as MAP2 and  **synapsin I** .  Ref: 8282267 Nippon Yakurigaku Zasshi, 1993
- Regulation of neuronal plasticity by phosphorylation of  **synapsin I**  and of postsynaptic substrates necessary for long term potentiation  [ **LTP** ]  is another dynamic area of investigation.  Ref: 1323238 Annu Rev Biochem, 1992
