# Supplementary material for: Content-rich biological network constructed by mining PubMed abstracts
Source: BMC Bioinformatics. 2004 Oct 8;5:147. doi: 10.1186/1471-2105-5-147 (PMC528731; doi:10.1186/1471-2105-5-147)
Supplement: Additional File 5 — The original Chilibot query results of the term "long-term potentiation (LTP)" and 22 other terms, limiting the latest references analyzed to the years 1990, 1995, 2000, and 2004. [file 1471-2105-5-147-S5.bz2 › chilibotAdditionalFile5/ltp1995/html/LTP_PKC.html]

 


 **LTP** and **PKC** 
  
Found 91 abstracts in PubMed,  **30 abstracts were retrieved and analyzed**.  


---

 Search Google  |
 PDF files only 
|  EDU domain only 

---

**Interactive relationship** (e.g. stimulation, inhibition, etc)

- Antibodies to postsynaptic  **PKC**  substrate neurogranin prevent long term potentiation  [ **LTP** ]  in hippocampal CA1 neurons.  Ref: 7620629 Eur J Neurosci, 1995
- But quantitative differences exist both the induction and the maintenance of Ca  **LTP**  appeared to be more susceptible to block by postsynaptic  **PKC**  inhibition than those of TS  **LTP** .  Ref: 8069668 Brain Res, 1994
- The tetanically and the ACPD induced  **LTP**  of the NMDAR EPSC were dependent on protein kinase C  **PKC**  stimulation, being strongly inhibited by the  **PKC**  inhibitor PKCI 19 31.  Ref: 7891148 J Neurosci, 1995
- A comparison of the potencies of  **PKC**  19 36 and Ala286 CaMKII 281 302 in the physiological assay with their Ki values for protein kinase inhibition in vitro indicates that the blockade of induction of  **LTP**  observed for each peptide is attributable to inhibition of  **PKC** .  Ref: 8197132 Proc Natl Acad Sci U S A, 1994
- Three days after long term potentiation  [ **LTP** ]   **LTP**  there is a decrease in the gene expression of protein F1 GAP 43 and gamma  **PKC**  in CA3 pyramidal cells that is correlated with the magnitude of  **LTP** .  Ref: 8750840 Brain Res Mol Brain Res, 1995
- The data suggest that a membrane insertion of alpha beta  **PKC**  is not a prerequisite for the  **LTP**  induced increased phosphorylation of  **PKC**  substrates.  Ref: 7552327 Brain Res, 1995
- functional cross talk between calcium CaM and  **PKC**  pathways occurs during the induction of  **LTP** .  Ref: 7646896 Neuron, 1995
- Immunoblot analysis of  **PKC**  immunoreactivity in cytosolic and membrane fractions revealed a  **LTP**  induced translocation of gamma  **PKC**  but not alpha beta  **PKC**  into the cytosol in dentate gyrus but also in the other ipsilateral hippocampal regions.  Ref: 8003659 Neuroreport, 1994
- A selective 50% increase only in the levels of mRNA NR1 subunits containing this consensus sequence for  **PKC**  phosphorylation was seen 48 h after  **LTP**  induction.  Ref: 7703398 Neuroreport, 1994
- Of the  **PKC**  isoforms, only gamma  **PKC**  was up regulated substantially 2 hr after  **LTP**  induction, declining to control levels 48 hr later.  Ref: 7917303 Neuron, 1994
- When the intracellular delivery of either  **PKC**  19 31 alone or PMB  **PKC**  19 31 was made to begin after the full establishment of Ca  **LTP** , it soon made the maintained potentiation begin to decline, the EPSP amplitude gradually returning to the control value before the exposure to increased calcium.  Ref: 8069668 Brain Res, 1994
- Changes in the activity of the enzyme protein kinase C  **PKC**  have been implicated in learning and memory consolidation, and in the induction of long term potentiation  [ **LTP** ] .  Ref: 8003245 Behav Brain Res, 1994
- The change in the expression of  **PKC**  sensitive NR1 subunits may be the molecular basis for the increased response of the post synaptic cell to released glutamate during the maintenance phase of  **LTP** .  Ref: 7703398 Neuroreport, 1994
- Induction of  **LTP**  in rat hippocampal slices by high frequency stimulation of Schaffer collateral CA1 synapses significantly increased the  **PKC**  dependent phosphorylation of GAP 43, as assessed by quantitative immunoblotting with a monoclonal antibody that recognizes an epitope that is specifically phosphorylated by  **PKC** .  Ref: 7891109 J Neurochem, 1995
- Activation of protein kinase C  **PKC**  and phosphorylation of its presynaptic substrate, the 43 kDa growth associated protein GAP 43, may contribute to the maintenance of hippocampal long term potentiation  [ **LTP** ]   **LTP**  by enhancing the probability of neurotransmitter release and or modifying synaptic morphology.  Ref: 7891109 J Neurochem, 1995
- The involvement of protein kinase C  **PKC**  dependent processes in adaptive and plastic changes underlying neuronal plasticity was tested in an in vivo animal model characterized by targeted cellular ablation of cortical and hippocampal neurons, cognitive impairment and lack of induction of long term potentiation  [ **LTP** ] .  Ref: 7613626 Eur J Neurosci, 1995
- Several lines of evidence indicate that protein kinase C  **PKC**  participates in long term potentiation  [ **LTP** ]   **LTP**  and in certain forms of learning.  Ref: 7583242 Brain Res, 1995
- At both 1 and 2 h after  **LTP** , changes in F1 GAP 43 hybridization were positively correlated with gamma  **PKC**  hybridization and negatively correlated with  **LTP**  magnitude.  Ref: 8750840 Brain Res Mol Brain Res, 1995
- Protein F1 GAP 43 and  **PKC**  gene expression patterns in hippocampus are altered 1 2 h after  **LTP** .  Ref: 8750840 Brain Res Mol Brain Res, 1995
- calcium calmodulin dependent protein kinase II CaMKII and protein kinase C  **PKC**  may play pivotal roles in the different phases of the expression of  **LTP** .  Ref: 8063002 Int J Biochem, 1994
- Thus postsynaptic  **PKC**  inhibition blocks both the induction and the maintenance of Ca  **LTP** , just as it has been shown to do to TS  **LTP** .  Ref: 8069668 Brain Res, 1994
- Protein kinase C  **PKC**  has attracted wide attention as a key enzyme for the expression of long term potentiation  [ **LTP** ]  in the hippocampus, a basic model for memory.  Ref: 7820584 Brain Res, 1994

**Parallel relationship** (e.g. studied together, co-existance, homology, etc.)

- This study tested whether protein kinase C  **PKC**  activation is necessary for sharpening as it is for  **LTP** .  Ref: 8071660 J Neurobiol, 1994
- Using intracellular delivery to rat CA1 hippocampal neurons, we have determined the relative potency of two protein kinase inhibitor peptides,  **PKC**  19 36 and Ala286 CaMKII 281 302, as inhibitors of the induction of  **LTP** .  Ref: 8197132 Proc Natl Acad Sci U S A, 1994
- Furthermore, 2 min and 15 min after induction of  **LTP**  in the Schaffer collateral CA1 pathway the distribution of alpha beta  **PKC**  between the two membrane fractions remained unchanged.  Ref: 7552327 Brain Res, 1995
- In this paper the effects of protein kinase C  **PKC**  inhibitors, polymyxin B PMB and  **PKC**  19 31, given intracellularly to the postsynaptic neuron in the CA1 region, on the induction and maintenance of Ca  **LTP**  were studied and compared with those found in a similar study earlier made in this laboratory on tetanic stimulation induced  **LTP**  TS  **LTP**  18.  Ref: 8069668 Brain Res, 1994
- Using a monoclonal antibody the translocation of the calcium dependent protein kinase C  **PKC**  isoenzymes alpha beta was studied in hippocampal slices after stimulation of glutamate receptors or induction of long term potentiation  [ **LTP** ] .  Ref: 7552327 Brain Res, 1995
- The possible involvement of the calcium dependent protein kinase C  **PKC**  isoenzymes alpha beta and gamma in mechanisms of long term potentiation  [ **LTP** ]   **LTP**  was investigated after tetanic stimulation of the perforant path in vivo.  Ref: 8003659 Neuroreport, 1994
- One way to investigate the role of  **PKC**  in long term potentiation  [ **LTP** ]  is to determine the degree of phosphorylation of its substrates after in situ phosphorylation in hippocampal slices.  Ref: 7755899 Behav Brain Res, 1995
- Using in situ hybridization, we investigated the expression of NR1 subunits sensitive or insensitive to modification by  **PKC**  in the granule cells of the dentate gyrus of the hippocampus, following the induction of long term potentiation  [ **LTP** ]   **LTP** .  Ref: 7703398 Neuroreport, 1994
- These results indicate that arachidonic acid may contribute to  **LTP**  maintenance by activation of presynaptic  **PKC**  and phosphorylation of GAP 43 substrate.  Ref: 7891109 J Neurochem, 1995
- Previous studies have used synthetic peptide analogs, corresponding to sequences within the pseudosubstrate domain of protein kinase C  **PKC**  or the autoregulatory domain of calcium calmodulin dependent protein kinase II CaMKII, in attempts to define the contribution of each of these protein kinases to induction of long term potentiation  [ **LTP** ]   **LTP** .  Ref: 8197132 Proc Natl Acad Sci U S A, 1994
- At 1 h... F1 GAP 43 and gamma  **PKC**  mRNA hybridization were increased, but increases were also observed after control stimulation.  Ref: 8750840 Brain Res Mol Brain Res, 1995
- This hypothesis is confirmed by the experiments in which  **LTP**  like phenomena for early and late cortical IPSPs were shown to be the result of inactivation of PKA and  **PKC** .  Ref: 7754689 Zh Vyssh Nerv Deiat Im I P PavlovaZh Vyssh Nerv Deiat Im I P Pavlova, 1995
- As the epsilon  **PKC**  could be activated by arachidonic acid, it is strongly suggested that in the case of  **LTP** , the presynaptic epsilon  **PKC**  is activated by arachidonic acid released postsynaptically and phosphorylates GAP 43, resulting in the increase in glutamate release.  Ref: 7721190 Nippon Yakurigaku Zasshi, 1995
