# Supplementary material for: Content-rich biological network constructed by mining PubMed abstracts
Source: BMC Bioinformatics. 2004 Oct 8;5:147. doi: 10.1186/1471-2105-5-147 (PMC528731; doi:10.1186/1471-2105-5-147)
Supplement: Additional File 5 — The original Chilibot query results of the term "long-term potentiation (LTP)" and 22 other terms, limiting the latest references analyzed to the years 1990, 1995, 2000, and 2004. [file 1471-2105-5-147-S5.bz2 › chilibotAdditionalFile5/ltp1995/html/LTP_PKA.html]

 


 **LTP** and **PKA** 
  
Found 10 abstracts in PubMed,  **10 abstracts were retrieved and analyzed**.  


---

 Search Google  |
 PDF files only 
|  EDU domain only 

---

**Interactive relationship** (e.g. stimulation, inhibition, etc)

- These data suggest that besides protein kinase C the synergistic activation of  **PKA**  is necessary for the maintenance of  **LTP** .  Ref: 8347813 Neuroreport, 1993
- Thus, activation of  **PKA**  may be a component of the mechanism that generates L  **LTP** .  Ref: 8389057 Science, 1993
- These results indicate that the synapsins are not necessary for 1 the induction or expression of two different forms of  **LTP**  in the hippocampus, 2 the enhancement in transmitter release elicited by activation of the cAMP dependent protein kinase  **PKA**  and 3 the depression of synaptic transmission caused by H 7.  Ref: 8606805 Neuropharmacology, 1995
- Since the late phase of CA1  **LTP**  also requires  **PKA**  but is normal in RI beta mutant mice, our data further suggest that different forms of synaptic plasticity are likely to employ different combinations of regulatory and catalytic subunits.  Ref: 7568030 Proc Natl Acad Sci U S A, 1995
- Ablation by gene targeting of the C beta 1 or the RI beta isoform of  **PKA**  produces a selective defect in mossy fiber  **LTP** , providing genetic evidence for the role of these isoforms in the mossy fiber pathway.  Ref: 8548807 Cell, 1995
- The cAMP dependent protein kinase  **PKA**  has been shown to play an important role in long term potentiation  [ **LTP** ]   **LTP**  in the hippocampus, but little is known about the function of  **PKA**  in long term depression LTD.  Ref: 7568030 Proc Natl Acad Sci U S A, 1995

**Parallel relationship** (e.g. studied together, co-existance, homology, etc.)

- The possible involvement of cAMP dependent protein kinase A  **PKA**  in mechanisms of long term potentiation  [ **LTP** ]  of the Schaffer collateral commissural input of rat CA1 neurones was investigated using several inhibitors in vitro.  Ref: 8347813 Neuroreport, 1993
- To investigate the involvement of N methyl D aspartate NMDA receptor, protein kinase C PKC and calmodulin on long term potentiation  [ **LTP** ]   **LTP**  formation in the superior colliculus SC, the effects of an NMDA receptor antagonist D APV, PKC inhibitors H 7, K 252a, K 252b, polymyxin B, a protein kinase A  **PKA**  inhibitor H 8 and a calcium calmodulin dependent kinase inhibitor calmidazolium on  **LTP**  formation were studied in guinea pig SC slices.  Ref: 8097659 Brain Res, 1993
- Using a genetic approach, we assessed the effects of mutations in protein kinase A  **PKA**  on long term potentiation  [ **LTP** ]   **LTP**  in the mossy fiber pathway and its relationship to spatial and contextual learning.  Ref: 8548807 Cell, 1995
- A genetic test of the effects of mutations in  **PKA**  on mossy fiber  **LTP**  and its relation to spatial and contextual learning.  Ref: 8548807 Cell, 1995
- Inhibitors of cyclic adenosine monophosphate cAMP dependent protein kinase  **PKA**  blocked L  **LTP** , and analogs of cAMP induced a potentiation that blocked naturally induced L  **LTP** .  Ref: 8389057 Science, 1993
- This hypothesis is confirmed by the experiments in which  **LTP**  like phenomena for early and late cortical IPSPs were shown to be the result of inactivation of  **PKA**  and PKC.  Ref: 7754689 Zh Vyssh Nerv Deiat Im I P PavlovaZh Vyssh Nerv Deiat Im I P Pavlova, 1993
