# Supplementary material for: Content-rich biological network constructed by mining PubMed abstracts
Source: BMC Bioinformatics. 2004 Oct 8;5:147. doi: 10.1186/1471-2105-5-147 (PMC528731; doi:10.1186/1471-2105-5-147)
Supplement: Additional File 5 — The original Chilibot query results of the term "long-term potentiation (LTP)" and 22 other terms, limiting the latest references analyzed to the years 1990, 1995, 2000, and 2004. [file 1471-2105-5-147-S5.bz2 › chilibotAdditionalFile5/ltp1995/html/LTP_NMDA.html]

 


 **LTP** and **NMDA** 
  
Found 486 abstracts in PubMed,  **30 abstracts were retrieved and analyzed**.  


---

 Search Google  |
 PDF files only 
|  EDU domain only 

---

**Interactive relationship** (e.g. stimulation, inhibition, etc)

- Post 2 DG long term potentiation  [ **LTP** ]  2 DG  **LTP**  is prevented by block of N methyl D aspartate  **NMDA**  receptors NMDARs.  Ref: 8747233 J Neurophysiol, 1995
- and 2 there is a bell shaped relationship between the degree of activation of  **NMDA**  receptors during the tetani and the magnitude of  **LTP**  of the fEPSPA tetani that generate  **LTP**  of fEPSPN have a low probability to induce  **LTP**  of fEPSPA.  Ref: 8747197 J Neurophysiol, 1995
- The present observation suggests that l  **LTP**  of fEPSPA has a lower threshold than that of fEPSPN, i.e., stronger activation of  **NMDA**  receptors during the tetani is required to induce  **LTP**  of fEPSPN than the one required for inducing  **LTP**  of fEPSPA.  Ref: 8747197 J Neurophysiol, 1995
- This sex difference in  **LTP**  was paralleled by a sex difference in the magnitude of N methyl D aspartate  **NMDA**  receptor activation generated by perforant path HFS.  Ref: 7501277 Neurosci Lett, 1995
- tetanic input, of the type typically used to induce long term potentiation  [ **LTP** ] , will increase calcium influx by increasing receptor binding as well as by reducing voltage dependent block of  **NMDA**  receptors.  Ref: 8580317 Biophys J, 1995
- **NMDA**  R1 antisense oligonucleotide influences the early stage of long term potentiation  [ **LTP** ]  in the CA1 region of rat hippocampus.  Ref: 8787844 Neurosci Lett, 1995
- For example, the immature N methyl D aspartate  **NMDA**  type glutamate receptor channel complex, which plays important roles in long term potentiation  [ **LTP** ]   **LTP** , neuronal migration and synaptic pruning, contains subunits that allow the channel to be opened more easily for a longer period than adult channels.  Ref: 8579213 Brain DevBrain Dev, 1995
- While the mechanisms responsible for  **LTP**  and LTD of excitatory synaptic responses mediated by AMPA receptors AMPARs have been extensively characterized, much less is known about the regulation of  **NMDA**  receptors NMDARs by synaptic activity.  Ref: 7544143 Neuron, 1995
- Drugs that antagonize N methyl D aspartate  **NMDA**  receptor activity, which is required for long term potentiation  [ **LTP** ]   **LTP**  at various hippocampal synapses, block  **LTP**  and impair watermaze learning.  Ref: 7477321 Nature, 1995
- In support of this, both hippocampal long term potentiation  [ **LTP** ]  and spatial learning in a watermaze are impaired by blocking  **NMDA**  receptors with a selective antagonist D 2 amino 5 phosphonovaleric acid AP5 or by a mutation in one of the receptor subunits.  Ref: 7477320 Nature, 1995

**Parallel relationship** (e.g. studied together, co-existance, homology, etc.)

- ... longer and more numerous trains of HFS produced an enduring,  **NMDA**  receptor dependent long term potentiation  [ **LTP** ]   **LTP**  of the potentials.  Ref: 7472506 J Neurosci, 1995
- n = 6, an antagonist of the allosteric glycine site of the  **NMDA**  receptors, Ts generated  **LTP**  of fEPSPA 63.2 8.2% but not of fEPSPN 12.6 4.0%.  Ref: 8747197 J Neurophysiol, 1995
- We have tested, in CA1 hippocampal slices, the hypothesis that the expression of long term potentiation  [ **LTP** ]   **LTP**  by alpha amino 3 hydroxy 5 methyl 4 isoxazolepropionic acid AMPA and or N methyl D aspartate  **NMDA**  receptors depends on the degree of  **NMDA**  receptors activation during the tetanus.  Ref: 8747197 J Neurophysiol, 1995
- As suggested by studies of long term potentiation  [ **LTP** ] ,  **NMDA**  receptor dependent postsynaptic calcium appears to be essential for the development of these storage sites and indeed to trigger their development.  Ref: 7501672 Pharmacol Biochem Behav, 1995
- Expression of  **LTP**  by AMPA and or  **NMDA**  receptors is determined by the extent of  **NMDA**  receptors activation during the tetanus.  Ref: 8747197 J Neurophysiol, 1995
- Although these results do not rule out a contribution of  **NMDA**  mediated dentate  **LTP**  to spatial learning, they indicate that this form of  **LTP**  is not required for normal spatial learning in the watermaze.  Ref: 7477321 Nature, 1995
- Furthermore, they confirm that activation of mGlu receptors by ACPD in vivo facilitates long term potentiation  [ **LTP** ] , and indicate that in the dentate gyrus, ACPD induced slow onset potentiation is  **NMDA**  receptor dependent.  Ref: 8750711 Eur J Pharmacol, 1995
- , an antagonist to the  **NMDA**  subclass of glutamate receptors, was administered prior to high frequency stimulation,  **LTP**  induction was blocked and AEPs were not affected.  Ref: 7643222 J Neurosci, 1995
- We have studied the role of the N methyl D aspartate  **NMDA**  R1 receptor subunit in the mechanism of long term potentiation  [ **LTP** ]   **LTP**  using an antisense oligodeoxynucleotide strategy.  Ref: 8787844 Neurosci Lett, 1995
- In the presence of the  **NMDA**  receptor antagonist 2 amino 5 phosphonovaleric acid APV,  **LTP**  could not be induced.  Ref: 7472421 J Neurosci, 1995
- They further support the assumption of the essential role of the  **NMDA**  R1 receptor subunit in the induction of  **LTP** .  Ref: 8787844 Neurosci Lett, 1995
- Although it is now generally accepted that mossy fibre  **LTP**  is expressed presynaptically, the locus of expression for  **NMDA**  receptor dependent  **LTP**  is controversial.  Ref: 7675078 Nature, 1995
- This has led to the hypothesis that  **NMDA**  receptors, through their involvement in  **LTP** , may be necessary for spatial and other forms of learning.  Ref: 7477321 Nature, 1995
- ACPD failed to induce  **LTP**  of pharmacologically isolated  **NMDA**  receptor mediated EPSPs.  Ref: 8532149 Neuropharmacology, 1995
- Here the two forms of  **LTP**  are compared and it is argued that the balance of evidence favours a postsynaptic locus for  **NMDA**  receptor dependent  **LTP** .  Ref: 7675078 Nature, 1995
- Spatial learning without  **NMDA**  receptor dependent long term potentiation  [ **LTP** ] .  Ref: 7477321 Nature, 1995
