# Supplementary material for: Content-rich biological network constructed by mining PubMed abstracts
Source: BMC Bioinformatics. 2004 Oct 8;5:147. doi: 10.1186/1471-2105-5-147 (PMC528731; doi:10.1186/1471-2105-5-147)
Supplement: Additional File 5 — The original Chilibot query results of the term "long-term potentiation (LTP)" and 22 other terms, limiting the latest references analyzed to the years 1990, 1995, 2000, and 2004. [file 1471-2105-5-147-S5.bz2 › chilibotAdditionalFile5/ltp1995/html/LTP_AMPA.html]

 


 **LTP** and **AMPA** 
  
Found 78 abstracts in PubMed,  **30 abstracts were retrieved and analyzed**.  


---

 Search Google  |
 PDF files only 
|  EDU domain only 

---

**Interactive relationship** (e.g. stimulation, inhibition, etc)

- A reducing and an oxidizing agent had no effect on long term potentiation  [ **LTP** ]  mediated by  **AMPA**  receptors.  Ref: 7813559 Eur J Pharmacol, 1994
- GYKI 52466 reduced the peak amplitude of  **AMPA**  EPSCs and blocked the induction of  **LTP** .  Ref: 7970204 Neurosci Lett, 1994
- At the time of expression, memory and  **LTP**  are blocked by antagonists of glutamate  **AMPA**  receptors.  Ref: 7958619 FASEB J, 1994
- Antagonists of NMDA and  **AMPA**  receptor prevent the development of  **LTP** , and conversely, the agonist of glycine site of NMDA receptor D cycloserine facilitates memory consolidation.  Ref: 8728878 Medicina (B Aires), 1995
- At the time of expression, memory and  **LTP**  are blocked by antagonists of glutamate  **AMPA**  receptors and are accompanied by an enhanced sensitivity of these receptors.  Ref: 7663877 Neurobiol Learn Mem, 1995
- The induction of ACPD induced  **LTP**  was blocked by the alpha amino 3 hydroxy 5 methyl 4 isoxazolepropionate  **AMPA**  receptor antagonist 6 cyano 7 nitroquinoxaline dione CNQX, in a reversible manner.  Ref: 8532149 Neuropharmacology, 1995
- 3H CNQX to  **AMPA**  receptors in slices expressing KCl induced  **LTP** .  Ref: 7874236 Hippocampus, 1994
- In slices in which area CA3 had been removed ACPD failed to induce  **LTP**  when applied alone or together with  **AMPA** .  Ref: 8532149 Neuropharmacology, 1995
- Tetanically induced  **LTP**  involves a similar increase in the  **AMPA**  and NMDA receptor components of the excitatory postsynaptic current investigations of the involvement of mGlu receptors.  Ref: 7891148 J Neurosci, 1995
- In addition, KCl induced  **LTP**  was associated with an increase in 3H  **AMPA**  3H amino 3 hydroxy 5 methylisoxazole 4 propionate binding to CA1 synaptic membranes when measured 40 min after high potassium exposure.  Ref: 7874236 Hippocampus, 1994
- Considered together, these data suggest that ACPD induced  **LTP**  is due to a direct increase in the  **AMPA**  receptor mediated synaptic conductance and involves postsynaptic induction and expression mechanisms.  Ref: 8532149 Neuropharmacology, 1995
- ACPD induced  **LTP**  was associated with a parallel increase in the sensitivity of CA1 neurones to  **AMPA** .  Ref: 8532149 Neuropharmacology, 1995
- These redox agents have no effect on  **AMPA**  synaptic transmission and did not significantly modify the induction and the expression of tetanic  **AMPA**   **LTP** .  Ref: 7882133 C R Acad Sci III, 1994
- Furthermore,  **LTP**  is generally associated with a long lasting increase of the synaptic efficacy of  **AMPA**  receptors but an increasing number of data also suggested that NMDA receptors could be potentiated as well.  Ref: 7775969 J Neurobiol, 1995
- Recent experimental evidence suggests that phospholipase induced changes in binding properties of the alpha amino 3 hydroxy 5 methyl 4 isoxazole propionate  **AMPA**  subtype of glutamate receptors account for the increase in synaptic response observed in long term potentiation  [ **LTP** ]   **LTP** .  Ref: 7743208 Brain Res, 1995
- In physiological conditions a high frequency stimulation HFS of Schaffer collateral commissural fibers induced a  **LTP**  expressed by a persistent increase 73 13%, mean SE, n = 8 10 of  **AMPA**  field potentials LTPA.  Ref: 7666170 J Neurophysiol, 1995
- ... long term potentiation  [ **LTP** ]  induced by single or repeated high frequency stimulation, was seen initially as a predominant potentiation of the  **AMPA**  receptor mediated component.  Ref: 8577363 Neuroscience, 1995
- We have compared the effects of redox drugs on long term potentiation  [ **LTP** ]  mediated by  **AMPA**  or NMDA receptors.  Ref: 7813559 Eur J Pharmacol, 1994
- In CA1 hippocampal neurons, the redox state of NMDA receptors determines  **LTP**  expressed by NMDA but not by  **AMPA**  receptors.  Ref: 7666170 J Neurophysiol, 1995
- These data suggest that in this chronic model of epileptiform activity, there is long term potentiation  [ **LTP** ]  of excitatory mediated events regulated primarily by  **AMPA**  receptors.  Ref: 7588593 Epilepsy Res, 1995
- **LTP**  was expressed, for the most part, in kainate  **AMPA**  receptor mediated responses.  Ref: 7580127 Cereb CortexCereb Cortex, 1995
- While the mechanisms responsible for  **LTP**  and LTD of excitatory synaptic responses mediated by  **AMPA**  receptors AMPARs have been extensively characterized, much less is known about the regulation of NMDA receptors NMDARs by synaptic activity.  Ref: 7544143 Neuron, 1995
- The most intense GluR2 4 immunoreactivity was observed in the target structures of mossy fibers, thus indicating that GluR2 4  **AMPA**  subunits may be involved in NMDA independent synaptic transmission pathways and long term potentiation  [ **LTP** ] .  Ref: 7552321 Brain Res, 1995
- The role of these different modulatory sites of the NMDA receptor and their relation with  **LTP**  are reviewed with a particular attention to the redox site which seems to be a selective target to distinguish between  **AMPA**  and NMDA  **LTP** .  Ref: 7775969 J Neurobiol, 1995

**Parallel relationship** (e.g. studied together, co-existance, homology, etc.)

- Tetanic stimulation yielded a long term potentiation  [ **LTP** ]   **LTP**  of the mixed  **AMPA**  NMDA receptor mediated population excitatory postsynaptic potentials.  Ref: 7551191 Eur J Neurosci, 1995
- A tentative mechanism for this modification, expressed as equal changes of  **AMPA**  and N methyl D aspartate receptor mediated components of the excitatory postsynaptic potential, is an alteration in transmitter release, while the initial asymmetric part of long term potentiation  [ **LTP** ]  indicates involvement of an additional short term modification.  Ref: 8577363 Neuroscience, 1995
- These silent synapses acquire  **AMPA**  type responses following  **LTP**  induction.  Ref: 7760933 Nature, 1995
- Simultaneous expression of long term depression of NMDA and long term potentiation  [ **LTP** ]  of  **AMPA**  receptor mediated synaptic responses in the CA1 area of the kainic acid lesioned hippocampus.  Ref: 7551191 Eur J Neurosci, 1995
- We have tested, in CA1 hippocampal slices, the hypothesis that the expression of long term potentiation  [ **LTP** ]   **LTP**  by alpha amino 3 hydroxy 5 methyl 4 isoxazolepropionic acid  **AMPA**  and or N methyl D aspartate NMDA receptors depends on the degree of NMDA receptors activation during the tetanus.  Ref: 8747197 J Neurophysiol, 1995
- Expression of  **LTP**  by  **AMPA**  and or NMDA receptors is determined by the extent of NMDA receptors activation during the tetanus.  Ref: 8747197 J Neurophysiol, 1995
- Using extracellular recording techniques in the CA1 region of the rat hippocampus, we have evaluated the effects of the redox reagents O dithiobis 2 nitrobenzoic acid DTNB and tris carboxyethyl phosphine TCEP on long term potentiation  [ **LTP** ]   **LTP**  expressed by alpha amino 3 hydroxy 5 methyl isoxazole 4 propionic acid  **AMPA**  and N methyl D aspartate NMDA receptors.  Ref: 7666170 J Neurophysiol, 1995
- synapses expressing only NMDA N methyl D aspartate receptors before potentiation are induced by  **LTP**  to express functional  **AMPA**  alpha amino 3 hydroxy 5 methyl 4 isoxazoleproprionate receptors.  Ref: 7760933 Nature, 1995
- NMDA redox site modulates long term potentiation  [ **LTP** ]  of NMDA but not of  **AMPA**  receptors.  Ref: 7813559 Eur J Pharmacol, 1994
- NBQX, a selective antagonist of the  **AMPA**  receptor, affects neither field potentials nor long term potentiation  [ **LTP** ]  in vivo.  Ref: 7552367 Brain Res, 1995
- These neurons could be subdivided into two populations on the basis of whether or not there was  **LTP**  n = 9, or only STP n = 6, of the  **AMPA**  receptor mediated component.  Ref: 7730988 J Physiol, 1995
- These data are consistent with the hypothesis that the movement of  **AMPA**  receptors between cellular compartments in the postsynaptic neurone could constitute one mechanism underlying long term potentiation  [ **LTP** ]  in the hippocampus.  Ref: 7545519 Br J Pharmacol, 1995
- In neonatal synaptoneurosomes, the effect of melittin on 3H  **AMPA**  binding was significantly reduced when compared to adult synaptoneurosomes, an effect which is consistent with the observation that  **LTP**  is not present in very young animals.  Ref: 7743208 Brain Res, 1995
- In contrast, these studies have suggested that mossy fibre synapses activate primarily or exclusively alpha amino 3 hydroxy 5 methyl 4 isoxazolepropionic acid  **AMPA**  receptors and, indeed, these synapses express a form of  **LTP**  that is entirely independent of NMDA receptors.  Ref: 7617037 Nature, 1995
- During alternating induction of long term depression and long term potentiation  [ **LTP** ] , the  **AMPA**  and N methyl D aspartate receptor mediated components could both be repeatedly regulated up and down.  Ref: 8577363 Neuroscience, 1995
- Tetanic stimulation in control media evoked a statistically identical long term potentiation  [ **LTP** ]   **LTP**  of both the  **AMPA**  and NMDA receptor mediated components of the dual component EPSC AM PAR and NMDAR EPSCs, as shown by a similar percentage increase in both components when measured at a holding potential of 30 mV, and also by an identical time course of the pre and post  **LTP**  induced EPSC at 30 mV and 70 mV.  Ref: 7891148 J Neurosci, 1995
- Administration of the phospholipase A2 PLA2 inhibitor bromophenacyl bromide BPB prior to potassium application prevented  **LTP**  formation as well as the changes in paired pulse facilitation and 3H  **AMPA**  binding that characterized this type of potentiation.  Ref: 7874236 Hippocampus, 1994
- The results indicate that activation of endogenous phospholipases may be an important mechanism in the regulation of  **AMPA**  receptor properties in  **LTP** .  Ref: 7743208 Brain Res, 1995
- This suggests that tetanic stimulation produced  **LTP**  of  **AMPA**  and LTD of NMDA receptor mediated responses simultaneously.  Ref: 7551191 Eur J Neurosci, 1995
