# Supplementary material for: Content-rich biological network constructed by mining PubMed abstracts
Source: BMC Bioinformatics. 2004 Oct 8;5:147. doi: 10.1186/1471-2105-5-147 (PMC528731; doi:10.1186/1471-2105-5-147)
Supplement: Additional File 5 — The original Chilibot query results of the term "long-term potentiation (LTP)" and 22 other terms, limiting the latest references analyzed to the years 1990, 1995, 2000, and 2004. [file 1471-2105-5-147-S5.bz2 › chilibotAdditionalFile5/ltp1995/html/LTP_TAU.html]

 


 **LTP** and **TAU** 
  
Found 3 abstracts in PubMed,  **3 abstracts were retrieved and analyzed**.  


---

 Search Google  |
 PDF files only 
|  EDU domain only 

---

**Interactive relationship** (e.g. stimulation, inhibition, etc)

**Parallel relationship** (e.g. studied together, co-existance, homology, etc.)

- Before and 20 min to 1 h after inducing  **LTP** , we attempted to measure the mean excitatory postsynaptic potential EPSP amplitude, intrasomatic current voltage relationship to a step RN or alpha function AN current waveform, membrane time constant  **tau**  m, spike threshold T50, peak excitatory postsynaptic current amplitude IP, synaptic conductance increase delta G, and synaptic reversal potential VR.  Ref: 3958783 J Neurophysiol, 1986
