# Supplementary material for: Content-rich biological network constructed by mining PubMed abstracts
Source: BMC Bioinformatics. 2004 Oct 8;5:147. doi: 10.1186/1471-2105-5-147 (PMC528731; doi:10.1186/1471-2105-5-147)
Supplement: Additional File 5 — The original Chilibot query results of the term "long-term potentiation (LTP)" and 22 other terms, limiting the latest references analyzed to the years 1990, 1995, 2000, and 2004. [file 1471-2105-5-147-S5.bz2 › chilibotAdditionalFile5/ltp1995/html/LTP_ZIF268.html]

 


 **LTP** and **ZIF268** 
  
Found 4 abstracts in PubMed,  **4 abstracts were retrieved and analyzed**.  


---

 Search Google  |
 PDF files only 
|  EDU domain only 

---

**Interactive relationship** (e.g. stimulation, inhibition, etc)

- The extent of  **LTP**  saturation in fascia dentata produced by the standard chronic  **LTP**  stimulation protocol was assessed both electrophysiologically and through the use of an anatomical marker activation of the immediate early gene  **zif268** .  Ref: 7931545 J Neurosci, 1994

- :-)

  **Parallel relationship** (e.g. studied together, co-existance, homology, etc.)

  - In contrast, administration of maximal electroconvulsive shock led to robust  **zif268**  activation throughout the hippocampus, enhancement of synaptic responses, occlusion of  **LTP**  produced by discrete high frequency stimulation, and spatial learning deficits in the water task.  Ref: 7931545 J Neurosci, 1994
