# Supplementary material for: Content-rich biological network constructed by mining PubMed abstracts
Source: BMC Bioinformatics. 2004 Oct 8;5:147. doi: 10.1186/1471-2105-5-147 (PMC528731; doi:10.1186/1471-2105-5-147)
Supplement: Additional File 5 — The original Chilibot query results of the term "long-term potentiation (LTP)" and 22 other terms, limiting the latest references analyzed to the years 1990, 1995, 2000, and 2004. [file 1471-2105-5-147-S5.bz2 › chilibotAdditionalFile5/ltp1995/html/LTP_CAMKII.html]

 


 **LTP** and **CAMKII** 
  
Found 30 abstracts in PubMed,  **30 abstracts were retrieved and analyzed**.  


---

 Search Google  |
 PDF files only 
|  EDU domain only 

---

**Interactive relationship** (e.g. stimulation, inhibition, etc)

- Mice expressing the mutant form of the kinase show an increased level of calcium independent  **CaMKII**  activity similar to that seen following  **LTP** .  Ref: 7781066 Cell, 1995
- These results suggest that postsynaptic  **CaMKII**  plays a role in the induction of  **LTP**  LTD in visual cortex.  Ref: 1320422 Neuroreport, 1992
- The  **CaMKII**  Asp 286 mice show normal  **LTP**  at high frequency stimulation, but in the 5 10 Hz range, they show a shift in the frequency response curve favoring LTD.  Ref: 7781067 Cell, 1995
- Evidently, activity of both PKC and  **CaMKII**  is somehow necessary for the postsynaptic induction of  **LTP** .  Ref: 1327679 Ciba Found Symp, 1992
- Thus both postsynaptic PKC and  **CaMKII**  are required for the induction of  **LTP**  and a presynaptic protein kinase appears to be necessary for the expression of  **LTP** .  Ref: 2549638 Science, 1989
- In the next 2 h, memory and  **LTP**  are accompanied by an enhancement of the activity of calcium calmodulin dependent protein kinase II  [ **CAMKII** ]  and of protein kinase C, and are blocked by inhibitors of these enzymes.  Ref: 7958619 FASEB J, 1994
- In the next 1 3 h, memory and  **LTP**  are accompanied by enhanced activity of protein kinases and are blocked by specific inhibitors of calcium calmodulin dependent protein kinase II  [ **CAMKII** ]  and protein kinase C.  Ref: 7663877 Neurobiol Learn Mem, 1995
- Simultaneously, recorded field potentials which were derived from neurons with intact  **CaMKII**  showed  **LTP** .  Ref: 1320422 Neuroreport, 1992
- The alpha calcium calmodulin kinase II alpha  **CaMKII**  is required for long term potentiation  [ **LTP** ]  in the CA1 region of the hippocampus.  Ref: 7695905 Neuron, 1995
- These results, together with previous studies, suggest that postsynaptic  **CaMKII**  activity is necessary and sufficient to generate  **LTP** .  Ref: 7997883 Science, 1994
- **CaMKII**  regulates the frequency response function of hippocampal synapses for the production of both LTD and  **LTP** .  Ref: 7781066 Cell, 1995
- calcium calmodulin dependent protein kinase II  **CaMKII**  and protein kinase C PKC may play pivotal roles in the different phases of the expression of  **LTP** .  Ref: 8063002 Int J Biochem, 1994
- Involvement of protein phosphorylation in  **LTP**  has been widely proposed, with protein kinase C PKC and calcium calmodulin kinase type II  **CaMKII**  as leading candidates.  Ref: 2847049 Nature, 1988
- The alpha  **CaMKII**  is highly enriched in postsynaptic densities of hippocampus and neocortex and may be involved in the regulation of long term potentiation  [ **LTP** ]   **LTP** .  Ref: 1378648 Science, 1992
- Calcium calmodulin dependent protein kinase II  [ **CAMKII** ]  CamK II is a major neuronal protein which plays a significant role in the cellular process of long term potentiation  [ **LTP** ]   **LTP** , and vesicular release of neurotransmitters.  Ref: 7538570 J Neurosci, 1995
- Induction of  **LTP**  is blocked by intracellular delivery of H 7, a general protein kinase inhibitor, or PKC 19 31, a selective protein kinase C PKC inhibitor, or  **CaMKII**  273 302, a selective inhibitor of the multifunctional calcium calmodulin dependent protein kinase  **CaMKII** .  Ref: 2549638 Science, 1989
- **CaMKII**  phosphorylates a wide range of neuronal proteins in vitro, presumably reflecting its involvement in the regulation of diverse functions such as postsynaptic responses e.g. long term potentiation  [ **LTP** ] , neurotransmitter synthesis and exocytosis, cytoskeletal interactions and gene transcription.  Ref: 1338943 Neurochem Int, 1992
- Calcium calmodulin dependent protein kinase II  [ **CAMKII** ]  CaM kinase II is the most abundant protein kinase in the brain and is believed to play an important role in the regulation of synaptic transmission, long term potentiation  [ **LTP** ]  and other forms of neuronal plasticity.  Ref: 7820630 Brain Res, 1994
- Inhibition of postsynaptic PKC or  **CaMKII**  blocks induction but not expression of  **LTP** .  Ref: 2549638 Science, 1989
- Strikingly, established  **LTP**  was not suppressed by a combination of PKC and  **CaMKII**  blocking peptides, or by intracellular postsynaptic H 7.  Ref: 1327679 Ciba Found Symp, 1992
- Postsynaptic expression of  **CaMKII**  1 290 increased  **CaMKII**  activity, enhanced synaptic transmission, and prevented more potentiation by an  **LTP**  inducing protocol.  Ref: 7997883 Science, 1994

**Parallel relationship** (e.g. studied together, co-existance, homology, etc.)

- To elucidate a role of calcium calmodulin dependent protein kinase II  **CaMKII**  in induction of long term potentiation  [ **LTP** ]   **LTP** , KN 62, a selective inhibitor for  **CaMKII** , was injected into layer 2 3 neurons of sliced visual cortex obtained from young rats.  Ref: 1320422 Neuroreport, 1992
- Here, a constitutively active form of this enzyme,  **CaMKII**  1 290, was introduced into neurons of hippocampal slices with a recombinant vaccinia virus to test the hypothesis that increased postsynaptic activity of this enzyme is sufficient to produce long term synaptic potentiation  **LTP** , a prominent cellular model of learning and memory.  Ref: 7997883 Science, 1994
- Impairment of spatial but not contextual memory in  **CaMKII**  mutant mice with a selective loss of hippocampal  **LTP**  in the range of the theta frequency.  Ref: 7781067 Cell, 1995
- The mice with a mutation in the alpha calcium calmodulin dependent kinase II alpha  **CaMKII** , a synaptic protein enriched in the hippocampus, are appropriate for addressing this issue because the hippocampus of these mice is deficient in  **LTP**  but maintains intact postsynaptic mechanisms.  Ref: 1321493 Science, 1992
- By increasing calcium accumulation during electrical activity and promoting  **CaMKII**  activity, muscarinic input provides parallel reinforcing pathways for the induction of long term potentiation  [ **LTP** ] , an important cellular memory mechanism.  Ref: 1337106 J Neurophysiol, 1992
- A comparison of the potencies of PKC 19 36 and Ala286  **CaMKII**  281 302 in the physiological assay with their Ki values for protein kinase inhibition in vitro indicates that the blockade of induction of  **LTP**  observed for each peptide is attributable to inhibition of PKC.  Ref: 8197132 Proc Natl Acad Sci U S A, 1994
- Earlier experiments have implicated alpha  **CaMKII**  in the regulation or induction of  **LTP** , but no information is available on the possible role of this enzyme in the two other forms of synaptic plasticity, STP and LTD.  Ref: 7953554 Curr Biol, 1994
- Previous studies have used synthetic peptide analogs, corresponding to sequences within the pseudosubstrate domain of protein kinase C PKC or the autoregulatory domain of calcium calmodulin dependent protein kinase II  **CaMKII** , in attempts to define the contribution of each of these protein kinases to induction of long term potentiation  [ **LTP** ]   **LTP** .  Ref: 8197132 Proc Natl Acad Sci U S A, 1994
- Field potential recordings from hippocampal slices taken from mutant mice show that STP and LTD are, like  **LTP** , absent or markedly attenuated in the absence of alpha  **CaMKII** .  Ref: 7953554 Curr Biol, 1994
- Effects of KN 62, a specific inhibitor of calcium calmodulin dependent protein kinase II  [ **CAMKII** ] , on long term potentiation  [ **LTP** ]  in the rat hippocampus.  Ref: 1850502 Neurosci Lett, 1991
- Potentiated transmission and prevention of further  **LTP**  by increased  **CaMKII**  activity in postsynaptic hippocampal slice neurons.  Ref: 7997883 Science, 1994
- We report here that at short time periods 1 6 h after injections of NMDA or sin 1 molsidomine, there is an increase in the levels of the mRNA encoding the alpha subunit of calcium calmodulin dependent protein kinase II  **CaMKII**  alpha, consistent with a report of elevated  **CaMKII**  alpha mRNA in postsynaptic neurons in the CA1 region of the hippocampus following  **LTP**  induction 54.  Ref: 7476022 Brain Res Mol Brain Res, 1995
- Using intracellular delivery to rat CA1 hippocampal neurons, we have determined the relative potency of two protein kinase inhibitor peptides, PKC 19 36 and Ala286  **CaMKII**  281 302, as inhibitors of the induction of  **LTP** .  Ref: 8197132 Proc Natl Acad Sci U S A, 1994
