# Supplementary material for: Content-rich biological network constructed by mining PubMed abstracts
Source: BMC Bioinformatics. 2004 Oct 8;5:147. doi: 10.1186/1471-2105-5-147 (PMC528731; doi:10.1186/1471-2105-5-147)
Supplement: Additional File 5 — The original Chilibot query results of the term "long-term potentiation (LTP)" and 22 other terms, limiting the latest references analyzed to the years 1990, 1995, 2000, and 2004. [file 1471-2105-5-147-S5.bz2 › chilibotAdditionalFile5/ltp1995/html/LTP_ACTIN.html]

 


 **LTP** and **ACTIN** 
  
Found 8 abstracts in PubMed,  **8 abstracts were retrieved and analyzed**.  


---

 Search Google  |
 PDF files only 
|  EDU domain only 

---

**Interactive relationship** (e.g. stimulation, inhibition, etc)

**Parallel relationship** (e.g. studied together, co-existance, homology, etc.)

- **Actin**  in synaptic cytoskeleton during long term potentiation  [ **LTP** ]  in hippocampal slices.  Ref: 1811261 Acta Histochem Suppl, 1991
- **Actin**  study of the synapses of surviving hippocampal slices during long term potentiation  [ **LTP** ]  .  Ref: 3413824 Tsitologiia, 1988
