# Supplementary material for: Content-rich biological network constructed by mining PubMed abstracts
Source: BMC Bioinformatics. 2004 Oct 8;5:147. doi: 10.1186/1471-2105-5-147 (PMC528731; doi:10.1186/1471-2105-5-147)
Supplement: Additional File 5 — The original Chilibot query results of the term "long-term potentiation (LTP)" and 22 other terms, limiting the latest references analyzed to the years 1990, 1995, 2000, and 2004. [file 1471-2105-5-147-S5.bz2 › chilibotAdditionalFile5/ltp1995/html/SYNAPSIN I_TAU.html]

 


 **SYNAPSIN I** and **TAU** 
  
Found 10 abstracts in PubMed,  **10 abstracts were retrieved and analyzed**.  


---

 Search Google  |
 PDF files only 
|  EDU domain only 

---

**Interactive relationship** (e.g. stimulation, inhibition, etc)

**Parallel relationship** (e.g. studied together, co-existance, homology, etc.)

- The phosphatase was active on many substrates, including p nitrophenyl phosphate, phosphotyrosine, phosphothreonine, phosphorylase a, myelin basic protein, histones, type 1 phosphatase inhibitor 2, microtubule  **tau**  protein, and  **synapsin I** .  Ref: 2553048 J Protein Chem, 1989
- The enzyme phosphorylated smooth muscle myosin light chain,  **synapsin I** , microtubule associated protein 2,  **tau**  protein, myelin basic protein, histone H1, and tyrosine hydroxylase in a calcium calmodulin dependent manner, suggesting that the enzyme is a multifunctional calmodulin dependent protein kinase capable of phosphorylating a large number of substrates.  Ref: 1309765 J Biol Chem, 1992
- Phalloidin staining and immunohistochemistry showed that the neuroblast was richer in F actin, beta tubulin, MAP1, MAP2,  **tau** , calspectin, and  **synapsin I**  than the matrix cell.  Ref: 1586571 Arch Histol Cytol, 1992
- The immunocytochemical features of the indusium griseum IG were compared with the corresponding hippocampus in 5 patients with Alzheimer s disease AD and 5 age matched nondemented individuals using antibodies against beta amyloid, the A68 protein Alz 50 antibody,  **tau** , ubiquitin and  **synapsin I** . beta Amyloid positive plaques were prominent in the AD hippocampus but were not present in the IG.  Ref: 1328542 J Neurol Sci, 1992
- Phosphorylation of additional phosphoproteins, including  **tau**  proteins,  **Synapsin I** , and GAP 43, was not affected by prenatal alcohol exposure.  Ref: 8216886 AlcoholAlcohol, 1992
- Autophosphorylation of the kinase slightly decreased or did not change its activities towards substrates of the first group myosin light chain of chicken gizzard,  **synapsin I** ,  **tau**  factor and microtubule associated protein 2.  Ref: 1648640 Jpn J Pharmacol, 1991
- **Tau**  proteins were also identified in synapses by immunofluorescent double staining with  **synapsin I** , located in the pinceau around the Purkinje cells, and in glomeruli.  Ref: 8275333 Brain Res Bull, 1994
