# Supplementary material for: Content-rich biological network constructed by mining PubMed abstracts
Source: BMC Bioinformatics. 2004 Oct 8;5:147. doi: 10.1186/1471-2105-5-147 (PMC528731; doi:10.1186/1471-2105-5-147)
Supplement: Additional File 5 — The original Chilibot query results of the term "long-term potentiation (LTP)" and 22 other terms, limiting the latest references analyzed to the years 1990, 1995, 2000, and 2004. [file 1471-2105-5-147-S5.bz2 › chilibotAdditionalFile5/ltp1995/html/SYNAPSIN I_ZIF268.html]

 


 **SYNAPSIN I** and **ZIF268** 
  
Found 1 abstracts in PubMed,  **1 abstracts were retrieved and analyzed**.  


---

 Search Google  |
 PDF files only 
|  EDU domain only 

---

**Interactive relationship** (e.g. stimulation, inhibition, etc)

**Parallel relationship** (e.g. studied together, co-existance, homology, etc.)

- Regulation of  **synapsin I**  gene expression by the zinc finger transcription factor  **zif268**  egr 1.  Ref: 8195167 J Biol Chem, 1994
- From this data we conclude that the  **synapsin I**  gene is a target of the  **zif268**  transcription factor.  Ref: 8195167 J Biol Chem, 1994
- Here we report that the  **zif268**  egr 1 protein bound in vitro to two sites in the proximal regulatory region of the human  **synapsin I**  gene.  Ref: 8195167 J Biol Chem, 1994
- An analysis of the temporal expression pattern of  **zif268**  egr 1 and  **synapsin I**  during neuronal differentiation of P19 embryonal carcinoma cells revealed that  **zif268**  egr 1 mRNA was induced on day 5 and  **synapsin I**  mRNA on day 8 after retinoic acid treatment.  Ref: 8195167 J Biol Chem, 1994
