# Supplementary material for: Content-rich biological network constructed by mining PubMed abstracts
Source: BMC Bioinformatics. 2004 Oct 8;5:147. doi: 10.1186/1471-2105-5-147 (PMC528731; doi:10.1186/1471-2105-5-147)
Supplement: Additional File 5 — The original Chilibot query results of the term "long-term potentiation (LTP)" and 22 other terms, limiting the latest references analyzed to the years 1990, 1995, 2000, and 2004. [file 1471-2105-5-147-S5.bz2 › chilibotAdditionalFile5/ltp1995/html/SYNAPSIN I_CAMKII.html]

 


 **SYNAPSIN I** and **CAMKII** 
  
Found 14 abstracts in PubMed,  **14 abstracts were retrieved and analyzed**.  


---

 Search Google  |
 PDF files only 
|  EDU domain only 

---

**Interactive relationship** (e.g. stimulation, inhibition, etc)

**Parallel relationship** (e.g. studied together, co-existance, homology, etc.)

- This bundling activity is reduced when  **synapsin I**  is phosphorylated by cAMP dependent protein kinase and virtually abolished when it is phosphorylated by calcium calmodulin dependent protein kinase II  [ **CAMKII** ]  or by both kinases.  Ref: 3104800 NatureNature, 1994
- we propose that calcium entry into the nerve terminal activates calcium calmodulin dependent protein kinase II  [ **CAMKII** ] , which phosphorylates  **synapsin I**  on site II, dissociating it from the vesicles and thereby removing a constraint in the release process.  Ref: 2859595 Proc Natl Acad Sci U S A, 1985
- A  **synapsin I**  like protein and calcium calmodulin dependent protein kinase II  [ **CAMKII** ]  were demonstrated by biochemical and immunochemical techniques to be present in squid nervous tissue.  Ref: 2859595 Proc Natl Acad Sci U S A, 1985
- **Synapsin I** , phosphorylated at sites 2 and 3 by purified calcium calmodulin dependent protein kinase II  [ **CAMKII** ] , bound with a 5 fold lower affinity to the vesicles than did dephospho  **synapsin I** .  Ref: 3087973 J Biol Chem, 1986
- phosphorylation of  **synapsin I**  on sites 2 and 3 by calcium calmodulin dependent protein kinase II  [ **CAMKII** ]  removes this inhibitory effect.  Ref: 2512374 J Neurosci, 1989
- **Synapsin I**  and calcium calmodulin dependent protein kinase II  [ **CAMKII** ]  were pressure injected into the preterminal digit of the squid giant synapse to test directly the possible regulation of neurotransmitter release by these substances.  Ref: 2859595 Proc Natl Acad Sci U S A, 1985
- Thus, the sequences surrounding the four sites that are phosphorylated by calcium calmodulin dependent protein kinase II  [ **CAMKII** ] , namely sites 2 and 3 in rat and bovine  **synapsin I** , exhibit a high degree of homology.  Ref: 3118371 Proc Natl Acad Sci U S A, 1987
- Effects of  **synapsin I**  and calcium calmodulin dependent protein kinase II  [ **CAMKII** ]  on spontaneous neurotransmitter release in the squid giant synapse.  Ref: 1978321 Proc Natl Acad Sci U S A, 1990
- Brief freezing of synaptosomes in the presence of gamma 32P ATP and either the catalytic subunit of cyclic AMP dependent protein kinase or calcium calmodulin dependent protein kinase II  [ **CAMKII** ]  rendered the synaptosomal interior accessible to these agents, as reflected by the phosphorylation of substrate proteins, such as  **synapsin I** , which reside within the nerve terminal.  Ref: 2536075 J Neurochem, 1989
- Conversely, injection of calcium calmodulin dependent protein kinase II  [ **CAMKII** ] , which phosphorylates  **synapsin I**  on site II, increased the rate of rise and amplitude and decreased the latency of the postsynaptic potential.  Ref: 2859595 Proc Natl Acad Sci U S A, 1985
- Intraterminal injection of  **synapsin I**  or calcium calmodulin dependent protein kinase II  [ **CAMKII** ]  alters neurotransmitter release at the squid giant synapse.  Ref: 2859595 Proc Natl Acad Sci U S A, 1985
- Among the protein molecules specifically located in presynaptic terminals,  **synapsin I**  and calcium calmodulin dependent protein kinase II  [ **CAMKII** ]  CaM kinase II have been shown to modulate evoked transmitter release in the squid giant synapse.  Ref: 1978321 Proc Natl Acad Sci U S A, 1990
- These included several forms of  **synapsin I** , calcium calmodulin dependent protein kinase II  [ **CAMKII** ]  CaM kinase II and avidin.  Ref: 1676419 J Physiol, 1991
- The present study presents evidence, using lesion induced degenerations of pre and postsynaptic neuronal populations in the neostriatum and substantia nigra, that calcium calmodulin dependent protein kinase II  [ **CAMKII** ] , as measured both by autophosphorylation of enzyme subunits and by  **synapsin I**  kinase activity, is present in high concentrations in several populations of presynaptic terminals.  Ref: 2545012 Synapse, 1989
