# Supplementary material for: Content-rich biological network constructed by mining PubMed abstracts
Source: BMC Bioinformatics. 2004 Oct 8;5:147. doi: 10.1186/1471-2105-5-147 (PMC528731; doi:10.1186/1471-2105-5-147)
Supplement: Additional File 5 — The original Chilibot query results of the term "long-term potentiation (LTP)" and 22 other terms, limiting the latest references analyzed to the years 1990, 1995, 2000, and 2004. [file 1471-2105-5-147-S5.bz2 › chilibotAdditionalFile5/ltp1995/html/SYNAPSIN I_ACTIN.html]

 


 **SYNAPSIN I** and **ACTIN** 
  
Found 34 abstracts in PubMed,  **30 abstracts were retrieved and analyzed**.  


---

 Search Google  |
 PDF files only 
|  EDU domain only 

---

**Interactive relationship** (e.g. stimulation, inhibition, etc)

- These results, demonstrating an interaction of  **synapsin I**  with  **actin**  in vitro, support the possibility that  **synapsin I**  is involved in clustering of synaptic vesicles at the presynaptic terminal.  Ref: 3104800 NatureNature, 1989
- To elucidate the structural basis for the interactions between  **synapsin I**  and F  **actin**  and how it relates to other characteristics of  **synapsin I** , we have performed a structure function analysis of fragments of  **synapsin I**  produced by cysteine specific cleavage with 2 nitro 5 thiocyanobenzoic acid.  Ref: 2497104 J Cell Biol, 1989
- **Synapsin I**  plays an important role in the regulation of neurotransmitter release, since it binds to synaptic vesicles and to the cytoskeleton, and it bundles F  **actin**  and microtubules.  Ref: 2129153 Biochem Int, 1990
- **Synapsin I**  is a neuron specific phosphoprotein that binds to small synaptic vesicles and  **actin**  filaments in a phosphorylation dependent fashion.  Ref: 1671174 Proc Natl Acad Sci U S A, 1991
- a comparison of their structure and interactions with spectrin binding proteins ankyrin,  **actin** ,  **synapsin I** , amelin, and calmodulin.  Ref: 3048888 CRC Crit Rev Biochem, 1988
- As purified from brain homogenates,  **synapsin I**  decreases the steady state viscosity of solutions containing F  **actin** , enhances the sedimentation of  **actin** , and bundles  **actin**  filaments.  Ref: 3115996 J Cell Biol, 1987
- The fraction of total  **actin**  polymerized by  **synapsin I**  strongly depends on the  **synapsin I**   **actin**  ratio.  Ref: 8305899 J Physiol Paris, 1993
- Using video enhanced microscopy, we have now obtained experimental evidence in support of this model the presence of dephosphorylated  **synapsin I**  is necessary for synaptic vesicles to bind  **actin** .  Ref: 7876313 J Cell Biol, 1995
- **Synapsin I**  is a synaptic vesicle specific phosphoprotein which is able to bind and bundle  **actin**  filaments in a phosphorylation dependent fashion.  Ref: 1317863 J Biol Chem, 1992
- **Synapsin I**  is a neuron specific phosphoprotein which binds to small synaptic vesicles and  **actin**  in a phosphorylation dependent fashion.  Ref: 8305899 J Physiol Paris, 1993
- Our findings suggest that  **synapsin I**  exerts a control on the physical characteristics of the cytoskeletal network of the nerve terminal and are consistent with the proposed role of  **synapsin I**  in mediating the interaction of synaptic vesicles with  **actin** .  Ref: 8305899 J Physiol Paris, 1993
- **Synapsin I** , which has been demonstrated to bundle F  **actin**  in vitro, has been postulated to regulate neurotransmitter release by cross linking synaptic vesicles to the  **actin**  cytoskeleton.  Ref: 7931311 J Neurochem, 1994
- Interaction of free and synaptic vesicle bound  **synapsin I**  with F  **actin** .  Ref: 1739463 Neuron, 1992
- **Synapsin I**  is a neuron specific phosphoprotein that binds to small synaptic vesicles and F  **actin**  in a phosphorylation dependent fashion.  Ref: 1739463 Neuron, 1992
- The models obtained under various ionic conditions reveal that  **synapsin I**  interacts with  **actin**  in a very complex fashion, sharing some of the properties of classical nucleating proteins but displaying also actions not described previously for other  **actin**  binding proteins.  Ref: 1597463 J Biol Chem, 1992
- Coordinated regulation of  **synapsin I**  interaction with F  **actin**  by calcium calmodulin and phosphorylation inhibition of  **actin**  binding and bundling.  Ref: 7849051 Biochemistry, 1995
- Similar studies conducted with purified tubulin and tubulin immobilized on Sepharose demonstrate that both tubulin and  **actin**  bind at approximately the same sites in the NH2 terminal half of  **synapsin I** .  Ref: 1899024 Biochemistry, 1991
- the cross linking between synaptic vesicles and  **actin**  is specific for the membrane of synaptic vesicles and does not reflect either a non specific binding of membranes to the highly surface active  **synapsin I**  molecule or trapping of vesicles within the thick bundles of  **actin**  filaments.  Ref: 7876313 J Cell Biol, 1995
- The inhibition of organelle movement in axoplasm by  **actin**  binding proteins such as DNase I, gelsolin and  **synapsin I**  has been attributed to their ability to disorganize the microtubule domains where most of the  **actin**  filaments are located.  Ref: 1570018 Nature, 1992
- **Synapsin I**  accelerates the initial rate of  **actin**  polymerization and increases the final steady state levels of polymerized  **actin** .  Ref: 8305899 J Physiol Paris, 1993

**Parallel relationship** (e.g. studied together, co-existance, homology, etc.)

- Under the conditions of our assay 0.45 microM  **synapsin I** , 4 microM F  **actin** , half maximal inhibition of  **actin**  binding and bundling by unphosphorylated  **synapsin I**  was found with 4.3 and 3.7 microM calmodulin, respectively.  Ref: 7849051 Biochemistry, 1995
- **Synapsin I**  is a neuronal phosphoprotein that can bundle  **actin**  filaments in vitro.  Ref: 3125185 J Cell Biochem, 1988
- Two complementary peptide fragments of synapsin generated by 2 nitro 5 thiocyanobenzoic cleavage and which map to opposite ends of the molecule participate in the bundling process, either by binding directly to  **actin**  or by binding to other  **synapsin I**  molecules.  Ref: 3115996 J Cell Biol, 1987
- Rather, these results are consistent with the possibility that dephospho  **synapsin I**  acts by a crosslinking mechanism involving some component s of the cytoskeleton, such as F  **actin** , to create a dense network that restricts organelle movement.  Ref: 2512374 J Neurosci, 1989
- The  **actin**  binding activity of  **synapsin I**  phosphorylated by cAMP dependent protein kinase or by calmodulin dependent protein kinase II showed similar sensitivity to calmodulin inhibition to unphosphorylated  **synapsin I** .  Ref: 7849051 Biochemistry, 1995
- **Synapsin I**  is an  **actin**  bundling protein this activity is controlled by phosphorylation.  Ref: 7849051 Biochemistry, 1995
- Effects of the neuronal phosphoprotein  **synapsin I**  on  **actin**  polymerization.  Ref: 1597463 J Biol Chem, 1992
- An antibody specific for N25 inhibits the  **actin**  binding activity of N25 and the  **actin**  bundling but not the  **actin**  binding activity of intact  **synapsin I** .  Ref: 1899024 Biochemistry, 1991
- In contrast to phosphorylation of sites two and three in intact  **synapsin I** , which abolishes F  **actin**  bundling activity, phosphorylation of these sites in the middle tail fragment failed to abolish this activity.  Ref: 2497104 J Cell Biol, 1989
- The effect was observed at both low and high ionic strength, was specific for  **synapsin I** , and was still present when polymerization was triggered by F  **actin**  seeds.  Ref: 1317863 J Biol Chem, 1992
- A domain of  **synapsin I**  involved with  **actin**  bundling shares immunologic cross reactivity with villin.  Ref: 3125185 J Cell Biochem, 1988
- We have found that dephosphorylated  **synapsin I**  induces a dose dependent increase in the number of  **actin**  filaments, which at high ionic strength is abolished by  **synapsin I**  phosphorylation.  Ref: 1739463 Neuron, 1992
- Previously, we identified an  **actin**  binding domain in the NH2 terminal 25 kDa fragment N25 generated by 2 nitro 5 thiocyanobenzoic acid NTCB cleavage of  **synapsin I**  and found that a complementary COOH terminal 52 kDa portion of the molecule N52 contained either a second  **actin**  binding site or a site of self association Petrucci, T.  Ref: 1899024 Biochemistry, 1991
- We found that dephosphorylated  **synapsin I**  accelerates the initial rate of  **actin**  polymerization and decreases the rate of filament elongation.  Ref: 1317863 J Biol Chem, 1992
- **synapsin I**  is able to promote  **actin**  polymerization and bundling of  **actin**  filaments in the presence of synaptic vesicles.  Ref: 7876313 J Cell Biol, 1995
- **Synapsin I**  bundles F  **actin**  in a phosphorylation dependent manner.  Ref: 3104800 NatureNature, 1995
- We report here the ability of the dephospho form of  **synapsin I**  to bundle F  **actin** .  Ref: 3104800 NatureNature, 1995
- These data support the view that  **synapsin I**  is involved in the regulation of the dynamics of the  **actin**  based network during the exo endocytotic cycle.  Ref: 1739463 Neuron, 1992
- By using fluorescence resonance energy transfer between purified components labeled with fluorescent probes, we now show that the binding of  **synapsin I**  to  **actin**  is a rapid phenomenon.  Ref: 8365471 FEBS Lett, 1993
- Fluorescence approaches to the study of the  **actin**  nucleating and bundling activities of  **synapsin I** .  Ref: 8305899 J Physiol Paris, 1993
- **Synapsin I**  is also able to interact with  **actin**  filaments in a phosphorylation dependent manner.  Ref: 2517594 Cell Biol Int Rep, 1989
- Identification of a new 84 82 kDa calmodulin binding protein, which also interacts with  **actin**  filaments, tubulin and spectrin, as  **synapsin I** .  Ref: 3030806 FEBS Lett, 1987
- We propose that  **synapsin I**  links synaptic vesicles to  **actin**  filaments in the presynaptic nerve terminal.  Ref: 2117454 Bioessays, 1990
- Dose response curves indicated that synapsin IIa was more potent than  **synapsin I**  in bundling  **actin**  filaments.  Ref: 7931311 J Neurochem, 1994
- We have analyzed the ability of  **synapsin I**  to interact with  **actin**  monomers and filaments using purified proteins derivatized with fluorescent probes.  Ref: 8305899 J Physiol Paris, 1993
- Domain C, the central homologous domain implicated in the binding of  **synapsin I**  to  **actin**  and to synaptic vesicles, is divided into nine exons.  Ref: 2110562 J Biol Chem, 1990
- **Synapsin I**  is a nerve terminal phosphoprotein which interacts with synaptic vesicles and  **actin**  in a phosphorylation dependent manner.  Ref: 8365471 FEBS Lett, 1993
- **Synapsin I** , a neuron specific phosphoprotein interacting with small synaptic vesicles and F  **actin** .  Ref: 2517594 Cell Biol Int Rep, 1989
- Along the axon, phosphorylation could be functional in preventing  **synapsin I**  from forming, with  **actin** , a dense meshwork that would restrict organelle movement.  Ref: 1715393 J Neurosci, 1991
- These observations suggest that  **synapsin I**  has a phosphorylation dependent nucleating effect on  **actin**  polymerization.  Ref: 1317863 J Biol Chem, 1992
- **Synapsin I**  an  **actin**  bundling protein under phosphorylation control.  Ref: 3115996 J Cell Biol, 1987
- Because of these properties, it has been hypothesized that  **synapsin I**  acts as a dynamic link between synaptic vesicles an the  **actin**  meshwork of the nerve terminal, thereby modulating the release of neurotransmitter.  Ref: 2517594 Cell Biol Int Rep, 1989
- Here we show that calmodulin in the presence of calcium is a competitive inhibitor of both  **actin**  binding and bundling by  **synapsin I** .  Ref: 7849051 Biochemistry, 1995
- Phalloidin staining and immunohistochemistry showed that the neuroblast was richer in F  **actin** , beta tubulin, MAP1, MAP2, tau, calspectin, and  **synapsin I**  than the matrix cell.  Ref: 1586571 Arch Histol Cytol, 1992
- We have visualized the  **actin**  bundling activity of  **synapsin I**  using a non perturbing method, video enhanced microscopy of fluoresceinated  **synapsin I**  and  **actin**  filaments.  Ref: 8305899 J Physiol Paris, 1993
- Effects of the neuronal phosphoprotein  **synapsin I**  on  **actin**  polymerization.  Ref: 1317863 J Biol Chem, 1992
- We have examined the interaction of purified phosphorylated and unphosphorylated bovine and human  **synapsin I**  with tubulin and  **actin**  filaments, using cosedimentation, viscometric, electrophoretic, and morphologic assays.  Ref: 3115996 J Cell Biol, 1987
- the ability to cross link synaptic vesicles and  **actin**  is specific for  **synapsin I**  and is not shared by other basic proteins.  Ref: 7876313 J Cell Biol, 1995
- Dephosphorylated  **synapsin I**  anchors synaptic vesicles to  **actin**  cytoskeleton an analysis by videomicroscopy.  Ref: 7876313 J Cell Biol, 1995
- Binding of  **synapsin I**  to  **actin**  can also be demonstrated when synaptic vesicles are present in the medium and appears to be modulated by ionic strength and  **synapsin I**  phosphorylation.  Ref: 8365471 FEBS Lett, 1993
- In the present paper we have analyzed the effects of  **synapsin I**  on the kinetics of  **actin**  polymerization and their modulation by site specific phosphorylation of  **synapsin I** .  Ref: 1317863 J Biol Chem, 1992
- Computer modeling of  **synapsin I**  binding to synaptic vesicles and F  **actin**  implications for regulation of neurotransmitter release.  Ref: 1671174 Proc Natl Acad Sci U S A, 1991
- Rapid binding of  **synapsin I**  to F and G  **actin** .  Ref: 8365471 FEBS Lett, 1993
- In this study, we have examined whether or not calmodulin can regulate one of the activities of  **synapsin I** , namely, its interaction with F  **actin** .  Ref: 7849051 Biochemistry, 1995
- In conclusion, three domains of  **synapsin I**  appear to be involved in F  **actin**  binding and bundling.  Ref: 2497104 J Cell Biol, 1989
- A 51 54 kD middle tail fragment retained the F  **actin**  binding and bundling activity of  **synapsin I** , but the isolated tail fragment did not retain either activity.  Ref: 2497104 J Cell Biol, 1989
- It has been hypothesized that dephosphorylated  **synapsin I**  inhibits neurotransmitter release either by forming a cage around synaptic vesicles cage model or by anchoring them to the F  **actin**  cytoskeleton of the nerve terminal crosslinking model.  Ref: 1671174 Proc Natl Acad Sci U S A, 1991
- Brain beta spectrin contains three structural domains and we suggest the position of several functional domains including f  **actin** ,  **synapsin I** , ankyrin and spectrin self association sites.  Ref: 8479293 Brain Res Mol Brain Res, 1993
- Dephosphorylated  **synapsin I**  was also able to induce  **actin**  polymerization and bundle formation in the absence of KCl and MgCl2.  Ref: 1317863 J Biol Chem, 1992
- Characterization of  **synapsin I**  fragments produced by cysteine specific cleavage a study of their interactions with F  **actin** .  Ref: 2497104 J Cell Biol, 1989
- **Synapsin I**  appears to bind G  **actin**  with a very high stoichiometry 1 2 4, and the complex behaves as an F  **actin**  nucleus, producing  **actin**  filaments under conditions where spontaneous polymerization is negligible.  Ref: 1597463 J Biol Chem, 1992
- It is hypothesized that, in the intact molecule, the two NH2 terminal domains contribute to a single high affinity  **actin**  and or tubulin binding site in the globular head region of  **synapsin I** , while the third  **actin**  binding domain constitutes the topographically distinct site required for the  **actin**  bundling activity of the native molecule.  Ref: 1899024 Biochemistry, 1991
- In vivo,  **synapsin I**  may link small synaptic vesicles to the  **actin**  based cortical cytoskeleton, and coordinate their availability for release in a Ca dependent fashion.  Ref: 3115996 J Cell Biol, 1987
