# Supplementary material for: Content-rich biological network constructed by mining PubMed abstracts
Source: BMC Bioinformatics. 2004 Oct 8;5:147. doi: 10.1186/1471-2105-5-147 (PMC528731; doi:10.1186/1471-2105-5-147)
Supplement: Additional File 5 — The original Chilibot query results of the term "long-term potentiation (LTP)" and 22 other terms, limiting the latest references analyzed to the years 1990, 1995, 2000, and 2004. [file 1471-2105-5-147-S5.bz2 › chilibotAdditionalFile5/ltp1995/html/SYNAPSIN I_SYNAPTOTAGMIN.html]

 


 **SYNAPSIN I** and **SYNAPTOTAGMIN** 
  
Found 6 abstracts in PubMed,  **6 abstracts were retrieved and analyzed**.  


---

 Search Google  |
 PDF files only 
|  EDU domain only 

---

**Interactive relationship** (e.g. stimulation, inhibition, etc)

**Parallel relationship** (e.g. studied together, co-existance, homology, etc.)

- The conditions used indicate that  **synaptotagmin** , as  **synapsin I** , is phosphorylated by calcium calmodulin dependent protein kinase II.  Ref: 8381369 FEBS Lett, 1993
- At both 4 and 12 DIC, the synaptic proteins rab3a,  **synapsin I** , and  **synaptotagmin**  were present in hippocampal neurons, but the subcellular distribution changed from one in which immunoreactivity was initially distributed within soma and neurites to a punctate varicose appearance.  Ref: 7965045 J Neurosci, 1994
- High magnification revealed that  **synaptotagmin**  I like immunoreactivity was mainly distributed in a fine granular pattern, but large, brightly fluorescent granules which were not labelled by anti synaptophysin or anti  **synapsin I**  were occasionally observed.  Ref: 7534885 Neuroscience, 1994
- No  **synaptotagmin**  I like immunoreactivity was found in large axons, while accumulations of the synaptic vesicle proteins synaptophysin and  **synapsin I**  were found in all types of axons.  Ref: 7534885 Neuroscience, 1994
