# Supplementary material for: Content-rich biological network constructed by mining PubMed abstracts
Source: BMC Bioinformatics. 2004 Oct 8;5:147. doi: 10.1186/1471-2105-5-147 (PMC528731; doi:10.1186/1471-2105-5-147)
Supplement: Additional File 5 — The original Chilibot query results of the term "long-term potentiation (LTP)" and 22 other terms, limiting the latest references analyzed to the years 1990, 1995, 2000, and 2004. [file 1471-2105-5-147-S5.bz2 › chilibotAdditionalFile5/ltp1995/html/SYNAPSIN I_SYNAPTOPHYSIN.html]

 


 **SYNAPSIN I** and **SYNAPTOPHYSIN** 
  
Found 40 abstracts in PubMed,  **30 abstracts were retrieved and analyzed**.  


---

 Search Google  |
 PDF files only 
|  EDU domain only 

---

**Interactive relationship** (e.g. stimulation, inhibition, etc)

**Parallel relationship** (e.g. studied together, co-existance, homology, etc.)

- The clear vacuoles, which are negative for  **synaptophysin**  and  **synapsin I** , are considered to be related to the retrieval of the limiting membranes of the released neurosecretory granules.  Ref: 1908264 Arch Histol Cytol, 1991
- No synaptotagmin I like immunoreactivity was found in large axons, while accumulations of the synaptic vesicle proteins  **synaptophysin**  and  **synapsin I**  were found in all types of axons.  Ref: 7534885 Neuroscience, 1994
- **Synapsin I**  and  **Synaptophysin**  are selectively localized in axonal endings of CNS neurons where they are associated with small synaptic vesicle membranes.  Ref: 1902873 J Neurosci, 1991
- A decrease in the synaptic protein  **synapsin I** ,  **synaptophysin**  content was detected at 10 8 M of TMT in synaptosomal fractions.  Ref: 7496810 Brain Res, 1995
- A procedure for the simultaneous purification of  **synapsin I**  and  **synaptophysin**  from calf brain was developed.  Ref: 1522178 J Chromatogr, 1992
- Second, using this system, we examined localization of  **synapsin I**  and  **synaptophysin**  in nerve endings after electrical stimulation.  Ref: 7820876 Cell Struct Funct, 1994
- Redistribution of  **synapsin I**  and  **synaptophysin**  in response to electrical stimulation in the rat neurohypophysial nerve endings.  Ref: 7820876 Cell Struct Funct, 1994
- This study describes the immunocytochemical distribution of five neuropeptides calcitonin gene related peptide CGRP, enkephalin, galanin, somatostatin, and substance P, three neuronal markers neurofilament triplet proteins, neuron specific enolase NSE, and protein gene product 9.5, and two synaptic vesicle associated proteins  **synapsin I**  and  **synaptophysin**  in the spinal cord and dorsal root ganglia of adult and newborn dogs.  Ref: 1862758 Am J Anat, 1991
- Other proteins including the synaptic vesicle proteins  **synapsin I**  and  **synaptophysin**  remained unaltered.  Ref: 7605916 Neuroreport, 1995
- **Synapsin I**  and  **Synaptophysin**  expression during ontogenesis of the mouse peripheral vestibular system.  Ref: 1902873 J Neurosci, 1991
- We conclude that  **synapsin I**  is a reliable, sensitive immunohistochemical marker for neuronal neuroendocrine differentiation in human neoplasms and may offer some advantages over  **synaptophysin**  when applied to formalin fixed, paraffin embedded tissues, particularly in the evaluation of primitive neuroectodermal tumors and neuroendocrine tumors.  Ref: 8287627 Clin NeuropatholClin Neuropathol, 1991
- Accumulation of Rab3a was accompanied by accumulation of  **synaptophysin**  and  **synapsin I** , two synaptic vesicle membrane proteins, and accumulation of clathrin light chain.  Ref: 8521869 Eur J Cell Biol, 1995
- The distribution of  **synapsin I**  and  **synaptophysin**  in hippocampal neurons developing in culture.  Ref: 1904480 J Neurosci, 1991
- To understand the dynamics of synaptic vesicles and  **synapsin I** , we have studied the localization of  **synapsin I**  and  **synaptophysin**  in resting and stimulated nerve endings by ultracryomicrotomy and colloidal gold immunocytochemistry.  Ref: 7820876 Cell Struct Funct, 1994
- Immunohistochemistry of  **synapsin I**  and  **synaptophysin**  in human nervous system and neuroendocrine tumors.  Ref: 8287627 Clin NeuropatholClin Neuropathol, 1994
- In adult retina,  **synapsin I**  and  **synaptophysin**  were also mainly localized in synaptic fields and processes but all three proteins showed a distinct pattern of distribution.  Ref: 1453474 J Neurosci Res, 1992
- Influence of spinal cord transection on the presence and axonal transport of CGRP, chromogranin A, VIP,  **synapsin I** , and  **synaptophysin**  like immunoreactivities in rat motor nerve.  Ref: 1281222 J Neurobiol, 1992
- These observations show that the distribution pattern of  **Synapsin I**  and  **Synaptophysin**  in peripheral extensions of vestibular afferent neurons during development is identical to that described in axonal processes of CNS neurons.  Ref: 1902873 J Neurosci, 1991
- Immunocytochemical localization of  **synaptophysin**  protein p38 and  **synapsin I**  in nerve terminals of rat neurohypophysis.  Ref: 1908264 Arch Histol Cytol, 1991
- The distribution of the synaptic vesicle associated phosphoprotein  **synapsin I**  after electrical stimulation of the frog neuromuscular junction was investigated by immunogold labeling and compared with the distribution of the integral synaptic vesicle protein  **synaptophysin** .  Ref: 1463610 Neuron, 1992
- The competence of axons to form presynaptic vesicle clusters in response to contact with the somata or dendrites of mature or immature neurons was determined by immunofluorescent staining for  **synapsin I**  or  **synaptophysin** .  Ref: 7965070 J Neurosci, 1994
- Intense immunoreactivity for both  **synapsin I**  and  **synaptophysin**  was observed in tumors containing well differentiated neurons gangliocytoma, ganglioglioma, neurocytoma.  Ref: 8287627 Clin NeuropatholClin Neuropathol, 1994
- In general, the pattern of expression of  **synapsin I**  mRNA paralleled those encoding other synaptic terminal specific proteins, such as  **synaptophysin** , VAMP 2, and SNAP 25, with noteworthy exceptions.  Ref: 8440778 J Comp Neurol, 1993
- **Synaptophysin**  and  **synapsin I** , the synaptic vesicle associated proteins, were demonstrated immunocytochemically in nerve terminals of the neurohypophysis of rats.  Ref: 1908264 Arch Histol Cytol, 1991
- At early stages of development, before cell cell contact, both  **synapsin I**  and  **synaptophysin**  were preferentially localized in axons, where they were particularly concentrated in the distal axon and growth cone.  Ref: 1904480 J Neurosci, 1991
- High magnification revealed that synaptotagmin I like immunoreactivity was mainly distributed in a fine granular pattern, but large, brightly fluorescent granules which were not labelled by anti  **synaptophysin**  or anti  **synapsin I**  were occasionally observed.  Ref: 7534885 Neuroscience, 1994
- Simultaneous purification of the neuroproteins  **synapsin I**  and  **synaptophysin** .  Ref: 1522178 J Chromatogr, 1992
- Concomitantly, the two synaptic vesicle proteins dropped,  **synaptophysin**  > 50% and  **synapsin I**  > 85%.  Ref: 7783952 Neurosci Lett, 1995
- Some PNETs and neuroendocrine tumors that were immunoreactive for  **synapsin I**  did not stain for  **synaptophysin** .  Ref: 8287627 Clin NeuropatholClin Neuropathol, 1995
- Moreover,  **synapsin I**   **synaptophysin**  immunoreactivity, at birth, was restricted to laminae I II, while in adult dogs, immunostaining was observed in terminal like elements throughout the spinal neuropil.  Ref: 1862758 Am J Anat, 1991
- This protein was demonstrated to be brain specific, and its distribution in various brain regions paralleled the distribution of  **synapsin I**  and  **synaptophysin** .  Ref: 2072093 J Neurochem, 1991
- In parallel, neurotoxic effects were assessed by determining the content of  **synaptophysin**  and  **synapsin I** , both in the total homogenates and in the synaptosomal fraction of the cultures.  Ref: 7496810 Brain Res, 1995
- Furthermore, these vesicle clusters stained positively for two SV associated proteins,  **synapsin I**  and  **synaptophysin** , by EM immunocytochemistry ICC.  Ref: 8583500 J Neurosci Res, 1995
- These results suggest that in the dog spinal cord and dorsal root ganglia, peptide containing pathways complete their development during postnatal life, together with the full expression of NSE and  **synapsin I**   **synaptophysin**  immunoreactivities.  Ref: 1862758 Am J Anat, 1991
- Development of calcitonin gene related peptide, chromogranin A, and synaptic vesicle markers in rat motor endplates, studied using immunofluorescence and confocal laser scanning.The presence of calcitonin gene related peptide and chromogranin A was investigated in the developing rat motor system, using immunofluorescence and confocal laser scanning, and compared with synaptic vesicle markers,  **synaptophysin**  and  **synapsin I** .  Ref: 1518519 Muscle Nerve, 1992
- Indirect immunofluorescence with Texas Red as label was used to investigate the distribution of 3 different groups of immunogens enzymes monoamine oxidase A and B, receptors beta adrenoceptor protein, and synaptic vesicle proteins  **synapsin I**  and  **synaptophysin**  in each cortical slice.  Ref: 7967715 J Neurosci Methods, 1994
- In stimulated terminals they appeared also in the axolemma and its infoldings, which however exhibited a lower  **synapsin I**   **synaptophysin**  ratio with respect to synaptic vesicles at rest.  Ref: 1463610 Neuron, 1992
- In contrast, the protein was found in muscle spindles of young rats and in perivascular terminals, where it co localized with  **synaptophysin**  and  **synapsin I** .  Ref: 7534885 Neuroscience, 1994
- In mature cultures,  **synapsin I**  and  **synaptophysin**  immunoreactivity was concentrated in puncta that were restricted to sites where axons contacted neuronal cell bodies or dendrites.  Ref: 1904480 J Neurosci, 1991
- After 5 days of treatment, cultures were analyzed for the presence of synapses by  **synapsin I**  and  **synaptophysin**  antibody labeling and by electron microscopy.  Ref: 7568106 Proc Natl Acad Sci U S A, 1995
- The recovery obtained was 40 micrograms g in brain for  **synaptophysin**  and 25 micrograms g in brain for  **synapsin I** .  Ref: 1522178 J Chromatogr, 1992
- To evaluate SNAP 25 expression, its distribution was compared to those of the synaptic vesicle associated proteins  **synapsin I**  and  **synaptophysin** .  Ref: 1453474 J Neurosci Res, 1992
- When neurons contacted one another, numerous puncta of  **synapsin I**  and  **synaptophysin**  formed within the first week in culture.  Ref: 1904480 J Neurosci, 1991
- **Synaptophysin**  was finally purified by preparative sodium dodecyl sulphate polyacrylamine gel electrophoresis and  **synapsin I**  by affinity chromatography using a calmodulin Sepharose column.  Ref: 1522178 J Chromatogr, 1992
- **Synapsin I** , synapsin II, and  **synaptophysin**  marker proteins of synaptic vesicles.  Ref: 7903586 Brain Pathol, 1993
- As a first step toward elucidating mechanisms involved in the sorting of synaptic vesicle proteins in neurons, we have used immunofluorescence microscopy to determine the distribution of two synaptic vesicle proteins,  **synapsin I**  and  **synaptophysin** , in hippocampal neurons developing in culture.  Ref: 1904480 J Neurosci, 1991
- Of these latter proteins, one is integral  **synaptophysin**  the other peripheral  **synapsin I**  to the synaptic vesicle membranes.  Ref: 7783952 Neurosci Lett, 1995
- In most cases, immunostaining for  **synapsin I**  was sharper and often more intense than for  **synaptophysin** .  Ref: 8287627 Clin NeuropatholClin Neuropathol, 1995
