# Supplementary material for: Content-rich biological network constructed by mining PubMed abstracts
Source: BMC Bioinformatics. 2004 Oct 8;5:147. doi: 10.1186/1471-2105-5-147 (PMC528731; doi:10.1186/1471-2105-5-147)
Supplement: Additional File 5 — The original Chilibot query results of the term "long-term potentiation (LTP)" and 22 other terms, limiting the latest references analyzed to the years 1990, 1995, 2000, and 2004. [file 1471-2105-5-147-S5.bz2 › chilibotAdditionalFile5/ltp1995/html/PLC_TRKA.html]

 


 **PLC** and **TRKA** 
  
Found 2 abstracts in PubMed,  **2 abstracts were retrieved and analyzed**.  


---

 Search Google  |
 PDF files only 
|  EDU domain only 

---

- J Neurosci Res, 1995   **Specificity of nerve growth factor signaling differential patterns of early tyrosine phosphorylation events induced by NGF, EGF, and bFGF.**.
  The specificity of nerve growth factor NGF action was examined by comparing early tyrosine phosphorylation events induced by NGF, epidermal growth factor EGF , and basic fibroblast growth factor bFGF .
  In PC12 cells, administration of either the differentiation factor NGF or the mitogenic factor EGF led to tyrosine phosphorylation of multiple polypeptides in the 100 110 kDa size range associated with PI 3 kinase.
  However, NGF induced a more prolonged phosphorylation, relative to a transient EGF effect.
  In contrast, the differentiation factor bFGF failed to induce measurable tyrosine phosphorylation of PI 3 kinase associated proteins.
  Similarly, NGF but not bFGF induced marked tyrosine phosphorylation of **PLC** gamma, another early signaling molecule, suggesting that multiple pathways exist for promoting differentiation, and or that these signaling molecules are not essential for differentiation.
  **TrkA** signaling was also compared between PC12 cells and NIH 3T3 cells heterologously expressing **trkA**, where receptor activation promotes mitogenesis.
  In this comparison, significant differences were observed in the tyrosine phosphorylation pattern of PI 3 kinase associated polypeptides, suggesting the existence of cell type specific molecular interactions influencing **trkA** signaling.
  Mechanistically, NGF stimulation of PC12 cells resulted in a weak or possibly indirect association between **trkA** and PI 3 kinase.
  Furthermore, NGF did not appear to activate or substantially alter the overall level of PI 3 kinase activity, raising the possibility that ligand induced phosphorylation may serve instead to relocalize constitutively active PI 3 kinase molecules within the cell.
  Taken together, data presented suggest that the temporal pattern of induced phosphorylation, the nature of induced associations with other phosphoproteins, and cell type specific components may all contribute to the generation of NGF signaling specificity.

  - Prog Clin Biol Res, 1994   **Expression and function of the nerve growth factor receptor TRK A **[TRKA]** in human neuroblastoma cell lines.**.
    Nerve growth factor NGF is known to play a critical role in the differentiation and survival of normal sympathetic neurons through its interaction with a specific cell surface receptor.
    We analyzed ten well characterized neuroblastoma cell lines for the expression and function of endogenous and exogenous p140TRK A, and p75LNGFR.
    Exogenous LNGFR or TRK A **[TRKA]** or both were introduced by transfection into three neuroblastoma cell lines.
    Transfected and untransfected neuroblastoma cell lines were analyzed by Northern analysis as well as tyrosine phosphorylation studies.
    Results indicate that endogenous TRK A **[TRKA]** is expressed and or p140TRK A is phosphorylated in 10 of 10 cell lines.
    However, no other downstream responses to NGF stimulation such as tyrosine phosphorylation of **PLC** gamma 1, PI 3 kinase, ERK1 and ERK2, induction of FOS and NGFI A mRNAs, and neurite extension were observed in the unresponsive cell lines.
    Transfection with p75LNGFR alone had no effect on responses to NGF stimulation.
    Three cell lines stably transfected with TRK A **[TRKA]** exhibited early responses to NGF stimulation, but neurite extension was not observed.
    Our results indicate that endogenous TRK A **[TRKA]** in non responsive cell lines is either defective, or present in amounts below a threshold level required to elicit measurable responses to NGF.
    Furthermore, even after transfection with exogenous TRK A **[TRKA]**, early responses were restored but later events such as neurite outgrowth did not occur, suggesting that downstream responsiveness is blocked as well.
