# Supplementary material for: Content-rich biological network constructed by mining PubMed abstracts
Source: BMC Bioinformatics. 2004 Oct 8;5:147. doi: 10.1186/1471-2105-5-147 (PMC528731; doi:10.1186/1471-2105-5-147)
Supplement: Additional File 5 — The original Chilibot query results of the term "long-term potentiation (LTP)" and 22 other terms, limiting the latest references analyzed to the years 1990, 1995, 2000, and 2004. [file 1471-2105-5-147-S5.bz2 › chilibotAdditionalFile5/ltp1995/html/PLC_TRKB.html]

 


 **PLC** and **TRKB** 
  
Found 2 abstracts in PubMed,  **2 abstracts were retrieved and analyzed**.  


---

 Search Google  |
 PDF files only 
|  EDU domain only 

---

**Interactive relationship** (e.g. stimulation, inhibition, etc)

- These results suggest that two different pathways, the c ras and the  **PLC**  gamma pathway, are activated by  **TrkB**  receptors in primary neurons.  Ref: 7595513 J Neurochem, 1995
- BDNF and NT 4 5 also induced an autophosphorylation of  **TrkB**  receptors and subsequently resulted in a phosphorylation and binding of phospholipase C gamma  **PLC**  gamma and SH2 containing sequence to the autophosphorylated  **TrkB**  receptors.  Ref: 7595513 J Neurochem, 1995

- :-)
