# Supplementary material for: Content-rich biological network constructed by mining PubMed abstracts
Source: BMC Bioinformatics. 2004 Oct 8;5:147. doi: 10.1186/1471-2105-5-147 (PMC528731; doi:10.1186/1471-2105-5-147)
Supplement: Additional File 5 — The original Chilibot query results of the term "long-term potentiation (LTP)" and 22 other terms, limiting the latest references analyzed to the years 1990, 1995, 2000, and 2004. [file 1471-2105-5-147-S5.bz2 › chilibotAdditionalFile5/ltp1995/html/PLC_ERK.html]

 


 **PLC** and **ERK** 
  
Found 3 abstracts in PubMed,  **3 abstracts were retrieved and analyzed**.  


---

 Search Google  |
 PDF files only 
|  EDU domain only 

---

**Interactive relationship** (e.g. stimulation, inhibition, etc)

**Parallel relationship** (e.g. studied together, co-existance, homology, etc.)

- SHC,  **PLC**  gamma 1, and  **ERK**  tyrosine phosphorylation.  Ref: 7646892 Neuron, 1995
- Fas APO 1 cross linking resulted also in  **ERK**  2 activation and in phospholipase A2 PLA2 induction, independently of the PC  **PLC**  aSMase pathway.  Ref: 8846779 EMBO J, 1995
- Fas APO 1 cross linking in these clones fails to activate PC  **PLC**  and aSMase, while nSMase,  **ERK**  2 and PLA2 activates are induced.  Ref: 8846779 EMBO J, 1995
