# Supplementary material for: Content-rich biological network constructed by mining PubMed abstracts
Source: BMC Bioinformatics. 2004 Oct 8;5:147. doi: 10.1186/1471-2105-5-147 (PMC528731; doi:10.1186/1471-2105-5-147)
Supplement: Additional File 5 — The original Chilibot query results of the term "long-term potentiation (LTP)" and 22 other terms, limiting the latest references analyzed to the years 1990, 1995, 2000, and 2004. [file 1471-2105-5-147-S5.bz2 › chilibotAdditionalFile5/ltp1995/html/PLC_PKC.html]

 


 **PLC** and **PKC** 
  
Found 169 abstracts in PubMed,  **30 abstracts were retrieved and analyzed**.  


---

 Search Google  |
 PDF files only 
|  EDU domain only 

---

**Interactive relationship** (e.g. stimulation, inhibition, etc)

- By down regulating protein kinase C  **PKC**  we demonstrated that  **PKC**  activates PC  **PLC**  and desensitizes PC PLD at no longer incubation times.  Ref: 7583579 Arterioscler Thromb Vasc Biol, 1995
- PMA and zymosan induced activation of PLD and PMA induced activation of  **PLC**  both seem to be mediated by protein kinase PK C beta whereas zymosan induced activation of  **PLC**  is negatively controlled by  **PKC**  delta.  Ref: 8519598 Cell Signal, 1995
- This response was inhibited by preincubating the cells with an inhibitor of phospholipase C  **PLC** , U73122, suggesting that  **PLC**  mediates the induction of  **PKC**  translocation by PDGF.  Ref: 8786826 Neurochem Res, 1995
- We therefore propose that, in submandibular mucous acinar cells, muscarinic activation of the PtdIns P2  **PLC**  pathway regulates phosphatidylcholine specific PLD through both the  **PKC**  and calcium mobilizing arms of the phosphoinositide response,.  Ref: 9026775 Pflugers Arch, 1995
- Activation of  **PLC**  leads to the formation of second messengers that synergistically activate protein kinase C  **PKC** .  Ref: 7608554 J Immunol, 1995
- It also activates phospholipase C  **PLC**  resulting in an increase in cytosolic calcium and diacylglycerol DAG that are the physiological activators of protein kinase C  **PKC** .  Ref: 7589454 FEBS Lett, 1995
- Stimulation of aldosterone synthesis in bovine adrenal zona glomerulosa ZGB cells by angiotensin II AngII is believed to be mediated by the phospholipase C  **PLC**  pathway that results in the increase of cytosolic free calcium concentration and in the activation of protein kinase C  **PKC** .  Ref: 7632615 J Steroid Biochem Mol Biol, 1995
- We demonstrate here that stable expression of dominant negative mutants of both zeta  **PKC**  and Raf 1 lead to reversion of PC  **PLC**  transformed cells.  Ref: 7673165 J Biol Chem, 1995
- Control of AA release by PKA, is mediated both by mechanisms which involve blunting of  **PLC**  activity and mechanisms which are downstream from the  **PLC**   **PKC**  cascade.  Ref: 7548185 Biochim Biophys Acta, 1995
- ... 3H BK binding was unaffected, indicating that the site of action of  **PKC**  alpha and  **PKC**  delta in the BK receptor G protein  **PLC**  pathway is after the receptor and before  **PLC** , i.e., the G protein.  Ref: 7623773 Mol Pharmacol, 1995
- These results clearly implicate both Raf 1 and zeta  **PKC**  as necessary downstream components for transduction of the mitogenic oncogenic signal generated by  **PLC**  mediated hydrolysis of phosphatidylcholine and suggest, together with other recent evidence, a bifurcation in the signaling pathway downstream of PC  **PLC** .  Ref: 7673165 J Biol Chem, 1995
- Its effect on AA release however was not blocked by inhibiting protein kinase C  **PKC**  with staurosporine SSP and consequently did not notably involve the  **PLC**   **PKC**  cascade.  Ref: 7548185 Biochim Biophys Acta, 1995

**Parallel relationship** (e.g. studied together, co-existance, homology, etc.)

- We investigated phospholipase D PLD activation as an alternate source of diacylglycerol in astrocytes, since the ratio of  **PLC**  to  **PKC**  activation by carbachol was lower in astrocytes than observed in neurons.  Ref: 7554228 Brain Res Dev Brain Res, 1995
- Several components of the IP signalling system including G proteins, phosphatidylinositol specific phospholipase C PI  **PLC** , protein kinase C  **PKC**  and calcium homeostasis are susceptible to inhibition disruption by aluminum compounds.  Ref: 7557263 Gen Pharmacol, 1995
- The contributions of phosphoinositide PI and phosphatidylcholine PC specific phospholipases PI specific phospholipase C PI  **PLC** , PC specific phospholipase C PC  **PLC** , and phospholipase D PLD to diacylglycerol DAG formation and regulation of the enzymes by G proteins, calcium, and protein kinase C  **PKC**  were examined in dispersed intestinal circular and longitudinal muscle cells.  Ref: 7651363 Mol Pharmacol, 1995
- **PKC**  was activated translocation of activity from cytosol to membrane following stimulation with carbachol, so we tested for activation of phospholipase C  **PLC**  as the source of diacylglycerol released from polyphosphoinositide PIP2 hydrolysis.  Ref: 7554228 Brain Res Dev Brain Res, 1995
- The lower toxicities of these compounds cannot be explained by a less pronounced inhibition of  **PKC**  or  **PLC** , respectively.  Ref: 7639930 Anticancer Drug Des, 1995
- Optimal concentrations of alpha thrombin and TRAP activated  **PLC**  maximally, but TRAP induced only about 50% protein kinase C  **PKC**  activation after 10 min stimulation compared with alpha thrombin.  Ref: 7542872 Biochem J, 1995
- The results suggest a crosstalk between a G protein linked receptor and a receptor tyrosine kinase, involving signalling via  **PLC**  beta and  **PKC**  to a downstream protein tyrosine phosphatase functioning in the control of EGF receptor activity.  Ref: 7556663 FEBS Lett, 1995
- We have used such PC  **PLC**  transformed cells to evaluate the roles of the cytoplasmic serine threonine kinases Raf 1, zeta protein kinase C zeta  **PKC**  and protein kinase A PKA in oncogenesis and mitogenic signal transduction elicited by phosphatidylcholine hydrolysis.  Ref: 7673165 J Biol Chem, 1995
- These results suggest that PGF2 alpha induced PLD activation is different from  **PLC**   **PKC**  systems.  Ref: 8848544 Prostaglandins, 1995
- The present study was designed to characterize the relationship between opioids and activation of phospholipase C  **PLC**  and protein kinase C  **PKC**  in brain injury induced pial vasoconstriction.  Ref: 7583321 Brain Res, 1995
- iv the new HePC congeners D21266, D21133 and D21805 affect the same targets as HePC, i.e.  **PKC**  and phosphatidylinositol bisphosphate specific phospholipase C  **PLC** .  Ref: 7639930 Anticancer Drug Des, 1995
- Further, D609, an inhibitor of phosphatidylcholine phospholipase C PC  **PLC** , and secondarily  **PKC**  also blocks TGF beta 1 induced transcription of the transgene in A549 cells while the phosphatidylinositol  **PLC**  pathway inhibitor U73122 is without effect.  Ref: 7775410 J Biol Chem, 1995
- A role for both the  **PKC**  associated and calcium mobilizing arms of the PtdIns P2  **PLC**  pathway in PLD regulation is thus suggested.  Ref: 9026775 Pflugers Arch, 1995
- The ET 1 evoked increases in DNA synthesis, IP3, calcium i, membrane  **PKC** , and 85 kD MARCKS protein phosphorylation in rat cortical astrocytes were prevented by either the selective endothelin ETA receptor antagonist, BQ 123, or the phospholipase C  **PLC**  specific inhibitor, U 73122.  Ref: 8567063 Glia, 1995
- U 73122, a phospholipase C  **PLC**  inhibitor, and staurosporine, a protein kinase C  **PKC**  inhibitor, did not inhibit PGF2 alpha stimulated 3H PtdEt accumulation.  Ref: 8848544 Prostaglandins, 1995
- In lymphocytes, receptor  **PLC**  linked  **PKC**  activation modulates calcium i not only by inhibiting calcium influx but also by stimulating plasma membrane calcium i extrusion.  Ref: 8746951 Cell Calcium, 1995
- Activation of  **PLC**  beta and protein kinase C  **PKC**  via G protein linked ATP receptors greatly diminished the basic EGF receptor activity in A 431 cells.  Ref: 7556663 FEBS Lett, 1995
- Together, these results suggest that PC  **PLC**  and  **PKC**  are in a TGF beta signaling pathway that results in elevated gene expression.  Ref: 7775410 J Biol Chem, 1995
- Collectively, these data suggest that LTB4 activates the NADPH oxidase in eosinophils by PLD and PtdIns 3 kinase independent mechanisms that involve calcium,  **PLC**  and  **PKC** .  Ref: 7575412 Biochem J, 1995
- Paradoxically, Ro 31 8220 doubled the amount of H2O2 produced by LTB4 which may relate to the ability of  **PKC**  to inhibit cell signalling through phospholipase C  **PLC** .  Ref: 7575412 Biochem J, 1995
- It could be that activation of certain  **PKC**  isozymes, probably  **PKC**  alpha and epsilon, by these activators causes feedback inhibition of  **PLC**  and, consequently, the killing in monocytes, because PMA blocks the Fc gamma R mediated intracellular inositol, 5 P3 formation and  **PKC**  translocation.  Ref: 7608554 J Immunol, 1995
