# Supplementary material for: Content-rich biological network constructed by mining PubMed abstracts
Source: BMC Bioinformatics. 2004 Oct 8;5:147. doi: 10.1186/1471-2105-5-147 (PMC528731; doi:10.1186/1471-2105-5-147)
Supplement: Additional File 5 — The original Chilibot query results of the term "long-term potentiation (LTP)" and 22 other terms, limiting the latest references analyzed to the years 1990, 1995, 2000, and 2004. [file 1471-2105-5-147-S5.bz2 › chilibotAdditionalFile5/ltp1995/html/PLC_PKA.html]

 


 **PLC** and **PKA** 
  
Found 18 abstracts in PubMed,  **18 abstracts were retrieved and analyzed**.  


---

 Search Google  |
 PDF files only 
|  EDU domain only 

---

**Interactive relationship** (e.g. stimulation, inhibition, etc)

- Activation of  **PKA**  in response to elevation of cAMP levels also lead to reversion of PC  **PLC**  induced transformation, implicating  **PKA**  as a negative regulator acting downstream of PC  **PLC** .  Ref: 7673165 J Biol Chem, 1995
- These results suggest that CD3 stimulation activates PIP2 hydrolysis by inducing tyrosine phosphorylation of  **PLC**  gamma 1, which is regulated negatively by PKC and  **PKA** .  Ref: 1832154 J Biol Chem, 1991
- Incubation of the purified enzymes with the catalytic subunit of protein kinase A  **PKA**  and gamma 32P ATP resulted in increased phosphorylation of  **PLC**  I and  **PLC**  II, but it had no inhibitory effect on their enzyme activities.  Ref: 8380992 Biochem J, 1993
- Control of AA release by  **PKA** , is mediated both by mechanisms which involve blunting of  **PLC**  activity and mechanisms which are downstream from the  **PLC**  PKC cascade.  Ref: 7548185 Biochim Biophys Acta, 1995
- Thus, phosphorylation of  **PLC**  gamma 1 by PKC or  **PKA**  at serine 1248 may modulate the interaction of  **PLC**  gamma 1 with the protein tyrosine kinase or the protein tyrosine phosphatase.  Ref: 1370476 J Biol Chem, 1992
- Phosphopeptide map analysis indicated that the sites of  **PLC**  gamma 1 phosphorylated in Jurkat cells treated with PMA and forskolin are the same as those phosphorylated in vitro by protein kinase C PKC and cAMP dependent protein kinase  **PKA** , respectively.  Ref: 1370476 J Biol Chem, 1992
- Phosphorylation of one or more membrane associated proteins by  **PKA**  may regulate myometrial  **PLC**  activity and play a role in the inhibitory effects of isoproterenol and relaxin.  Ref: 1324160 Endocrinology, 1992

**Parallel relationship** (e.g. studied together, co-existance, homology, etc.)

- TSH stimulates thyroid cells via both the adenylate cyclase  **PKA**  and the  **PLC**  PKC Ca signal transduction pathways.  Ref: 7747250 Surg Clin North Am, 1995
- These results imply that H ras functions, in this system, to decrease levels of cAMP, thus negating the regulatory effect of  **PKA**  on  **PLC** .  Ref: 7802640 Biochem Biophys Res Commun, 1994
- The relative roles of the adenylate cyclase protein kinase A system AC  **PKA** , the phospholipase C protein kinase C system  **PLC**  PKC, and increases in cytosolic calcium in mediating the final actions of parathyroid hormone PTH remain ill defined.  Ref: 7511344 Am J Physiol, 1994
- We have used such PC  **PLC**  transformed cells to evaluate the roles of the cytoplasmic serine threonine kinases Raf 1, zeta protein kinase C zeta PKC and protein kinase A  **PKA**  in oncogenesis and mitogenic signal transduction elicited by phosphatidylcholine hydrolysis.  Ref: 7673165 J Biol Chem, 1995
- Bradykinin BDK was used to stimulate  **PLC**  and AA release, while arginine vasopressin AVP, forskolin FSK, isobutylmethylxanthine IBMX were used to increase cAMP levels and stimulate  **PKA** .  Ref: 7548185 Biochim Biophys Acta, 1995
- In parallel experiments, H 89, a specific inhibitor of  **PKA** , was preincubated for 60 min prior to addition of BDK and this resulted in a reversal of FSK IBMX induced inhibition of basal and BDK stimulated  **PLC**  activity and AA release.  Ref: 7548185 Biochim Biophys Acta, 1995
- Regulation of phospholipases C  **PLC**  and arachidonic acid AA release by cAMP dependent protein kinase  **PKA**  was investigated in MDCK D1 cells.  Ref: 7548185 Biochim Biophys Acta, 1995
- The results strongly suggest a role for  **PKA**  in the regulation of  **PLC**  activity and AA release in MDCK D1 cells.  Ref: 7548185 Biochim Biophys Acta, 1995
- These results suggest that  **PKA**  mediated phosphorylation of  **PLC**  may regulate TCR CD3 induced InsPL hydrolysis.  Ref: 1318020 Biochem J, 1992
- We postulate that these effects are due to the regulatory action of  **PKA**  on  **PLC** .  Ref: 7802640 Biochem Biophys Res Commun, 1994
- Although an important role for the  **PLC**  PKC system in the regulation of phosphate transport in response to PTH has been suggested, previous studies from our laboratory and others, in OK cells, have emphasized the major role of AC  **PKA** .  Ref: 7511344 Am J Physiol, 1994
