# Supplementary material for: Content-rich biological network constructed by mining PubMed abstracts
Source: BMC Bioinformatics. 2004 Oct 8;5:147. doi: 10.1186/1471-2105-5-147 (PMC528731; doi:10.1186/1471-2105-5-147)
Supplement: Additional File 5 — The original Chilibot query results of the term "long-term potentiation (LTP)" and 22 other terms, limiting the latest references analyzed to the years 1990, 1995, 2000, and 2004. [file 1471-2105-5-147-S5.bz2 › chilibotAdditionalFile5/ltp1995/html/PLC_NMDA.html]

 


 **PLC** and **NMDA** 
  
Found 4 abstracts in PubMed,  **4 abstracts were retrieved and analyzed**.  


---

 Search Google  |
 PDF files only 
|  EDU domain only 

---

**Interactive relationship** (e.g. stimulation, inhibition, etc)

- Both glutamate induced neuronal degeneration and the increases in  **PLC**  delta immunoreactivity were prevented by removal of extracellular calcium or the application of an N methyl D aspartate  **NMDA**  receptor antagonist, MK 801.  Ref: 7563235 J Neurosci Res, 1995
- Both kinetic analysis and equilibrium saturation experiments indicated that  **PLC**  treatment produced a decrease in affinity for 3H N 1 thienyl cyclohexyl piperidine 3H TCP, a ligand for the N methyl D aspartate  **NMDA**  receptor associated ionic channel, when the channel was fully activated by high concentrations of glutamate and glycine but increased its binding under conditions in which the channel was presumably closed.  Ref: 2154675 Mol Pharmacol, 1990
- It was revealed that nitric oxide NO formation secondary to calcium influx by N methyl D aspartate  **NMDA**  receptor activation leads to modifications of  **PLC**  delta 1 similar to those seen in AD brains.  Ref: 8534418 Alzheimer Dis Assoc Disord, 1995
- These results suggest that NO formation secondary to calcium influx by  **NMDA**  receptor activation leads to similar modifications of  **PLC**  delta to those seen in AD.  Ref: 7563235 J Neurosci Res, 1995
- In contrast to  **PLC**  from C. perfringens, phosphatidylinositol specific  **PLC**  treatment did not detectably modify the binding properties of the quisqualate AMPA receptor or the  **NMDA**  receptor channel.  Ref: 2154675 Mol Pharmacol, 1990

**Parallel relationship** (e.g. studied together, co-existance, homology, etc.)

- Binding of 3H glutamate and 3H glycine to the  **NMDA**  receptors was not modified by  **PLC**  treatment, but there was a large decrease in the binding of the  **NMDA**  antagonist 3H 3 2 carboxypiperazine 4 yl propyl 1 phosphonic acid.  Ref: 2154675 Mol Pharmacol, 1990
