# Supplementary material for: Content-rich biological network constructed by mining PubMed abstracts
Source: BMC Bioinformatics. 2004 Oct 8;5:147. doi: 10.1186/1471-2105-5-147 (PMC528731; doi:10.1186/1471-2105-5-147)
Supplement: Additional File 5 — The original Chilibot query results of the term "long-term potentiation (LTP)" and 22 other terms, limiting the latest references analyzed to the years 1990, 1995, 2000, and 2004. [file 1471-2105-5-147-S5.bz2 › chilibotAdditionalFile5/ltp1995/html/PLC_TAU.html]

 


 **PLC** and **TAU** 
  
Found 7 abstracts in PubMed,  **7 abstracts were retrieved and analyzed**.  


---

 Search Google  |
 PDF files only 
|  EDU domain only 

---

**Interactive relationship** (e.g. stimulation, inhibition, etc)

- However, NDPK inhibited GTP  **tau**  S stimulated  **PLC** .  Ref: 7780113 Chin Med Sci J, 1995
- In contrast, activation of G proteins with GTP  **tau**  S or AlF4 stimulates both prostaglandin synthesis and  **PLC** .  Ref: 2122640 Adv Exp Med Biol, 1990
- Anti  **PLC**  delta 1 antibody marked the same NFT bearing neurons containing  **tau**  immunoreactivity.  Ref: 8534418 Alzheimer Dis Assoc Disord, 1995

**Parallel relationship** (e.g. studied together, co-existance, homology, etc.)

- Anti  **PLC**  delta antibody marked the same NFT bearing neurons containing  **tau**  immunoreactivity with  **tau**  more clearly on NFT filaments and  **PLC**  delta covering it superficially at the light microscope level.  Ref: 7712177 Brain Res, 1995
- These findings suggest that  **PLC**   **tau** , and perhaps the 76 kDa co precipitated protein, are substrates of cyclic AMP dependent protein kinase in BALB c 3T3 cells however, the lack of effect of cyclic AMP elevation on PDGF stimulated inositol phosphate formation indicates that the intrinsic activity of  **PLC**   **tau**  is unaltered by cyclic AMP mediated phosphorylation.  Ref: 1702622 Biochem J, 1990
- Previous studies have demonstrated enhanced phosphorylation of phospholipase C  **tau**   **PLC**   **tau** , a key regulatory enzyme in phosphoinositide metabolism, in cells treated with platelet derived growth factor PDGF and epidermal growth factor, both of which act via specific receptor tyrosine kinases.  Ref: 1702622 Biochem J, 1990
- The effect of nucleoside diphosphate kinase NDPK on the activity of guanine nucleotide regulatory protein G protein mediated phospholipase C  **PLC**  and on the 35S GTPT  **tau**  S binding of G protein was investigated in this work in order to demonstrate the mechanism behind the regulation of G protein and its effector  **PLC**  by NDPK.  Ref: 7780113 Chin Med Sci J, 1995
- Treatment of cells with cyclic AMP agonists also enhanced, with similar kinetics, the phosphorylation of a 76 kDa protein co precipitated by anti  **PLC**   **tau**  monoclonal antibodies.  Ref: 1702622 Biochem J, 1990
- In addition, using permeabilized lymphoma cells, we have found that 1 GTP or GTP  **tau**  S augments, and pertussis toxin inhibits, phospholipase C  **PLC**  activity and receptor capping.  Ref: 1968926 J Immunol, 1990
- **PLC**  delta marked the same neurons containing  **tau**  immunoreactivity and yet  **tau**  and  **PLC**  delta often marked different structures within the same neuron, with  **tau**  more clearly on NFT and  **PLC**  delta covering it superficially.  Ref: 8239311 Ann N Y Acad Sci, 1993
- The stimulation of  **PLC**  in turkey erythrocyte membrane by both GTP and GTP  **tau**  S indicated that the  **PLC**  stimulation was mediated by G protein.  Ref: 7780113 Chin Med Sci J, 1995
