# Supplementary material for: Content-rich biological network constructed by mining PubMed abstracts
Source: BMC Bioinformatics. 2004 Oct 8;5:147. doi: 10.1186/1471-2105-5-147 (PMC528731; doi:10.1186/1471-2105-5-147)
Supplement: Additional File 5 — The original Chilibot query results of the term "long-term potentiation (LTP)" and 22 other terms, limiting the latest references analyzed to the years 1990, 1995, 2000, and 2004. [file 1471-2105-5-147-S5.bz2 › chilibotAdditionalFile5/ltp1995/html/PLC_ACTIN.html]

 


 **PLC** and **ACTIN** 
  
Found 29 abstracts in PubMed,  **29 abstracts were retrieved and analyzed**.  


---

 Search Google  |
 PDF files only 
|  EDU domain only 

---

**Interactive relationship** (e.g. stimulation, inhibition, etc)

- This process may involve binding of  **PLC**  gamma 1 to  **actin**  filaments, since  **actin**  was immunoprecipitated together with  **PLC**  gamma 1 in the cytoskeleton after EGF treatment.  Ref: 8125925 J Biol Chem, 1994
- Using immunofluorescence microscopy, we show that a truncated protein composed of the SH2 and SH3 domains of  **PLC**  gamma was localized to the  **actin**  cytoskeleton.  Ref: 8334708 Cell, 1993
- Dissociation of erythrocyte ghost cytoskeletons with the  **actin**  binding protein DNase 1 resulted in a dose dependent inhibition of agonist and guanine nucleotide stimulated  **PLC**  responses in ghosts and caused release of  **PLC**  from ghost or cytoskeleton preparations.  Ref: 1429646 J Biol Chem, 1992
- Large amounts of gelsolin  **actin**  1 1 complex gelsolin complex were detected in the  **PLC**  delta and  **PLC**  gamma 1 fractions.  Ref: 1313007 J Biol Chem, 1992
- These data demonstrate the specific association of a receptor and G protein regulated  **PLC**  with a component of the detergent insoluble cytoskeleton and indicate that the integrity of the  **actin**  cytoskeleton is important for localization and effective coupling of  **PLC**  to the relevant G protein.  Ref: 1429646 J Biol Chem, 1992
- In REF52 cells,  **PLC**  gamma was associated with the  **actin**  cytoskeleton and was evenly distributed along the length of the  **actin**  microfilaments.  Ref: 1651494 Proc Natl Acad Sci U S A, 1991
- These findings suggest that this novel RhoGAP is involved in the Rho signalling pathway, probably downstream of Rho activation, and mediates the stimulation of  **PLC**  delta, which leads to  **actin**  related cytoskeletal changes through the hydrolysis of PIP2, which binds to  **actin**  binding proteins such as gelsolin and profilin.  Ref: 7835339 EMBO J, 1995

**Parallel relationship** (e.g. studied together, co-existance, homology, etc.)

- When the steady state levels of the predominant 6.7 kb species were normalized to  **actin**  mRNA,  **PLC**  were 6.3 fold lower than ILC and 1.7 fold lower than ALC n = 3 replicate isolations of poly A RNA.  Ref: 1505454 Endocrinology, 1992
- Furthermore, analysis by reverse transcriptase polymerase chain reaction revealed that the ratio of the expression of  **PLC**  delta to that of beta  **actin**  in the neoplasms was significantly lower than the ratios in the non neoplastic colon mucosae of carcinogen treated and control rats P <.  Ref: 7528022 Mol Carcinog, 1994
- Cytoskeletal association of  **PLC**  gamma was not transformation sensitive, although the  **actin**  cytoskeleton was more disordered in simian virus 40 transformed cells.  Ref: 1651494 Proc Natl Acad Sci U S A, 1991
- A peptide corresponding to a basic consensus amino acid motif present in both  **actin**  binding proteins and phosphoinositide specific phospholipases C was synthesized and its effect on the activity of a recombinant phospholipase C beta 2  **PLC**  beta 2 expressed in baculovirus infected insect cells was studied.  Ref: 8397113 FEBS Lett, 1993
- Teleocidin, a phorbol ester type tumor promoter, enhanced  **actin**  redistribution, vacuole formation and c fos expression of  **PLC**  PRF 5 hepatoma cells.  Ref: 8485167 Biochim Biophys Acta, 1993
