# Supplementary material for: Content-rich biological network constructed by mining PubMed abstracts
Source: BMC Bioinformatics. 2004 Oct 8;5:147. doi: 10.1186/1471-2105-5-147 (PMC528731; doi:10.1186/1471-2105-5-147)
Supplement: Additional File 5 — The original Chilibot query results of the term "long-term potentiation (LTP)" and 22 other terms, limiting the latest references analyzed to the years 1990, 1995, 2000, and 2004. [file 1471-2105-5-147-S5.bz2 › chilibotAdditionalFile5/ltp1995/html/PLC_SYNAPTOPHYSIN.html]

 


 **PLC** and **SYNAPTOPHYSIN** 
  
Found 1 abstracts in PubMed,  **1 abstracts were retrieved and analyzed**.  


---

 Search Google  |
 PDF files only 
|  EDU domain only 

---

**Interactive relationship** (e.g. stimulation, inhibition, etc)

- When certain epithelial non NE cells, such as human hepatocellular carcinoma  **PLC**  cells, were cDNA transfected to synthesize  **synaptophysin** , the new molecules appeared in specific SET vesicles.  Ref: 7798314 J Cell Biol, 1994

**Parallel relationship** (e.g. studied together, co-existance, homology, etc.)

- As this was in contrast to other reports that only NE cells were able to sort  **synaptophysin**  away from other plasma membrane proteins into presynaptic or SLMV type vesicles, we have further characterized the vesicles containing  **synaptophysin**  in transfected  **PLC**  cells.  Ref: 7798314 J Cell Biol, 1994
