# Supplementary material for: Content-rich biological network constructed by mining PubMed abstracts
Source: BMC Bioinformatics. 2004 Oct 8;5:147. doi: 10.1186/1471-2105-5-147 (PMC528731; doi:10.1186/1471-2105-5-147)
Supplement: Additional File 5 — The original Chilibot query results of the term "long-term potentiation (LTP)" and 22 other terms, limiting the latest references analyzed to the years 1990, 1995, 2000, and 2004. [file 1471-2105-5-147-S5.bz2 › chilibotAdditionalFile5/ltp1995/html/TRKA_TRKB.html]

 


 **TRKA** and **TRKB** 
  
Found 84 abstracts in PubMed,  **30 abstracts were retrieved and analyzed**.  


---

 Search Google  |
 PDF files only 
|  EDU domain only 

---

**Interactive relationship** (e.g. stimulation, inhibition, etc)

- Trigeminal neurons from trkC  **trkA**  embryos did not respond to NT3 and nodose neurons from  **trkB**  embryos likewise failed to respond to NT3.  Ref: 7556091 EMBO J, 1995
- Immunohistochemistry with  **TrkB** ,  **TrkA** , and p75 antisera revealed that VGNs made  **TrkB**  and p75 proteins, but not  **TrkA**  protein.  Ref: 8568514 J Neurobiol, 1995
- **trkA**  interacts preferably with nerve growth factor NGF,  **trkB**  with brain derived neurotrophic factor BDNF and neurotrophin 4 5 NT 4 5 and trkC with neurotrophin 3 NT 3.  Ref: 7796806 EMBO J, 1995
- It is likely that binding of BDNF to  **TrkB** , which appears to be more abundant and ubiquitous than  **TrkA** , restricts the diffusion of BDNF relative to that of NGF.  Ref: 7665731 J Comp Neurol, 1995
- Although generated to  **trkA**  fusion proteins, this panel of MAbs also recognized  **trkB**  and trkC in flow cytometric studies of NIH3T3 cells transfected with  **trkB**  or trkC cDNAs.  Ref: 7642800 J Comp Neurol, 1995
- In situ hybridization studies confirmed that the high affinity BDNF receptor  **TrkB**  was much more widely expressed in neurons than was the high affinity NGF receptor  **TrkA** .  Ref: 7665731 J Comp Neurol, 1995
- **TrkA**  and TrkC expression was identified in sympathetic ganglia and within the adrenal medulla, with intense  **TrkB**  expression restricted to paraganglia, of the normal developing human sympathetic nervous system.  Ref: 7604872 Am J Pathol, 1995
- In contrast to the drop in NGF and  **trkA**  mRNA expression, NT 4 mRNA levels increased at the time of follicular assembly, coinciding with the abrupt appearance of  **trkB**  mRNA.  Ref: 7664689 Endocrinology, 1995

**Parallel relationship** (e.g. studied together, co-existance, homology, etc.)

- Developmental changes in NT3 signalling via  **TrkA**  and  **TrkB**  in embryonic neurons.  Ref: 7556091 EMBO J, 1995
- Neurotrophins promote neuronal survival by signalling through Trk receptor tyrosine kinases nerve growth factor signals through  **TrkA** , brain derived neurotrophic factor BDNF and neurotrophin NT 4 through  **TrkB**  and NT3 through TrkC.  Ref: 7556091 EMBO J, 1995
- At P5, the innermost part of the inner nuclear layer INL also expressed  **TrkA** ,  **TrkB**  and p75 mRNAs.  Ref: 8750972 Brain Res, 1995
- To investigate the possibility that the neurotrophin tyrosine kinase receptors are also recognition molecules by virtue of their immunoglobulin like domains, the ability of  **TrkA**  and  **TrkB**  to influence neurite outgrowth was tested in vitro.  Ref: 7582119 Eur J Neurosci, 1995
- Identification of a structural motif capable of specifically recognizing the various neurotrophins was achieved by assessing their affinities to isolated recombinant modules of  **TrkA**  and  **TrkB** .  Ref: 7499302 J Biol Chem, 1995
- and high levels of trkC mRNA, and relatively high amount of  **trkB**  mRNA, while levels of  **trkA**  mRNA was undetectable.  Ref: 8580426 Neuroreport, 1995
- However, double homozygous  **trkA**  ;  **trkB**  DRG and  **trkB**  ; trkC vestibular neurons showed the same degree of survival as single  **trkA**  and  **trkB**  mice, respectively, suggesting that those neurons required both Trk signaling pathways for survival.  Ref: 8575307 Development, 1995
- However, RA induced  **TrkB**  mRNA expression is transcriptionally regulated, while the transient RA induced increase of  **TrkA**  mRNA is a consequence of extended mRNA stability.  Ref: 7559588 J Biol Chem, 1995
- Thus, astroglial cells in culture internalize NGF through a specific receptor mediated process, express  **trkA**  and full length  **trkB**  mRNAs at low levels, and respond to exogenous NGF by expressing a fibrous morphology under serum free culture conditions.  Ref: 7650754 J Neurosci Res, 1995
- NT 3 is a somewhat promiscuous ligand that can also activate  **TrkA**  and  **TrkB**  receptors at high concentrations.  Ref: 7486690 Ann N Y Acad Sci, 1995
- Specific neurotrophin binding to leucine rich motif peptides of  **TrkA**  and  **TrkB** .  Ref: 7589499 FEBS Lett, 1995
- The presence of the neurotrophin, nerve growth factor, brain derived neurotrophic factor, neurotrophin 3 and neurotrophin 4 NGF, BDNF, NT 3 and NT 4 and their receptors of the tyrosine kinase family  **trkA** ,  **trkB**  and trkC have been investigated in the choroid plexus and dura mater of the adult rat by ribonuclease protection assay.  Ref: 8580426 Neuroreport, 1995
- We examined the cellular distribution of mRNAs coding for the neurotrophin receptors  **TrkA** ,  **TrkB**  and p75 in the rat retina during early postnatal development.  Ref: 8750972 Brain Res, 1995
- The extracellular domains of the  **TrkA**  and  **TrkB**  neurotrophin receptors contain defined structural modules such as immunoglobulin like domains and leucine rich motifs LRMs Schneider and Schweiger, Oncogene 6 1991 1807 1811.  Ref: 7589499 FEBS Lett, 1995
- RT PCR analysis of these spheres of undifferentiated cells revealed the expression of mRNA for the  **trkB**  neurotrophin receptor, both with and without the catalytic domain, and little or no expression of  **trkA**  or trkC.  Ref: 7643217 J Neurosci, 1995
- These results show that NT3 can signal through  **TrkA**  and  **TrkB**  in neurons at certain stages of development and may explain why the phenotype of NT3 mice is more severe than that of trkC mice.  Ref: 7556091 EMBO J, 1995
- and high levels of  **trkB**  mRNA, and undetectable levels of  **trkA**  and trkC mRNA.  Ref: 8580426 Neuroreport, 1995
- Selective regulation of  **TrkA**  and  **TrkB**  receptors by retinoic acid and interferon gamma in human neuroblastoma cell lines.  Ref: 7559588 J Biol Chem, 1995
- The influence of age on immunohistochemically demonstrable neurotrophin receptor proteins p75,  **trkA** ,  **trkB** , and trkC proteins was studied in the cerebellar cortex of Wistar male rats aged 3 young, 12 adult and 24 old months.  Ref: 8523897 Mech Ageing Dev, 1995
- The Trk family of tyrosine protein kinases,  **TrkA** ,  **TrkB** , and TrkC, are the signaling receptors that mediate the biological properties of the NGF family of neurotrophins.  Ref: 7486690 Ann N Y Acad Sci, 1995
- Immunocytochemistry was utilized to evaluate  **TrkA** ,  **TrkB** , and TrkC protein expression at the cellular level in the developing human fetal sympathetic nervous system and in a selection of neuroblastoma tumor specimens.  Ref: 7604872 Am J Pathol, 1995
- Human neuroblastoma NB cell lines constitutively express low levels of  **TrkA**  mRNA, while  **TrkB**  mRNA is not readily detectable.  Ref: 7559588 J Biol Chem, 1995
- The neurotrophin receptors  **TrkA**  and  **TrkB**  are inhibitory for neurite outgrowth.  Ref: 7582119 Eur J Neurosci, 1995
- Taken together, the results indicate the existence in postnatal rat brain of a large overlapping population of cholinergic neurons that are responsive to ligands for the neurotrophin receptors  **TrkA**  and  **TrkB** ... and small distinct populations that show specificity for NGF or BDNF but not both.  Ref: 7552337 Brain Res, 1995
- Thus, RA and IFN gamma differentially regulate  **TrkA**  or  **TrkB**  expression in the same cell type by predominantly transcriptional  **TrkB**  or post transcriptional  **TrkA**  mechanisms.  Ref: 7559588 J Biol Chem, 1995
- Induction of both  **TrkA**  and  **TrkB**  mRNA does not require new protein synthesis.  Ref: 7559588 J Biol Chem, 1995
- Cell monolayers of fibroblasts transfected to express either the  **TrkA**  or  **TrkB**  receptor reduced neurite outgrowth of phaeochromocytoma PC12 cells by 50 60% when compared to mock transfected fibroblasts or fibroblasts transfected with the epidermal growth factor receptor.  Ref: 7582119 Eur J Neurosci, 1995
- On the basis of the recent identification of a nerve growth factor NGF binding site within  **TrkA** , the ability of the different structural entities within the extracellular domain of  **TrkB**  to bind the various neurotrophins was determined by using a recombinant receptor approach.  Ref: 7669784 Biochemistry, 1995
- The ovary also expresses some of the neurotrophin receptors, including p75 NGFR,  **trkB**  the receptor for NT 4 5 and brain derived neurotropic factor BDNF, and  **trkA**  the NGF receptor.  Ref: 7664689 Endocrinology, 1995
- In the rat aortic balloon deendothelialization model of vascular injury, the expression of NGF, BNDF, and their receptors trk A  [ **TRKA** ]  and trk B  [ **TRKB** ]  increased dramatically in the area of injury within 3 days and persisted during the formation of the neointima.  Ref: 7639328 Am J Pathol, 1995
- Immunohistochemistry, with  **TrkB**  and  **TrkA**  antisera, revealed that these neurons produced  **TrkB**  protein, the functional receptor for NT 4 5 and BDNF... the high affinity receptor for NGF.  Ref: 7623136 J Neurosci, 1995
- Chimeras of trkC where this domain was exchanged for the homologous sequences from  **trkB**  or  **trkA**  gained high affinity binding to BDNF or NGF respectively, while deletion of this domain in trkC or  **trkA**  abolished binding to NT 3 or NGF respectively.  Ref: 7796806 EMBO J, 1995
- **TrkA** ,  **TrkB**  and p75 mRNA expression is developmentally regulated in the rat retina.  Ref: 8750972 Brain Res, 1995
- trkC mRNA is expressed first, followed by  **trkB**  mRNA and finally  **trkA**  mRNA.  Ref: 8747136 Neuroreport, 1995
- Although studies in some... cell lines indicate that NT3 can also signal through  **TrkA**  and  **TrkB** , it is not known if such signalling can occur in neurons.  Ref: 7556091 EMBO J, 1995
- Interferon gamma IFN gamma selectively increases  **TrkA**  mRNA without affecting  **TrkB**  mRNA levels.  Ref: 7559588 J Biol Chem, 1995
- Also, chicken  **TrkA** ,  **TrkB**  and TrkC have been cloned, sequenced and studied by in situ hybridization.  Ref: 8645570 Int J Dev Biol, 1995
