# Supplementary material for: Content-rich biological network constructed by mining PubMed abstracts
Source: BMC Bioinformatics. 2004 Oct 8;5:147. doi: 10.1186/1471-2105-5-147 (PMC528731; doi:10.1186/1471-2105-5-147)
Supplement: Additional File 5 — The original Chilibot query results of the term "long-term potentiation (LTP)" and 22 other terms, limiting the latest references analyzed to the years 1990, 1995, 2000, and 2004. [file 1471-2105-5-147-S5.bz2 › chilibotAdditionalFile5/ltp1995/html/TRKA_ERK.html]

 


 **TRKA** and **ERK** 
  
Found 1 abstracts in PubMed,  **1 abstracts were retrieved and analyzed**.  


---

 Search Google  |
 PDF files only 
|  EDU domain only 

---

**Interactive relationship** (e.g. stimulation, inhibition, etc)

- NGF induced phosphorylation of the  **trkA**  receptor, activated a cascade of cellular intermediaries such as phospholipase C gamma 1 and  **ERK**  proteins, and stimulated c fos gene transcription in all  **trkA**  expressing clones.  Ref: 7761093 Oncogene, 1995

- :-)
