# Supplementary material for: Content-rich biological network constructed by mining PubMed abstracts
Source: BMC Bioinformatics. 2004 Oct 8;5:147. doi: 10.1186/1471-2105-5-147 (PMC528731; doi:10.1186/1471-2105-5-147)
Supplement: Additional File 5 — The original Chilibot query results of the term "long-term potentiation (LTP)" and 22 other terms, limiting the latest references analyzed to the years 1990, 1995, 2000, and 2004. [file 1471-2105-5-147-S5.bz2 › chilibotAdditionalFile5/ltp1995/html/TRKB_NMDA.html]

 


 **TRKB** and **NMDA** 
  
Found 3 abstracts in PubMed,  **3 abstracts were retrieved and analyzed**.  


---

 Search Google  |
 PDF files only 
|  EDU domain only 

---

- J Neurosci, 1994   **Regulation of brain derived neurotrophic factor BDNF expression and release from hippocampal neurons is mediated by non **NMDA** type glutamate receptors.**.
  We have examined the influence of glutamate on cortical brain derived neurotrophic factor BDNF expression using in situ hybridization and immunohistochemistry.
  Kainic acid KA produced an upregulation of hippocampal and neocortical BDNF mRNA as well as BDNF protein that was blocked by a non **NMDA** antagonist, dinitroquinoxaline dione DNQX , but was not affected by the **NMDA** antagonist 2 amino 7 phosphonoheptanoic acid AP7 .
  Basal levels of BDNF mRNA were not affected by **NMDA**, DNQX, or AP7 treatment.
  BDNF protein was also increased after kainate exposure with a spatial and temporal course distinct from that seen for the expression of BDNF mRNA.
  A dramatic shift in BDNF immunoreactivity IR was observed from intracellular compartments to the neuropil surrounding CA3 pyramidal cells 2 3 hr after KA exposure.
  This shift in localization of BDNF IR suggests a constitutive release of BDNF at the level of the cell body and dendrites.
  Moreover, we have localized mRNAs for full length and truncated **trkB**, to a co incident population of neurons and glia.
  These data suggest the neurons that produce BDNF also express components necessary for a biological response to the same neurotrophic factor.
  The present study also demonstrates increased BDNF IR in the mossy fiber terminal zone of hippocampus after exposure to KA, as well as an increase in **trkB** mRNA, and provides evidence of local release of this neurotrophin into the surrounding neuropil where it would be available for local utilization.
  The synthesis and putative release of BDNF from somatic and or dendritic sites within the hippocampus provide evidence of a potential autocrine or paracrine role for BDNF, and establish a local source of trophic support for the maintenance of synaptic plasticity and anatomic reorganization in the mature nervous system.

  - Brain Res Mol Brain Res, 1993   **Rapid increase of BDNF mRNA levels in cortical neurons following spreading depression regulation by glutamatergic mechanisms independent of seizure activity.**.
    Levels of mRNA for nerve growth factor NGF , brain derived neurotrophic factor BDNF , neurotrophin 3 NT 3 and the tyrosine kinase receptors **trkB** and trkC have been studied using in situ hybridization in the rat brain after topical application of KCl to the cortical surface which induces spreading depression .
    Repeated episodes of spreading depression during 2 h caused a rapid and marked increase of BDNF mRNA levels in deep and, in particular, superficial cortical layers of the ipsilateral hemisphere to 213 and 417% of control, respectively .
    Maximal levels were reached within 2 h after the cessation of spreading depression and at 24 h BDNF mRNA expression had returned to control values.
    Levels of BDNF mRNA were unaffected in the hippocampus, in areas outside the cerebral cortex and in the contralateral hemisphere.
    Furthermore, no change of the expression of mRNA for NGF, NT 3, trkC or the full length **trkB** receptor was detected at any time point.
    However, at 2 h after spreading depression there was an increased level 150% of control in superficial cortical layers of mRNA hybridizing to an oligonucleotide probe detecting both truncated receptors lacking the tyrosine kinase domain and full length **trkB** receptors.
    Also one single episode of spreading depression gave rise to a significant increase of cortical BDNF mRNA levels to 207% of control , which was attenuated by 61% after administration of the competitive **NMDA** receptor antagonist CGS 19755.
    The results provide evidence that mild brain insults associated with glutamate release and elevated intracellular calcium, such as spreading depression, also in the absence of seizure activity can lead to activation of the BDNF gene in cortical neurons.
