# Supplementary material for: Content-rich biological network constructed by mining PubMed abstracts
Source: BMC Bioinformatics. 2004 Oct 8;5:147. doi: 10.1186/1471-2105-5-147 (PMC528731; doi:10.1186/1471-2105-5-147)
Supplement: Additional File 5 — The original Chilibot query results of the term "long-term potentiation (LTP)" and 22 other terms, limiting the latest references analyzed to the years 1990, 1995, 2000, and 2004. [file 1471-2105-5-147-S5.bz2 › chilibotAdditionalFile5/ltp1995/html/ERK_PKC.html]

 


 **ERK** and **PKC** 
  
Found 9 abstracts in PubMed,  **9 abstracts were retrieved and analyzed**.  


---

 Search Google  |
 PDF files only 
|  EDU domain only 

---

**Interactive relationship** (e.g. stimulation, inhibition, etc)

- We conclude that  **PKC**  and EGF act through parallel pathways to stimulate  **ERK**  phosphorylation and activity.  Ref: 7860643 J Cell Physiol, 1995

- :-)

  **Parallel relationship** (e.g. studied together, co-existance, homology, etc.)

  - Thus the  **PKC**  stimulated cyclic AMP response can limit the activation of  **ERK**  2 in response to bradykinin.  Ref: 7998998 Biochem J, 1994
  - Unlike known TPA resistant cells whose resistance is mainly due to lack or down modulation of protein kinase C, UT16 cells showed TPA induced activation of  **PKC** , Raf 1, and  **ERK**  MAP kinases similar to the parental U937 cells.  Ref: 7478524 Oncogene, 1995
  - The role of protein kinase C  **PKC**  in the EGF stimulated  **ERK**  signaling pathway was further examined by inhibition of  **PKC**  with the staurosporine analog, CGP41251, and by down regulation of  **PKC**  via chronic treatment with PMA.  Ref: 7860643 J Cell Physiol, 1995
  - We have previously demonstrated that, in order to induce full activation of  **ERK**  2 with bradykinin, it is necessary to obliterate  **PKC**  stimulated cyclic AMP formation.  Ref: 7702566 Biochem J, 1995
