# Supplementary material for: Content-rich biological network constructed by mining PubMed abstracts
Source: BMC Bioinformatics. 2004 Oct 8;5:147. doi: 10.1186/1471-2105-5-147 (PMC528731; doi:10.1186/1471-2105-5-147)
Supplement: Additional File 5 — The original Chilibot query results of the term "long-term potentiation (LTP)" and 22 other terms, limiting the latest references analyzed to the years 1990, 1995, 2000, and 2004. [file 1471-2105-5-147-S5.bz2 › chilibotAdditionalFile5/ltp1995/html/ERK_PKA.html]

 


 **ERK** and **PKA** 
  
Found 4 abstracts in PubMed,  **4 abstracts were retrieved and analyzed**.  


---

 Search Google  |
 PDF files only 
|  EDU domain only 

---

**Interactive relationship** (e.g. stimulation, inhibition, etc)

**Parallel relationship** (e.g. studied together, co-existance, homology, etc.)

- Injury to hypoglossal motor neurons resulted in an increase in extracellular regulated kinase  **ERK** , or MAP kinase and  **ERK**  kinase MEK, or MAP kinase kinase mRNAs, but in a decrease in the expression of the catalytic subunits of  **PKA**  C alpha and C beta mRNAs.  Ref: 7769990 Brain Res Mol Brain Res, 1995
- A recently described protein kinase, PK40erk, 1 a member of the  **ERK**  family of kinases, can produce in vitro many of the properties of Alzheimer like hyperphosphorylated TAU. cAMP dependent protein kinase A  **PKA**  phosphorylates TAU to a lesser extent.  Ref: 8166686 Biochem Biophys Res Commun, 1994
- With all protein kinases tested, namely  **PKA** , CK1, CK2, MAP kinase  **ERK**  1, c Fgr, Lyn, CSK and TPK IIB p38Syk, staurosporine inhibition was competitive with respect to ATP, regardless of its inhibitory power.  Ref: 8529658 Eur J Biochem, 1995
