# Supplementary material for: Content-rich biological network constructed by mining PubMed abstracts
Source: BMC Bioinformatics. 2004 Oct 8;5:147. doi: 10.1186/1471-2105-5-147 (PMC528731; doi:10.1186/1471-2105-5-147)
Supplement: Additional File 5 — The original Chilibot query results of the term "long-term potentiation (LTP)" and 22 other terms, limiting the latest references analyzed to the years 1990, 1995, 2000, and 2004. [file 1471-2105-5-147-S5.bz2 › chilibotAdditionalFile5/ltp1995/html/ERK_TAU.html]

 


 **ERK** and **TAU** 
  
Found 6 abstracts in PubMed,  **6 abstracts were retrieved and analyzed**.  


---

 Search Google  |
 PDF files only 
|  EDU domain only 

---

**Interactive relationship** (e.g. stimulation, inhibition, etc)

- A recently described protein kinase, PK40erk, 1 a member of the  **ERK**  family of kinases, can produce in vitro many of the properties of Alzheimer like hyperphosphorylated  **TAU** . cAMP dependent protein kinase A PKA phosphorylates  **TAU**  to a lesser extent.  Ref: 8166686 Biochem Biophys Res Commun, 1994

**Parallel relationship** (e.g. studied together, co-existance, homology, etc.)

- The immunoreactivity of cortical and brainstem type Lewy bodies has been investigates with antibodies to the cyclin dependent kinase 5 cdk5, to the extracellular regulated kinase 1  **ERK**  1, and to the cdc2p34 kinase and with antibodies specific for phosphorylation epitopes typical of paired helical filament  **tau**  PHF  **tau** .  Ref: 7485409 Am J Pathol, 1995
- Both cortical and brainstem type Lewy bodies in diffuse Lewy body disease and brainstem type Lewy bodies in Parkinson s disease were found to be immunoreactive for cdk5 but not for cdc2p34 or  **ERK**  1 or with the PHF  **tau**  antibodies.  Ref: 7485409 Am J Pathol, 1995
