# Supplementary material for: Content-rich biological network constructed by mining PubMed abstracts
Source: BMC Bioinformatics. 2004 Oct 8;5:147. doi: 10.1186/1471-2105-5-147 (PMC528731; doi:10.1186/1471-2105-5-147)
Supplement: Additional File 5 — The original Chilibot query results of the term "long-term potentiation (LTP)" and 22 other terms, limiting the latest references analyzed to the years 1990, 1995, 2000, and 2004. [file 1471-2105-5-147-S5.bz2 › chilibotAdditionalFile5/ltp1995/html/ERK_ACTIN.html]

 


 **ERK** and **ACTIN** 
  
Found 6 abstracts in PubMed,  **6 abstracts were retrieved and analyzed**.  


---

 Search Google  |
 PDF files only 
|  EDU domain only 

---

- J Biol Chem, 1995   The prototype mitogen activated protein kinase module is a three kinase cascade consisting of the MAP kinase, extracellular signal regulated protein kinase 1 or ERK2, the MAP **ERK** kinase MEK1 or MEK2, and the MEK kinase, Raf 1 or B Raf.
  This and other MAP kinase modules are thought to be critical signal transducers in major cellular events including proliferation, differentiation, and stress responses.
  To identify novel mammalian MAP kinase modules, polymerase chain reaction was used to isolate a new MEK family member, MEK5, from the rat.
  MEK5 is more closely related to MEK1 and MEK2 than to the other known mammalian MEKs, MKK3 and MKK4.
  MEK5 is thought to lie in an uncharacterized MAP kinase pathway, because MEK5 does not phosphorylate the **ERK** MAP kinase family members ERK1, ERK2, ERK3, JNK SAPK, or p38 HOG1, nor will Raf 1, c Mos, or MEKK1 highly phosphorylate it.
  Alternative splicing results in a 50 kDa alpha and a 40 kDa beta isoform of MEK5.
  MEK5 beta is ubiquitously distributed and primarily cytosolic.
  MEK5 alpha is expressed most highly in liver and brain and is particulate.
  The 23 amino acids encoded by the 5 exon in the larger alpha isoform are similar to a sequence found in certain proteins believed to associate with the **actin** cytoskeleton.
  this alternatively spliced modular domain may lead to the differential subcellular localization of MEK5 alpha.

  - J Cell Biol, 1995   **Integrin function molecular hierarchies of cytoskeletal and signaling molecules.**.
    Integrin receptors play important roles in organizing the **actin** containing cytoskeleton and in signal transduction from the extracellular matrix.
    The initial steps in integrin function can be analyzed experimentally using beads coated with ligands or anti integrin antibodies to trigger rapid focal transmembrane responses.
    A hierarchy of transmembrane actions was identified in this study.
    Simple integrin aggregation triggered localized transmembrane accumulation of 20 signal transduction molecules, including RhoA, Rac1, Ras, Raf, MEK, **ERK**, and JNK.
    In contrast, out of eight cytoskeletal molecules tested, only tensin coaccumulated.
    Integrin aggregation alone was also sufficient to induce rapid activation of the JNK pathway, with kinetics of activation different from those of **ERK**.
    The tyrosine kinase inhibitors herbimycin A or genistein blocked both the accumulation of 19 out of 20 signal transduction molecules and JNK and **ERK** mediated signaling.
    Cytochalasin D had identical effects.
    . . . three other tyrosine kinase inhibitors did not.
    The sole exception among signaling molecules was the kinase pp125FAK which continued to coaggregate with alpha 5 beta 1 integrins even in the presence of these inhibitors.
    Tyrosine kinase inhibition also failed to block the ability of ligand occupancy plus integrin aggregation to trigger transmembrane accumulation of the three cytoskeletal molecules talin, alpha actinin, and vinculin.
    these molecules accumulated even in the presence of cytochalasin D.
    However, it was necessary to fulfill all four conditions,i.e., integrin aggregation, integrin occupancy, tyrosine kinase activity, and **actin** cytoskeletal integrity, to achieve integrin mediated focal accumulation of other cytoskeletal molecules including F **actin** and paxillin.
    Integrins therefore mediate a transmembrane hierarchy of molecular responses.

    - Cancer Res, 1995   **SCH 51344 inhibits ras transformation by a novel mechanism.**.
      A pyrazolo quinoline compound, 6 methoxy 4 2 2 hydroxyethoxyl ethyl amino 3 methyl 1M pyrazo lo b quinoline SCH 51344 , was identified based on its ability to derepress human smooth muscle alpha **actin** promoter activity in ras transformed cells.
      In this study, we show that SCH 51344 reverts several key aspects of ras transformation, such as morphological changes, **actin** filament organization, and anchorage independent growth, and also inhibits Val 12 Ras induced maturation of Xenopus oocytes.
      SCH 51344 is also a potent inhibitor of the anchorage independent growth of human tumor lines known to contain multiple genetic alterations in addition to activated ras genes.
      We have sought to determine whether SCH 51344 disrupts the signaling pathway that activates mitogen activated protein MAP kinase or extracellular signal regulated kinase **ERK** in normal and ras transformed fibroblast cells.
      NIH 3T3 cells transformed by different oncogenes, which have products that participate at different steps of the Ras signaling pathway, were tested in a soft agar colony formation assay to determine which step of the pathway is inhibited by SCH 51344.
      Our results indicate that SCH 51344 inhibits the ability of v abl, v mos, H ras, v raf, and mutant active MAP kinase kinase transformed NIH 3T3 cells to grow in soft agar.
      Only v fos transformed cells were found to be resistant to the treatment of SCH 51344.
      SCH 51344 treatment had very little effect, if any, on the activation of MAP kinase kinase, MAP kinase, and p90RSK activity in response to growth factor stimulation.
      Treatment of ras transformed cells with SCH 51344 led to stimulation of serum response factor DNA binding activity and activation of serum response element dependent gene transcription, accounting for its ability to activate alpha **actin** promoter activity in ras transformed cells.
      Our results indicate that SCH 51344 inhibits ras transformation by a novel mechanism and acts at a point either downstream or parallel to extracellular signal regulated kinase dependent Ras signaling pathway.

      - Science, 1995   Members of the Rho family of small guanosine triphosphatases regulate the organization of the **actin** cytoskeleton.
        Rho controls the assembly of **actin** stress fibers and focal adhesion complexes, Rac regulates **actin** filament accumulation at the plasma membrane to produce lamellipodia and membrane ruffles, and Cdc42 stimulates the formation of filopodia.
        When microinjected into quiescent fibroblasts, Rho, Rac, and Cdc42 stimulated cell cycle progression through G1 and subsequent DNA synthesis.
        Furthermore, microinjection of dominant negative forms of Rac and Cdc42 or of the Rho inhibitor C3 transferase blocked serum induced DNA synthesis.
        Unlike Ras, none of the Rho GTPases activated the mitogen activated protein kinase cascade that contains the protein kinases c Raf1, MEK , and **ERK** .
        Instead, Rac and Cdc42, but not Rho, stimulated a distinct MAP kinase, the c Jun kinase JNK SAPK .
        Rho, Rac, and Cdc42 control signal transduction pathways that are essential for cell growth.

        - Virology, 1995   **Intracellular internalization and signaling pathways triggered by the large subunit of HSV 2 ribonucleotide reductase ICP10 .**.
          The large subunit of the HSV 2 ribonucleotide reductase RR ICP10 is a chimera consisting of a serine threonine Ser Thr protein kinase domain at the amino terminus and the RR domain at the carboxy terminus.
          Transformed human cells that constitutively express ICP10 JHLa1 were stained with anti LA 1 antibody recognizes ICP10 amino acids 13 26 and immunogold conjugated goat anti rabbit IgG and were examined by electron microscopy.
          ICP10 associated gold particles were observed on the cell surface and in structures with ultrastructural characteristics of endocytic vesicles, multivesicular bodies, and lysosomes, consistent with endocytic internalization.
          ICP10 was also associated with the cytoskeleton fraction of JHLa1 cells and, at least in part, it colocalized with **actin** filaments.
          This was evidenced by immunoprecipitation of 35S methionine labeled cell fractions and immunofluorescent staining of Triton treated cells with anti LA 1 antibody and phalloidin.
          Endocytic localization of gold particles was not seen in cells that constitutively express the ICP10 transmembrane TM deleted mutant p139TM JHL15 .
          p139TM did not associate with the cytoskeleton and was almost entirely localized within the cytoplasm.
          raf and **Erk** evidenced decreased mobility consistent with an activated state in JHLa1, but not JHL15, cells, and chloramphenicol acetyl transferase CAT expression from a c fos cat hybrid construct was significantly increased in JHLa1 but not JHL15 cells.
          The data indicate that effector molecules downstream of ras are activated in JHLa1 cells and the ICP10 TM segment plays a critical role in ICP10 intracellular localization and its ability to activate signaling pathways.
          This behavior is analogous to that of an activated growth factor receptor kinase.

          - Cell Growth Differ, 1995   **Transformation resistant mos revertant is unable to activate MAP kinase kinase in response to v mos or v raf.**.
            To study the mechanism by which v mos induces cell transformation, we generated a transformed rat cell line DTM containing two functional copies of mos, one encoding the p37v mos of the m1 wild type strain of Moloney murine sarcoma virus Mo MuSV and the other the p85gag mos fusion protein of the ts110 mutant of Moloney murine sarcoma virus.
            Subsequently, we isolated a revertant cell line F 1 following transfection of DTM with a mutant retroviral construct pIC4Neo carrying a selectable marker.
            Like DTM, the F 1 revertant contained two integrated copies of v mos, expressed mos containing viral RNA, and contained rescuable transforming viruses.
            The revertant did not grow in soft agar, showed a greatly reduced ability to form tumors in nude mice, and exhibited organized tubulin and **actin** structures similar to those found in normal cells.
            Revertant cells were resistant to retransformation by v mos and v raf but could be retransformed by v ras.
            MAP kinase **ERK** 2 and MAP kinase kinase MKK 1 activity, which are constitutively elevated in v mos and v raf transformed cells, exhibits levels in the F 1 revertant similar to those seen in nontransformed cells.
            F 1 and normal REF 1 cells express elevated levels of protein phosphatases in comparison to DTM cells.
            In vivo treatment with okadaic acid, a potent protein phosphatase inhibitor, leads to an increase in MKK 1 and MAP kinase activity in F 1 cells but not in REF 1.
            The results support the hypothesis that mos acts through the MAP kinase cascade MKK 1 and **ERK** 2 to induce cell transformation.
            blocking v mos activation of that cascade possibly because of increased levels of phosphatase prevents transformation.
