# Supplementary material for: Content-rich biological network constructed by mining PubMed abstracts
Source: BMC Bioinformatics. 2004 Oct 8;5:147. doi: 10.1186/1471-2105-5-147 (PMC528731; doi:10.1186/1471-2105-5-147)
Supplement: Additional File 5 — The original Chilibot query results of the term "long-term potentiation (LTP)" and 22 other terms, limiting the latest references analyzed to the years 1990, 1995, 2000, and 2004. [file 1471-2105-5-147-S5.bz2 › chilibotAdditionalFile5/ltp1995/html/ERK_ACTININ.html]

 


 **ERK** and **ACTININ** 
  
Found 1 abstracts in PubMed,  **1 abstracts were retrieved and analyzed**.  


---

 Search Google  |
 PDF files only 
|  EDU domain only 

---

- J Cell Biol, 1995   **Integrin function molecular hierarchies of cytoskeletal and signaling molecules.**.
  Integrin receptors play important roles in organizing the actin containing cytoskeleton and in signal transduction from the extracellular matrix.
  The initial steps in integrin function can be analyzed experimentally using beads coated with ligands or anti integrin antibodies to trigger rapid focal transmembrane responses.
  A hierarchy of transmembrane actions was identified in this study.
  Simple integrin aggregation triggered localized transmembrane accumulation of 20 signal transduction molecules, including RhoA, Rac1, Ras, Raf, MEK, **ERK**, and JNK.
  In contrast, out of eight cytoskeletal molecules tested, only tensin coaccumulated.
  Integrin aggregation alone was also sufficient to induce rapid activation of the JNK pathway, with kinetics of activation different from those of **ERK**.
  The tyrosine kinase inhibitors herbimycin A or genistein blocked both the accumulation of 19 out of 20 signal transduction molecules and JNK and **ERK** mediated signaling.
  Cytochalasin D had identical effects.
  . . . three other tyrosine kinase inhibitors did not.
  The sole exception among signaling molecules was the kinase pp125FAK which continued to coaggregate with alpha 5 beta 1 integrins even in the presence of these inhibitors.
  Tyrosine kinase inhibition also failed to block the ability of ligand occupancy plus integrin aggregation to trigger transmembrane accumulation of the three cytoskeletal molecules talin, alpha **actinin**, and vinculin.
  these molecules accumulated even in the presence of cytochalasin D.
  However, it was necessary to fulfill all four conditions,i.e., integrin aggregation, integrin occupancy, tyrosine kinase activity, and actin cytoskeletal integrity, to achieve integrin mediated focal accumulation of other cytoskeletal molecules including F actin and paxillin.
  Integrins therefore mediate a transmembrane hierarchy of molecular responses.
