# Supplementary material for: Content-rich biological network constructed by mining PubMed abstracts
Source: BMC Bioinformatics. 2004 Oct 8;5:147. doi: 10.1186/1471-2105-5-147 (PMC528731; doi:10.1186/1471-2105-5-147)
Supplement: Additional File 5 — The original Chilibot query results of the term "long-term potentiation (LTP)" and 22 other terms, limiting the latest references analyzed to the years 1990, 1995, 2000, and 2004. [file 1471-2105-5-147-S5.bz2 › chilibotAdditionalFile5/ltp1995/html/ERK_SYNAPTOPHYSIN.html]

 


 **ERK** and **SYNAPTOPHYSIN** 
  
Found 1 abstracts in PubMed,  **1 abstracts were retrieved and analyzed**.  


---

 Search Google  |
 PDF files only 
|  EDU domain only 

---

- Brain Res, 1995   **Denervation induced abnormal phosphorylation in hippocampal neurons.**.
  This study demonstrates that combined dopaminergic and cholinergic denervation of the hippocampus results in the appearance of morphologically altered, Tau reactive, apical dendrites of granule cells in the rat dentate gyrus.
  The denervated granule cells and their apical dendrites also display immunoreactivity to a mitogen activated protein kinase, **ERK** 1, and also evidence of abnormal phosphorylation of these dendrites as revealed by SMI 31 immunoreactivity.
  Dopaminergic denervation alone also causes mitogen activated protein kinase reactivity without the Tau reactive apical dendrities.
  These results suggest an analogy to **synaptophysin** loss and the appearance of dendritic threads described in Alzheimer s disease AD , as an early stage in the formation of neurofibrillary tangles NFT .
  This is the first animal model in which abnormal phosphorylation of Tau has been shown to be produced experimentally in vivo.
