# Supplementary material for: Content-rich biological network constructed by mining PubMed abstracts
Source: BMC Bioinformatics. 2004 Oct 8;5:147. doi: 10.1186/1471-2105-5-147 (PMC528731; doi:10.1186/1471-2105-5-147)
Supplement: Additional File 5 — The original Chilibot query results of the term "long-term potentiation (LTP)" and 22 other terms, limiting the latest references analyzed to the years 1990, 1995, 2000, and 2004. [file 1471-2105-5-147-S5.bz2 › chilibotAdditionalFile5/ltp1995/html/CREB_PKC.html]

 


 **CREB** and **PKC** 
  
Found 7 abstracts in PubMed,  **7 abstracts were retrieved and analyzed**.  


---

 Search Google  |
 PDF files only 
|  EDU domain only 

---

**Interactive relationship** (e.g. stimulation, inhibition, etc)

- This conclusion is based on abrogation of sIg induced  **CREB**  Ser133 phosphorylation by long term phorbol ester treatment to deplete  **PKC** , and mimicking of sIg induced  **CREB**  phosphorylation and CRE dependent gene expression by short term  **PKC**  agonism.  Ref: 7836756 J Immunol, 1995
- However, this paper also shows that 12 O tetradecanoylphorbol 13 acetate TPA which activates  **PKC**  also leads to the phosphorylation of  **CREB**  in oPT cells, suggesting the potential involvement of other signal transduction pathways in the transcriptional regulation of these cells.  Ref: 7827622 J Neuroendocrinol, 1994

- :-)

  **Parallel relationship** (e.g. studied together, co-existance, homology, etc.)

  - Furthermore, CD40 ligand CD40L and LPS, two  **PKC**  independent forms of B cell stimulation, failed to induce phosphorylation of  **CREB**  Ser133.  Ref: 7836756 J Immunol, 1995
  - Taken together with  **PKC**  mediation of  **CREB**  Ser133 phosphorylation in B cells, these results suggest that the dominant mode of  **CREB**  regulation is cell type specific.  Ref: 7836756 J Immunol, 1995
  - The presence and activity of  **PKC** , PKA, and P  **CREB**  in developing chicken skin are further characterized by immunoblot, kinase activity, and gel shift assays.  Ref: 7556946 Dev Biol, 1995
