# Supplementary material for: Content-rich biological network constructed by mining PubMed abstracts
Source: BMC Bioinformatics. 2004 Oct 8;5:147. doi: 10.1186/1471-2105-5-147 (PMC528731; doi:10.1186/1471-2105-5-147)
Supplement: Additional File 5 — The original Chilibot query results of the term "long-term potentiation (LTP)" and 22 other terms, limiting the latest references analyzed to the years 1990, 1995, 2000, and 2004. [file 1471-2105-5-147-S5.bz2 › chilibotAdditionalFile5/ltp1995/html/CREB_PKA.html]

 


 **CREB** and **PKA** 
  
Found 38 abstracts in PubMed,  **30 abstracts were retrieved and analyzed**.  


---

 Search Google  |
 PDF files only 
|  EDU domain only 

---

**Interactive relationship** (e.g. stimulation, inhibition, etc)

- The lack of  **CREB**  mediated transcriptional stimulation is due to the presence of a heat stable inhibitor of  **PKA**  that prevents activation of  **PKA**  and subsequent  **CREB**  phosphorylation in the nucleus.  Ref: 7799950 Mol Cell Biol, 1995
- These data suggest that specific factors interacting with both the PEPCK TATA region and the  **CREB**  activation domain are required for insulin inhibition of  **PKA**  induced transcription.  Ref: 8182041 J Biol Chem, 1994
- Transcriptional activation by this site, although sensitive to  **PKA**  antagonism, is not blocked by  **CREB**  mutants.  Ref: 1331087 J Biol Chem, 1992
- Our results indicate that the minimal requirements for induction of PEPCK by  **PKA**  and inhibition by insulin include 1 the  **CREB**  activation domain, 2 the PEPCK TATA sequence, and 3 insulin responsive hepatoma cells.  Ref: 8182041 J Biol Chem, 1994
- The CBP protein mediates  **PKA**  induced transcription by binding to the  **PKA**  phosphorylated activation domain of  **CREB** .  Ref: 8545107 Oncogene, 1995
- Remarkably, monomeric  **CREB**  acts as a potent, constitutive activator under conditions in which native  **CREB**  is inducible by  **PKA** .  Ref: 7935435 Mol Cell Biol, 1994
- The CaMKIV mediated gene induction requires the activity of  **CREB**  ATF family members but is independent of  **PKA**  activity.  Ref: 8065343 Mol Cell Biol, 1994
- This work establishes that  **CREB**  contains distinct basal and  **PKA**  activated domains, that they operate independently for both loss of function and gain of function,.  Ref: 8394325 J Biol Chem, 1993
- The transcriptional transactivation functions of  **CREB**  are activated by its phosphorylation by cAMP dependent protein kinase A  **PKA** .  Ref: 7799950 Mol Cell Biol, 1995
- The ability of okadaic acid to enhance  **PKA**  stimulated transcription in vitro was entirely dependent on the presence of  **CREB**  in the reactions.  Ref: 8386317 Mol Cell Biol, 1993
- The increase in LPL production was shown to be linked to the stimulation of the  **PKA**  signal transduction pathway and was apparently transmitted via the transcription factor  **CREB** .  Ref: 1327133 Biochemistry, 1992
- Two glutamine rich domains that are important for native,  **PKA**  inducible  **CREB**  activity are required for the constitutive activity of monomeric  **CREB** .  Ref: 7935435 Mol Cell Biol, 1994
- Likewise, okadaic acid prevents the dephosphorylation of  **PKA**  phosphorylated  **CREB**  in rat liver nuclear extracts and enhances the ability of  **PKA**  to stimulate transcription from the PEPCK promoter in cell free reactions.  Ref: 8386317 Mol Cell Biol, 1993
- H4 hepatoma cells were treated with or without insulin following cotransfection with chloramphenicol acetyltransferase reporter genes and expression vectors coding for the cAMP response element binding protein  **CREB**  activation domain fused to the GAL4 DNA binding domain CRG and the catalytic subunit of  **PKA** .  Ref: 8182041 J Biol Chem, 1994
- Mutation of the Glu CRE octamer attenuates both the binding of  **CREB**  and cAMP dependent  **PKA**  stimulated transcriptional activity in transient transfection experiments but does not affect the binding of adjacent  **CREB**  associated proteins.  Ref: 8413297 Mol Cell Biol, 1993
- The cAMP response element binding protein  **CREB**  is generally considered to be responsive to elevation of cAMP through the activity of protein kinase A  **PKA** .  Ref: 7836756 J Immunol, 1995
- Cyclic AMP cAMP dependent protein kinase A  **PKA**  stimulates the transcription of many eucaryotic genes by catalyzing the phosphorylation of the cAMP regulatory element binding protein  **CREB** .  Ref: 8386317 Mol Cell Biol, 1993
- calcium stimulated  **CREB**  serine 133 phosphorylation is mediated by a calcium activated kinase and is not dependent on the cAMP dependent protein kinase  **PKA** .  Ref: 7876182 J Biol Chem, 1995
- Examination of a series of  **CREB**  deletion mutants mapped basal activity to interacting domains, located on either side of the previously identified  **PKA**  activation domain amino acids 98 142.  Ref: 8394325 J Biol Chem, 1993
- These findings support previous observations that nuclear PP2A is the primary phosphatase that dephosphorylates  **PKA**  phosphorylated  **CREB** .  Ref: 8065321 Mol Cell Biol, 1994
- We have previously identified a nuclear protein of M r 265K, CBP, that binds specifically to the  **PKA**  phosphorylated form of  **CREB** .  Ref: 7913207 Nature, 1994
- We propose that the hierarchical phosphorylation at the  **PKA**  and GSK 3 sites of  **CREB**  are essential for cAMP control of  **CREB** .  Ref: 7798217 J Biol Chem, 1994
- From stage 29 on, phosphorylated  **CREB**  P  **CREB** , reflecting the activity of protein kinase A  **PKA** , begins to be seen in placode but not in interplacode epithelia.  Ref: 7556946 Dev Biol, 1995
- The degree of  **CREB**  phosphorylation, assessed with antiserum specific for  **CREB**  phosphorylated at Ser 133, correlated with the amount of  **PKA**  liberated.  Ref: 8336722 Mol Cell Biol, 1993
- **CREB**  activity is modulated by several signalling agents protein kinase A  **PKA** , calcium, and transforming growth factor beta and via functional interactions with cell specific transcription factors.  Ref: 7935435 Mol Cell Biol, 1994
- In contrast to other  **PKA**  mediated cellular responses which are rapid and quantitative, the slow, incremental regulation of  **CREB**  activity by cAMP suggests that multifunctional kinases like  **PKA**  may coordinate cellular responses by dictating the kinetics and stoichiometry of phosphorylation for key substrates like  **CREB** .  Ref: 8336722 Mol Cell Biol, 1993

**Parallel relationship** (e.g. studied together, co-existance, homology, etc.)

- Cyclic AMP cAMP regulates a number of eukaryotic genes by mediating the protein kinase A  **PKA**  dependent phosphorylation of the  **CREB**  transcription factor at Ser 133.  Ref: 8336722 Mol Cell Biol, 1993
- Cyclic AMP regulates the expression of numerous genes, for example, through the protein kinase A  **PKA**  mediated phosphorylation of transcription factor  **CREB**  at Ser 133.  Ref: 8028671 Nature, 1994
- In the context of a GAL4  **CREB**  fusion protein in which the DNA binding bZIP domain of  **CREB**  is replaced by GAL4 binding domain, a single amino acid substitution of serine 133, phosphorylated by  **PKA**  and critical for the transactivation function of  **CREB** , attenuates both Tax and  **PKA**  mediated transcriptional responses.  Ref: 8549766 FEBS Lett, 1995
- Although the COOH terminal serine of the peptide can be phosphorylated by  **PKA**  and several other kinases, the phospho  **CREB**  peptide is specific for GSK 3 with Kms of 140 and 200 microM for GSK 3 alpha and GSK 3 beta isoforms, respectively.  Ref: 7978284 Anal Biochem, 1994
- Induction by glucocorticoid, in the absence or presence of  **PKA** , was not affected by CRG, indicating that interaction of GRU bound factors with  **CREB**  is not required for glucocorticoid induction of PEPCK.  Ref: 8114762 Mol Endocrinol, 1993
- Our results indicate that CREM alpha can contribute to  **PKA**  mediated gene activation when selectively heterodimerized with  **CREB** .  Ref: 7961842 J Biol Chem, 1994
- Modulation of Tax and  **PKA**  mediated expression of HTLV I promoter via cAMP response element binding and modulator proteins  **CREB**  and CREM.  Ref: 8549766 FEBS Lett, 1995
- The presence and activity of PKC,  **PKA** , and P  **CREB**  in developing chicken skin are further characterized by immunoblot, kinase activity, and gel shift assays.  Ref: 7556946 Dev Biol, 1995
- Here, we show that CBP and p300 have similar binding affinity for the  **PKA**  phosphorylated form of  **CREB** ,.  Ref: 7870179 Nature, 1995
- However, whether  **CREB**  participates directly in basal  **PKA**  independent transcription is still an open question, and existing studies conflict over the identification of putative basal activation domains.  Ref: 8394325 J Biol Chem, 1993
- Finally, when  **PKA**  phosphorylated  **CREB**  was treated with immunopurified PP2A and PP1, the PP2A treated  **CREB**  did not stimulate transcription from the PEPCK promoter in vitro.  Ref: 8386317 Mol Cell Biol, 1993
- Deletion or point mutation of the TCATT motif located on the 3 side of the CRE octamer results in enhanced transcriptional responses to  **PKA** , suggesting that the  **CREB**  associated proteins decrease the ability of  **CREB**  to mediate  **PKA**  stimulated transcription.  Ref: 8413297 Mol Cell Biol, 1993
- Two models have been proposed to explain how CREM alpha could prevent the activation of genes by  **PKA**  phosphorylated  **CREB**  inhibitory CREM alpha homodimers could prevent occupancy of the CRE by  **CREB** , or CREM alpha could block gene activation by forming non functional  **CREB** .CREM alpha heterodimers.  Ref: 7961842 J Biol Chem, 1994
- **CREB**  is activated through phosphorylation by protein kinase A  **PKA** , but precisely how phosphorylation stimulates  **CREB**  function is unknown.  Ref: 7913207 Nature, 1994
- Activation of these receptors stimulates gene expression via the cAMP  **PKA**  pathway and the transacting factor  **CREB** .  Ref: 8380441 J Neurochem, 1993
- In this study, we test the hypothesis that the stoichiometry and kinetics of  **CREB**  phosphorylation are determined by the liberation and subsequent translocation of  **PKA**  catalytic subunit C subunit into the nucleus.  Ref: 8336722 Mol Cell Biol, 1993
- These results demonstrate that, consistent with its inhibitory effect on the activation of  **PKA**  within oPT, melatonin prevents or reverses the phosphorylation of  **CREB**  induced by activation of the cyclic AMP signal transduction pathway.  Ref: 7827622 J Neuroendocrinol, 1994
- Deletion of C terminal regions of the  **CREB**  activation domain from CRG diminished basal activation without affecting induction by  **PKA** .  Ref: 8114762 Mol Endocrinol, 1993
- By selectively perturbing molecular function of Gs, the catalytic subunit of  **PKA**  C, or the nuclear factor  **CREB** , in cells through microinjection of inhibitory agents specific for these molecules or activated forms of these molecules, we have obtained evidence for a requirement for the function of each of these molecules in the hormonal stimulation of cAMP regulated genes.  Ref: 7935349 Mol Cell Biochem, 1993
- We show that  **CREB**  and CREM bind all three CRE sequences of the HTLV I promoter which are important determinants in Tax elicited transactivation as well as  **PKA**  mediated activation of the HTLV I promoter.  Ref: 8549766 FEBS Lett, 1995
- The 265K nuclear protein CBP was initially identified as a co activator for the protein kinase A  **PKA**  phosphorylated form of the transcription factor  **CREB** .  Ref: 7870179 Nature, 1995
- We find that  **CREB**  fails to transactivate a CRE containing somatostatin chloramphenicol acetyltransferase reporter even when coexpressed with the catalytic subunit of  **PKA** .  Ref: 7799950 Mol Cell Biol, 1995
- Small t also inhibited the dephosphorylation of cAMP dependent protein kinase  **PKA**  phosphorylated  **CREB**  in rat liver nuclear extracts.  Ref: 8065321 Mol Cell Biol, 1994
- These data suggest that small t enhances Bt2cAMP stimulated gene transcription by inhibiting the dephosphorylation of  **PKA**  phosphorylated  **CREB**  by nuclear PP2A.  Ref: 8065321 Mol Cell Biol, 1994
- The present study examines the expression, distribution, and phosphorylation in the developing murine palate of a substrate for  **PKA**  known as the cAMP response element binding protein  **CREB** .  Ref: 7622577 J Cell Physiol, 1995
- Cells stimulated with forskolin responded with a robust time and dose dependent increase in nuclear phospho  **CREB**  immunoreactivity P  **CREB**  ir, confirming that activation of this transcription factor occurred through the cyclic AMP  **PKA**  pathway.  Ref: 7827622 J Neuroendocrinol, 1994
- Phosphorylation of  **CREB**  cyclic AMP cAMP response element CRE binding protein by cAMP dependent protein kinase  **PKA**  leads to the activation of many promoters containing CREs.  Ref: 8065343 Mol Cell Biol, 1994
- In summary,  **PKA**  signaling and transacting factors such as  **CREB** , Fos and Jun are probably involved in transcriptional inhibition of GnRH gene by hCG in GT1 7 neurons.  Ref: 7664977 Mol Cell Endocrinol, 1995
- Activation of protein kinase A  **PKA**  by cAMP results in phosphorylation of cAMP response element binding protein  **CREB**  and induction of specific gene expression.  Ref: 8394325 J Biol Chem, 1993
- Nuclear PP2A appears to be the primary phosphatase that dephosphorylates  **PKA**  phosphorylated  **CREB** .  Ref: 8386317 Mol Cell Biol, 1993
- These results indicate that  **CREB**  is directly involved in basal and  **PKA**  induced expression of PEPCK,.  Ref: 8114762 Mol Endocrinol, 1993
