# Supplementary material for: Content-rich biological network constructed by mining PubMed abstracts
Source: BMC Bioinformatics. 2004 Oct 8;5:147. doi: 10.1186/1471-2105-5-147 (PMC528731; doi:10.1186/1471-2105-5-147)
Supplement: Additional File 5 — The original Chilibot query results of the term "long-term potentiation (LTP)" and 22 other terms, limiting the latest references analyzed to the years 1990, 1995, 2000, and 2004. [file 1471-2105-5-147-S5.bz2 › chilibotAdditionalFile5/ltp1995/html/CREB_NMDA.html]

 


 **CREB** and **NMDA** 
  
Found 6 abstracts in PubMed,  **6 abstracts were retrieved and analyzed**.  


---

 Search Google  |
 PDF files only 
|  EDU domain only 

---

**Interactive relationship** (e.g. stimulation, inhibition, etc)

- These results support the proposal that an intracerebroventricular injection of  **NMDA**  may selectively potentiate DNA binding activities of both AP1 and  **CREB**  through activation of the  **NMDA**  receptor complex in mouse brain.  Ref: 8035176 J Neurochem, 1994
- These results support the proposal that an intracerebroventricular injection of  **NMDA**  may selectively potentiate DNA binding activities of both AP1 and  **CREB**  through in vivo activation of the  **NMDA**  receptor complex in the murine brain.  Ref: 7975930 Yakubutsu Seishin Kodo, 1994
- An injection of  **NMDA**  increased binding of both probes for activator protein 1 AP1 and cyclic AMP response element binding protein  **CREB**  1 to 5 h after the injection compared with that of saline, in a dose dependent manner at doses from 0.05 to 0.4 micrograms.  Ref: 7975930 Yakubutsu Seishin Kodo, 1994
- L Glu,  **NMDA** , and KA evoked a dose and time dependent increase in AP 1 DNA binding activity and had no effect on  **CREB**  binding.  Ref: 7650753 J Neurosci Res, 1995
- In contrast,  **NMDA**  virtually did not alter DNA binding activities of both  **CREB**  and Myc in discrete structures of murine brain under similar experimental conditions.  Ref: 7833794 Neurochem Int, 1994
- Potentiation of the AP1 and  **CREB**  binding was prevented in a dose dependent manner by the administration of either of the noncompetitive  **NMDA**  antagonist 5 methyl 1 1 dihydro 5H dibenzo a, d cyclohepten 0 imine, the  **NMDA**  antagonist D, L E 2 amino 4 propyl 5 phosphono 3 pentenoic acid, the glycine antagonist dichlorokynurenic acid, or the proposed polyamine antagonist ifenprodil.  Ref: 7975930 Yakubutsu Seishin Kodo, 1994

**Parallel relationship** (e.g. studied together, co-existance, homology, etc.)

- The effect of L glutamate L Glu and its structural analogs N methyl D aspartate  **NMDA** , quisqualate QA, and kainate KA on the DNA binding activity of the Activator Protein 1 AP 1 and the calcium cAMP Responsive Element Binding Protein  **CREB**  families of transcription factors was examined in cultured chick retinal Müller glia cells.  Ref: 7650753 J Neurosci Res, 1995
- An injection of saline transiently increased binding of both probes for activator protein 1 AP1 and cyclic AMP response element binding protein  **CREB**  30 min after the injection, and  **NMDA**  was effective in inducing a more potent increment of binding of both probes 1 5 h after the injection than did saline.  Ref: 8035176 J Neurochem, 1994
