# Supplementary material for: Content-rich biological network constructed by mining PubMed abstracts
Source: BMC Bioinformatics. 2004 Oct 8;5:147. doi: 10.1186/1471-2105-5-147 (PMC528731; doi:10.1186/1471-2105-5-147)
Supplement: Additional File 5 — The original Chilibot query results of the term "long-term potentiation (LTP)" and 22 other terms, limiting the latest references analyzed to the years 1990, 1995, 2000, and 2004. [file 1471-2105-5-147-S5.bz2 › chilibotAdditionalFile5/ltp1995/html/CREB_AMPA.html]

 


 **CREB** and **AMPA** 
  
Found 1 abstracts in PubMed,  **1 abstracts were retrieved and analyzed**.  


---

 Search Google  |
 PDF files only 
|  EDU domain only 

---

- J Neurosci Res, 1995   **Excitatory amino acid induced AP 1 DNA binding activity in Müller glia.**.
  The effect of L glutamate L Glu and its structural analogs N methyl D aspartate NMDA , quisqualate QA , and kainate KA on the DNA binding activity of the Activator Protein 1 AP 1 and the Ca2ion cAMP Responsive Element Binding Protein **CREB** families of transcription factors was examined in cultured chick retinal Müller glia cells.
  L Glu, NMDA, and KA evoked a dose and time dependent increase in AP 1 DNA binding activity and had no effect on **CREB** binding.
  The order of potency for stimulating AP 1 DNA binding was NMDA > or = Glu > KA >> QA.
  L Glu responses were partially blocked by 6 cyano 7 nitroquinoxaline dione CNQX and by 3 RS 2 carboxypiperazin 4 yl propyl 1 phosphonate CPP indicating that the increase in DNA binding is mediated both by an alpha amino 3 hydroxy 5 methyl 4 isoxazolepropionate **AMPA** low affinity KA and a NMDA subtypes of L Glu receptors.
  Since Müller glia L Glu receptors are probably mediators of the efficacy of the excitatory transmission in the retina, the present findings suggest that a stimulus transcription coupling triggered by L Glu in the glial cells might have a role in the long term modulation of these synapses.
