# Supplementary material for: Content-rich biological network constructed by mining PubMed abstracts
Source: BMC Bioinformatics. 2004 Oct 8;5:147. doi: 10.1186/1471-2105-5-147 (PMC528731; doi:10.1186/1471-2105-5-147)
Supplement: Additional File 5 — The original Chilibot query results of the term "long-term potentiation (LTP)" and 22 other terms, limiting the latest references analyzed to the years 1990, 1995, 2000, and 2004. [file 1471-2105-5-147-S5.bz2 › chilibotAdditionalFile5/ltp1995/html/CREB_ATF.html]

 


 **CREB** and **ATF** 
  
Found 154 abstracts in PubMed,  **30 abstracts were retrieved and analyzed**.  


---

 Search Google  |
 PDF files only 
|  EDU domain only 

---

**Interactive relationship** (e.g. stimulation, inhibition, etc)

- The B motif contains nucleotide sequences known as a cyclic AMP response element or tax response element which binds members of the  **ATF**   **CREB**  family of transcription factors.  Ref: 7745688 J Virol, 1995
- The transcription factors  **ATF**  1 and  **CREB**  1 bind constitutively to the hypoxia inducible factor 1 HIF 1 DNA recognition site.  Ref: 8524640 Nucleic Acids Res, 1995
- In each case, the N terminal domain of E1A243, which mediates a direct interaction with YY1, was responsible for disruption of the  **ATF**   **CREB**  YY1 complex.  Ref: 7494244 J Virol, 1995
- Monoclonal antibodies raised against the CRE binding factors  **ATF**  1 and  **CREB**  1 supershifted the constitutive factors  **ATF**  1 and  **CREB**  1 supershifted the constitutive factor, while Jun and Fos family members, which constitute the AP 1 factor, were immunologically undetectable.  Ref: 8524640 Nucleic Acids Res, 1995
- ...  **ATF**   **CREB**  proteins bind exclusively to sequences with adjacent half sites.  Ref: 7630732 Nucleic Acids Res, 1995
- The bZip proteins GCN4 and C EBP differ in their DNA binding specificities GCN4 binds well to the pseudopalindromic AP1 site 5 A4T3G2A1C0T1C2 A3 T4 3 and to the palindromic  **ATF**   **CREB**  sequence 5 A4T3G2A1 C0\*G0 T1 C2 A3 T4 3.  Ref: 7479080 Nucleic Acids Res, 1995
- LEF 1 participates in regulation of the enhancer associated with the T cell receptor TCR alpha gene by inducing a sharp bend in the DNA and facilitating interactions between Ets 1, PEBP2 alpha, and  **ATF**   **CREB** , transcription factors bound at sites flanking the LEF 1 site.  Ref: 7651541 Nature, 1995
- This finding suggested that the mechanism of transcriptional repression by YY1 might involve a direct interaction with members of the  **ATF**   **CREB**  family of transcription factors.  Ref: 7769693 J Virol, 1995
- Transcriptional repression of the c fos gene by YY1 is mediated by a direct interaction with  **ATF**   **CREB** .Transcriptional activation of the mouse c fos gene by the adenovirus 243 amino acid E1A protein requires a binding site for transcription factor YY1 located at 54 of the c fos promoter.  Ref: 7769693 J Virol, 1995
- These elements include a proximal cyclic AMP response element CRE AP 1 like motif TGACATCA that binds c Jun, JunD, CRE binding  **CREB** , and  **ATF**  proteins, a near consensus glucocorticoid response element, and a distal consensus AP 1 site that binds c Fos, Fra 1, and JunD.  Ref: 7791794 Mol Cell Biol, 1995
- Overexpression of YY1 in HeLa cells resulted in repression of a mutant c fos chloramphenicol acetyltransferase reporter that lacked binding sites for YY1, suggesting that repression can be triggered through protein protein interactions with  **ATF**   **CREB**  family members.  Ref: 7769693 J Virol, 1995
- These data support a model in which YY1 binds simultaneously to its own DNA binding site in the c fos promoter and also to adjacent DNA bound  **ATF**   **CREB**  proteins in order to effect repression.  Ref: 7769693 J Virol, 1995
- The factor Atf1, contains a bZIP domain at its C terminus with strong homology to members of the  **ATF**   **CREB**  family of mammalian factors and in vitro binds specifically to  **ATF**  CRE recognition sites.  Ref: 8557039 EMBO J, 1995
- Recombinant  **ATF**  1 and  **CREB**  1 proteins bound HIF1 probes either as homodimers or as heterodimers, indicating a new binding specificity for  **ATF**  1  **CREB**  1.  Ref: 8524640 Nucleic Acids Res, 1995
- These results suggest a model involving the inhibition of IFN gamma AP 1  **CREB**   **ATF**  DNA binding complexes as one of the mechanisms involved in the negative regulatory action of glucocorticoids on IFN gamma gene expression and support the relevance of AP 1  **CREB**   **ATF**  binding factors during the transcriptional activation of the IFN gamma promoter in T cells.  Ref: 7759501 J Biol Chem, 1995
- After 40 min of renal ischemia followed by reperfusion for as little as 5 min, binding of  **ATF**  2 and c Jun... to oligonucleotides containing either an  **ATF**  cAMP response element  **ATF**  CRE or the jun2TRE from the c jun promoter, was significantly increased.  Ref: 8530413 J Biol Chem, 1995

**Parallel relationship** (e.g. studied together, co-existance, homology, etc.)

- While most mutations abolish DNA binding, substitution of a histidine residue results in a GCN4 derivative with  **ATF**   **CREB**  binding specificity.  Ref: 7630732 Nucleic Acids Res, 1995
- The AP 1 and  **ATF**   **CREB**  proteins are structurally related and recognize identical half sites TGAC, but they differ in their requirements for half site spacing.  Ref: 7630732 Nucleic Acids Res, 1995
- We found that Tax enhanced the binding of one member of the  **ATF**   **CREB**  family,  **CREB**  1, to each of the three HTLV I LTR 21 bp repeats but not another member designated CRE BP1 or CREB2.  Ref: 7745688 J Virol, 1995
- The amino acid sequence homology and general structural features of the predicted protein indicate that this gene encodes a general transcription factor belonging to the  **Creb**   **ATF**  subfamily of the bZip super family.  Ref: 8586413 Genomics, 1995
- Furthermore, utilizing electrophoretic mobility shift analysis, we identified activator protein 1 AP 1 cAMP response element binding protein activating transcription factor  **CREB**   **ATF**  binding elements situated in positions of the IFN gamma promoter previously identified as essential for promoter activity.  Ref: 7759501 J Biol Chem, 1995
- The common binding factor in the nuclear extracts was revealed to be an  **ATF**  1  **CREB**  heterodimer by gel retardation assays using specific antibodies.  Ref: 7659506 Nucleic Acids Res, 1995
- The X ray structure of the GCN4 bZIP protein bound to DNA containing the  **ATF**   **CREB**  recognition sequence has been refined at 2.2 A.  Ref: 7500340 J Mol Biol, 1995
- AP 1 proteins may represent a subclass and perhaps evolutionary offshoot of  **ATF**   **CREB**  proteins that can tolerate overlapping half sites.  Ref: 7630732 Nucleic Acids Res, 1995
- This shows that at least one specific member of the  **ATF**   **CREB**  family of transcription factors is involved in mediating transactivation by the HCMV IE86 protein.  Ref: 7666507 J Virol, 1995
- In the absence of E1A243, YY1 represses CRE dependent transcription of c fos by physically interacting with  **ATF**   **CREB**  proteins bound to the 67 CRE.  Ref: 7494244 J Virol, 1995
- The core promoter of the human DNA polymerase beta beta Pol encoding gene POL beta is regulated through cis elements for the  **ATF**   **CREB**  protein s, and GC box binding and initiation site binding proteins.  Ref: 7590351 Gene, 1995
- These results suggest that BLV Tax interacts directly with  **CREB**   **ATF**  like factors to activate viral mRNA transcription.  Ref: 8525616 Virology, 1995
- Gel mobility shift assays with antibodies against CRE binding protein  **CREB**  and activating transcription factor 1  **ATF**  1 showed that both  **CREB**  and  **ATF**  1 interacted with the CRE in MTC cells.  Ref: 7476962 Mol Endocrinol, 1995
- Various members of the AP 1 and  **ATF**   **CREB**  families of transcription factors are targets for E1A dependent regulation, including cJun, the protein product of the c jun proto oncogene.  Ref: 7646484 Bioessays, 1995
- Due to the critical nature of the CRE for the induction of IL 1 beta transcription, an effort was made to determine the importance of the cAMP signaling pathway s by determining whether CRE binding protein  **CREB**  and other  **CREB**  activating transcription factor  **ATF**  family members that responded to cAMP were associated with the DNA protein complex that forms at this site.  Ref: 7594450 J Immunol, 1995
- Moreover, dominant negative mutants of the c Jun proto oncogene were able to mimic the same down regulatory effect exerted by dexamethasone, and mutations that abolished the binding of the AP 1  **CREB**   **ATF**  factors were able to block the glucocorticoid effect.  Ref: 7759501 J Biol Chem, 1995
- Supershift electrophoretic gel mobility shift assays and immunoprecipitation analysis provided further evidence that both  **CREB**  and  **ATF**  1 are present in the complex.  Ref: 7594450 J Immunol, 1995
- Perinatal activation was not impaired when a CRE reporter transgene was assayed in mice that contain a targeted mutation of the CRE binding protein  **CREB**  gene, providing further evidence for functional redundancy among the members of the  **CREB**   **ATF**  gene family.  Ref: 7753790 Proc Natl Acad Sci U S A, 1995
- Here we present evidence that expression of E1A243 leads to relief of YY1 mediated repression by a disruption of the  **ATF**   **CREB**  YY1 complex.  Ref: 7494244 J Virol, 1995
- We conclude that the  **ATF**  1  **CREB**  heterodimer is involved in the constitutive expression of the Na, K ATPase alpha 1 subunit gene.  Ref: 7659506 Nucleic Acids Res, 1995
- Western blot analysis of isolated fractions, using  **CREB**  and  **ATF**  1 specific Ab showed an increased level of these proteins in the enriched fractions.  Ref: 7594450 J Immunol, 1995
- This cDNA contained one extended open reading frame that predicted a protein of 700 amino acids with a basic region and a leucine zipper that is highly similar to members of the  **Creb**   **ATF**  subfamily.  Ref: 8586413 Genomics, 1995
- The AP 1 and  **ATF**   **CREB**  families of eukaryotic transcription factors are dimeric DNA binding proteins that contain the bZIP structural motif.  Ref: 7630732 Nucleic Acids Res, 1995
- In vitro binding studies revealed that the uPA 5 TRE sequence is recognized by the cyclic AMP unresponsive  **ATF**  2 factor, but not by the cyclic AMP inducible  **CREB** .  Ref: 7624151 Oncogene, 1995
- Replacement of two GCN4 residues on this surface Ala244 and Leu247 by their ATF1 counterparts largely converts GCN4 into a protein with  **ATF**   **CREB**  specificity.  Ref: 7630732 Nucleic Acids Res, 1995
- These results suggest that the AP 1 and  **ATF**   **CREB**  proteins differ in positioning a short surface that includes the invariant arginine.  Ref: 7630732 Nucleic Acids Res, 1995
- In vitro and in vivo binding assays were used to demonstrate that YY1 can interact with  **ATF**   **CREB**  proteins, including  **CREB** ,  **ATF**  2, ATFa1, ATFa2, and ATFa3.  Ref: 7769693 J Virol, 1995
- A  **CREB**   **ATF**  element was found to be essential for basal transcription of the flt 1 expression.  Ref: 7499271 J Biol Chem, 1995
- By several biochemical criteria, the GMEBs differed from many of the previously described  **CREB**  CREM  **ATF**  family members.  Ref: 7665613 J Biol Chem, 1995
- Two of the highly related proteins, cyclic AMP responsive element binding protein  **CREB**  and activation transcriptional factor 1  **ATF**  1, have been shown to activate transcription in response to cAMP by interacting with CRE.  Ref: 7751619 J Immunol, 1995
- A new member of the  **ATF**   **CREB**  family of transcription factors, called B  **ATF** , has been isolated from a cDNA library prepared from Epstein Barr virus stimulated human B cells.  Ref: 8570175 Oncogene, 1995
- Here we investigate the distinctions between AP 1 and  **ATF**   **CREB**  proteins by determining the DNA binding properties of mutant and hybrid proteins.  Ref: 7630732 Nucleic Acids Res, 1995
- They further suggest that the  **ATF**   **CREB**  YY1 complex serves as a target for the adenovirus 243 amino acid E1A protein.  Ref: 7769693 J Virol, 1995
- The 46 kDa subunit of X2BP cross reacted with anti rat  **CREB**  polyclonal Abs but not to anti human  **CREB**  Abs in Western analysis and supershift assays, indicating that it may be a novel member of the  **ATF**   **CREB**  family.  Ref: 7594590 J Immunol, 1995
- In this study, we investigated the mechanism by which Tax activates gene expression in conjunction with members of the  **ATF**   **CREB**  family.  Ref: 7745688 J Virol, 1995
- Adenovirus E1A243 disrupts the  **ATF**   **CREB**  YY1 complex at the mouse c fos promoter.The adenovirus E1A243 protein can activate transcription of the mouse c fos gene in a manner that depends on treatment of cells with inducers or analogs of cyclic AMP.  Ref: 7494244 J Virol, 1995
- Comparison of the core promoters from the bovine and human genes revealed striking similarity, including an almost precise match of the tsp, the  **ATF**   **CREB**  binding and Sp1 binding sites, and the spacing separating them.  Ref: 7590351 Gene, 1995
- Determinants of half site spacing preferences that distinguish AP 1 and  **ATF**   **CREB**  bZIP domains.  Ref: 7630732 Nucleic Acids Res, 1995
- In contrast, ICP8 mutant infected cells contained elevated amounts of NF kappa B activity equivalent to WT virus, no induction of Sp1 relative to WT virus, and reduced  **ATF**   **CREB**  activity relative to WT virus.  Ref: 7491786 Virology, 1995
- In human cells the basal transcription of the major promoter is dependent on 4 cis acting elements a TTAAGA motif analogous to the TATA box, two E2F sites that are present as inverted repeats, and an  **ATF**   **CREB**  site.  Ref: 8589638 Biochem Mol Biol Int, 1995
- Similarly, expression of E1A243 in HeLa cells prevented the association of a YY1 VP16 fusion protein with endogenous  **ATF**   **CREB**  proteins bound to the 67 CRE of a transfected c fosCAT reporter plasmid.  Ref: 7494244 J Virol, 1995
- Furthermore, the purified BLV TaxH6 enhances binding of members of the  **CREB**   **ATF**  family of bZip proteins to CRE motifs by interacting with their bZip domains in vitro.  Ref: 8525616 Virology, 1995
- While oligonucleotides containing either legitimate  **ATF**  CRE or AP 1 binding sequences competed for binding, antibody supershift experiments suggested that neither  **CREB**   **ATF**  1 nor AP 1 are major factors binding to IdATF.  Ref: 7565791 Mol Cell Biol, 1995
- The promoter region contains a TATA box, a GC rich region, and putative transcription factor binding elements such as cAMP response element binding protein activating transcription factor  **CREB**   **ATF**  and ets.  Ref: 7499271 J Biol Chem, 1995
- Secondly, analysis of a Fos derivative containing the GCN4 leucine zipper indicates that Fos represents a novel intermediate between AP 1 and  **ATF**   **CREB**  proteins.  Ref: 7630732 Nucleic Acids Res, 1995
