# Supplementary material for: Content-rich biological network constructed by mining PubMed abstracts
Source: BMC Bioinformatics. 2004 Oct 8;5:147. doi: 10.1186/1471-2105-5-147 (PMC528731; doi:10.1186/1471-2105-5-147)
Supplement: Additional File 5 — The original Chilibot query results of the term "long-term potentiation (LTP)" and 22 other terms, limiting the latest references analyzed to the years 1990, 1995, 2000, and 2004. [file 1471-2105-5-147-S5.bz2 › chilibotAdditionalFile5/ltp1995/html/CREB_CAMKII.html]

 


 **CREB** and **CAMKII** 
  
Found 3 abstracts in PubMed,  **3 abstracts were retrieved and analyzed**.  


---

 Search Google  |
 PDF files only 
|  EDU domain only 

---

**Interactive relationship** (e.g. stimulation, inhibition, etc)

- Transient transfection studies revealed that phosphorylation of Ser142 by  **CaMKII**  blocks the activation of  **CREB**  that would otherwise occur when Ser133 is phosphorylated.  Ref: 7958915 Genes Dev, 1994
- When Ser142 was mutated to alanine,  **CREB**  was activated by  **CaMKII** , as well as by CaMKIV.  Ref: 7958915 Genes Dev, 1994
- ...  **CaMKII**  phosphorylates  **CREB**  at Ser133 and a second site, Ser142.  Ref: 7958915 Genes Dev, 1994
- Because both  **CaMKII**  and CaMKIV can phosphorylate  **CREB** , we pursued further the mechanism by which  **CaMKII**  and CaMKIV differentially regulate  **CREB**  activity.  Ref: 7958915 Genes Dev, 1994

**Parallel relationship** (e.g. studied together, co-existance, homology, etc.)

- The results indicate that CaMKIV is much more potent than  **CaMKII**  in activating  **CREB**  in three different cell lines.  Ref: 7958915 Genes Dev, 1994
