# Supplementary material for: Content-rich biological network constructed by mining PubMed abstracts
Source: BMC Bioinformatics. 2004 Oct 8;5:147. doi: 10.1186/1471-2105-5-147 (PMC528731; doi:10.1186/1471-2105-5-147)
Supplement: Additional File 5 — The original Chilibot query results of the term "long-term potentiation (LTP)" and 22 other terms, limiting the latest references analyzed to the years 1990, 1995, 2000, and 2004. [file 1471-2105-5-147-S5.bz2 › chilibotAdditionalFile5/ltp1995/html/CREB_CAMKIV.html]

 


 **CREB** and **CAMKIV** 
  
Found 2 abstracts in PubMed,  **2 abstracts were retrieved and analyzed**.  


---

 Search Google  |
 PDF files only 
|  EDU domain only 

---

**Interactive relationship** (e.g. stimulation, inhibition, etc)

- It was also found in these studies that Ser133 of  **CREB**  is essential for its activation by  **CaMKIV** .  Ref: 7958915 Genes Dev, 1994
- The  **CaMKIV**  mediated gene induction requires the activity of  **CREB**  ATF family members but is independent of PKA activity.  Ref: 8065343 Mol Cell Biol, 1994
- The results indicate that  **CaMKIV**  is much more potent than CaMKII in activating  **CREB**  in three different cell lines.  Ref: 7958915 Genes Dev, 1994
- When Ser142 was mutated to alanine,  **CREB**  was activated by CaMKII, as well as by  **CaMKIV** .  Ref: 7958915 Genes Dev, 1994
- Mutagenesis studies and phosphopeptide mapping analysis demonstrated that in vitro,  **CaMKIV**  phosphorylates  **CREB**  at Ser133 only.  Ref: 7958915 Genes Dev, 1994
- Because both CaMKII and  **CaMKIV**  can phosphorylate  **CREB** , we pursued further the mechanism by which CaMKII and  **CaMKIV**  differentially regulate  **CREB**  activity.  Ref: 7958915 Genes Dev, 1994
