# Supplementary material for: Content-rich biological network constructed by mining PubMed abstracts
Source: BMC Bioinformatics. 2004 Oct 8;5:147. doi: 10.1186/1471-2105-5-147 (PMC528731; doi:10.1186/1471-2105-5-147)
Supplement: Additional File 5 — The original Chilibot query results of the term "long-term potentiation (LTP)" and 22 other terms, limiting the latest references analyzed to the years 1990, 1995, 2000, and 2004. [file 1471-2105-5-147-S5.bz2 › chilibotAdditionalFile5/ltp1995/html/PI-3K_ACTIN.html]

 


 **PI-3K** and **ACTIN** 
  
Found 11 abstracts in PubMed,  **11 abstracts were retrieved and analyzed**.  


---

 Search Google  |
 PDF files only 
|  EDU domain only 

---

**Interactive relationship** (e.g. stimulation, inhibition, etc)

- The inhibitory profiles of WT for pit and  **actin**  ring formation were similar to that for PI 3 kinase  [ **PI-3K** ]  activity in OCLs.  Ref: 7890044 FEBS Lett, 1995
- Phosphatidylinositol 3 kinase PI 3 kinase  [ **PI-3K** ]  has been implicated in cellular events such as mitogenic signaling,  **actin**  organization, and receptor sorting.  Ref: 7629144 J Biol Chem, 1995

**Parallel relationship** (e.g. studied together, co-existance, homology, etc.)

- Furthermore, as Rac has been implicated in at least two diverse cellular responses that are also though to require activation of PI 3 kinase  [ **PI-3K** ]  a reorganization of the  **actin**  cytoskeleton known as membrane ruffling and the neutrophil oxidative burst these results suggest that Rac may be a major effector protein for the PI 3 kinase  [ **PI-3K** ]  signalling pathway in many cell types.  Ref: 7627555 Curr Biol, 1995
- The addition of 0.2 unit mL thrombin caused aggregation dependent redistribution of cytoskeletal proteins  **actin**  binding protein, talin, vinculin, and alpha actinin, glycoproteins GPIIb IIIa, PECAM, and signaling molecules PI3 kinase  [ **PI-3K** ] , pp60c src to the cytoskeletal fraction of platelets.  Ref: 7626626 Biochemistry, 1995
- Aggregation 60 80% of control was restored if 1 unit mL thrombin was added, but there was no corresponding redistribution of  **actin**  binding protein, talin, vinculin, alpha actinin, GPIIb IIIa, PECAM, PI3 kinase  [ **PI-3K** ] , and pp60c src to the cytoskeleton.  Ref: 7626626 Biochemistry, 1995
- This coprecipitation was observed even after depolymerization of  **actin**  fibres, suggesting that PI 3 kinase  [ **PI-3K** ]  binds directly to alpha actinin.  Ref: 8093010 Biochem J, 1994
