# Supplementary material for: Content-rich biological network constructed by mining PubMed abstracts
Source: BMC Bioinformatics. 2004 Oct 8;5:147. doi: 10.1186/1471-2105-5-147 (PMC528731; doi:10.1186/1471-2105-5-147)
Supplement: Additional File 5 — The original Chilibot query results of the term "long-term potentiation (LTP)" and 22 other terms, limiting the latest references analyzed to the years 1990, 1995, 2000, and 2004. [file 1471-2105-5-147-S5.bz2 › chilibotAdditionalFile5/ltp1995/html/PKC_PKA.html]

 


 **PKC** and **PKA** 
  
Found 381 abstracts in PubMed,  **30 abstracts were retrieved and analyzed**.  


---

 Search Google  |
 PDF files only 
|  EDU domain only 

---

**Interactive relationship** (e.g. stimulation, inhibition, etc)

- Simultaneous activation of both  **PKA**  and  **PKC**  pathways resulted in further depletion of  **PKA**  C and an important loss 50% of RII, a subunit which was enhanced by the activation of either system alone.  Ref: 8559283 Neuroendocrinology, 1995
- In conclusion, these data demonstrate that the pituitary cell contents of RI, RII, and C subunits of  **PKA**  are regulated under the activation of  **PKA**  itself as well as  **PKC**  in a manner that can exhibit further alteration when both systems come simultaneously into play.  Ref: 8559283 Neuroendocrinology, 1995
- We have previously shown that direct activation of protein kinase A  **PKA**  and protein kinase C  **PKC**  induced changes in the expression of genes coding for  **PKA**  RII beta and C alpha subunit isoforms in cultured anterior pituitary cells, suggesting the possibility of interconnected regulation at this point.  Ref: 8559283 Neuroendocrinology, 1995
- ... crg 2 mRNA accumulation was significantly inhibited by  **PKC**  inhibitors, but minimally or not affected by inhibitors of tyrosine kinase or  **PKA**  activity.  Ref: 8903850 Brain Behav Immun, 1995
- Similarly, short term treatment with phorbol 12 myristate 13 acetate, which mimics diacylglycerol activation of protein kinase C  **PKC** , or with agentsi.e. forskolin, 8 4 chlorophenylthio cAMP, and isobutylmethylxanthine that lead to activation of cAMP dependent protein kinase  **PKA**  also stimulated transport by NHE 1 and NHE 2 but depressed that by NHE 3.  Ref: 7493949 J Biol Chem, 1995
- Depending on its mode, this interaction may trigger a wide range of signal transmitting systems, in which membrane components receptors, G and Ras proteins, adaptor proteins, tyrosine specific protein kinases and cytoplasmic protein kinases  **PKC** ,  **PKA** , MAR kinase cascade components play a crucial role.  Ref: 8600989 Biokhimiia, 1995
- The results suggested that subcellular distribution and activity of  **PKA**  in cardiomyocytes may be regulated by  **PKC** .  Ref: 8745576 Chin Med Sci J, 1995
- Finally, gonadotropin releasing hormone, a neuropeptide that has the potentiality to activate both  **PKA**  and  **PKC**  signaling in gonadotropes, was able to alter  **PKA**  subunit cell content  **PKA**  C was significantly reduced at either a subliminal 0.1 nM or maximal 10 nM concentration.  Ref: 8559283 Neuroendocrinology, 1995
- Control of AA release by  **PKA** , is mediated both by mechanisms which involve blunting of PLC activity and mechanisms which are downstream from the PLC  **PKC**  cascade.  Ref: 7548185 Biochim Biophys Acta, 1995
- **PKC**  dependent phosphorylation of a serine, located in the highly conserved cytoplasmatic region between the third and the fourth transmembraneous domain has been found to be a prerequisite for  **PKA**  modulation of the alphaIIA isoform.  Ref: 7478928 Pflugers Arch, 1995
- The change of  **PKA**  activity induced by PMA was abolished completely by pretreatment of polymyxin B or depletion of protein kinase C  **PKC** .  Ref: 8745576 Chin Med Sci J, 1995

**Parallel relationship** (e.g. studied together, co-existance, homology, etc.)

- Consistent with this idea is the fact that the phosphorylation sites for  **PKA**  and  **PKC**  and the ADP ribosylation sites occur on different polypeptides comprising the channel complex.  Ref: 8569078 Kidney Int, 1995
- We demonstrate that in immortalized normal human tracheal epithelial cells NT 1 and 56FHTE8o 14C labeled glycoconjugate secretion may be regulated independently by agonists of the protein kinase A  **PKA**  and protein kinase C  **PKC**  signaling pathways.  Ref: 8572248 Am J Physiol, 1995
- In astrocytes, MuRantes mRNA accumulation in response to NDV was completely blocked by tyrosine kinase inhibitors, and partially by  **PKC**  and  **PKA**  inhibitors.  Ref: 8903850 Brain Behav Immun, 1995
- To delineate the signaling circuitry, a series of synthetic PTH and PTHRP fragments were used that stimulate the adenylate cyclase cAMP  **PKA**  and or phospholipase C diacylglycerol  **PKC**  pathways.  Ref: 7650018 J Biol Chem, 1995
- An investigation of signalling pathways involved in the regulation of E2DH activity revealed that stimulation of both the protein kinase C  **PKC**  and  **PKA**  pathways may be involved in regulation of E2DH activity.  Ref: 8547183 J Steroid Biochem Mol Biol, 1995
- The presence and activity of  **PKC** ,  **PKA** , and P CREB in developing chicken skin are further characterized by immunoblot, kinase activity, and gel shift assays.  Ref: 7556946 Dev Biol, 1995
- A model of how the well concerted  **PKA**  and  **PKC**  signaling may be involved in the formation and size regulation of dermal condensation is presented.  Ref: 7556946 Dev Biol, 1995
- We observed that activation of  **PKA**  causes diffused expression of N CAM in mesenchyme while activation of  **PKC**  causes the disappearance of N CAM in precondensed mesenchymal regions.  Ref: 7556946 Dev Biol, 1995
- These data support the concept that the NHE isoforms are differentially responsive to agonists of the  **PKA**  and  **PKC**  pathways.  Ref: 7493949 J Biol Chem, 1995
- However, inhibition of both  **PKA**  and  **PKC**  prevented PTH 1 34 mediated suppression of Na H exchanger activity, indicating that PTH 1 34 acted through both signaling pathways.  Ref: 7650018 J Biol Chem, 1995
- Since these peptide hormones can stimulate production of diverse second messengersi.e. cAMP and diacylglycerol that activate protein kinase A  **PKA**  and protein kinase C  **PKC**  in target cells, it is conceivable that either one or both of these pathways can participate in modulating exchanger activity.  Ref: 7559463 J Biol Chem, 1995
- Finally, reporter gene assays in HeLa cells treated with either a cAMP analogue or a phorbol ester suggest that the  **PKA** , but not the  **PKC**  signalling pathway is involved in oxygen sensing.  Ref: 8524640 Nucleic Acids Res, 1995
- In renal proximal tubule OK cells, PTH inhibits the activity of the apical membrane Na H exchanger, although it is unclear whether the signal is transmitted through protein kinase A  **PKA**  and or protein kinase C  **PKC** .  Ref: 7650018 J Biol Chem, 1995
- Increases in activity of both protein kinase A  **PKA**  and protein kinase C  **PKC**  contribute to short term facilitation of Aplysia sensorimotor synapses evoked by serotonin 5 HT.  Ref: 7472503 J Neurosci, 1995
- We demonstrate that a VT peptide was specifically phosphorylated by protein kinase C  **PKC**  in vitro, but not by protein kinase A  **PKA** .  Ref: 7559402 J Biol Chem, 1995
- PTHRP 1 16, a biologically inert fragment, was incapable of influencing either the  **PKA**  or  **PKC**  pathway.  Ref: 7559463 J Biol Chem, 1995
- We have used such PC PLC transformed cells to evaluate the roles of the cytoplasmic serine threonine kinases Raf 1, zeta protein kinase C zeta  **PKC**  and protein kinase A  **PKA**  in oncogenesis and mitogenic signal transduction elicited by phosphatidylcholine hydrolysis.  Ref: 7673165 J Biol Chem, 1995
- activation of the PTH receptor can stimulate both the  **PKA**  and  **PKC**  pathways, each of which can independently lead to inhibition of NHE 3 activity.  Ref: 7650018 J Biol Chem, 1995
- The effects of cyclic adenosine monophosphate dependent protein kinase  **PKA**  and calcium dependent protein kinase  **PKC**  modulators on secretagogue stimulated gastric acid secretion were studied in the continuously perfused stomach of the anesthetized rat.  Ref: 8577820 Pharmacology, 1995
- Phosphorylation by  **PKC**  inhibits channel activity regardless of whether the channel was previously phosphorylated by  **PKA** .  Ref: 8569078 Kidney Int, 1995
- These data, together with those obtained in the presence of gonadotropin releasing hormone, provide further support for a hormonally induced interplay between  **PKA**  and  **PKC**  signaling pathways at the crucial level of  **PKA**  in the pituitary gland including gonadotropes.  Ref: 8559283 Neuroendocrinology, 1995
- These results suggest that GnTV may be activated by membranous  **PKC**  or  **PKA** , indirectly or directly, via phosphorylation of Ser Thr residues.  Ref: 8748153 Glycoconj J, 1995
- The results suggest that increases in  **PKA**  and  **PKC**  activities by 5 HT contribute to short minutes and intermediate hours forms of facilitation of sensorimotor synapses while increases in  **PKA**  activity also mediate long term days maintenance of synaptic facilitation.  Ref: 7472503 J Neurosci, 1995
- In contrast, in immortalized cystic fibrosis CF human tracheal epithelial cells CFT 1 and CFT 2, regulation is defective for agonists specific for the  **PKA**  but not for the  **PKC**  pathway.  Ref: 8572248 Am J Physiol, 1995
- In thymocytes, ETOH induced apoptosis was abrogated by chelation of extracellular calcium with EGTA, and inhibition of protein synthesis with CHX, or of  **PKC**  with H7 but not of  **PKA**  with HA 1004.  Ref: 8655290 Immunopharmacology, 1995
- Whereas agonists antagonist of  **PKA**  did not affect permeability and conductance of all gap junction channels, variable changes were observed under  **PKC**  stimulation.  Ref: 8590800 Mol Biol Cell, 1995
- However, each peptide fragment inhibited Na H exchanger activity by 40 50%, suggesting that  **PKC**  and possibly  **PKA**  were capable of transducing the PTH PTHRP signal to the transporter.  Ref: 7650018 J Biol Chem, 1995
- A phosphorylation stoichiometry of 1.94 0.1 32P RyR for  **PKA** , 0.89 0.08 for CamPK II and 0.95 0.16 for  **PKC**  was obtained under these conditions.  Ref: 8529260 Cell Calcium, 1995
- The results showed that  **PKA**  activators and  **PKC**  inhibitors can expand a feather bud domain by enhancing dermal condensation, while  **PKC**  activators and  **PKA**  inhibitors can expand interbud domains.  Ref: 7556946 Dev Biol, 1995
- We have examined the effects of activators and inhibitors of protein kinase A  **PKA** ,  **PKC** , and PKG on permeability and single channel conductance of gap junction channels composed of Cx45, Cx43, or Cx26 subunits.  Ref: 8590800 Mol Biol Cell, 1995
- Phosphorylation of TC vesicles with  **PKA** , CamPK II, or protein kinase C  **PKC**  reduced the calcium loading rate of TC vesicles 3 fold, 2.1 fold and 1.7 fold, respectively, measured in the presence of 1 mM MgCl2.  Ref: 8529260 Cell Calcium, 1995
- Hence, the signal transduction mechanisms underlying the transcriptional activation of alpha 1 IV gene in mesangial cells by high glucose are mediated by pathways involving the  **PKC**  system and possibly the cAMP  **PKA**  system.  Ref: 8573741 J Diabetes ComplicationsJ Diabetes Complications, 1995
- Furthermore, activators or inhibitors of protein kinase A  **PKA** , protein kinase C  **PKC** , or tyrosine kinase do not affect nAChR transcript levels in these cultured neurons.  Ref: 8613734 J Neurosci, 1995
- These findings support a role of  **PKA**  and  **PKC**  in the modulation of stimulated gastric acid secretion in vivo.  Ref: 8577820 Pharmacology, 1995
