# Supplementary material for: Content-rich biological network constructed by mining PubMed abstracts
Source: BMC Bioinformatics. 2004 Oct 8;5:147. doi: 10.1186/1471-2105-5-147 (PMC528731; doi:10.1186/1471-2105-5-147)
Supplement: Additional File 5 — The original Chilibot query results of the term "long-term potentiation (LTP)" and 22 other terms, limiting the latest references analyzed to the years 1990, 1995, 2000, and 2004. [file 1471-2105-5-147-S5.bz2 › chilibotAdditionalFile5/ltp1995/html/PKC_NMDA.html]

 


 **PKC** and **NMDA** 
  
Found 68 abstracts in PubMed,  **30 abstracts were retrieved and analyzed**.  


---

 Search Google  |
 PDF files only 
|  EDU domain only 

---

**Interactive relationship** (e.g. stimulation, inhibition, etc)

- The results indicate that  **PKC**  does not mediate ethanol inhibition of  **NMDA**  receptor function in cerebral cortical cells,.  Ref: 8974336 Alcohol Alcohol Suppl, 1994
- Furthermore, down regulation of  **PKC**  inhibited the increase in TRE binding activity by  **NMDA**  and KA.  Ref: 7616215 J Neurochem, 1995
- Inhibition of  **NMDA**  receptor function by PMA and ethanol could also be overcome by  **PKC**  antagonists, implicating  **PKC**  in the inhibitory effect of ethanol.  Ref: 8974336 Alcohol Alcohol Suppl, 1994
- LysoPC reversibly inhibited the  **NMDA**  gated currents by 43% and this inhibition was not affected by a selective  **PKC**  inhibitor, GF109203X.  Ref: 8554602 Biochem Biophys Res Commun, 1995
- Previous work, using primary cultures of rat cerebellar granule cells, showed that both exposure to alcohol and activation of protein kinase C  **PKC**  by the phorbol ester PMA reduced the potency of the co agonist, glycine, to enhance  **NMDA**  receptor function measured as an increase in intracellular calcium, resulting in inhibition of the  **NMDA**  response at low glycine concentrations.  Ref: 8974336 Alcohol Alcohol Suppl, 1994
- Our findings demonstrate that  **PKC**  is not directly involved in the  **NMDA**  receptor mediated signal transduction processes associated with biochemical differentiation and c fos induction in cerebellar granule neurons.  Ref: 7643961 Neurochem Res, 1995
- We therefore propose that  **PKC**  not only potentiates but is also required for the  **NMDA**  evoked elevation in cytosolic calcium in mouse striatal neurons.  Ref: 8075826 Eur J Neurosci, 1994
- Confirming the dependence on  **PKC**  activity, desensitization of  **PKC**  resulting from long term PMA treatment led to an impairment of the  **NMDA**  response, leaving the KCl induced response intact.  Ref: 8075826 Eur J Neurosci, 1994
- A consequence of  **NMDA**  receptor activation is calcium influx, which in turn can result in translocation of  **PKC**  from cytosol to membrane.  Ref: 8742791 NIDA Res Monogr, 1995
- Such an ischemia induced upregulation which is reported to occur physiologically by the activation of  **PKC** , is reflected by the selective loss of the depressive control of the synaptic  **NMDA**  calcium influx by adenosine.  Ref: 7897401 J Neural Transm Suppl, 1994
- Several isoforms of the  **NMDA**  receptor 1 NR1 subunits of the ionotropic  **NMDA**  N methyl D aspartate glutamate receptor contain a consensus site for phosphorylation by protein kinase C  **PKC**  which, once phosphorylated results in an increased conductance through the receptor channel.  Ref: 7703398 Neuroreport, 1994
- Thus, a common pathway that includes  **PKC**  could, at least in part, be involved in the calcium signaling pathways for the increase in TRE binding activity coupled with the activation of  **NMDA**  and non  **NMDA**  receptors.  Ref: 7616215 J Neurochem, 1995
- Furthermore, in HEK 293 cells co transfected with NR2A and NR1 subunits containing the C terminal exon with the  **PKC**  phosphorylation sites, our preliminary studies indicate that the  **NMDA**  evoked current is potentiated by intracellular  **PKC** .  Ref: 7530547 J Physiol Paris, 1994
- In other experiments, we have demonstrated that the  **NMDA**  receptor subunit NR1 is phosphorylated by  **PKC**  on several distinct sites, and most of these sites are located within a single alternatively spliced exon in the C terminal domain.  Ref: 7530547 J Physiol Paris, 1994

**Parallel relationship** (e.g. studied together, co-existance, homology, etc.)

- These evidences suggest that an appearance of the voltage dependency of INMDA in NTS neurons might be due to a developmental change in combinations of subunits composing the  **NMDA**  receptor and or in the intracellular modulators of the INMDA other than  **PKC** .  Ref: 7922517 Brain Res, 1994
- This observation supports the hypothesis that a retrograde messenger is produced postsynaptically following  **NMDA**  receptor activation and diffuses to the presynaptic terminal to activate  **PKC** .  Ref: 7891109 J Neurochem, 1995
- The present study investigates the roles of protein kinase C  **PKC**  and A PKA activities in  **NMDA**  mediated calcium entry in primary cultures of mouse striatal neurons.  Ref: 8075826 Eur J Neurosci, 1994
- This series of studies has investigated the involvement of the  **NMDA**  receptor and the translocation of  **PKC**  in the seemingly unrelated phenomena of neuropathic pain and tolerance and dependence to narcotic analgesic drugs.  Ref: 8742791 NIDA Res Monogr, 1995
- We are currently examining  **PKC**  effects on the  **NMDA**  evoked current responses of mutant NR1 receptors that lack the C terminal phosphorylation sites.  Ref: 7530547 J Physiol Paris, 1994
- We have now compared the effects of ethanol and  **PKC**  activation of  **NMDA**  receptor function in primary cultures of rat cerebral cortical cells.  Ref: 8974336 Alcohol Alcohol Suppl, 1994
- The present results provide further evidence for a role of  **PKC**  in N methyl D aspartate  **NMDA**  receptor mediated mechanisms of thermal hyperalgesia.  Ref: 8592645 Neurosci Lett, 1995
- These results demonstrate that cyanide stimulates  **PKC**  activation and translocation from the cytosol to membranes in select brain areas and  **NMDA**  receptor activation mediates this process.  Ref: 7853358 J Biochem Toxicol, 1994
- a non  **NMDA**  receptor antagonist, or GM1 ganglioside an intracellular  **PKC**  inhibitor treatment was given to examine the effects of these agents on the development and expression of thermal hyperalgesia in morphine tolerant rats.  Ref: 7908958 J Neurosci, 1994
- This work has demonstrated that the  **NMDA**  receptor and  **PKC**  translocation are importantly involved in neuropathic pain and morphine tolerance or dependence.  Ref: 8742791 NIDA Res Monogr, 1995
- Possible determinants of the differing mechanisms of ethanol s actions include the subunit composition of the  **NMDA**  receptor and or the isoforms of  **PKC**  present in the different cells.  Ref: 8974336 Alcohol Alcohol Suppl, 1994
- The role of protein kinase C  **PKC**  in N methyl D aspartate  **NMDA**  receptor mediated biochemical differentiation and c fos protein expression was investigated in cultured cerebellar granule neurons.  Ref: 7643961 Neurochem Res, 1995
- The specific stimulation of  **NMDA**  receptors in  **PKC**  depleted granule neurons or in the presence of reasonably specific  **PKC**  inhibitors also produced significant elevation in the activity of glutaminase and the expression of c fos protein.  Ref: 7643961 Neurochem Res, 1995
- These results provide further evidence for the involvement of  **PKC**  in  **NMDA**  receptor mediated mechanisms of morphine tolerance.  Ref: 7552251 Brain Res, 1995
- both the development and expression of thermal hyperalgesia in morphine tolerant rats are mediated by central  **NMDA**  and non  **NMDA**  receptors and subsequent protein kinase C  **PKC**  activation.  Ref: 7908958 J Neurosci, 1994
- The  **NMDA**  D serine evoked rise in cytosolic calcium, observed in the absence of external magnesium, was potentiated by the  **PKC**  activator phorbol 12 myristate 13 acetate PMA only when submaximal effective concentrations of this agonist and co agonist were used.  Ref: 8075826 Eur J Neurosci, 1994
- In addition, the  **PKC**  activator did not alter the  **NMDA**  D serine evoked response in the presence of varying concentrations of magnesium.  Ref: 8075826 Eur J Neurosci, 1994
- The protein kinase C  **PKC**  inhibitors staurosporine and calphostin C inhibited the increase in TRE binding activity caused by  **NMDA**  and KA at the same concentration at which they inhibited that caused by TPA.  Ref: 7616215 J Neurochem, 1995
- The amplification of  **NMDA**  toxicity by quisqualate or DHPG was attenuated by a series of protein kinase C  **PKC**  inhibitors, suggesting that class I mGluRs operate, at least in part, through activation of  **PKC** .  Ref: 8532158 Neuropharmacology, 1995
- In this study 32P labeling of non  **NMDA**  GluRs was investigated in cultured hippocampal neurons stimulated 2 15 min with agonists that selectively stimulate either calcium calmodulin dependent protein kinase II CaM kinase II, calcium phospholipid dependent protein kinase C  **PKC** , or cAMP dependent protein kinase A PKA.  Ref: 7509863 J Neurosci, 1994
- These results indicate that lysoPC inhibits  **NMDA**  induced currents by a mechanism independent of PLA2 mediated  **PKC**  activation.  Ref: 8554602 Biochem Biophys Res Commun, 1995
