# Supplementary material for: Content-rich biological network constructed by mining PubMed abstracts
Source: BMC Bioinformatics. 2004 Oct 8;5:147. doi: 10.1186/1471-2105-5-147 (PMC528731; doi:10.1186/1471-2105-5-147)
Supplement: Additional File 5 — The original Chilibot query results of the term "long-term potentiation (LTP)" and 22 other terms, limiting the latest references analyzed to the years 1990, 1995, 2000, and 2004. [file 1471-2105-5-147-S5.bz2 › chilibotAdditionalFile5/ltp1995/html/PKC_AMPA.html]

 


 **PKC** and **AMPA** 
  
Found 17 abstracts in PubMed,  **17 abstracts were retrieved and analyzed**.  


---

 Search Google  |
 PDF files only 
|  EDU domain only 

---

**Interactive relationship** (e.g. stimulation, inhibition, etc)

**Parallel relationship** (e.g. studied together, co-existance, homology, etc.)

- Since Bergmann glia  **AMPA**  kainate receptors are probably mediators of the efficacy of the parallel fiber Purkinje cell synapse, the present findings suggest that the calcium  **PKC**  signalling cascade might play a role in such modulation.  Ref: 7682963 Eur J Pharmacol, 1993
- In the presence of a physiological concentration of magnesium in the extracellular medium, glutamate induces  **PKC**  translocation by binding to both N methyl D aspartate NMDA and alpha amino 3 hydroxy 5 methylisoxazolepropionic acid  **AMPA**  excitatory amino acid receptors.  Ref: 1649249 J Neurochem, 1991
