# Supplementary material for: Content-rich biological network constructed by mining PubMed abstracts
Source: BMC Bioinformatics. 2004 Oct 8;5:147. doi: 10.1186/1471-2105-5-147 (PMC528731; doi:10.1186/1471-2105-5-147)
Supplement: Additional File 5 — The original Chilibot query results of the term "long-term potentiation (LTP)" and 22 other terms, limiting the latest references analyzed to the years 1990, 1995, 2000, and 2004. [file 1471-2105-5-147-S5.bz2 › chilibotAdditionalFile5/ltp1995/html/PKC_TAU.html]

 


 **PKC** and **TAU** 
  
Found 16 abstracts in PubMed,  **16 abstracts were retrieved and analyzed**.  


---

 Search Google  |
 PDF files only 
|  EDU domain only 

---

**Interactive relationship** (e.g. stimulation, inhibition, etc)

- These results prove that the type I, II, and III  **PKC**  are products of  **PKC**  genes,  **tau** , beta, and alpha, respectively.  Ref: 3426619 Biochem Biophys Res Commun, 1987
- By contrast, no significant alteration was observed in AT8 and  **Tau**  1 immunofluorescence following either  **PKC**  or PKM microinjection.  Ref: 8600297 J Neurosci Res, 1995
- Immunoblot analysis revealed that the expressed  **PKC**  in COS cells transfected with either alpha, beta, or  **tau**  cDNA of  **PKC**  were recognized by specific antibodies against the type III, II, and I  **PKC**  isozymes, respectively.  Ref: 3426619 Biochem Biophys Res Commun, 1987
- Here we show that the sites of phosphorylation by four kinases PKA,  **PKC** , CK and CaMK all lie in the C terminal microtubule binding half of  **tau** , but only the phosphorylation by CaM kinase shows the pronounced shift in electrophoretic mobility characteristic for  **tau**  from Alzheimer neurofibrillary tangles.  Ref: 2120043 EMBO J, 1990

**Parallel relationship** (e.g. studied together, co-existance, homology, etc.)

- Changes in the in vitro phosphorylation pattern of three other non  **PKC**  substrates in Alzheimer s disease, including one with characteristics similar to microtubule associated protein  **tau** , are also reported.  Ref: 1827625 Exp Neurol, 1991
- TTX R current activation  **tau**  m was faster and current inactivation  **tau**  h changed from a single to a bi exponential after PMA exposure, suggesting that  **PKC**  phosphorylation may have activated formerly quiescent Na channels.  Ref: 8229203 J Neurosci, 1993
- However, higher concentrations of ALCl3 inhibited the  **tau**  phosphorylation with P34, PKP, and  **PKC**  to a maximum at 1 mM level.  Ref: 8270765 J Environ Sci Health B, 1993
- Western blot analysis showed no major changes in pituitary  **PKC**  alpha,  **PKC**  beta and  **PKC**  zeta when 6 day old and 3 month old female rats were compared, while  **PKC**   **tau**  was not detected.  Ref: 7958391 Mol Cell Endocrinol, 1994
- ...  **PKC**   **tau**  and  **PKC**  delta increased markedly during this period.  Ref: 7958391 Mol Cell Endocrinol, 1994
- Immunocytochemical studies of rat cerebellum using specific antibodies against type I, II, and III  **PKC**  revealed the presence of the type I  **PKC**  in the Purkinje cells where transcripts of  **tau**  cDNA were localized, the type II  **PKC**  in the granule cells where transcripts of beta cDNA were detected, and the type III  **PKC**  in both the Purkinje and granule cells.  Ref: 3426619 Biochem Biophys Res Commun, 1987
- Multiple  **PKC**  isozymes such as conventional  **PKC**  cPKC alpha and gamma, new  **PKC**  nPKC epsilon, and atypical  **PKC**  aPKC zeta, lambda, and  **tau**  were expressed in chondroblasts but cPKC beta and nPKC delta were not detected.  Ref: 7488176 Biochem Biophys Res Commun, 1995
