# Supplementary material for: Content-rich biological network constructed by mining PubMed abstracts
Source: BMC Bioinformatics. 2004 Oct 8;5:147. doi: 10.1186/1471-2105-5-147 (PMC528731; doi:10.1186/1471-2105-5-147)
Supplement: Additional File 5 — The original Chilibot query results of the term "long-term potentiation (LTP)" and 22 other terms, limiting the latest references analyzed to the years 1990, 1995, 2000, and 2004. [file 1471-2105-5-147-S5.bz2 › chilibotAdditionalFile5/ltp1995/html/PKC_CAMKII.html]

 


 **PKC** and **CAMKII** 
  
Found 22 abstracts in PubMed,  **22 abstracts were retrieved and analyzed**.  


---

 Search Google  |
 PDF files only 
|  EDU domain only 

---

**Interactive relationship** (e.g. stimulation, inhibition, etc)

- **PKC**  19 36 was 30 fold more potent than Ala286  **CaMKII**  281 302.  Ref: 8197132 Proc Natl Acad Sci U S A, 1994

**Parallel relationship** (e.g. studied together, co-existance, homology, etc.)

- Inhibition of postsynaptic  **PKC**  or  **CaMKII**  blocks induction but not expression of LTP.  Ref: 2549638 Science, 1989
- Previous studies have used synthetic peptide analogs, corresponding to sequences within the pseudosubstrate domain of protein kinase C  **PKC**  or the autoregulatory domain of calcium calmodulin dependent protein kinase II  **CaMKII** , in attempts to define the contribution of each of these protein kinases to induction of long term potentiation LTP.  Ref: 8197132 Proc Natl Acad Sci U S A, 1994
- Synthetic peptides corresponding to the autoinhibitory domains of calcium calmodulin dependent protein kinase II  [ **CAMKII** ]  CaMK 281 309, smooth muscle myosin light chain kinase MLCK 480 501, and protein kinase C  **PKC**  19 36 as well as a peptide derived from the heat stable inhibitor of cAMP dependent protein kinase PKI tide were tested for their inhibitory specificities.  Ref: 2153665 J Biol Chem, 1990
- Thus both postsynaptic  **PKC**  and  **CaMKII**  are required for the induction of LTP and a presynaptic protein kinase appears to be necessary for the expression of LTP.  Ref: 2549638 Science, 1989
- The studies showed 1 endogenous membrane bound  **CaMKII**  and  **PKC**  as well as exogenous, highly purified  **PKC**  inhibit proline uptake by phosphorylated, lyzed resealed BBMV when compared with control vesicles.  Ref: 8251336 Pediatr Nephrol, 1993
- The role of several biological molecules in learning and memory are considered, for example, protein kinase C  **PKC** , Ca Calmodulin kinase II  **CaMKII** , GAP 43, and glutamate receptors.  Ref: 8569741 Mol Cell BiochemMol Cell Biochem, 1993
- To assess the involvement of protein kinases in the Ca dependent stimulation of Cl conductance, we employed pseudosubstrate peptide inhibitors of protein kinase C  **PKC**  and the Ca CaM dependent protein kinase II  **CaMKII** .  Ref: 1708204 Am J Physiol, 1991
- Strikingly, established LTP was not suppressed by a combination of  **PKC**  and  **CaMKII**  blocking peptides, or by intracellular postsynaptic H 7.  Ref: 1327679 Ciba Found Symp, 1992
- We found that long lasting synaptic enhancement was prevented by prior intracellular injection of potent and selective inhibitory peptide blockers of either protein kinase C  **PKC**  or calcium calmodulin dependent protein kinase II  **CaMKII** , such as  **PKC**  19 31 or  **CaMKII**  273 302, but not by control peptides.  Ref: 1327679 Ciba Found Symp, 1992
- We investigated 1 the effect of calcium and phospholipid dependent protein kinase protein kinase C  **PKC**  and calcium calmodulin dependent protein kinase II  **CaMKII**  on sodium chloride NaCl linked proline transport by renal brush border membrane vesicles BBMV from adult rats using the hypoosmotic shock technique lysis of vesicles.  Ref: 8251336 Pediatr Nephrol, 1993
- Using intracellular delivery to rat CA1 hippocampal neurons, we have determined the relative potency of two protein kinase inhibitor peptides,  **PKC**  19 36 and Ala286  **CaMKII**  281 302, as inhibitors of the induction of LTP.  Ref: 8197132 Proc Natl Acad Sci U S A, 1994
- Both  **PKC**  and  **CaMKII**  are possible sources of the persistent kinase activities.  Ref: 8063002 Int J Biochem, 1994
- This study analyzed the ability of the N methyl D aspartate receptor antagonist dextrorphan DX to prevent neuronal degeneration analyzed by light microscopy, calmodulin CaM redistribution analyzed by immunocytochemistry and changes in activity of two major calcium dependent protein kinases calcium calmodulin dependent protein kinase II  [ **CAMKII** ]  CaM KII and protein kinase C  **PKC**  analyzed by specific substrate phosphorylation after 20 min of global ischemia four vessel occlusion model in rats.  Ref: 7686173 J Cereb Blood Flow Metab, 1993
- Protein kinases other than  **CaMKII**  or  **PKC**  ex.  Ref: 8063002 Int J Biochem, 1994
- Induction of LTP is blocked by intracellular delivery of H 7, a general protein kinase inhibitor, or  **PKC**  19 31, a selective protein kinase C  **PKC**  inhibitor, or  **CaMKII**  273 302, a selective inhibitor of the multifunctional calcium calmodulin dependent protein kinase  **CaMKII** .  Ref: 2549638 Science, 1989
- calcium calmodulin dependent protein kinase II  **CaMKII**  and protein kinase C  **PKC**  may play pivotal roles in the different phases of the expression of LTP.  Ref: 8063002 Int J Biochem, 1994
- Postsynaptic injection of CA2 CaM induces synaptic potentiation requiring  **CaMKII**  and  **PKC**  activity.  Ref: 7646896 Neuron, 1995
- Pseudosubstrate inhibitors or high affinity substrates of  **CaMKII**  or  **PKC**  blocked calcium CaM induced potentiation, indicating the requirement of  **CaMKII**  and  **PKC**  activities in synaptic potentiation.  Ref: 7646896 Neuron, 1995
- The enzyme responsible for the production of NO, nitric oxide synthase NOS, is phosphorylated by protein kinase C  **PKC** , the cAMP dependent protein kinase PKA, and the calcium calmodulin dependent protein kinase II  [ **CAMKII** ]  CaM II.  Ref: 8230323 J Neurosci Res, 1993
- 2 the activity, expression and subcellular distribution cytosol, particulate, BBM of calcium dependent protein kinases in kidneys from 7 day old and adult rats using MBP 4 14 and autocamtide II phosphorylation assays for  **PKC**  and  **CaMKII** , respectively, endogenous protein phosphorylation using gel electrophoresis and autoradiography and Western immunoblot analysis to detect  **PKC**  and  **CaMKII** .  Ref: 8251336 Pediatr Nephrol, 1993
- Involvement of protein phosphorylation in LTP has been widely proposed, with protein kinase C  **PKC**  and calcium calmodulin kinase type II  **CaMKII**  as leading candidates.  Ref: 2847049 Nature, 1988
- the voltage clamped, nonelectrogenic component of proline transport was inhibited by  **PKC**  but not  **CaMKII**  mediated phosphorylation.  Ref: 8251336 Pediatr Nephrol, 1993
- The relative specificity of  **PKC**  19 36, Ala286  **CaMKII**  281 302, and several other  **CaMKII**  peptide analogs for protein kinase inhibition in vitro was also determined.  Ref: 8197132 Proc Natl Acad Sci U S A, 1994
- A comparison of the potencies of  **PKC**  19 36 and Ala286  **CaMKII**  281 302 in the physiological assay with their Ki values for protein kinase inhibition in vitro indicates that the blockade of induction of LTP observed for each peptide is attributable to inhibition of  **PKC** .  Ref: 8197132 Proc Natl Acad Sci U S A, 1994
- Evidently, activity of both  **PKC**  and  **CaMKII**  is somehow necessary for the postsynaptic induction of LTP.  Ref: 1327679 Ciba Found Symp, 1992
